# Photocaged 5′ cap analogues for optical control of mRNA translation in cells

**DOI:** 10.1038/s41557-022-00972-7

**Published:** 2022-06-20

**Authors:** Nils Klöcker, Florian P. Weissenboeck, Melissa van Dülmen, Petr Špaček, Sabine Hüwel, Andrea Rentmeister

**Affiliations:** Institute of Biochemistry, Westfälische Wilhelms-Universität Münster, Münster, Germany

**Keywords:** RNA, Translation, Chemical modification

## Abstract

The translation of messenger RNA (mRNA) is a fundamental process in gene expression, and control of translation is important to regulate protein synthesis in cells. The primary hallmark of eukaryotic mRNAs is their 5′ cap, whose molecular contacts to the eukaryotic translation initiation factor eIF4E govern the initiation of translation. Here we report 5′ cap analogues with photo-cleavable groups (FlashCaps) that prohibit binding to eIF4E and resist cleavage by decapping enzymes. These compounds are compatible with the general and efficient production of mRNAs by in vitro transcription. In FlashCap-mRNAs, the single photocaging group abrogates translation in vitro and in mammalian cells without increasing immunogenicity. Irradiation restores the native cap, triggering efficient translation. FlashCaps overcome the problem of remaining sequence or structure changes in mRNA after irradiation that limited previous designs. Together, these results demonstrate that FlashCaps offer a route to regulate the expression of any given mRNA and to dose mRNA therapeutics with spatio-temporal control.

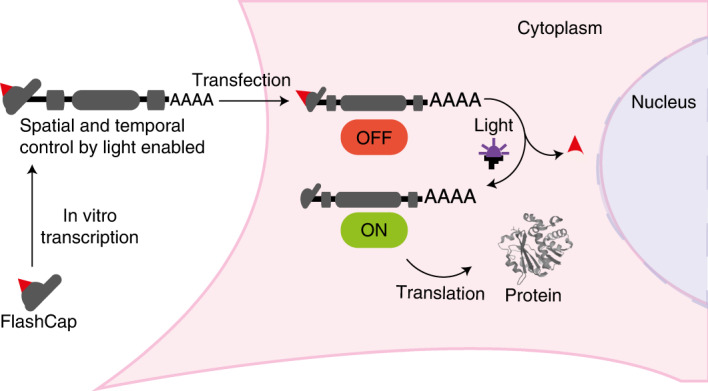

## Abstract (web summary)

Analogues of mRNA 5′ caps containing a photo-cleavable group have now been developed. These so-called FlashCaps can be used for routine in vitro transcription to make long mRNAs containing a cap. In cells, the capped mRNAs are translationally muted; however, upon irradiation by light, the photo-cleavable group is removed without leaving any remaining modification and mRNA is then translated into the corresponding protein.

## Main

**Fig. 1:**
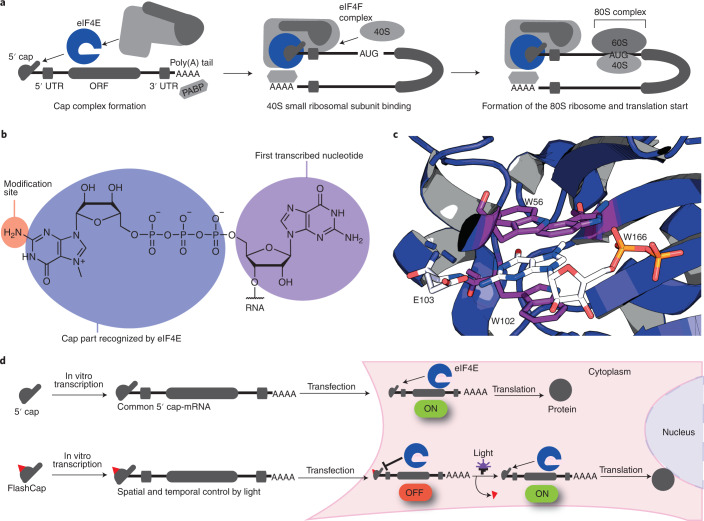
The 5′ cap is a hallmark of eukaryotic mRNAs governing translation initiation. **a**, Key steps in translation initiation. The eukaryotic translation initiation factor eIF4E binds directly to the 5′ cap. The heterotrimeric eIF4F complex assembles on the 5′ cap, leading to binding of the 40S ribosomal subunit, assembly of the eukaryotic 80S ribosome and translation initiation. **b**, Eukaryotic mRNA featuring the cap 0 structure with a recognition site for eIF4E, the site used for chemical modification in this study and the first transcribed nucleotide. **c**, Structure of eIF4E, highlighting molecular interactions for cap 0 recognition. **d**, The concept of FlashCaps for light-induced translation. A single photo-cleavable group (red triangle) at the cap 0 impairs binding to eIF4E. FlashCaps are compatible with routine protocols for transcription and transfection. Following light-induced deprotection, the native mRNA with a 5′ cap 0 is released and translated. UTR, untranslated region; PABP, poly(A) binding protein; ORF, open reading frame.

Messenger RNAs (mRNAs) have recently entered the public stage as most versatile medical modalities. Prominent examples are the mRNA-based vaccines by Moderna and BioNTech/Pfizer that code for spike proteins to protect against infection by SARS-CoV-2 (ref. ^1^). The mRNA technology is not limited to vaccination, and can also greatly improve, for example, therapy for autoimmune diseases or personalized cancer treatment^2^. Translation of mRNA into proteins is one of the fundamental and highly conserved processes in the cell and works for endogenous and exogenous transcripts (Fig. 1a). Its regulation is essential in cell differentiation, cell proliferation and localized translation^3,4^, but is also relevant for pathologies^5^. In mRNA therapy, however, one cannot currently control when and where mRNA has an effect, that is, when and where it is translated into proteins, which then have a pharmacological effect.

A hallmark of eukaryotic mRNAs is their 5′ cap, which, in its simplest form (cap 0), links an *N*7-methylated guanosine to the first transcribed nucleotide via a 5′-5′ triphosphate bridge (Fig. 1b). Higher-order cap structures contain additional methyl groups^6^. The 5′ cap plays a key role in translation initiation, as the *N*7-methylated guanosine is essential for recognition by the translation initiation factor eIF4E (Fig. 1c). Importantly, the molecular contacts with the cap are sequence-independent, that is, they are identical for all mRNAs^7^. The 5′ cap is also crucial for many mRNA processing and quality control steps and protects eukaryotic mRNAs from degradation by exonucleases^3,5,8^. Dedicated decapping enzymes (Dcp1-2, DcpS) are required for mRNA turnover and homeostasis^9,10^. Together with the poly(A) tail at the 3′ end, the 5′ cap forms an mRNA ‘closed-loop’, facilitated by interactions between the cap-binding eIFs and the poly(A)-binding protein (PABP). The closed loop promotes recruitment of the small ribosomal subunit (40S) and the complex enters the next initiation stages, leading to formation of the 80S ribosome and translation (Fig. 1a)^5^. RNA without the 5′ cap is barely translated and is highly immunogenic^11–13^. Therefore, production of mRNAs for biological studies and therapeutic applications routinely involves in vitro transcription in the presence of synthetic cap analogues to obtain 5′-capped mRNAs^14–16^ (Fig. 1d).

In nature, the initiation phase of translation is the target of multiple types of regulatory intervention, enabling confinement of gene expression to a certain time span and cell region, for example, in neurons or multicellular organisms^5,17^. The ability to control translation by external triggers—especially by light—would greatly enrich our ability to dissect cellular processes at the molecular level with high spatio-temporal precision. The directed release of mRNAs for translation at a certain time and destination would also provide an avenue to control the pharmacokinetics of mRNA therapeutics. In this context, it would be important to avoid a drastic increase of immunogenicity.

However, methods to control gene expression externally at the mRNA level are scarce. Natural mechanisms triggering mRNA translation by light are still unknown, and only one example of integrating photo-sensitive units in translation has been reported so far^18^. Chemical approaches to directly photocage RNAs provide control of several RNA-regulated processes involving short regulatory RNAs, such as small interfering RNAs, microRNAs, morpholinos and aptamers^19–23^. The chemical or chemo-enzymatic synthesis of long mRNAs, however, suffers from low yields^24^. Moreover, the installation of multiple modifications in the mRNA does not necessarily impede the ribosome^25^. Previous approaches towards controlling mRNA translation by light required tags^26^, multiple photocaging groups^27^ or photoswitches^28–31^—including photoswitches at the 5′ cap—that left the RNA altered. Remaining chemical modifications in the mRNA might affect the properties of the mRNA, as shown for natural modifications^32,33^. Additional sequence elements may alter mRNA interactions, potentially disrupting the regulatory processes of mRNA turnover^34^.

In this Article we report optochemical control of mRNA translation in eukaryotic cells. Our approach is based on a synthetic cap analogue (FlashCap) that efficiently interferes with the initiation stage of translation. Irradiation of FlashCap-mRNAs liberates an unaltered cap 0-mRNA molecule that is accessible for translation into hundreds of protein copies (Fig. 1a,d). This concept capitalizes on a single photocaging group at a defined position to leverage strong effects on the translation of ~1,000-nt-long mRNAs. It is generally applicable, as synthetic 5′ cap analogues are routinely used in the production of mRNAs by in vitro transcription for research and therapeutic purposes. FlashCaps are therefore an efficient and readily applicable solution to make mRNA studies controllable by light, without requiring new production steps and without introducing artefacts into measurements.

## Results

**Fig. 2:**
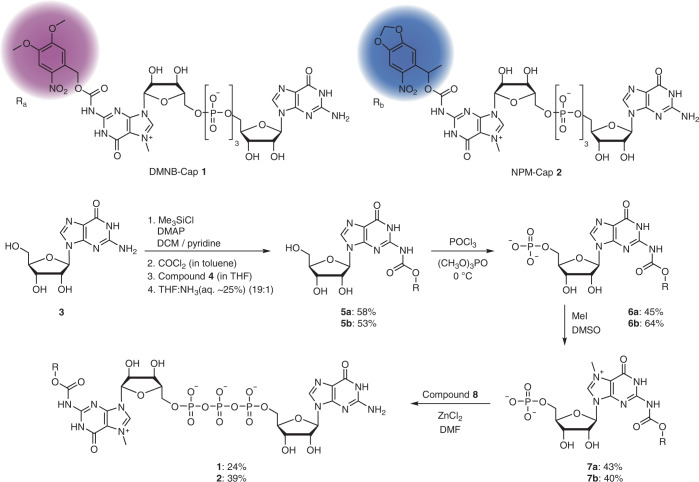
A general strategy for synthesizing cap analogues with photo-cleavable groups (FlashCaps) for triggering translation by light. The self-immolative carbamate linkage ensures efficient light-mediated release of cap 0. Details are provided in Extended Data Fig. 1.

To achieve a strong effect on translation, we analysed the molecular interactions between the 5′ cap and the translation initiation factor eIF4E (Fig. 1c), as well as previous work on the effect of cap modifications on binding^16,28,35–37^. We anticipated that the installation of a sterically demanding residue (such as a photo-cleavable group) at the N^2^ position of the guanosine should interfere with the direct hydrogen bonding to E103 that is required for proper positioning of the 5′ cap (Fig. 1c). At the same time, photo-deprotection should rapidly reconstitute the natural cap 0 and initiate translation. We therefore developed a synthesis route to 5′ caps with photo-cleavable groups at the N^2^ position of the cap guanosine (Fig. 2). To promote cap 0 release, we connected the photo-cleavable group via a self-immolative carbamate linkage. Photo-cleavage releases CO_2_, driving the deprotection reaction. Starting from guanosine (**3**), we first protected the three hydroxyl groups using trimethylsilyl (TMS) chloride. In a one-pot reaction, we then converted the free amino group of the guanosine to isocyanate, which was directly reacted with the *ortho*-nitrobenzyl (ONB) alcohol **4c** as the photo-cleavable group or the redshifted derivatives 3,4-dimethoxy-2-nitrobenzyl (DMNB), 6-nitropiperonyl (NP) or 6-nitropiperonyl-methyl (NPM) alcohol (**4a–d**; Supplementary Fig. 22). During workup in THF with aqueous ammonia, the TMS groups were removed to obtain the photocaged guanosines, **5a–d**. The photocaged guanosines were then monophosphorylated at the 5′-OH to give **6a**,**b**, methylated to **7a**,**b** and coupled to guanosine-5′-diphosphate imidazolide, prepared from guanosine diphosphate (GDP) as previously described^38^.

**Fig. 3:**
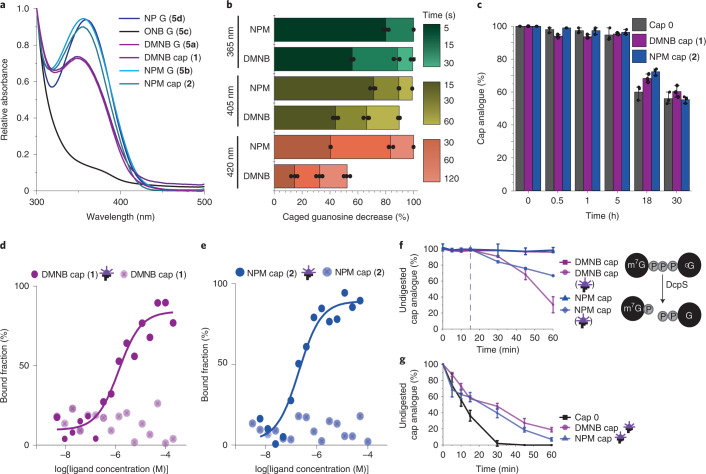
Characterization of FlashCaps. **a**, Absorption spectra of the indicated guanosines and cap analogues. **b**, Time- and wavelength-dependent photo-deprotection for the indicated caged guanosines. **c**, Stability of FlashCaps in cell lysate at 37 °C in comparison to cap 0. **d**,**e**, Affinity measurements of FlashCaps **1** (**d**) and **2** (**e**) before and after irradiation with eIF4E (Cy5-labelled) using MST. The average of three independent measurements is shown. **f**, Stability of FlashCaps against enzymatic degradation by DcpS with or without irradiation at 15 min (dashed line) (365 nm, 30 s). **g**, Stability of irradiated FlashCaps against enzymatic degradation by DcpS (365 nm, 30 s) in comparison to cap 0. Data of *n* = 3 independent experiments are shown as mean values ± s.d. Source data

We measured the absorption spectra of the synthesized guanosines with photo-cleavable groups at the N^2^ position (**5a–d**). ONB guanosine (**5c**) showed only low absorbance above 300 nm, DMNB guanosine (**5a**) showed an absorption maximum at 350 nm, and NP (**5d**) and NPM (**5b**) guanosine were slightly redshifted with a maximum at 360 nm (Fig. 3a), in line with literature on the respective photo-cleavable groups^20,39,40^.

**Extended Data Fig. 1:**
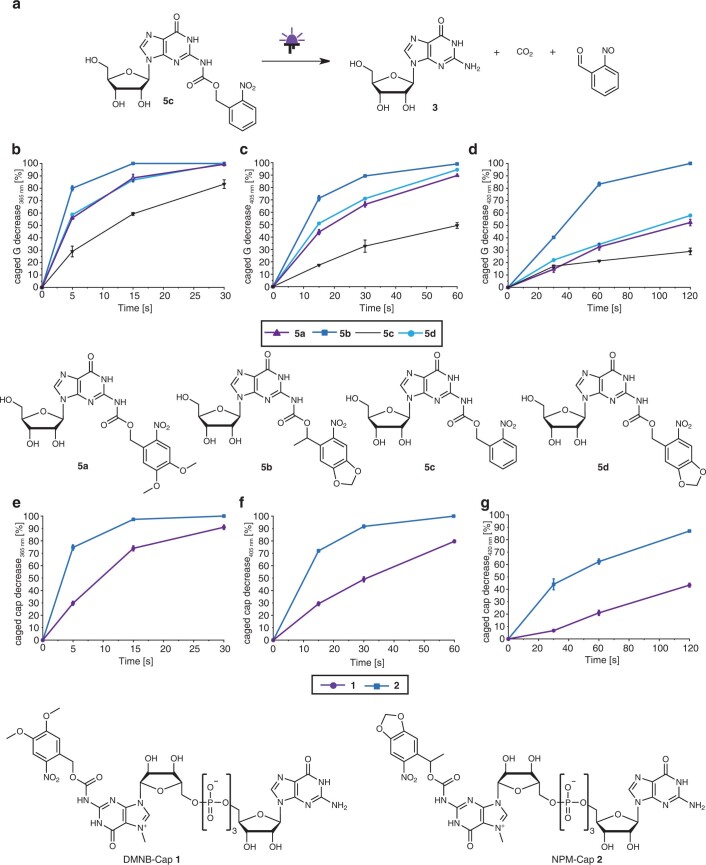
Irradiation studies of photocaged guanosines and FlashCaps. Photo-cleavage reaction by the example of **5c** (**a**). Illustration of the decrease of caged guanosine (G) after irradiation with 365 nm (**b**), 405 nm (**c**) or 420 nm (**d**) and the decrease of FlashCaps after irradiation with 365 nm (**e**), 405 nm (**f**) or 420 nm (**g**) for various periods of time. The samples (500 µM) were analysed via HPLC after irradiation via LED (365 nm (142 mW/cm^2^), 405 nm (142 mW/cm^2^); 420 nm (60 mW/cm^2^)). The percentage of uncaged guanosine or FlashCap was calculated by integration of the resulting peaks. Data points and error bars denote mean values ± standard deviation for *n* = 3 independent replicates. Source data

To choose the most suitable photo-cleavable group for biological applications, we irradiated **5a–c** in aqueous solution at neutral pH and analysed their decrease as well as formation of the native guanosine using HPLC and liquid chromatography mass spectrometry (LC-MS; Fig. 3b, Extended Data Fig. 1 and Supplementary Fig. 3). Time-dependent analyses revealed that at 365 nm (light-emitting diode (LED), 140 mW cm^−2^), short irradiation (5–15 s) was sufficient to remove the photo-cleavable group in 10 µl of a 500 µM solution of **5a**,**b**,**d** and release the free guanosine (Fig. 3b and Extended Data Fig. 1). At 405 nm, the NP, NPM and DMNB groups were efficiently removed after 60 s, more efficiently than the ONB group (Fig. 3b and Extended Data Fig. 1). At 420 nm, the NPM group was completely removed after 120 s (Fig. 3b and Extended Data Fig. 1). We therefore chose DMNB and NPM groups for further studies and synthesized the respective cap 0 analogues. The resulting FlashCaps contain the DMNB (**1**) or the NPM (**2**) group at the N^2^ position connected via a carbamate functionality (Fig. 2). Their absorption spectra above 300 nm and their uncaging kinetics were similar to the respective photocaged guanosines (**5a**,**b**) (Fig. 3a and Extended Data Fig. 1) and formation of cap 0 was confirmed (Supplementary Figs. 7–10). We also assessed the biological stability of the carbamate linkage by incubating cap 0 (**0**) or FlashCaps (**1**, **2**) in cell lysate followed by HPLC analysis (Fig. 3c and Supplementary Fig. 4). FlashCaps exhibited high stabilities over 30 h, similar to the cap 0, suggesting that the carbamate linkage is not the primary point of degradation in lysate.

Next, we evaluated how the photo-cleavable groups affect interaction of the 5′ cap with eIF4E. Binding measurements of FlashCaps and Cy5-labelled eIF4E using microscale thermophoresis (MST) did not result in a binding curve in the case of the photocaged caps (**1**, **2**) (Fig. 3d,e). Under identical conditions, a *K*_d_ value of 0.3 µM was determined for cap 0 (Supplementary Fig. 5), in line with the literature^35,41^. Importantly, after light-induced removal of the photocaging groups from **1** or **2**, the characteristic binding curve and a *K*_d_ value in a similar range to cap 0 was obtained (Fig. 3d,e), indicating efficient formation of cap 0 (Supplementary Table 2).

We also investigated how the photo-cleavable groups affected interactions with cap-modifying enzymes. DcpS is a pyrophosphatase hydrolysing the cap structure to m^7^GMP and GDP in eukaryotic cells (Fig. 3f)^8^. Similar to the results with eIF4E, DcpS (H277N)—a binding but non-cleaving variant of the decapping enzyme—interacted with cap 0 but not with FlashCaps (Supplementary Fig. 6). However, if FlashCaps were briefly irradiated before the assay was performed, the *K*_d_ value of the DcpS variant was in the same range as for cap 0 (Supplementary Fig. 6), indicating light-induced liberation of functional cap 0.

We also tested whether the photo-cleavable groups would affect the enzymatic degradation of cap structures (Fig. 3f,g). Catalytically active DcpS-WT rapidly cleaved cap 0 into m^7^GMP and GDP, resulting in >50% degradation within 15 min (Fig. 3g)^8^. In contrast, FlashCaps **1** and **2** remained almost completely intact during that time (~98% undigested cap), demonstrating that the photo-cleavable groups abrogate enzymatic cleavage of FlashCaps (Fig. 3f). As expected, the DcpS-mediated cleavage of FlashCaps was triggered in situ by irradiation with light (365 nm, 30 s), confirming that light-mediated release of the photo-cleavable group renders the reconstituted cap 0 readily available to enzymatic conversion (Fig. 3f).

Taken together, these data demonstrate that FlashCaps efficiently impede the interaction with cap-binding proteins and cap-degrading enzymes and that irradiation by light releases fully functional cap 0 that is readily recognized by cap-binding partners in vitro.

**Fig. 4:**
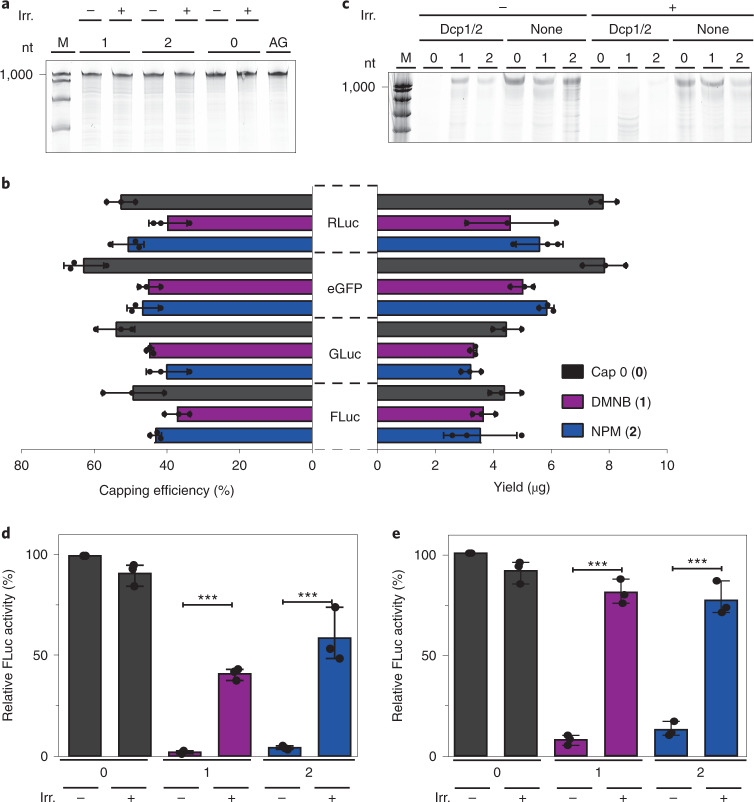
Characterization of cap-caged mRNA. **a**, PAGE analysis of mRNA from IVT with commercial caps (0, AG) or FlashCaps (**1**, **2**). Shown is one representative gel from *n* = 3 independent replicates. **b**, Yield and capping efficiency of the indicated mRNAs from IVT (50 µl) in the presence of FlashCaps. **c**, Stability of differently capped (**0**, **1**, **2**) mRNA against Dcp1/2 before and after irradiation. Samples were either incubated with enzyme (Dcp1/2) or without (none). Shown is one representative gel from *n* = 3 independent replicates. **d**, In vitro translation of FlashCap-FLuc-mRNA before and after irradiation. Data of *n* = 3 independent experiments are shown as mean values ± s.d. Statistical significance was determined by two-tailed Student's *t*-test. Significance levels were defined as *P < 0.05, **P < 0.01, ***P < 0.001. The *P* value for **2** (+) versus **2** (−) is 9.08 × 10^−4^. The *P* value for **1** (+) versus **1** (−) is 2.3 × 10^−5^. **e**, Same as **d**, but with modified nucleotides (m^1^Ψ, m^5^C). Data of *n* = 3 independent experiments are shown as mean values ± s.d. Statistical significance was determined by two-tailed Student's *t*-test. Significance levels were defined as *P < 0.05, **P < 0.01, ***P < 0.001. The *P* value for **2** (+) versus **2** (−) is 2.69 × 10^−4^. The *P* value for **1** (+) versus **1** (−) is 4.3 × 10^−5^. nt, nucleotide; irr., irradiated (365 nm, 30 s); M, Marker; AG, ApppG cap; FLuc, Firefly luciferase; RLuc, Renilla luciferase; GLuc, Gaussia luciferase; eGFP, enhanced green fluorescent protein. Source data

Next, we were interested in whether FlashCaps are suitable for the preparation of long mRNAs containing a photocaged 5′ cap (FlashCap-mRNAs) using standard molecular biology methods. In vitro transcription (IVT) using phage T7 RNA polymerase and synthetic cap analogues is routinely used to produce capped mRNAs for biological studies^42^ and therapeutic applications^43^. The cap analogue is incorporated as the first G by transcriptional priming, yielding capped and uncapped RNA. The latter can be removed by enzymatic treatment with polyphosphatase and XRN1 (ref. ^16^). Comparative evaluation of IVT with FlashCaps or cap 0 revealed that all tested 5′ caps yielded intact mRNAs (Fig. 4a). The yield and capping efficiency in the presence of **1** or **2** were slightly lower but in the same range as for cap 0, according to our analysis of four different mRNAs (Fig. 4b). These data show that transcriptional priming with FlashCaps is efficient and that **1** and **2** can be routinely used for IVT with T7 polymerase to produce long FlashCap-mRNAs with yields comparable to cap 0.

We then probed the interaction of long FlashCap-mRNAs with cap-binding proteins or cap-modifying enzymes. In the major mRNA turnover pathway, the decapping enzyme Dcp1/2 cleaves mRNA to release the 5′ monophosphorylated mRNA, which is degraded by the exoribonuclease XRN1 (refs. ^9,10^). We tested this cap-dependent decay in vitro by treating cap 0-mRNA and FlashCap-mRNA with Dcp1/2 followed by XRN1 digestion (Fig. 4c). This treatment completely degraded cap 0-mRNA, whereas FlashCap-mRNAs with **1** or **2** remained intact (Fig. 4c). When FlashCap-mRNAs were irradiated before the enzymatic treatment, they became susceptible to enzymatic degradation, indicating light-dependent release of the free cap 0, which is recognized by Dcp1/2. In control reactions, which were irradiated but not treated with the enzymes, mRNAs with cap 0 or FlashCaps remained intact, confirming that irradiation alone does not degrade long mRNAs (Fig. 4c).

As FlashCaps abrogate eIF4E binding, which is the rate-limiting step for translation initiation, we were curious as to how FlashCap-mRNA would impact translation. We therefore tested in vitro translation (IVTL) of luciferase-mRNAs with cap 0 and FlashCaps using rabbit reticulocyte lysate. To our delight, the translation of FlashCap-mRNAs was drastically reduced (Fig. 4d), in line with results from our eIF4E-binding studies (Fig. 3d,e). FlashCap-RLuc-mRNAs with **1** or **2** exhibited only 2–4% of luciferase activity relative to cap 0-mRNA (Fig. 4d). However, if the FlashCap-mRNAs were irradiated, translation was increased by 15–20-fold, reaching 41 ± 2% (**1**) (s.d., *n* = 3) or 59 ± 11% (**2**) (s.d., *n* = 3) relative to the native cap 0. Under the same irradiation conditions, the IVTL of cap 0-mRNAs was only slightly reduced (to 91 ± 5%; s.d., *n* = 3) and the mRNAs remained intact (Fig. 4a,d).

Taken together, these data demonstrate that FlashCap-mRNAs are translationally muted and efficiently activated by brief irradiation with light. The released mRNAs are intact, functional and contain a 5′ cap 0, but no sequence changes or remaining chemical modifications.

**Fig. 5:**
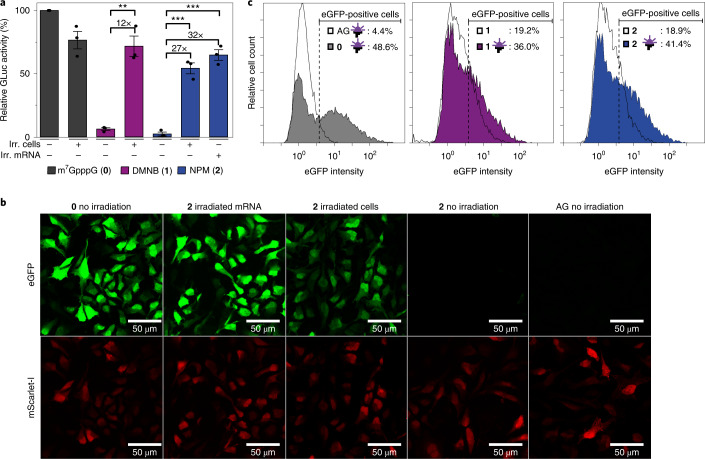
Light-induced translation in cells. **a**, Relative luciferase activity from HeLa cells transfected with differently capped GLuc-mRNAs. The mRNA was capped with the indicated cap analogue. Data of *n* = 3 independent experiments are shown as mean values ± s.e.m. Statistical significance was determined by two-tailed Student's *t*-test. Significance levels were defined as *P < 0.05, **P < 0.01, ***P < 0.001. The *P* value for **1** (+ irr. cells, − irr. mRNA) versus **1** (− irr. cells, − irr. mRNA) is 1.26 × 10^−3^. The *P* value for **2** (+ irr. cells, − irr. mRNA) versus **2** (− irr. cells, − irr. mRNA) is 3.53 × 10^−4^. The *P* value for **2** (− irr. cells, + irr. mRNA) versus **2** (− irr. cells, − irr. mRNA) is 1.52 × 10^−4^. **b**, Confocal laser scanning microscopy images of HeLa cells co-transfected with differently capped eGFP-mRNAs and cap 0-mScarlet-I-mRNA. mRNAs contain m^5^C and m^1^Ψ. AG: ApppG-capped mRNA represents cap-independent translation. (**0**): m^7^GpppG-capped eGFP-mRNA. **2**: NPM capped-eGFP-mRNA, either non-irradiated, irradiated in cells or irradiated before transfection (irradiated mRNA). The top row shows the eGFP fluorescence and the bottom row the mScarlet-I fluorescence. Scale bars, 50 µm. For all images, background subtraction was performed with ImageJ (30 pixels). Shown is one representative set of *n* = 3 independent experiments. **c**, Flow cytometry of HeLa cells transfected with differently capped mRNAs (with cap analogues **0**, **1**, **2** or ApppG (AG)). Untransfected cells are set as gate for eGFP-negative cells. Irradiation is indicated by an LED icon. Shown is one representative measurement of *n* = 3 independent experiments. Source data

Next we investigated the translation of FlashCap-mRNAs in cultured mammalian cells using *Gaussia* luciferase (GLuc) or enhanced green fluorescent protein (eGFP) as the secreted or intracellular reporter. Luciferase activity for HeLa cells transfected with FlashCap-mRNAs or controls was normalized to cap-dependent translation of cap 0-mRNA. The cap-dependent translation of FlashCap-mRNAs with **1** or **2** was reduced to 6 ± 1% (s.e.m., *n* = 3) and 2 ± 2% (s.e.m., *n* = 3), respectively (Fig. 5a). Half of the cell samples were briefly irradiated 6 h after transfection. Irradiation strikingly increased the luciferase signal of cells transfected with FlashCap-mRNA, resulting in 72 ± 8% (s.e.m., *n* = 3) in the case of **1** and 54 ± 4% (s.e.m., *n* = 3) in the case of **2**. This corresponds to a remarkable 12–27-fold irradiation-dependent increase in translation. A 32-fold increase was observed when FlashCap-mRNA with **2** was irradiated before transfection. The irradiation itself only slightly decreased the translation (77 ± 7%; s.e.m., *n* = 3) in HeLa cells, as shown by controls with cap 0-mRNA. Of note, the absolute amount of cap-dependent translation triggered by light almost reaches the level of irradiated cells transfected with control mRNA (72 ± 8%, s.e.m., *n* = 3), supporting the notion that intracellular uncaging is efficient and fully functional mRNA is generated (Fig. 5a). Taken together, these data demonstrate that irradiation efficiently releases cap 0-mRNA and triggers translation in living cells transfected with FlashCap-mRNAs, without compromising cell viability and mRNA integrity (Fig. 3a and Supplementary Fig. 13).

**Extended Data Fig. 2:**
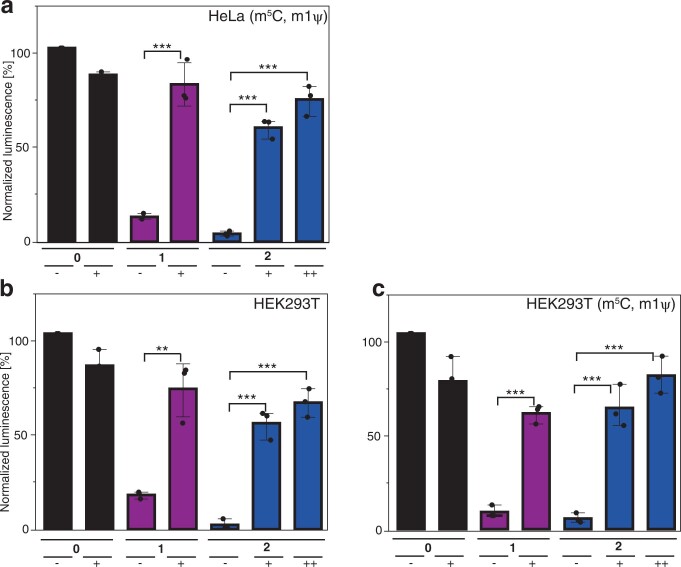
In-cell translation assay showing luciferase activity of FlashCap-GLuc-mRNAs normalized to m^7^GpppG-mRNA. The samples were either irradiated before transfection (++), in cells (+) or left untreated (−). **a**, Measured and normalized luminescence values, which were obtained from the cell media of HeLa cells that were previously transfected with differently capped GLuc-mRNA (containing m^5^C and m1Ψ) either irradiated or not irradiated in a 96 well plate. The *P* value for **1**(+) versus **1**(−) is 4.66 × 10^−4^. The *P* value for **2**(+) versus **2**(-) is 5.70 × 10^−5^. The *P* value for **2**(++) versus **2**(−) is 1.16 × 10^−5^. **b**, Measured and normalized luminescence values, which were obtained from the cell media of HEK293T cells that were previously transfected with differently capped GLuc-mRNA and either irradiated or not irradiated in a 96 well plate. The *P* value for **1**(+) versus **1**(−) is 3.57 × 10^−3^. The *P* value for **2**(+) versus **2**(−) is 1.7 × 10^−4^. The *P* value for **2**(++) versus **2**(−) is 4.3 × 10^−4^. **c**, Measured and normalized luminescence values, which were obtained from the cell media of HEK293T cells that were previously transfected with differently capped GLuc-mRNA (containing m^5^C and m1Ψ) and either irradiated or not irradiated in a 96 well plate. The *P* value for **1**(+) versus **1**(−) is 9.05 × 10^−4^. The *P* value for **2**(+) versus **2**(−) is 9.32 × 10^−4^. The *P* value for **2**(++) versus **2**(−) is 2.09 × 10^−4^. Statistical significance was determined by two-tailed t-test. Data and error bars represent average and standard error of the mean of three independent (*n* = 3) cell experiments. Significance-levels were defined as p < 0.05:*, p < 0.01:**, p < 0.001:***. Source data

In current mRNA-based therapeutics, modified nucleosides are widely used to increase translation^32^ and reduce immunogenicity^33,44^. To test whether FlashCaps are compatible with such modifications, we produced FlashCap-mRNAs containing 5-methylcytosine (m^5^C) and N1-methyl-pseudouridine (m^1^Ψ). As expected, these internal RNA modifications increased the amount of protein produced in all cases (Supplementary Fig. 14)^32^. Normalized to control-mRNAs containing the same modifications, the light-dependent turn-on effect of FlashCap-mRNAs remained in the same range both in vitro (Fig. 4e) and in cells (Supplementary Fig. 15). Light-induced translation of FlashCap-mRNAs was also achieved in HEK293T cells, demonstrating their functionality in different human cell lines (Extended Data Fig. 2 and Supplementary Fig. 16).

**Extended Data Fig. 3:**
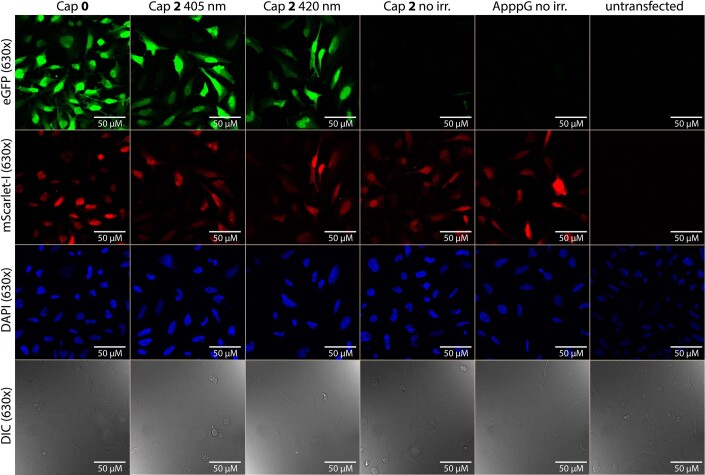
Representative 630x magnification confocal microscopy images of irradiated (405 nm, 420 nm) and non-irradiated HeLa cells transfected with eGFP- and mScarlet-I-mRNA with DAPI staining. HeLa cells were transfected with differently capped eGFP-mRNA containing m^5^C and m^1^Ψ and m^7^GpppG-capped mScarlet-I-mRNA containing m^5^C and m^1^Ψ. Untransfected cells served as control. ApppG-capped mRNA represents cap-independent translation. The m^7^GpppG-capped eGFP-mRNA (**0**) served as positive control. The NPM-(**2**) caged eGFP-mRNA was either not irradiated or irradiated in cells (405 nm, 60 s/420 nm 180 s). The top two rows show the 630x magnification (63x objective) of the red channel (mScarlet-I) or the green channel (eGFP) while the bottom two show the DAPI staining and DIC channel. Shown is one representative experiment of three independent experiments (*n* = 3). Source data

**Extended Data Fig. 4:**
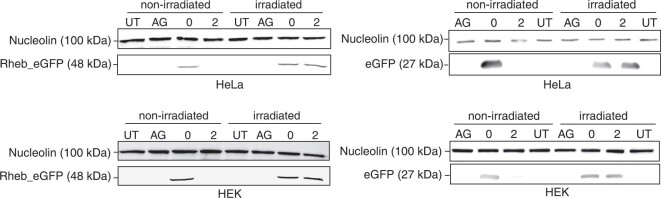
Western Blots of eGFP and Rheb_eGFP. The eGFP, Rheb_eGFP and Nucleolin protein levels of HEK293T and HeLa cell samples transfected with either ApppG-, m^7^GpppG- or NPM-capped mRNA were analyzed via Western blotting at 24 h post transfection. Irradiation of transfected cells was performed 4 h post transfection at 365 nm for 30 sec. As marker, the prestained PageRuler (ThermoFisher) was used and as primary antibodies anti-nucleolin-antibody or anti-eGFP-antibody were used, respectively. Additionally, a HRP secondary antibody was used. Shown is one representative gel from *n* = 3 independent experiments. Source data

To assess the effect of FlashCaps and light on translation for a different mRNA and using a different assay, we co-transfected HeLa cells with differently capped eGFP-mRNAs and cap 0-mScarlet-I-mRNAs as internal reference. Imaging by confocal microscopy revealed that a green fluorescent signal was barely detectable when using FlashCap-eGFP-mRNA with **2** (Fig. 5b). Control cells transfected with cap0-eGFP-mRNA showed bright fluorescence under the same conditions. However, if the cells transfected with FlashCap-mRNA were irradiated, strong green fluorescence was visible, comparable to cells transfected with cap 0-mRNA (Fig. 5b and Extended Data Fig. 3). Similarly, irradiation of FlashCap-mRNA before transfection strongly increased the fluorescence. Quantification of the microscopy images confirmed a notable increase, supporting the data obtained by the luminescence assay (Supplementary Fig. 19). Furthermore, we tested a transcript coding for Rheb, a guanosine-5′-triphosphate-binding protein that is ubiquitously expressed in humans. A western blot confirmed that FlashCap-Rheb-mRNA was muted, but efficiently translated upon irradiation (Extended Data Fig. 4), indicating that FlashCaps are compatible with biologically relevant mRNAs.

To analyse the effect of irradiation on translation also on the single-cell level, we performed flow cytometry of HeLa cells transfected with differently capped eGFP-mRNAs (Fig. 5c). Direct comparison revealed a marked increase in eGFP-positive cells when FlashCap-mRNA-containing cells had been irradiated. FlashCap-mRNAs with **1** or **2** then led to 36.0% or 41.4% eGFP-positive cells. These values are close to the 48.6% observed for the positive control (cap 0-mRNA; Fig. 5c). Without irradiation, FlashCap-mRNAs led to a substantially lower fraction of eGFP-positive cells (18–19%), albeit higher than the negative control (4%). This can be attributed to partial uncaging during this long experiment, as the same FlashCap-eGFP-mRNA shows no relevant background in confocal laser scanning microscopy (CLSM) images (Fig. 5b), nor in western blots (Extended Data Fig. 4). The histograms unambiguously show that the eGFP intensity of the irradiated samples is much higher compared to non-irradiated samples. Taken together, the flow cytometry data show, on a single-cell level, that the eGFP fluorescence intensity is increased for FlashCap-mRNAs in response to irradiation (Fig. 5c). The data independently confirm the findings from luminescence, western blot and microscopy analyses, showing that irradiation of FlashCap-mRNAs highly increases the translation of a variety of reporter mRNAs.

**Fig. 6:**
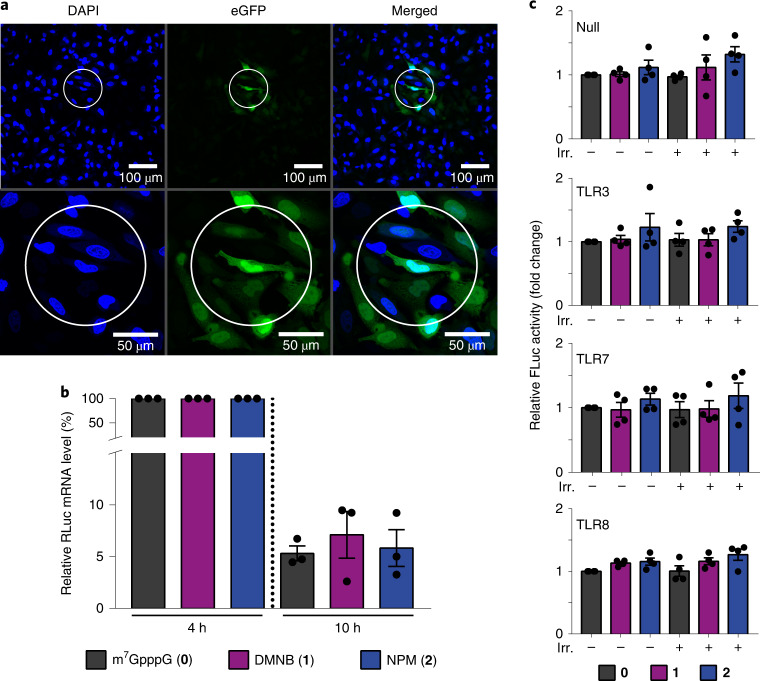
Spatio-temporal control of translation, stability and the immune response of FlashCap-mRNAs in cells. **a**, Irradiation of the circled area in a confocal laser scanning microscope and analysis of fluorescence of HeLa cells transfected with FlashCap **2**-eGFP-mRNA. Nuclei are stained by DAPI (blue). Scale bars, 100 µm or 50 µm (zoomed). Individual colour channels were adjusted. Shown is one representative image from *n* = 3 independent experiments. The microscopy images were hyperstacked and the background subtracted (30 pixels) with ImageJ. **b**, Stability of FlashCap-mRNAs. RT–qPCR data show the relative RLuc-mRNA level at 4 h or 10 h post transfection in HeLa cells. The 4-h time point is used for normalization and was set as 100%. Data of *n* = 3 independent experiments are shown as mean values ± s.e.m. **c**, Immune response of FlashCap-mRNAs. Shown is the FLuc activity of four different HEK-NF-ĸB cell lines (Null, TLR3, TLR7, TLR8) transfected with differently capped RLuc-mRNAs (either cap 0 or FlashCap **1** or **2**). TLR3, TLR7 and TLR8 indicate the overexpression of the respective TLR in that cell line. Data are normalized to the cap 0-mRNA without irradiation. Data of *n* = 4 independent experiments are shown as mean values ± s.e.m. Source data

A key feature of light-triggered processes is the exquisite and facile spatio-temporal control. Using a CLSM set-up, we tested whether brief irradiation of a predefined circle with a diameter of 120 µm using the 405-nm laser would activate translation in a subset of cells. Indeed, we observed that cells transfected with FlashCap-eGFP-mRNA containing **2** developed green fluorescence exclusively in the circled area (Fig. 6a). These data show that FlashCap-mRNAs enable control of translation in a subset of cells. Spatial control of translation on a micrometre scale can be readily achieved using a commercial CLSM set-up.

mRNA therapeutics have recently gained enormous interest. For the use of modified mRNAs in vivo in humans it is important to estimate the effects on the stability of the mRNA as well as on the elicited immune response. Previous studies reported that untranslated mRNAs are subject to degradation as part of a quality control mechanism^45^. To assess whether translationally muted FlashCap-mRNAs are prone to degradation, we determined the stability of mRNAs in cells in comparison to cap 0-mRNAs (Fig. 6b). Using quantitative real-time PCR with reverse transcription (RT–qPCR), we compared the amount of differently capped mRNAs at 4 h and 10 h after transfection. We observed similar levels of remaining mRNA 10 h after transfection, suggesting that the half-life of mRNAs is not affected by the photo-cleavable group at the 5′ cap (Fig. 6b and Supplementary Fig. 21).

To assess the effect of FlashCaps on the immune response, we used reporter HEK-NF-ĸB cell lines overexpressing a nuclear factor (NF)-ĸB-driven Firefly luciferase and different Toll-like receptors (TLRs)^46^. The control cell line (Null) has no TLR overexpressed and provides a measure for the activation of endogenously expressed pathogen recognition receptors. The FlashCap-mRNAs did not exhibit a substantial increase in response to TLR3, TLR7 or TLR8, nor to the control cell line in comparison to cap 0-mRNA (Fig. 6c). This was observed both for the unirradiated and the irradiated forms. These data suggest that the application and activation of FlashCap-mRNAs can be expected to elicit an immune response similar to cap 0-mRNAs and may thus prove suitable for application in therapeutic mRNAs.

## Conclusions

With the approval of mRNAs as a therapeutic modality, the number of studies on mRNA aiming to improve the technology and addressing other diseases can be expected to rise, both in the field of basic research as well as in preclinical and clinical studies. However, so far, no strategy exists to efficiently time the expression of the administered mRNA, nor to control the delivery and uptake into certain tissues without alterations remaining in the mRNA. Even in cell culture, the administration and liberation of exogenous mRNA currently cannot be efficiently controlled in space and time.

We developed a technique to control the translation of any given mRNA by light. FlashCaps are 5′ cap analogues containing a single photocaging group connected via a self-immolative carbamate linkage, leading to fast and efficient liberation of the natural cap 0 structure, as demonstrated by multiple assays in vitro. FlashCaps are compatible with common molecular biology techniques. They are simply added instead of the synthetic 5′ cap analogue to the in vitro transcription to make any mRNA of interest with efficiencies similar to the cap 0-mRNA. The resulting FlashCap-mRNAs are (1) translationally muted in vitro and in cells, (2) contain only a single photo-cleavable group, (3) release native cap 0-mRNA, (4) do not require changes in sequence or permanent chemical alterations and (5) are not immunogenic. We demonstrate the functionality of FlashCap-mRNAs in two different cell lines and for light-activated translation into both intracellular (eGFP, RLuc, Rheb) and secreted (GLuc) proteins. The irradiation conditions required to release cap 0-mRNA are compatible with cell viability and translation, and the photo-cleavable groups have even proven compatible with animal models in previous studies^47,48^. An up to 32-fold light-induced increase of translation was observed in HeLa cells. We also confirmed that translationally muted FlashCap-mRNAs are not preferentially degraded and do not elicit an increased immune response compared to cap 0-mRNAs. FlashCaps are therefore a highly efficient and readily applicable solution to make mRNA studies controllable by light, without requiring new production steps and without introducing permanent artefacts.

## Methods

### Absorbance spectra analysis

The analysis of the absorbance properties of the photocaged guanosines was performed using a quartz cuvette (Hellma) together with an FP-8500 fluorescence spectrometer (Jasco). The respective guanosines were dissolved in water at a final concentration of 100 µM. For the absorbance measurements, 20 µl of the solution was further diluted in water to give a final volume of 100 µl (20 µM), which was transferred into the cuvette followed by the absorbance measurement. Values are normalized to the highest measured value of each measurement.

### Irradiation of samples

LEDs (LED Engin) were used to irradiate mRNA samples, guanosines, cells and cap analogues. The UV-A LED (*λ*_max_ = 365 nm) and the blue-light LEDs (*λ*_max_ = 405 nm, *λ*_max_ = 420 nm) were operated at 5 V and 600 mA input power (the respective output power is shown in Supplementary Fig. 1).

Irradiation was performed in a custom-made LED set-up at 23 °C. The samples were irradiated in a PCR tube or a cell culture dish (Supplementary Fig. 2) unless stated otherwise. Samples were irradiated at 365 nm (142 mW cm^−2^) for 30 s, 405 nm (142 mW cm^−2^) for 60 s or 420 nm (52 mW cm^−2^) for 120 s, unless otherwise noted.

### Guanosine and dinucleotide irradiation studies

The respective guanosines or cap analogues were dissolved in ddH_2_O if possible (if needed, organic solvents were added to increase solubility) to give a solution with a final concentration of 500 µM. The solution (10 µl) was transferred into a PCR tube and irradiated as described above. Subsequently, the solution was analysed by HPLC.

### HPLC analysis

HPLC analysis and purification of cap analogues were performed on an Agilent1260 Infinity HPLC system equipped with a diode array detector (DAD) (190–640 nm) using a Nucleodur C18 Pyramid reversed-phase column (5 μm, 125 × 4 mm) from Macherey–Nagel. Elution was carried out at a flow rate of 1 ml min^−1^ by applying a linear gradient from buffer A (50 mM ammonium acetate, pH 6.0) to buffer B (1:1 buffer A:acetonitrile). If other conditions were used, this is described in the respective section.

### MST measurements

MST measurements were performed on a Monolith NT.115 series instrument (NanoTemper). Before the thermophoresis measurements, proteins were labelled by incubation with Cy5-NHS (Lumiprobe) for 30 min at room temperature (r.t.). Unreacted dye was separated from the protein using PD SpinTrap G-25 gel filtration columns (GE Healthcare) according to the manufacturer’s protocol. Serial dilutions of the cap analogues (starting from 200 µM of cap analogue) in MST reaction buffer (20 mM HEPES, 50 mM KCl, 0.2 mM EDTA, 0.01% Triton-X, 700 µM mercaptoethanol, 0.01% Tween-20, pH 8) were prepared and mixed with an equal volume of the labelled protein (~50 nM). The mixture was filled into premium coated capillaries (4 μl) and directly measured. The MST power was set to 30–40%, the LED power to 20% red (excitation, 625 nm; emission, 680 nm). Thermophoresis measurements were performed with the following settings: fluorescence before (5 s), MST on (30 s), fluorescence after (3 s). The capillaries were measured three times in direct succession as technical replicates. MST data were normalized to baseline differences and *K*_d_ values were calculated using nonlinear regression assuming a Hill coefficient of 1.0 (GraphPad Prism). MST is known to produce occasional outliers. This was handled as follows: 16 data points were measured per binding curve and at least 12 data points were used for each fit.

### yDcpS hydrolysis assay

The hydrolytic activity of yDcpS (New England Biolabs) was assayed using the following experimental conditions: 50 mM Tris-HCl containing 1 mM Mg(Ac)_2_, 30 mM (NH_4_)_2_SO_4_ and 1 mM dithiothreitol (final pH 8.0) at 37 °C. Together with the respective cap analogue, an internal standard (either adenine monophosphate or 4,5,7-trihydroxy-3-phenyl­coumarin, with a final concentration of 200 µM) was added. Finally, 20 U of yDcpS were added. The hydrolysis process was started by incubation at 37 °C. At 0, 5, 10, 15, 30, 45 and 60 min of the hydrolysis, 10-µl aliquots of the reaction mixture were withdrawn and the reaction was stopped by heat inactivation of the enzyme (10 min at 90 °C). The samples were then subjected to analytical HPLC and analysed at 260 nm. Hydrolysis products were identified by comparison of their retention times with those of reference standards.

### Expression and purification of MTAN, LuxS, hTgs, hDcpS and eIF4E

The enzymes 5'-methylthioadenosine nucleosidase (MTAN), LuxS, hDcpS H277N, eIF4E and hTgs were produced and purified as previously described^16,35,49,50^.

### In vitro transcription

The DNA template required for the in vitro transcription was synthesized by PCR, in which the DNA sequence coding for eGFP, Firefly luciferase (FLuc), Gaussia luciferase (GLuc) and Renilla luciferase (RLuc) were amplified from pMRNA vectors containing the respective sequence. After purification (NucleoSpin Gel and PCR Clean-up, Macherey–Nagel), the resulting linear dsDNA was used as template (200 ng). The runoff template is an alternative to the PCR-DNA template that was used for the GLuc, mScarlet-I and eGFP-mRNAs used in cell studies and fluorescence microscopy. Plasmid DNA (3 µg) was incubated with 1× FastDigest buffer (Thermo Fisher) and 3 µl of PacI FastDigest enzyme for 10 min at 37 °C, followed by inactivation at 65 °C for 10 min. Subsequently, the ends were dephosphorylated by adding 3 µl of FastAP and incubation at 37 °C for 15 min and inactivation at 65 °C for 5 min. The product was purified using the NucleoSpin Gel and PCR Clean-up kit (Macherey–Nagel). The concentration was measured at 260 nm with a Tecan Infinite M1000 PRO instrument. The resulting linear dsDNA was used as template (400 ng). The in vitro transcription was performed with T7 polymerase (Thermo Scientific) in transcription buffer (40 mM Tris/HCl, 25 mM NaCl, 8 mM MgCl_2_, 2 mM spermidine(HCl)_3_) by adding either an A/C/UTP (0.5 mM) mix or A/m^5^C/m^1^ΨTP mix (0.5 mM), guanosine-5'-triphosphate (0.25 mM), the respective cap analogue (1 mM), T7 RNA polymerase (50 U; Thermo Scientific) and pyrophosphatase (0.1 U; Thermo Scientific) for 4 h at 37 °C. After the reaction, the DNA template was digested in the presence of 2 U of DNase I for 1 h at 37 °C and then mRNAs were purified using the RNA Clean & Concentrator-5 kit (Zymo Research). To digest non-capped RNAs, 10 U of the RNA 5′-polyphosphatase (Epicentre) as well as the supplied reaction buffer were added to purified mRNAs. After an incubation period of 30 min at 37 °C, 0.5 U of the 5′–3′ exoribonuclease XRN1 (NEB) and MgCl_2_ (5 mM) were added. The reaction mixture was incubated for 60 min at 37 °C. Subsequently, capped mRNAs were purified using the RNA Clean & Concentrator-5 kit (Zymo Research).

### In vitro luminescence assay

For in vitro translation, the Retic Lysate IVT kit (Invitrogen), a eukaryotic cell-free protein expression system, was used. In a total volume of 15 µl, 40 ng of the FLuc–mRNA (capped as indicated), 50 µM l-methionine and 150 mM potassium acetate were mixed with 8.5 µl of the reticulocyte lysate and incubated for 90 min at 30 °C. Samples were mixed with 8.5 µl of the reticulocyte lysate and incubated for 90 min at 30 °C. Afterwards, 2 µl of the respective translation mix was further used in a luminescence assay. The translation efficiencies of the differently capped FLuc–mRNAs were measured using a luciferase assay based on the Beetle-juice Luciferase Assay Firefly (pjk). Luciferase activity was determined after adding 50 µl of freshly prepared substrate solution to the translation mixture. Luminescence was assessed using a Tecan Infinite M1000 PRO microplate reader with an integration time of 3 s. Differently capped mRNAs were used. ApppG-capped mRNA represents cap-independent translation and was subtracted as background from the other samples. All values were normalized to m^7^GpppG-capped mRNA.

### Mammalian cell culture

HeLa cells (Merck) were cultured in MEM Earle’s medium (PAN) supplemented with l-glutamine (2 mM, PAN), non-essential amino acids (1%, PAN), penicillin and streptomycin (1%, PAN) and fetal calf serum (FCS; 10%, PAN) under standard conditions (5% CO_2_, 37 °C). HEK293T cells (DSMZ) were cultured in Dulbecco’s modified Eagle’s medium (DMEM; PAN) supplemented with l-glutamine (2 mM, PAN), penicillin and streptomycin (1%, PAN) and FCS (10%, PAN) under standard conditions (5% CO_2_, 37 °C).

HEK-NF-κB cells (TRON) were cultured under standard conditions (5% CO_2_, 37 °C) in DMEM supplemented with FCS (10%), HEPES buffer (1%), l-glutamine (1%), non-essential amino acids (1%) and sodium pyruvate (1%). For selection, the following antibiotics were added to the culture of the HEK-NF-κB-Null, HEK-NF-κB-TLR7 and HEK-NF-κB-TLR8 cell lines: blasticidin (10 µg ml^−1^), Zeocin (100 µg ml^−1^) and Geneticin (G418; 250 µg ml^−1^). The HEK-NF-ĸB-TLR3 cell line was cultured in the absence of Geneticin. All of the cell lines overexpress an NF-κB driven Firefly luciferase, which allows the detection of NF-κB production in a luminescence assay and can be used as an indicator for the induction of an immune response^46^. Additionally, the cell lines HEK-NF-κB-TLR3, HEK-NF-κB-TLR7 and HEK-NF-κB-TLR8 overexpress the respective TLRs.

### Stability assay of 5′ caps in cell lysate

For preparation of HeLa cell lysate, HeLa cells were cultured as mentioned above. At 24 h before cell lysis, 3 × 10^6^ cells were seeded on a Petri dish (90 mm). The cells were collected and pelleted by centrifugation. The cell pellets were stored at −80 °C. For cell lysis, the medium was removed and the cells were washed with 1× phosphate buffered saline (PBS), then lysed with CelLytic M reagent (1.5 ml, Sigma Aldrich) according to the manufacturer’s instructions and stored at −80 °C. The lysis mixture was centrifuged (11,000 r.p.m., 3 min, 4 °C) and the supernatant was used for the cell lysate stability assay. To the cell lysate were added the respective cap analogue (500 µM) and 4,5,7-trihydroxy-3-phenyl­coumarin (100 µM) as internal standard, followed by incubation for different periods of time (0, 0.5, 1, 5, 18 and 30 h) at 37 °C. The samples were analysed by HPLC.

### MTT assay

HeLa cells (Merck) were cultured as mentioned above. One day before transfection, the cells were seeded in a 96-well plate (30,000 cells per well) and cultured in minimal essential medium (MEM) with antibiotics. The cells were transfected with mRNA (100 ng) in Opti-MEM (10 µl) using Lipofectamine MessengerMAX transfection reagent (0.3 µl) in Opti-MEM (9.7 µl). The cells were incubated with the mRNA/Lipofectamine MessengerMAX mixture for 6 h at 37 °C in a total volume of 100 µl. The samples were irradiated under the indicated conditions. Subsequently, the cell medium with the transfection agent was replaced by fresh medium and the cells were incubated overnight at 37 °C in medium. At 24 h post transfection, MTT solution (16.5 mg MTT in 3.3 ml of PBS) was added to the 96-well plate (12.5 µl per well). After 4 h of incubation at 37 °C, the supernatant was removed and 0.04 M HCl in isopropanol was added to the wells. After incubation for 1.5 h at r.t., 100 µl of the supernatant was placed in a new 96-well plate and absorption at 550 nm was measured using the Tecan Infinite M1000 PRO plate reader.

### In-cell luminescence assay

HeLa or HEK293T cells were cultured as mentioned above. One day before transfection, the cells were seeded in a 96-well plate (30,000 cells per well) and cultured in MEM with antibiotics. The cell were transfected with mRNA (100 ng) in Opti-MEM (10 µl) using Lipofectamine MessengerMAX transfection reagent (0.3 µl) in Opti-MEM (9.6 µl). The cells were incubated with the mRNA/Lipofectamine MessengerMAX mixture for 6 h at 37 °C in a total volume of 100 µl. The samples were irradiated at 365 nm for 30 s if not stated otherwise. Subsequently, the cell medium with the transfection agent was replaced with fresh medium and the cells were incubated overnight at 37 °C in medium. At 24 h post transfection, the supernatant was collected. To perform the luminescence measurement, a Gaussia-Juice Luciferase Assay kit (PJK) was used. The supernatant of the previously prepared samples was transferred to a 96-well plate (5 µl of supernatant per well). Afterwards, 50 µl of a reaction mixture (PJK Reconstruction buffer and Coelenterazine) was added to the wells and the luminescence activity was measured using a Tecan Infinite M1000 PRO plate reader. The activity in relative light units (RLU) was determined with an integration time of 3 s. Differently capped mRNAs were used. ApppG-capped mRNA represents cap-independent translation and was subtracted as background from the other samples. All values were normalized to m^7^GpppG-capped mRNA.

### CLSM

For microscopic imaging, HeLa cells were cultured as mentioned above. One day before transfection, 2 × 10^5^ cells were seeded on glass coverslips in a 12-well plate in 1 ml of medium (indicated in cell culture section). Cells were transfected using 1.5 µl of Lipofectamine MessengerMAX (Invitrogen) in Opti-MEM (48.5 μl) and eGFP-mRNA (containing m^5^C and m^1^Ψ; 1 μg) in Opti-MEM (50 μl). In the case of mScarlet-I/eGFP co-transfection, a total amount of 1 µg mRNA (eGFP 800 ng, mScarlet-I 200 ng) was used. For confocal microscopy the runoff plasmid of pRNA2-(A)128 (Addgene) with eGFP or mScarlet-I was used as template for in vitro transcription. At 24 h post transfection, cells were fixed with 300 μl per well of 4% paraformaldehyde in PBS for 10 min at r.t. After washing, the nuclei were stained with 4′,6-diamidino-2-phenylindole (DAPI; 1:10 in PBS). After washing with PBS and water, the coverslips were mounted on microscopy slides using Aqua-Poly/mount (Polysciences). A Leica TCS SP8 CLSM was used to image fixed cells with a ×63 water immersion objective lens (HC PL APO ×63/1.20 W CORR UVIS CS2). Images were captured at a green channel for eGFP fluorescence (*λ*_ex_ = 488 nm, *λ*_em_ = 492–558 nm), a red channel for mScarlet-I fluorescence (*λ*_ex_ = 568 nm, *λ*_em_ = 583–693 nm), a blue channel for DAPI (*λ*_ex_ = 358 nm, *λ*_em_ = 443–510 nm) and at the differential interference correlation channel. The objectives used in this study were HC PL APO ×63/1.20 W CORR UVIS CS2 and HC PL FLUOSTAR ×10/0.30 Ph1 objectives, the laser was a diode laser (405 nm; 8.3 mW, laser power in the focus plane with a ×10 objective), and the detectors were photomultiplier (Hamamatsu R 9624) HyD detectors. For all microscopy images, hyperstacking and background subtraction were performed with ImageJ (30 pixels).

### RNA isolation and RT–qPCR

For RT–qPCR, HeLa cells (Merck), were cultured as described above. One day before transfection, 2 × 10^5^ cells were seeded in medium (1 ml) in a 12-well plate. Cells were transfected using 1.5 µl of Lipofectamine MessengerMAX (Invitrogen) in Opti-MEM (48.5 μl). 1 µg of RLuc-mRNA in Opti-MEM (50 μl) was prepared. The cells were incubated with the mRNA/Lipofectamine MessengerMAX mixture for 4 h at 37 °C in a total volume of 1 ml. Subsequently, the cell medium with the transfection agent was replaced with fresh medium. The cells were collected at 4 h or 10 h post transfection by adding 500 µl of lysis buffer (10 mM Tris-HCl (pH 8), 150 mM NaCl, 0.5 mM EDTA, 0.1% NP40). The RNA was isolated from the cell lysate via phenol/chloroform extraction. The isolated total RNA was incubated with DNase I (2 U) in DNase reaction buffer (1×) in a total volume of 20 µl for 30 min at 37 °C to digest the remaining DNA. Addition of EDTA (final concentration 5 mM) and incubation for 2 min at 65 °C was used to inactivate the enzymes. For reverse transcription, 1× RT buffer, dNTPs (final concentration 0.5 mM) with random hexamer primer (5 μM) and Maxima H Minus reverse transcriptase (25 U) were mixed for 10 min at 25 °C followed by 30 min at 50 °C and finally 5 min at 85 °C. The resulting complementary DNA (cDNA) was diluted 1:3 in ddH_2_O and 3 µl of the diluted cDNA was added into a 96-well qPCR plate. 17 µl of Mastermix, containing forward primer (0.5 μM), reverse primer (0.5 μM) and 1× iTaq Universal SYBR Green Supermix (Bio-Rad), was added to the provided cDNA in the 96-well plate (Supplementary Table 1). The following PCR program was applied: (1) initial denaturation (95 °C for 3 min), (2) denaturation (95 °C for 5 s), (3) elongation (55 °C for 30 s), (4) plate read, (5) 39 × cycle (2)–(4), (6) melt curve (60 °C–95 °C, 0.5 °C per 4 s) and (7) plate read. Quantitative real-time PCR measurements were performed on a Bio-Rad CFX96TM Real-Time System with a C1000TM Touch Thermal Cycler. Data analysis was performed with the CFX Manager 3.1 (Bio-Rad).

### Flow cytometry

For flow cytometry, HeLa cells were cultured as mentioned above. One day before transfection, 2 × 10^5^ cells were seeded in a 12-well plate in 1 ml of medium. Cells were transfected using 1.5 µl of Lipofectamine MessengerMAX (Invitrogen) in Opti-MEM (48.5 μl) and 1 µg eGFP-mRNA in Opti-MEM (50 µl). The cells were incubated with the mRNA/Lipofectamine MessengerMAX mixture for 4 h at 37 °C in a total volume of 1 ml. The samples were irradiated at 365 nm for 30 s. Subsequently, the cell medium with the transfection agent was replaced with fresh medium and the cells were incubated overnight at 37 °C. At 24 h post transfection, the cells were collected with trypsin/EDTA and washed with PBS. The cell suspension was filtered through a 40-µm filter to avoid cell clumps. The eGFP signal was measured with a flow cytometer (Beckman Coulter Cytomics FC 500). During flow cytometry, 10,000 cells (total cell count) were measured per sample. Analysis was performed with the CxP Analysis Software.

### Detection of immunogenicity in HEK-NF-ĸB cells

HEK-NF-ĸB cells were cultured as mentioned above. One day before transfection, 1.5 × 10^5^ cells were seeded in medium (500 µl) in a 24-well plate. Cells were transfected using Metafectene Pro (2 μl; Biontex) in PBS (28 μl) and (non-irradiated or irradiated) RLuc-mRNA (500 ng) in PBS (30 μl). At 20 h post transfection, the cells were collected and washed with PBS. The pellets were resuspended in 50 µl of PBS and used for the luminescence assay. The luminescence measurement was performed using the Beetle-Juice Luciferase assay Firefly kit (pjk). The reagents were prepared as suggested by the manufacturer. The 50 µl of cell suspension were mixed with 50 µl of 2× Lysis Juice. After incubation for 15 min at 37 °C (450 U min^−1^), 20 µl of the cell lysate was transferred to a 96-well plate (in duplicates), then 50 µl of the freshly prepared Firefly reaction mixture was injected into the well with an acquisition time of 3,000 ms. The samples were normalized to the m^7^GpppG-capped mRNA.

### Western blots

HEK293T and HeLa cells were cultured as mentioned above. One day before transfection, 2 × 10^5^ cells were seeded in a 12-well plate in 1 ml of medium. Cells were transfected using 1.5 µl of Lipofectamine MessengerMAX (Invitrogen) in Opti-MEM (48.5 μl) and 1 µg of eGFP-mRNA in Opti-MEM (50 µl). At 4 h post transfection, the cells were irradiated (142 mW cm^−2^, 365 nm, 30 s) and the transfection medium was replaced with fresh medium. At 24 h post transfection, the cells were collected and washed with PBS. The cells were lysed with CelLytic M (Sigma Aldrich). To determine the protein concentration of the cell lysate, a Bradford assay was performed using BSA calibration standards and a dilution of cell lysate (1:25), then 50 µl of the sample was incubated (10 min, r.t., exclusion of light) with 1× Roti-Quant (Roth) staining solution (200 μl) and the extinction at 595 nm was determined. The proteins (40 µg) were separated via tris-glycine–PAGE (12% polyacrylamide (PAA) gel, 120 V, 1.5 h, r.t.). The proteins were transferred onto a nitrocellulose membrane Roti-NC (Roth) in a semi-dry transfer buffer with 90 mA for 75 min at r.t. To validate protein transfer, a Ponceau S (0.5% Ponceau S + 1% glacial acetic acid) stain was performed. The membrane was cut into two appropriate pieces for subsequent antibody treatment and washed with 1× PBS + 0.01% Tween (PBST). Blocking of the membrane was performed in blocking buffer (3% BSA in PBS) for 1 h at r.t., followed by incubation with the respective primary antibodies—anti-eGFP mouse monoclonal antibody (Santa Cruz Biotechnology) and anti-nucleolin mouse monoclonal antibody (Thermo Fisher Scientific)—overnight at 4 °C and three times washing with PBST for 5 min at r.t. The membrane pieces were incubated with a horseradish peroxidase (HRP)-conjugated secondary antibody (polyclonal rabbit anti-mouse immunoglobulins/HRP; Dako Diagnostica) for 1 h at r.t. and then washed three times with PBST. For chemiluminescence detection, the EZ-ECL chemiluminescence detection kit (Biological Industries) was used and the results were analysed with a Chemo Star Advanced Fluorescence & ECL imager (Intas).

### Decapping assay

The RNA was prepared as mentioned above. A 1-µg sample of capped eGFP-mRNA was mixed with mRNA decapping enzyme reaction buffer (NEB; final concentration 1×) in a total volume of 19.7 µl. The mixture was irradiated (except for the control without irradiation). Then either 0.3 µl of mRNA decapping enzyme (NEB) or 0.3 µl of H_2_O as negative control was added. After incubation for 30 min at 37 °C, 1 µl of XRN1 and 2.5 µl of MgCl_2_ were added to each Eppendorf tube (to the controls as well) and incubated for 1 h at 37 °C, then 2 µl of each sample were loaded on a 7.5% PAA gel. RiboRuler Low Range (Thermo Fisher) was used as a marker.

### Remethylation assay

A solution of LuxS (5 µM), MTAN (5 µM), the corresponding cap analogue (400 µM), SAM (6 mM) and hTgs (20 µM) in buffer (5 mM Tris-HCl, 10 mM MgCl_2_, 5 mM KCl, pH 8.0) was incubated at 37 °C. At 0, 5, 15, 30 and 60 min of the methylation reaction, 10-µl aliquots of the reaction mixture were withdrawn and the reaction was stopped by heat inactivation of the enzyme (10 min at 90 °C). The samples were then analysed by HPLC, monitoring the absorbance at 260 nm. Methylation products were assigned by comparison of their retention times with those of reference standards.

### Statistical analysis

For statistical analysis of the luminescence data, an unpaired, parametric, two-tailed Student’s *t*-test was used. When compared with the m^7^GpppG mRNA, an additional Welch correction was used (*P < 0.05, **P < 0.01, ***P < 0.001).

### Reporting summary

Further information on research design is available in the Nature Research Reporting Summary linked to this Article.

## Online content

Any methods, additional references, Nature Research reporting summaries, source data, extended data, supplementary information, acknowledgements, peer review information; details of author contributions and competing interests; and statements of data and code availability are available at 10.1038/s41557-022-00972-7.

## Supplementary information

Supplementary Information Supplementary Figs. 1–80, synthetic procedures, references for synthetic procedures and Tables 1–4.

Reporting Summary

## Data Availability

The data generated or analysed during this study are included in this Article and its Supplementary Information. Protein structures and models used for the figures are available under PDB accession code 1EJ1. Source data are provided with this paper.

## Source data

Source Data Fig. 3 Statistical source data
Source Data Fig. 4 Statistical source data and unprocessed gels
Source Data Fig. 5 Statistical source data and microscopy images
Source Data Fig. 6 Statistical source data and microscopy images
Source Data Extended Data Fig. 1 Statistical source Ddta
Source Data Extended Data Fig. 2 Statistical source data
Source Data Extended Data Fig. 3 Microscopy images
Source Data Extended Data Fig. 4 Unprocessed and uncropped western blots

## Code availability

No custom software was used in this study. The following software was used for data collection: Leica Application Suite X (3.5.6.21594), CxP Analysis Software, CFX Manager Software V 3.1 (Bio-Rad), Qualitative Navigator B.08.00, Agilent ChemStation for LC 3D systems Rev. B.04.03 and Agilent OpenLab CDS ChemStation Edition Rev. C.01.10. The following software was used for data analysis: Origin (2016b), Origin (2021b), Origin (2019b), GraphPad Prism 7, ImageJ (Version 20160205), MestReNova 14, LasX (Version), Excel 2016 and PerkinElmer ChemDraw 20.1.
