# Supplementary material for: Photocaged 5′ cap analogues for optical control of mRNA translation in cells
Source: Nat Chem. 2022 Jun 20;14(8):905–13. doi: 10.1038/s41557-022-00972-7 (PMC7613264; doi:10.1038/s41557-022-00972-7)
Supplement: Supplementary file 1 — Supplementary Figs. 1–80, synthetic procedures, references for synthetic procedures and Tables 1–4. [file 41557_2022_972_MOESM1_ESM.pdf]

nature portfolio

https://doi.org/10.1038/s41557-022-00972-7

Supplementary information

Photocaged 5′ cap analogues for optical control of mRNA translation in cells

In the format provided by the authors and unedited

Table of contents

Material Sources 1
Supplementary Figures 4
Synthetic procedures 24
References 75

Material Sources

All chemicals were purchased from Alfa Aesar, Applichem, Fluorochem, Sigma Aldrich or TCI unless otherwise noted. The HPLC grade acetonitrile was purchased from VWR. All components were used without further purification.

G(5′)ppp(5′)A (GpppA) cap analogue, ARCA ($m_2^{7,3'-O}$G(5')ppp(5')G) cap analogue and m^7G(5′)ppp(5′)G (GpppG) cap analogue were purchased from New England Biolabs or Jena Bioscience.

Oligonucleotides were purchased from Biolegio.

Phusion High–Fidelity DNA Polymerase, FastAP Thermosensitive Alkaline Phosphatase, RiboLock RNA Inhibitor, T7 RNA polymerase, pyrophosphatase, DNase I were purchased from Thermo Scientific, nuclease P1 from Sigma Aldrich.

Unless stated otherwise, solvents were evaporated at 40 °C.

^{1}H, ^{13}C and ^{31}P NMR spectra were measured on Agilent DD2 600 spectrometer (600 MHz, 151 MHz and 243 MHz) and Agilent DD2 500 spectrometer (500 MHz, 126 MHz and 202 MHz). The measurements were performed in DMSO–*d6* or D_2O and referred to residual solvent signal. Complete assignment (if present) is based on heteronuclear correlation experiments HSQC and H, C–HMBC. Chemical shifts (δ) are in ppm and coupling constants (J) in Hz. The numbering system for the assignment of NMR signals is given for the majority of the compounds individually.

High resolution mass spectra were measured: Orbitrap LTQ XL (Thermo Fisher Scientific) spectrometer using ESI technique and Orbitrap Velos Pro (Thermo Fisher Scientific) spectrometer using ESI technique.

For determination of other mass spectra, HPLC – triple–quadrupole mass spectrometry system was used. The system consists of Agilent 1260 Infinity II with dual λ absorbance detector (G7114A) HPLC part and Agilent Ultivo

mass spectrometer with JetStream ion source (type of ESI). Column: Agilent Poroshell 120 EC–C18 (3.0x150/2.7μm). The polar compounds were separated using the following LC method: eluent 20 mM NH_4OAc buffer (pH = 6)/CH_3CN, gradient 0–30%, column temperature = 20 °C. For other compounds the LC method: eluent 20 mM NH_4OAc buffer (pH = 6)/CH_3CN, gradient 0–100%, column temperature = 40 °C.

HPLC analysis was performed on an Agilent 1260 Infinity HPLC equipped with a diode array detector (DAD) (190–640 nm) using a Nucleodur® C18 Pyramid reversed–phase column (5 μm, 125 x 4 mm) from Macherey–Nagel. Elution was performed at a flow rate of 1 mL/min applying a linear gradient for buffer A (100 mM K–Phosphate, pH = 6.5) and buffer B (1:1 buffer A : acetonitrile).

For flash chromatography, Interchim puriFlash® XS520Plus with multi λ absorbance detector and various types of columns were used: a) 330g column, 50μm spherical silica gel, Interchim [PF–50SIHP–F0330]; b) 120g column, 50μm spherical silica gel, Interchim [PF–50SIHP–F0120]; c) 40g column, 25μm spherical silica gel, Interchim [PF–25SIHC–F0040].

For reversed–phase (RP) flash chromatography and preparative HPLC, Büchi PrepChrom C–700 multi λ absorbance detector and various types of columns were used: a) Teledyne ISCO columns RediSepRf® variation „HP C18 Aq GOLD‟ in sizes 50g, 100g and 150g (RP flash chromatography); b) Macherey–Nagel VP 250/21 NUCLEODUR C18 Pyramid, 10 μm [REF 762285.210] (preparative HPLC).

K–Phosphate Buffer (phosphate (K_2HPO_4/KH_2PO_4) buffer for the analytical HPLC)

K_2HPO_4 (17.4 g) and KH_2PO_4 (27.2 g) were dissolved in ddH_2O (3 L). The pH was tested (pH = 6.5) by calibrated digital pH–meter and filtrated over sterile 0.22 μm filter under vacuum.

2–Nitrobenzyl alcohol (**4c**)[CAS: 612–25–9]

Commercially available. Supplier: Sigma–Aldrich [code: N12805–25G]

4,5–Dimethoxy–2–nitrobenzyl alcohol (**4a**) [CAS: 1016–58–6]

Commercially available. Supplier: FluoroChem [code: 018587]

6–Nitropiperonal (**9**) [CAS: 712–97–0]

Commercially available. Supplier: ChemPur [code: 005878–100]

Guanosine (**3**) [CAS: 118–00–3]

Commercially available. Supplier: FluoroChem [code: 212784]

Supplementary Figures

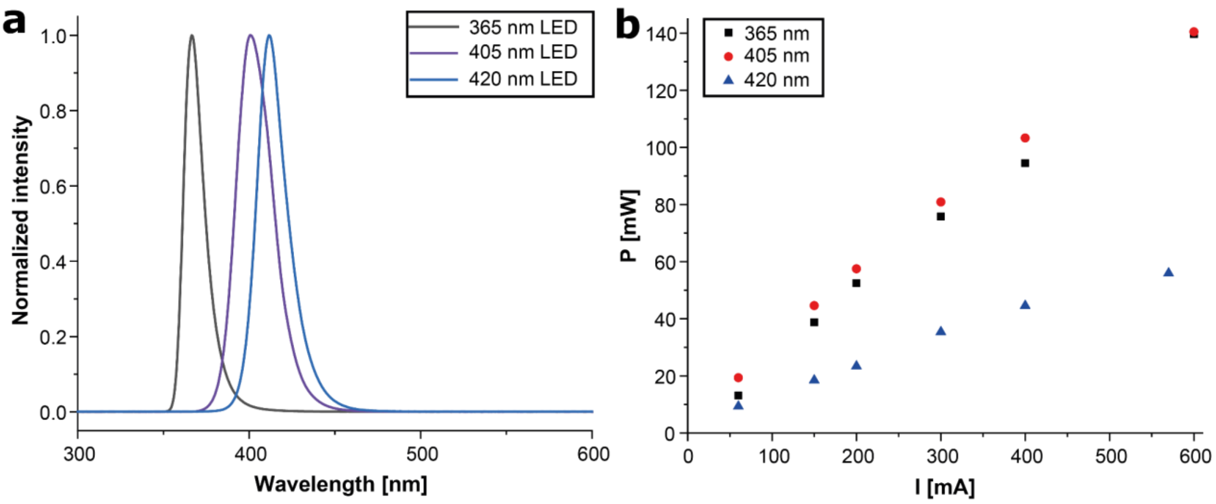

Supplementary Figure 1*.* Performance of LEDs used in this study. a, LED emission spectra, measured using a Jasco FP–8300 in previous studies.[1] **b**, LED output power (mW) as a function of applied current (mA at 5 V) for the respective LEDs measured on a 1 cm^2 sensor (Ophir Photonics).

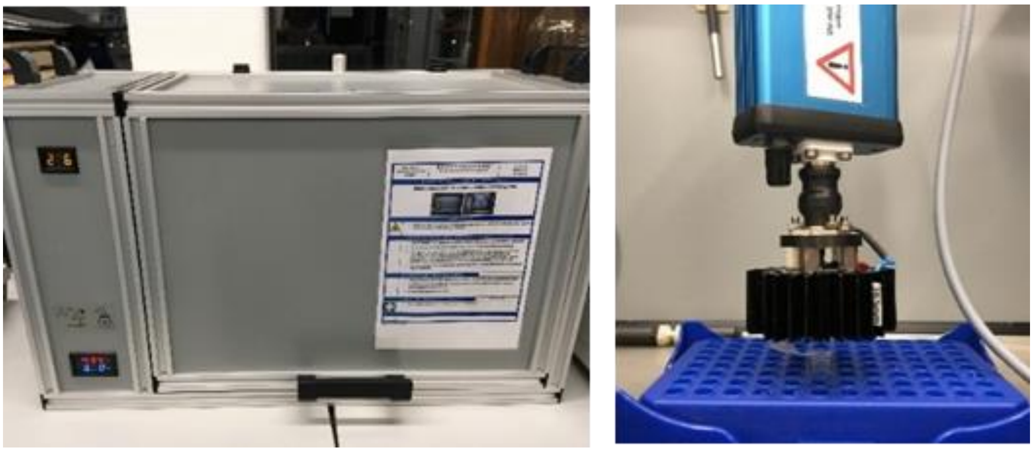

Supplementary Figure 2. LED setup used in this study. Outside of the box (left) and distance between the LED and the sample (right).

Supplementary Table 1. Primer sequences for RT-qPCR.

Primer Name	Sequence 5′-3′
**GAPDH forward**	CAAATTCCATGGCACCGTCA
**GAPDH revers**	TCGCCCCACTTGATTTTGG
**RLuc forward**	GCCAGTAGCGCGGTGTATTA
**RLuc revers**	GCCAAACAAGCACCCCAATC

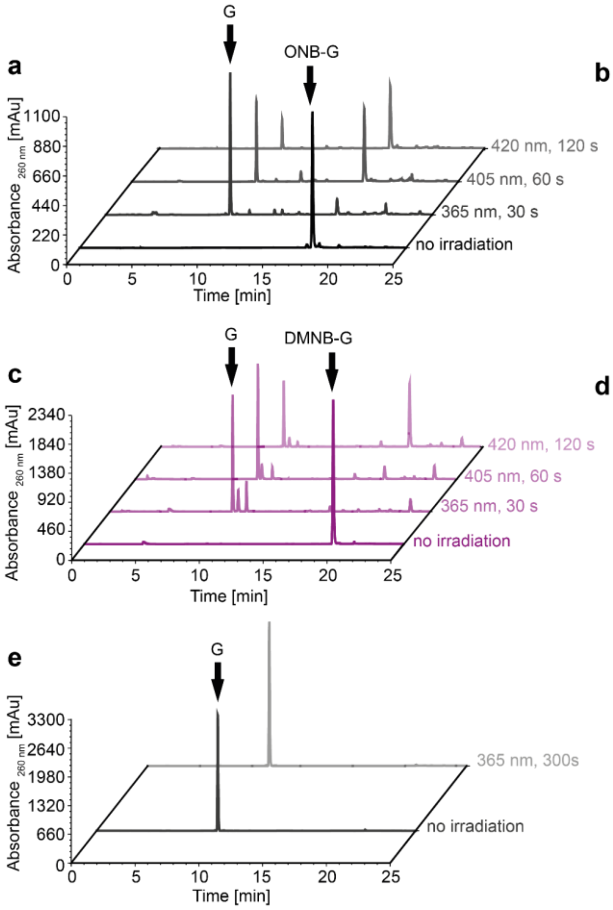

Supplementary Figure 3. Representative HPLC traces after irradiation of photocaged guanosines under different conditions. Chromatograms for non-irradiated and the longest irradiated guanosine (G) are shown for different wavelengths. The ONB-G (t = 16.5 min) (**a**), NP-G (t = 17.5 min) (**b**), DMNB-G (t = 18.3 min) (**c**), NPM-G (t = 19.2 min) (**d**) and non caged G (t = 7.2 min) (**e**) were analysed after irradiation at indicated conditions. Non-caged guanosine (**e**) was irradiated to validated that irradiation has no effect on guanosine itself.

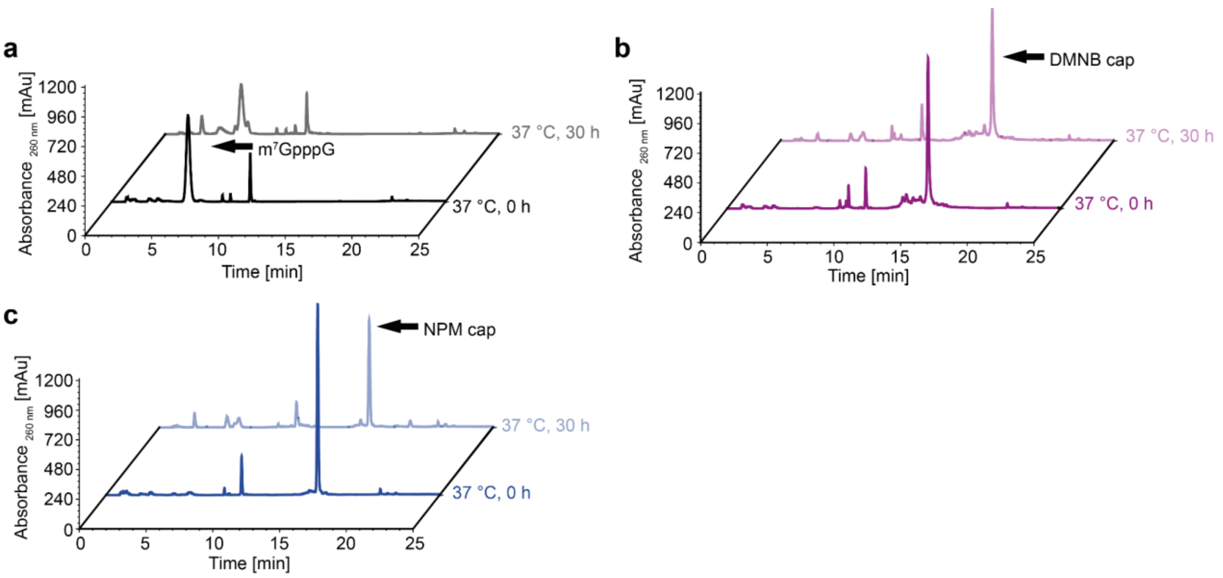

Supplementary Figure 4. Representative HPLC traces of the cell lysate stability assay. The chromatograms show the HPLC analysis of the caged cap analogues in HeLa cell lysate before and after incubation at 37 °C for 30 hours. The peak at 11 minutes represents the internal standard that was used to quantify the cap analog degradation. **a**, representation of the analysis of uncaged m^{7}GpppG (t = 6 min). **b**, illustration of the HPLC analysis of the DMNB cap (t = 16 min). **c**, HPLC analysis of the NPM cap (t = 17.2 min).

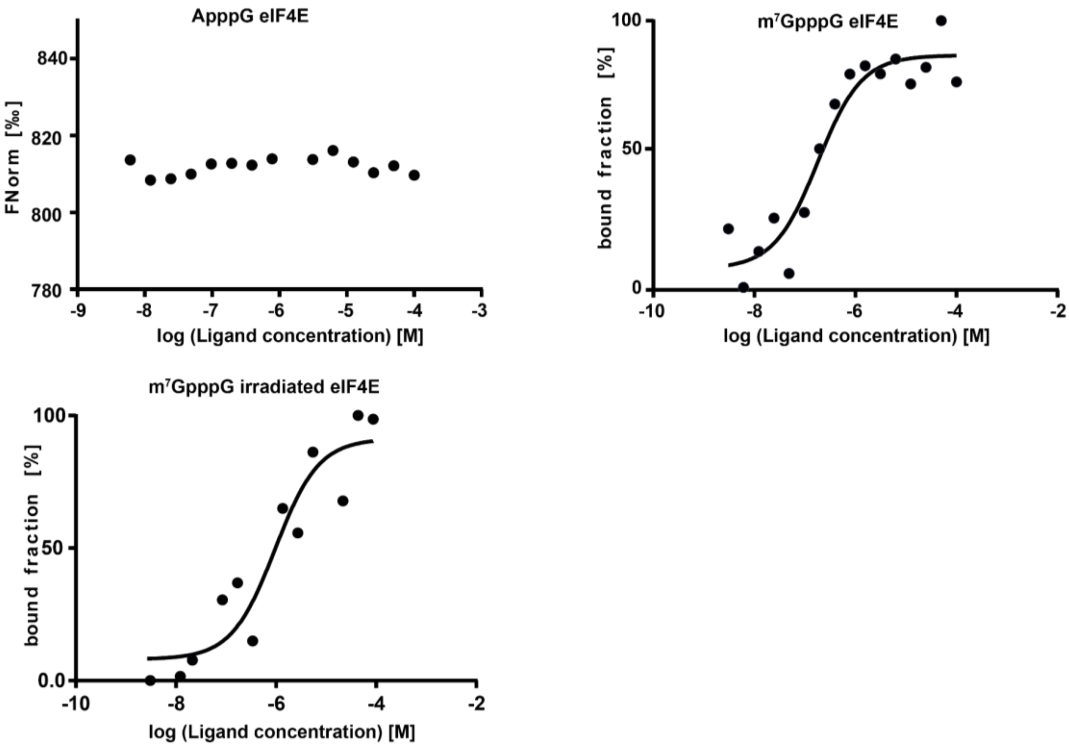

Supplementary Figure 5. Representative MST measurements of indicated cap analogues and Sc. eIF4E. The top left panel shows the results for the negative control ApppG, which is reported to have no affinity to eIF4E.[1,6,7] The right panel depicts the MST measurements of the positive control m^7GpppG, which was reported to have a K_d in the sub micromolar range.[1] The bottom left panel depicts a measurement of the irradiated m^7GpppG (30 s, 365 nm).

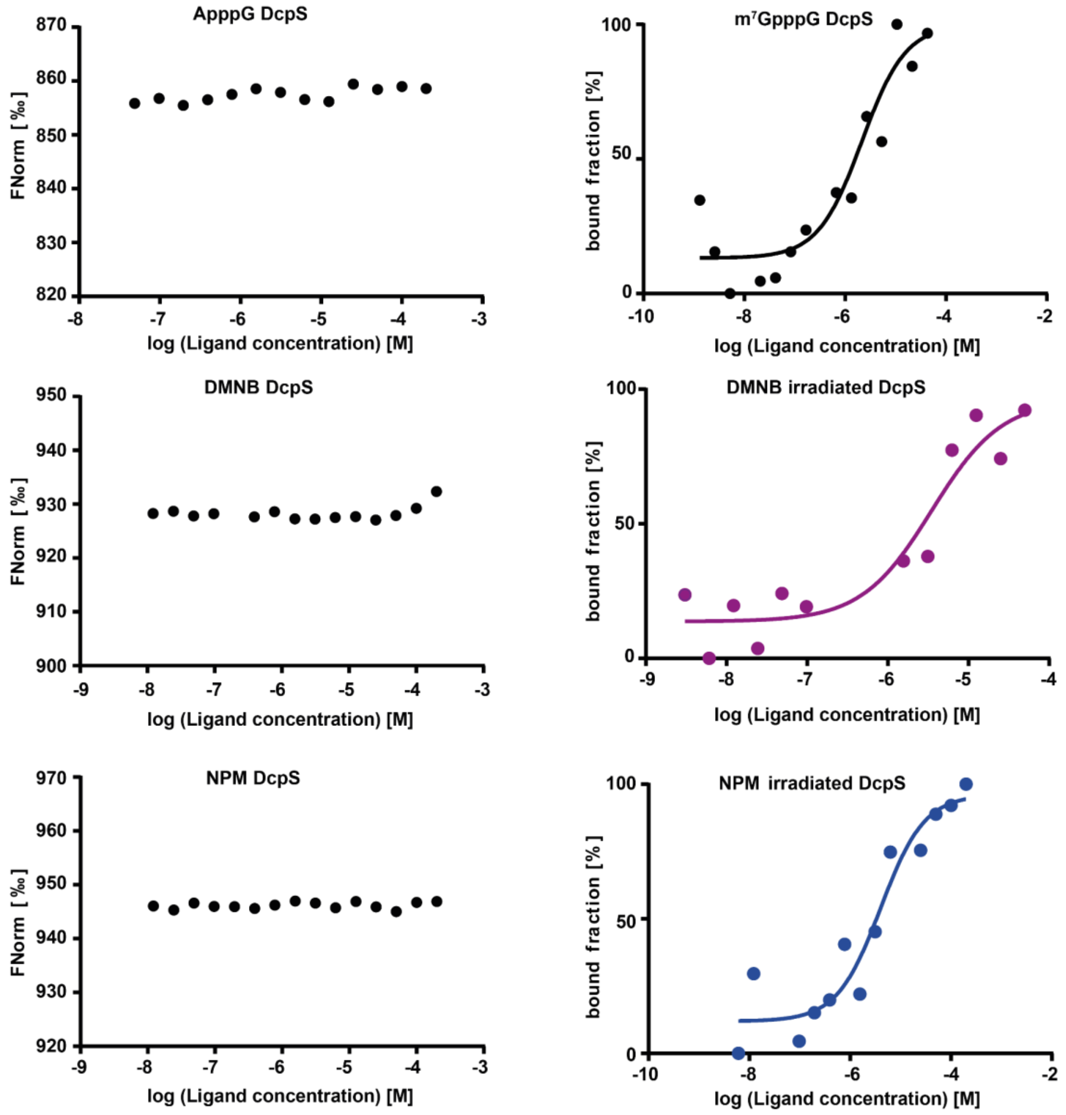

Supplementary Figure 6. Representative MST measurements of the cap analogues and human DcpS H227N. The left three panels show results for the negative control ApppG, which was reported to show no affinity to human DcpS H227N,[1] and the DMNB/NPM cap analogues show similar results. The right three panels depict the MST measurements of the uncaged cap analogues and the positive control m^{7}GpppG, which is known to have a K_d in the lower micromolar region. The curves of the irradiated caged cap analogues indicate that their K_d for human DcpS H227N lies within the same range as m^{7}GpppG.

Supplementary Table 2. Results from MST-based affinity measurements for cap analogues and the cap-binding proteins eIF4E from *S. cerevisiae* and DcpS H227N from humans. The K_d value and standard deviation from average derive from n=3 independent measurements.

Cap	Protein	K_d [µM]	Standard dev. [µM]
**m^{7}GpppG**	*Sc.* eIF4E	0.3	0.1
**m^{7}GpppG irradiated**	*Sc.* eIF4E	0.6	0.4
**DMNB irradiated**	*Sc.* eIF4E	3.4	4.0
**NPM irradiated**	*Sc.* eIF4E	0.3	0.2
**GpppA/NPM/DMNB**	*Sc.* eIF4E	No binding	No binding
**m^{7}GpppG**	*h* DcpS (H227N)	1.7	0.8
**DMNB irradiated**	*h* DcpS (H227N)	6.9	3.0
**NPM irradiated**	*h* DcpS (H227N)	4.0	1.0
**GpppA/NPM/DMNB**	*h* DcpS (H227N)	No binding	No binding

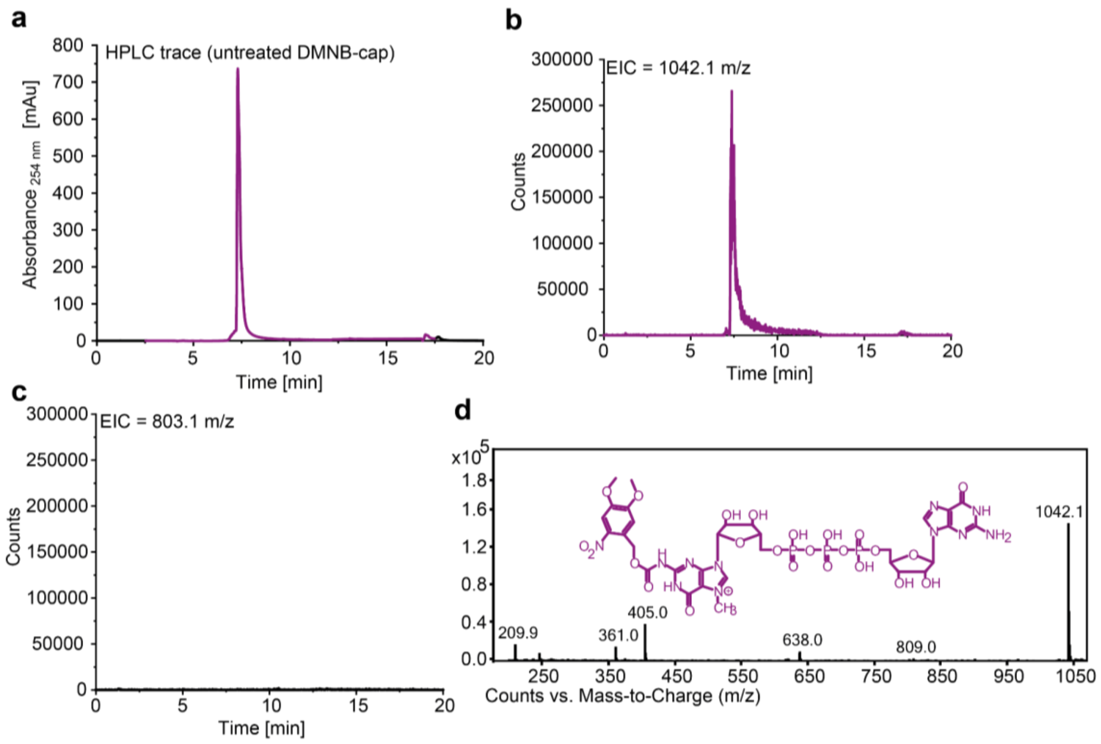

Supplementary Figure 7. LC-MS analysis of non-irradiated DMNB cap analogue. a, HPLC-chromatogram of DMNB cap analogue (t = 7.5 min) before irradiation. **b**, Extracted ion count of 1042.1 m/z corresponding to the calculated mass of the DMNB cap analogue $[C_{31}H_{39}N_{11}O_{24}P_3]^+$= 1042.1377 $[M]^+$. **c**, Extracted ion count of 803.1 m/z corresponding to the calculated mass of the m^{7}GpppG cap analogue $[C_{21}H_{30}N_{10}O_{18}P_3]^+$= 803.0947 $[M]^+$. **d**, ESI-MS spectrum of the main peak at 7.5 minutes retention time in positive mode.

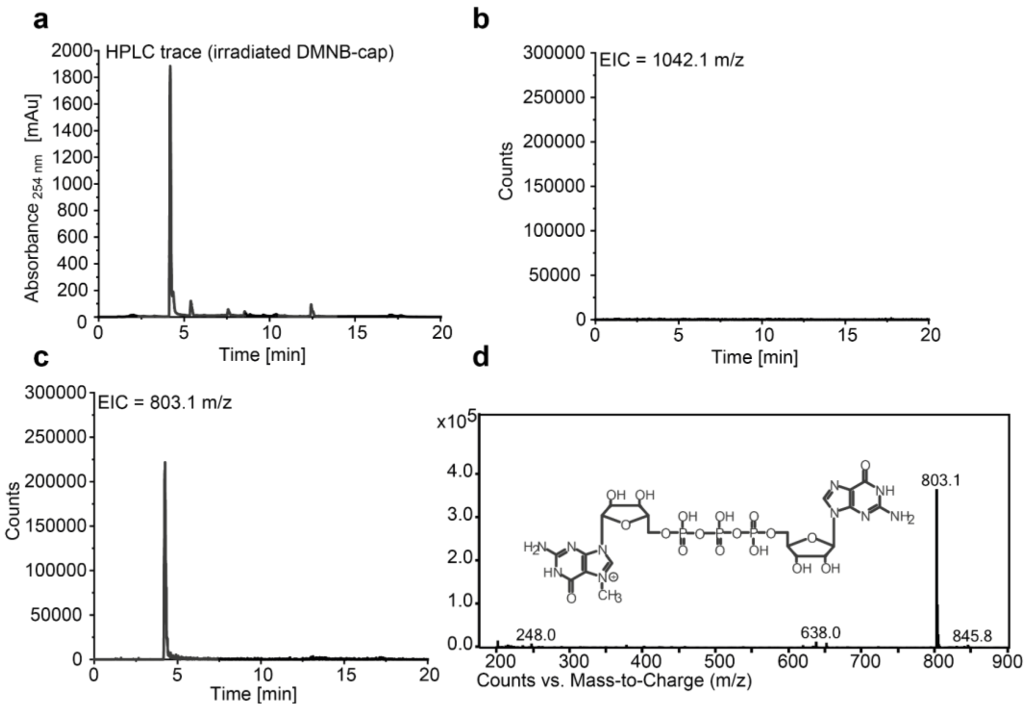

Supplementary Figure 8. LC-MS analysis of irradiated DMNB cap analogue. a, UV trace of irradiated (3 min, 365 nm), pure DMNB cap analogue (t = 4.8 min). **b**, Extracted ion count for 1042.1 m/z corresponding to the calculated mass of the DMNB cap analogue $[C_{31}H_{39}N_{11}O_{24}P_3]^+$= 1042.1377 $[M]^+$. **c**, Extracted ion count of 803.1 m/z corresponding to the calculated mass of the m^{7}GpppG cap analogue $[C_{21}H_{30}N_{10}O_{18}P_3]^+$= 803.0947 $[M]^+$. **d**, ESI-MS spectrum of the main peak at 4.8 minutes retention time in positive mode.

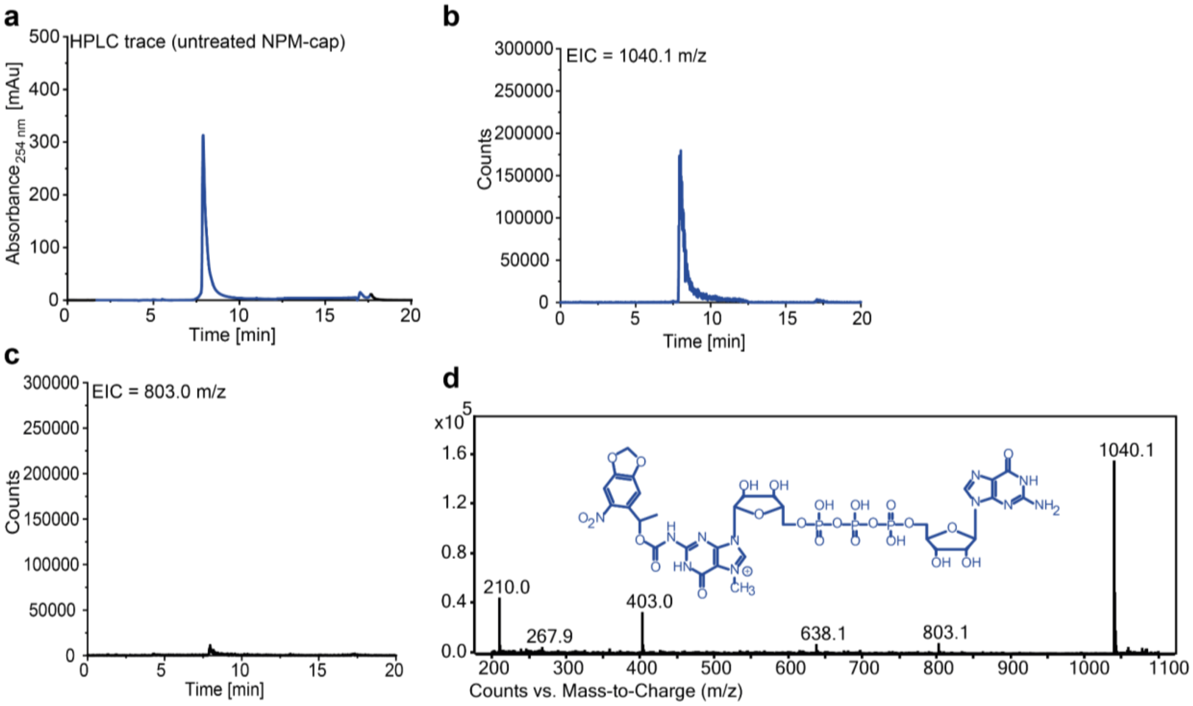

Supplementary Figure 9. LC-MS analysis of non-irradiated NPM cap analogue. a, UV trace of untreated, pure NPM cap analogue (t = 8 min). **b**, Extracted ion count of 1040.1 m/z corresponding to the calculated mass of the NPM cap analogue $[C_{31}H_{37}N_{11}O_{24}P_3]^+$= 1040.1220 $[M]^+$. **c**, Extracted ion count of 803.1 m/z corresponding to the calculated mass of the m^{7}GpppG cap analogue $[C_{21}H_{30}N_{10}O_{18}P_3]^+$= 803.0947 $[M]^+$. **d**, ESI-MS spectrum of the main peak at 8 minutes retention time in positive mode.

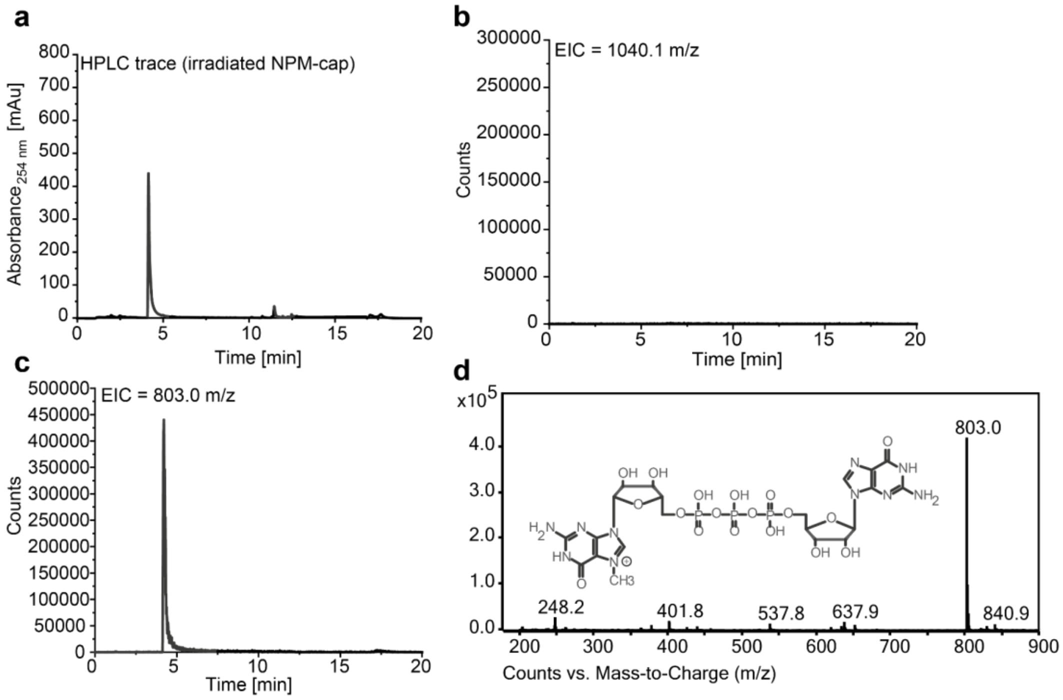

Supplementary Figure 10. LC-MS analysis irradiated NPM cap analogue. a, UV trace of irradiated (3 min, 365 nm), pure NPM cap analogue (t = 8 min). **b**, Extracted ion count of 1040.1

m/z corresponding to the calculated mass of the NPM cap analogue $[C_{31}H_{37}N_{11}O_{24}P_3]^+$= 1040.1220 $[M]^+$. **c**, Extracted ion count of 803.1 m/z corresponding to the calculated mass of the m^7GpppG cap analogue $[C_{21}H_{30}N_{10}O_{18}P_3]^+$= 803.0947 $[M]^+$. **d**, ESI-MS spectrum of the main peak at 4.8 minutes retention time in positive mode.

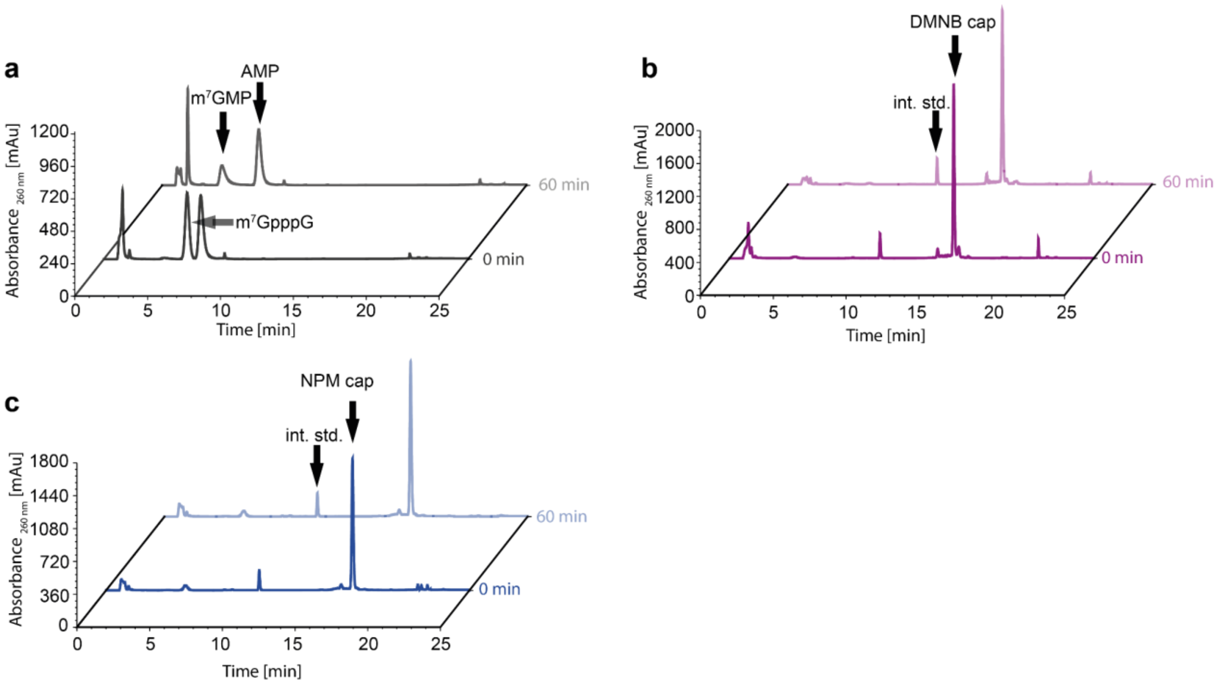

Supplementary Figure 11. Representative HPLC chromatograms of the yDcpS assay. HPLC analysis of the cap analogues in a mixture containing yDcpS (20 U), a decapping enzyme that cleaves the m^7GpppG cap resulting in m^7GMP and GDP, is shown. Each panel direclty compares HPLC analysis before and after incubation at 37 °C for 60 minutes. **a**, Incubation of m^7GpppG, adenine monophosphate (AMP) was used as an internal standard (t = 7 min). The peak with the retention time of 6 minutes represents the cap analogue. GDP (only visible after 60 minutes) is represented by the peak with the retention time of 4 minutes. **b, c**: Similar as for **a**, but for the DMNB cap analogue (t = 15.6 min) (**b**) and the NPM cap analogue (t = 17 min) (**c**). In case of the caged cap analogues 4,5,7-trihydroxy-3-phenylcumarin was used as an internal standard (int. std.) and no decapping is observed.

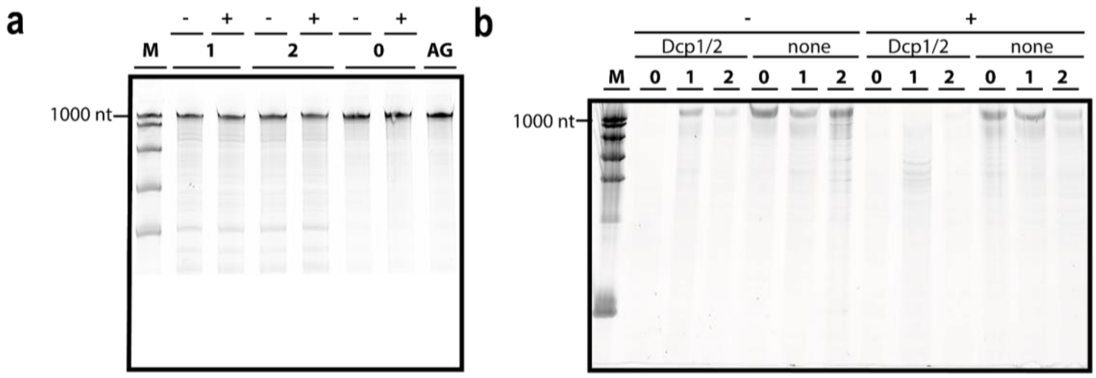

Supplementary Figure 12. Full size gel images corresponding to the gels depicted in Figure 4. a, PAGE analysis of mRNA after IVT with commercial caps (**0**, AG(ApppG)) or FlashCaps (**1**,**2**) unirradiated (-) and irradiated (+) (365 nm, 30 s); M = Marker (Riboruler LR). **b**, Stability of differently capped (**0**,**1**,**2**) mRNA against Dcp1/2 before (-) and after irradiation (+). Samples were either incubated with enzyme (Dcp1/2) or without (none); M = Marker (Riboruler LR). Representative gel image of *n*=3 experiments is shown.

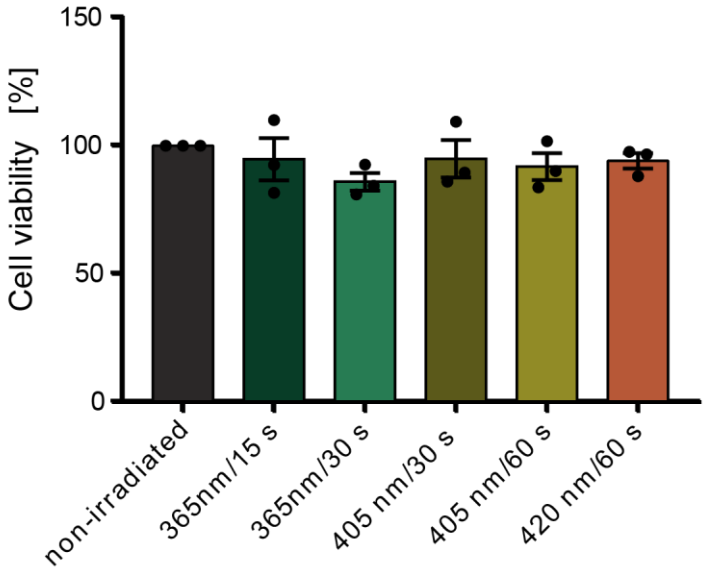

Supplementary Figure 13*. *Cell viability after irradiation of HeLa cells. Viability of HeLa cells seeded on a 96-well plate after irradiation using indicated conditions. Irradiation was performed 6 h post transfection. The MTT assay was conducted 24 h post transfection. The cell viability under different irradiation conditions was normalized to the control (transfected cells but non-irradiated). Data and error bars represent average and standard error of the mean of three independent (*n*=3) cell experiments.

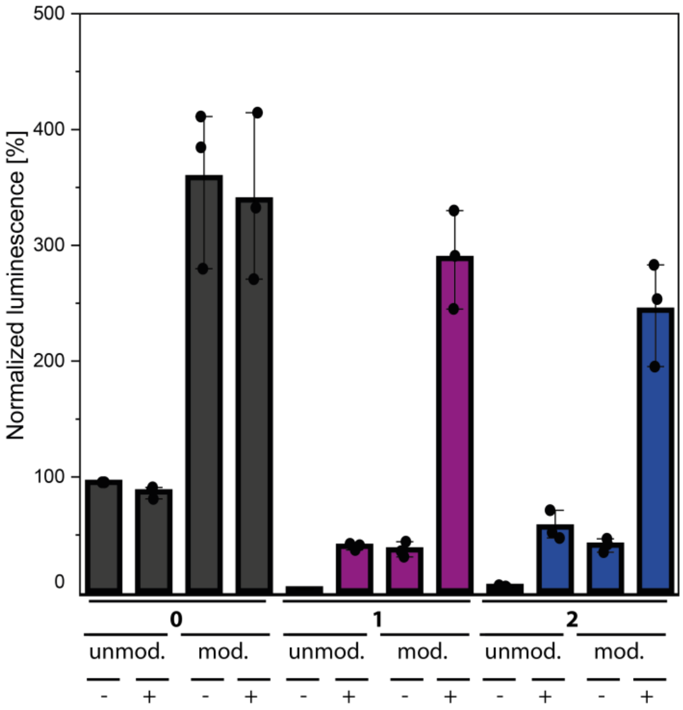

Supplementary Figure 14*. In vitro* translation assay showing luciferase activity relative to the m⁷GpppG-capped FLuc-mRNA without modified nucleotides. The data illustrate the difference in cap-dependent *in vitro* translation between differently capped (**0**;**1**;**2**) mRNAs without modified nucleosides (unmod.) or with m^5C and $m^1\Psi$ (mod.). The samples were either irradiated at 365 nm for 30 seconds (+) or unirradiated (-). Data and error bars represent average and standard error of the mean of three independent (*n*=3) cell experiments.

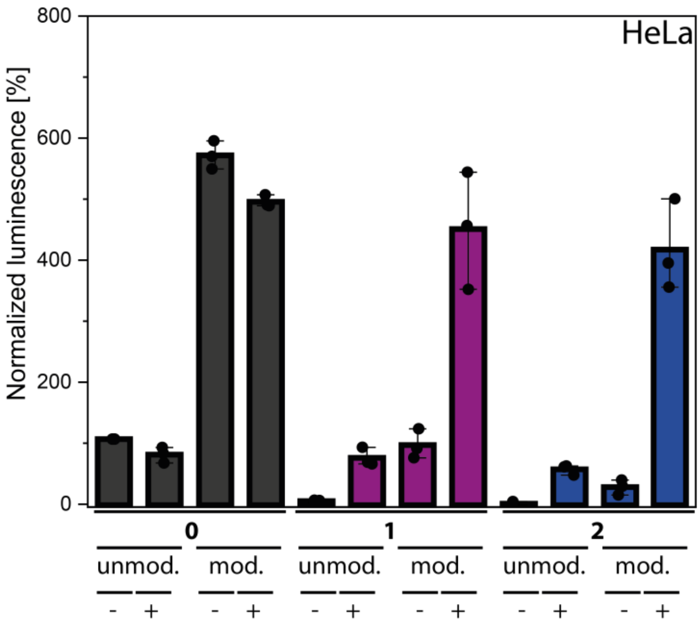

Supplementary Figure 15. In-cell (HeLa) translation assay of FlashCap-mRNAs showing luminescence relative to the m⁷GpppG-capped GLuc-mRNA without modified nucleotides. The data illustrate the difference in cap-dependent translational activity between differently capped (**0**;**1**;**2**) mRNA without modified nucleosides (unmod.) or with m^5C and m1Ψ (mod.) in HeLa cells. The samples were either irradiated with 365 nm for 30 seconds (+) or kept untreated (-). Data and error bars represent average and standard error of the mean of three independent (*n*=3) cell experiments.

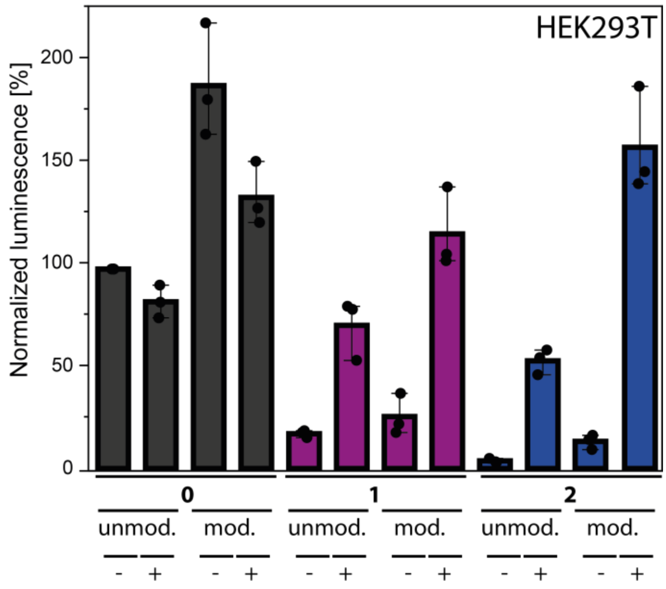

Supplementary Figure 16. In-cell (HEK293T) luminescence translation assay of FlashCap-mRNAs relative to the m^7GpppG capped GLuc-mRNA without modified nucleotides. This data illustrate the difference in cap-dependent translational activity between differently capped (**0**;**1**;**2**) mRNA without m^5C and m1Ψ (unmod.) and with these modified nucleotides (mod.) in

HEK293T cells. The samples were either irradiated with 365 nm for 30 seconds (+) or kept unirradiated (-). Data and error bars represent average and standard error of the mean of three independent (*n*=3) cell experiments.

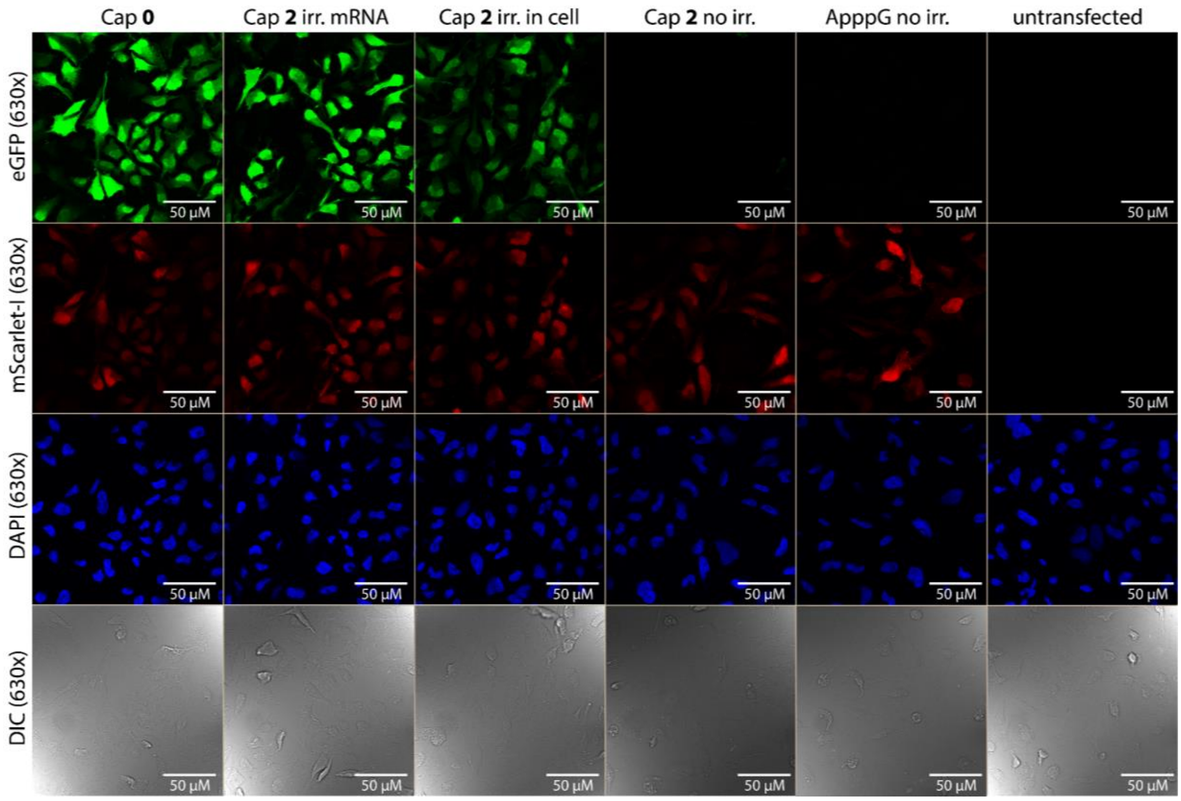

Supplementary Figure 17. Representative 630x magnification confocal microscopy images (as shown in Figure 5 but with DAPI) of irradiated (365 nm) and non-irradiated HeLa cells transfected with eGFP- and mScarlet-I-mRNA with DAPI staining. HeLa cells were transfected with differently capped eGFP-mRNA containing m^5C and m1Ψ and m^7GpppG-capped mScarlet-I-mRNA containing m^5C and $m^1Ψ$. Untransfected cells served as a control. ApppG-capped mRNA represents cap-independent translation. The m^7GpppG-capped eGFP-mRNA (**0**) served as positive control. The NPM-(**2**) caged eGFP-mRNA was either not irradiated (no irr.), irradiated in cells (365 nm, 30 s) (irr. in cell) or irradiated (365 nm, 30 s) before transfection (irr. mRNA). The top two rows show the 630x magnification (63x objective) of the red channel (mScarlet-I) or the green channel (eGFP) while the bottom two show the DAPI staining and DIC channel. Shown is one representative experiment of three independent experiments (*n*=3).

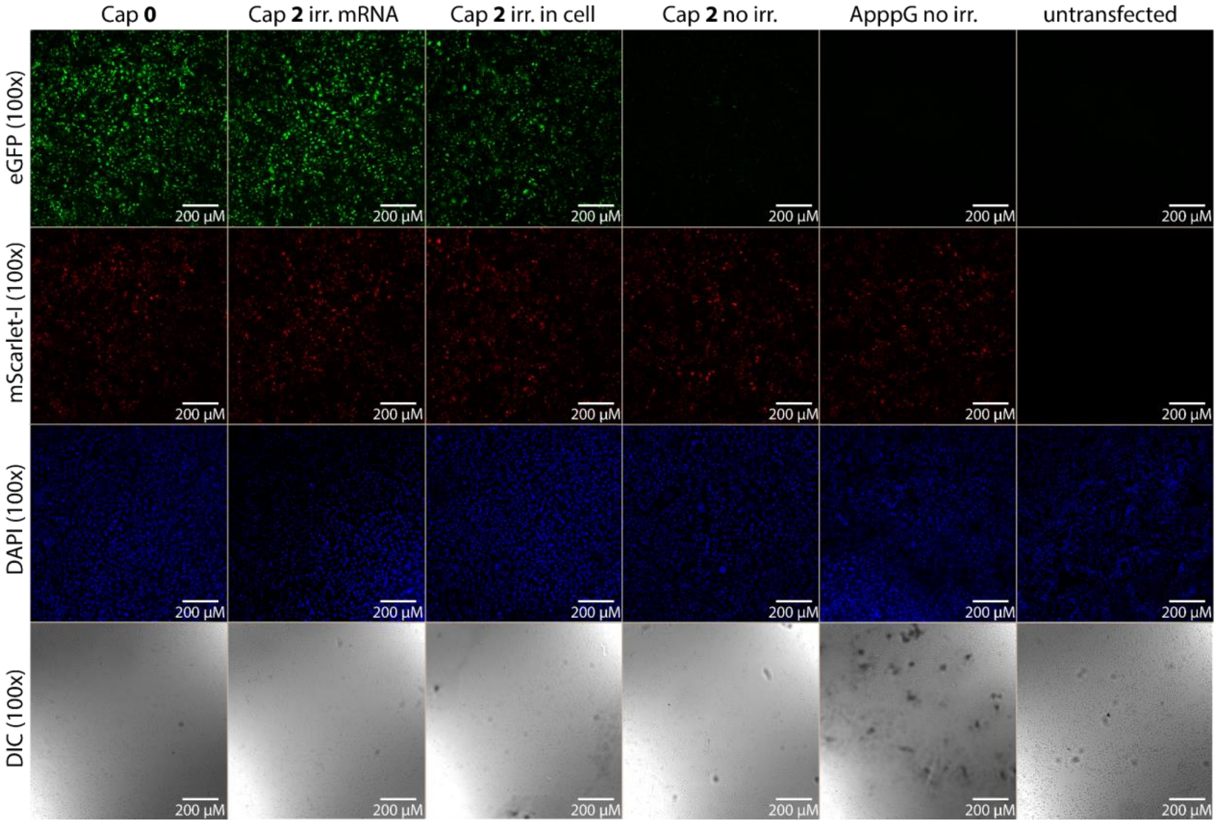

Supplementary Figure 18 Representative 100x magnification confocal microscopy images of the samples shown in Figure 5 and analysed in Suppl. Fig. 19 transfected with eGFP- and mScarlet-I-mRNA with DAPI staining. HeLa cells were transfected with differently capped eGFP-mRNA containing m^5C and $m^1\Psi$ and m^7GpppG-capped mScarlet-I-mRNA containing m^5C and m1Ψ. Untransfected cells served as a control. ApppG-capped mRNA represents cap-independent translation. The m^7GpppG-capped eGFP-mRNA (**0**) served as positive control. The NPM-(**2**) caged eGFP-mRNA was either not irradiated (no irr.), irradiated in cells (irr. In cell; 365 nm, 30 s) or irradiated (365 nm, 30 s) before transfection (irr. mRNA). The top two rows show the 100x magnification (10x objective) of the red channel (mScarlet-I) or the green channel (eGFP) while the bottom two show the DAPI staining and DIC channel. Shown is one representative experiment of three independent experiments (*n*=3).

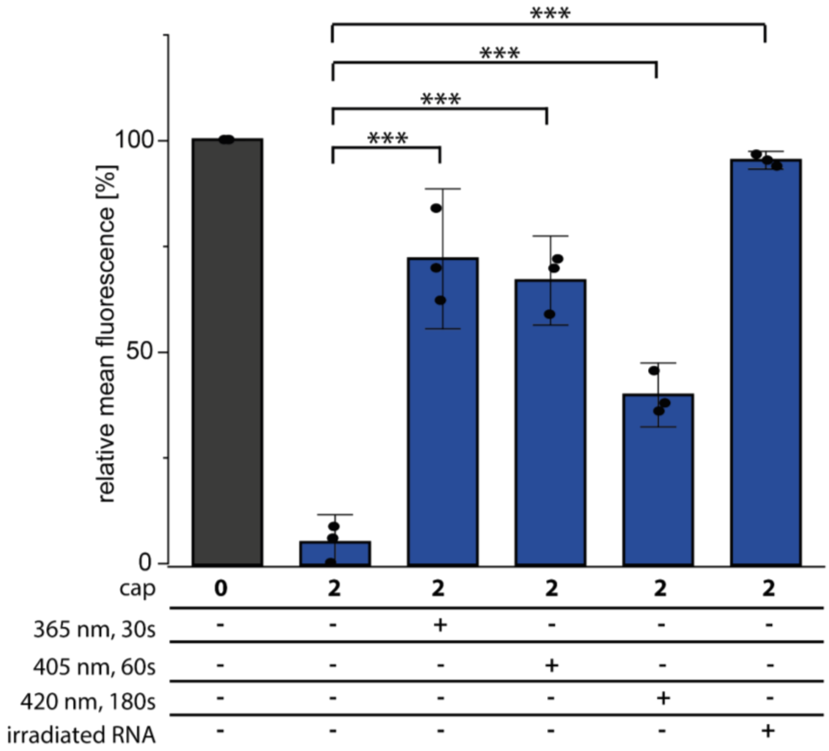

Supplementary Figure 19*. Quantification of green fluorescence from HeLa cells 24 h after transfection with eGFP-mRNA.* The mean eGFP fluorescence in the 100x magnification microscopy images (e.g. Supplementary Fig. 18) was determined using ImageJ and normalized to the positive control (m^7GpppG, cap 0). ApppG-capped mRNA represents cap-independent translation and was subtracted as background from all samples. Statistical significance was determined by two-tailed t-test. Data and error bars represent average and standard error of the mean of three independent (n=3) cell experiments. Significance-levels were defines as p<0.05:*, p<0.01:**, p<0.001:***. P value for **2**(365 nm, 30 s) versus **2**(-) is 6.11×10^{-4}. P value for **2**(405 nm, 60 s) versus **2**(-) is $2{,}03 \times 10^{-4}$. P value for **2**(420 nm, 60 s) versus **2**(-) is 8.2×10^{-4}. P value for **2**(irradiated RNA) versus **2**(-) is 4.46×10^{-6}.

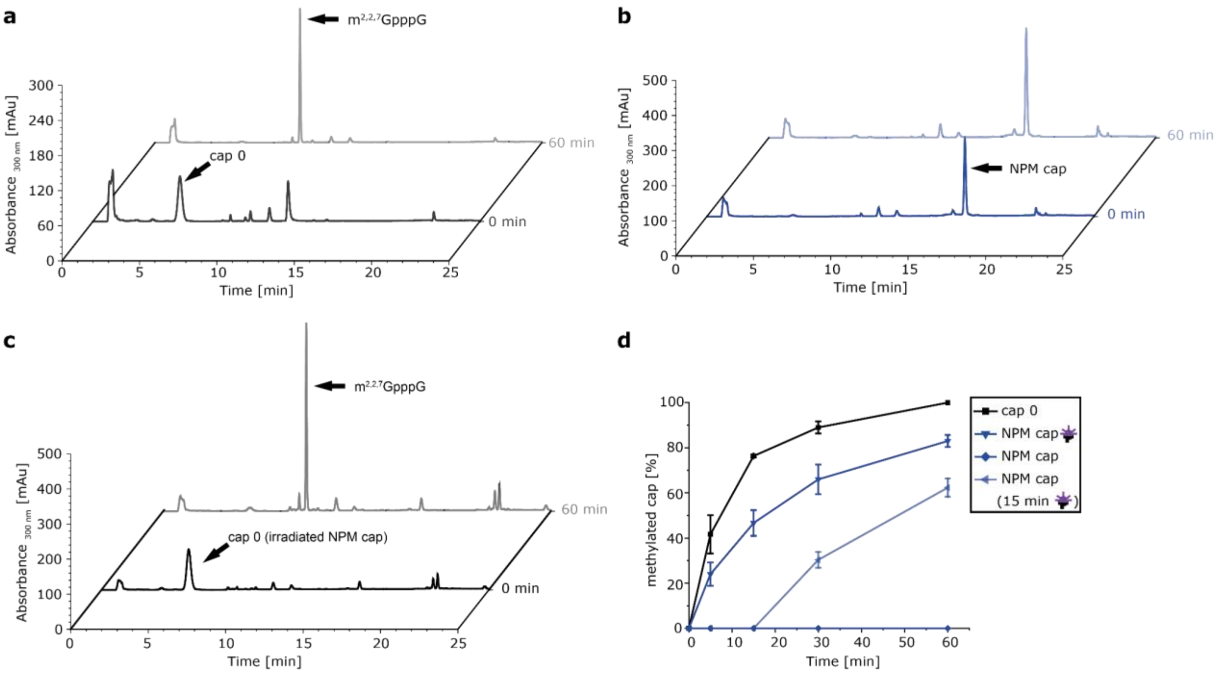

Supplementary Figure 20. Representative chromatograms of the remethylation assay. HPLC analysis of the cap analogues in a mixture containing SAM (6 mM) and hTgs1 (20 µM), an enzyme that methylates the N^2 position of the m^7GpppG cap resulting in $m^{2,2,7}GpppG$, is shown. Each panel compares the HPLC analysis before and after incubation at 37 °C for 60 minutes. **a**, Methylation of m^7GpppG (t = 6 min). The peak with the retention time of 9.5 min represents the dimethylated cap analogue $m^{2,2,7}GpppG$ formed during the reaction. **b**, Methylation of NPM cap analogue (t = 16.8 min). **c**, Methylation of irradiated (365 nm, 30 seconds) NPM cap analogue, i.e. m^7GpppG (t = 6 min). **d**, Graph representing the methylation of the FlashCaps over time. The caps were either irradiated before incubation with the methyl transferase, after 15 minutes of incubation or not at all. Data and error bars represent average and standard deviation of three independent (*n*=3) experiments.

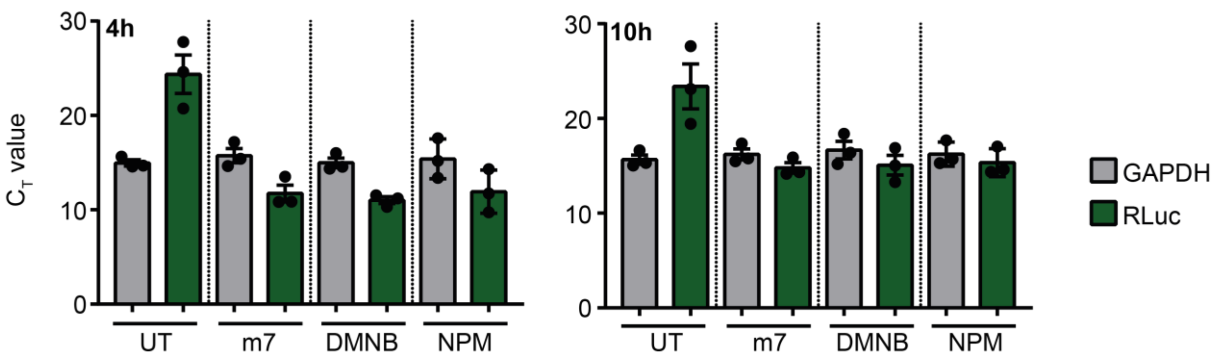

Supplementary Figure 21. Stability of FlashCap-mRNAs. RT-qPCR data showing the C_t values determined for RLuc and GAPDH mRNA at 4 h or 10 h post transfection in HeLa cells. Data and error bars show average and SEM of three independent (*n*=3) cell experiments.

Supplementary Table 3. Promoter sequences used in this study

Promoter	Sequences
**forward**	TAA TAC GAC TCA CTA TAG GGA AAT AAG
**revers**	ATC CAG TCG CGCTGC TCT CGC

Supplementary Table 4. mRNA sequences used in this study

Construct	RNA sequences
**eGFP**	5′-<br>GGGAAAUAAGAGAGAAAAGAAGAGUAAGAAGAAAUAUAAGAGAAUUCGCCACCAUG<br>GUGAGCAAGGGCGAGGAGCUGUUCACCGGGGUGGUGCCCAUCCUGGUCGAGCUGG<br>ACGGCGACGUAAACGGCCACAAGUUCAGCGUGUCCGGCGAGGGCGAGGGCGAUGC<br>CACCUACGGCAAGCUGACCCUGAAGUUCAUCUGCACCACCGGCAAGCUGCCCGUGC<br>CCUGGCCCACCCUCGUGACCACCCUGACCUACGGCGUGCAGUGCUUCAGCCGCUAC<br>CCCGACCACAUGAAGCAGCACGACUUCUUCAAGUCCGCCAUGCCCGAAGGCUACGU<br>CCAGGAGCGCACCAUCUUCUUCAAGGACGACGGCAACUACAAGACCCGCGCCGAGG<br>UGAAGUUCGAGGGCGACACCCUGGUGAACCGCAUCGAGCUGAAGGGCAUCGACUU<br>CAAGGAGGACGGCAACAUCCUGGGGCACAAGCUGGAGUACAACUACAACAGCCACA<br>ACGUCUAUAUCAUGGCCGACAAGCAGAAGAACGGCAUCAAGGUGAACUUCAAGAUC<br>CGCCACAACAUCGAGGACGGCAGCGUGCAGCUCGCCGACCACUACCAGCAGAACAC<br>CCCCAUCGGCGACGGCCCCGUGCUGCUGCCCGACAACCACUACCUGAGCACCCAGU<br>CCGCCCUGAGCAAAGACCCCAACGAGAAGCGCGAUCACAUGGUCCUGCUGGAGUUC<br>GUGACCGCCGCCGGGAUCACUCUCGGCAUGGACGAGCUGUACAAGUAGGGAUCCG<br>CUGCCUUCUGCGGGGCUUGCCUUCUGGCCAUGCCCUUCUUCUCUCCCUUGCACCU<br>GUACCUCUUGGUCUUUGAAUAAAGCCUGAGUAGGAAGAAUAUUAAAAAAAAAAAAA<br>AAAAAAAAAAAAAAAAAAAAAAAAAAAAAAAAUUAAU-3′
**FLuc**	5′-<br>GGGAAAUAAGAGAGAAAAGAAGAGUAAGAAGAAAUAUAAGAGAAUUCGCCACCAUG<br>GAAGACGCCAAAAACAUAAAGAAAGGCCCGGCGCCAUUCUAUCCGCUGGAAGAUGG<br>AACCGCUGGAGAGCAACUGCAUAAGGCUAUGAAGAGAUACGCCCUGGUUCCUGGAA<br>CAAUUGCUUUUACAGAUGCACAUAUCGAGGUGGACAUCACUUACGCUGAGUACUUC<br>GAAAUGUCCGUUCGGUUGGCAGAAGCUAUGAAACGAUAUGGGCUGAAUACAAAUCA<br>CAGAAUCGUCGUAUGCAGUGAAAACUCUCUUCAAUUCUUUAUGCCGGUGUUGGGC<br>GCGUUAUUUAUCGGAGUUGCAGUUGCGCCCGCGAACGACAUUUAUAAUGAACGUG<br>AAUUGCUCAACAGUAUGGGCAUUUCGCAGCCUACCGUGGUGUUCGUUUCCAAAAG<br>GGGUUGCAAAAAAUUUUGAACGUGCAAAAAAAGCUCCCAAUCAUCCAAAAAAUUAU<br>UAUCAUGGAUUCUAAAACGGAUUACCAGGGAUUUCAGUCGAUGUACACGUUCGUCA<br>CAUCUCAUCUACCUCCCGGUUUUAAUGAAUACGAUUUUGUGCCAGAGUCCUUCGAU<br>AGGGACAAGACAAUUGCACUGAUCAUGAACUCCUCUGGAUCUACUGGUCUGCCUAA<br>AGGUGUCGCUCUGCCUCAUAGAACUGCCUGCGUGAGAUUCUCGCAUGCCAGAGAU<br>CCUAUUUUUGGCAAUCAAAUCAUUCCGGAUACUGCGAUUUUAAGUGUUGUUCCAUU<br>CCAUCACGGUUUUGGAAUGUUUACUACACUCGGAUAUUUGAUAUGUGGAUUUCGA<br>GUCGUCUUAAUGUAUAGAUUUGAAGAAGAGCUGUUUCUGAGGAGCCUUCAGGAUU<br>ACAAGAUUCAAAGUGCGCUGCUGGUGCCAACCCUAUUCUCCUUCUUCGCCAAAAGC<br>ACUCUGAUUGACAAAUACGAUUUAUCUAAUUUACACGAAAUUGCUUCUGGUGGCGC<br>UCCCCUCUCUAAGGAAGUCGGGGAAGCGGUUGCCAAGAGGUUCCAUCUGCCAGGU<br>AUCAGGCAAGGAUAUGGGCUCACUGAAACUACUUCAGCUAUUCUGAUUACCCCCGA<br>GGGGAGGCAAGGAUAUGGGCUCACUGAGACUACAUCAGCUAUUCUGAUUACACCC<br>GAGGGGGAUGAUAAACCGGGCGCGGUCGGUAAAGUUGUUCCAUUUUUUGAAGCGA<br>AGGUUGUGGAUCUGGAUACCGGGAAAACGCUGGGCGUUAAUCAAAGAGGCGAACU<br>GUGUGUGAGAGGUCCUAUGAUUAUGUCCGGUUAUGUAAACAAUCCGGAAGCGACC<br>AACGCCUUGAUUGACAAGGAUGGAUGGCUACAUUCUGGAGACAUAGCUUACUGGG<br>ACGAAGACGAACACUUCUUCAUCGUUGACCGCCUGAAGUCUCUGAUUAAGUACAAA<br>GGCUAUCAGGUGGCUCCCGCUGAAUUGGAAUCCAUCUUGCUCCAACACCCCAACAU<br>CUUCGACGCAGGUGUCGCAGGUCUUCCCGACGAUGACGCCGGUGAACUUCCCGCC<br>GCCGUUGUUGUUUUGGAGCACGGAAAGACGAUGACGGAAAAAGAGAUCGUGGAUU<br>ACGUCGCCAGUCAAGUAACAACCGCGAAAAAGUUGCGCGGAGGAGUUGUGUUUGU<br>GGACGAAGUACCGAAAGGUCUUACCGGAAAACUCGACGCAAGAAAAAUCAGAGAGA<br>UCCUCAUAAAGGCCAAGAAGGGCGGAAAGAUCGCCGUGUAAGGAUCCGCUGCCUUC<br>UGCGGGGCUUGCCUUCUGGCCAUGCCCUUCUUCUCUCCCUUGCACCUGUACCUCU

	UGGUCUUUGAAUAAAGCCUGAGUAGGAAGAAUAUUAAAAAAAAAAAAAAAAAAAAAA AAAAAAAAAAAAAAAAAAAAAAAAUUAAU-3′
**GLuc**	5′- GGGAAAUAAGAGAGAAAAGAAGAGUAAGAAGAAAUAUAAGAGAAUUCGCCACCAUG GGAAAUAAGAGAGAAAAGAAGAGUAAGAAGAAAUAUAAGAGAAUUCGCCACCAUGG GAGUCAAAGUUCUGUUUGCCCUGAUCUGCAUCGCUGUGGCCGAGGCCAAGCCCAC CGAGAACAACGAAGACUUCAACAUCGUGGCCGUGGCCAGCAACUUCGCGACCACGG AUCUCGAUGCUGACCGCGGGAAGUUGCCCGGCAAGAAGCUGCCGCUGGAGGUGCU CAAAGAGAUGGAAGCCAAUGCCCGGAAAGCUGGCUGCACCAGGGGCUGUCUGAUC UGCCUGUCCCACAUCAAGUGCACGCCCAAGAUGAAGAAGUUCAUCCCAGGACGCUG CCACACCUACGAAGGCGACAAAGAGUCCGCACAGGGCGGCAUAGGCGAGGCGAUCG UCGACAUUCCUGAGAUUCCUGGGUUCAAGGACUUGGAGCCCAUGGAGCAGUUCAU CGCACAGGUCGAUCUGUGUGUGGACUGCACAACUGGCUGCCUCAAAGGGCUUGCC AACGUGCAGUGUUCUGACCUGCUCAAGAAGUGGCUGCCGCAACGCUGUGCGACCU UUGCCAGCAAGAUCCAGGGCCAGGUGGACAAGAUCAAGGGGGCCGGUGGUGACUA AGGAUCCGCUGCCUUCUGCGGGGCUUGCCUUCUGGCCAUGCCCUUCUUCUCUCCC UUGCACCUGUACCUCUUGGUCUUUGAAUAAAGCCUGAGUAGGAAGAAUAUUAAAAA AAAAAAAAAAAAAAAAAAAAAAAAAAAAAAAAAAAAAAUUAAU-3′
**mScarlet-I 128 poly A**	5′ GGGAGAGAUAUCACGCGUUCUAGAGCUAGCGCUACCGGACUCAGAUCUCGAGCUCA AGCUUCGAAUUCUGCAGUCGACGGUACCGCGGGCCCGGGAUCCACCUCGCCACCAU GAGUAAAGGAGAAGCUGUGAUUAAAGAGUUCAUGCGCUUCAAAGUUCACAUGGAG GGUUCUAUGAACGGUCACGAGUUCGAGAUCGAAGGCGAAGGCGAGGGCCGUCCGU AUGAAGGCACCCAGACCGCCAAACUGAAAGUGACUAAAGGCGGCCCGCUGCCUUUU UCCUGGGACAUCCUGAGCCCGCAAUUUAUGUACGGUUCUAGGGCGUUCAUCAAACA CCCAGCGGAUAUCCCGGACUAUUAUAAGCAGUCUUUUCCGGAAGGUUUCAAGUGG GAACGCGUAAUGAAUUUUGAAGAUGGUGGUGCCGUGACCGUCACUCAGGACACCU CCCUGGAGGAUGGCACCCUGAUCUAUAAAGUUAAACUGCGUGGUACUAAUUUCCA CCUGAUGGCCCGGUGAUGCAGAAAAAGACGAUGGGUUGGGAGGCGUCUACCGAAC GCUUGUAUCCGGAAGAUGGUGUGCUGAAAGGCGACAUUAAAAUGGCCCUGCGCCU GAAAGAUGGUGGCCGCUAUCUGGCUGACUUCAAAACCACGUACAAAGCCAAGAAAC CUGUGCAGAUGCCUGGCGCGUACAAUGUGGACCGCAAACUGGACAUCACCUCUCAU AAUGAAGAUUAUACGGUGGUAGAGCAAUAUGAGCGCUCCGAGGGUCGUCAUUCUA CCGGUGGCAUGGAUGAACUAUACAAAUAAAGCGGCCGCAGCUCGCUUUCUUGCUG UCCAAUUUCUAUUAAAGGUUCCUUUGUUCCCUAAGUCCAACUACUAAACUGGGGGA UAUUAUGAAGGGCCUUGAGCAUUUGGAUUCUGCCUAAUAAAAAACAUUUAUUUUCA UUGCGUUUAGCUCGCUUUCUUGCUGUCCAAUUUCUAUUAAAGGUUCCUUUGUUCC CUAAGUCCAACUACUAAACUGGGGGAUAUUAUGAAGGGCCUUGAGCAUUUGGAUUC UGCCUAAUAAAAAACAUUUAUUUUCAUUGAAAAAAAAAAAAAAAAAAAAAAAAAAAAAA AAAAAAAAAAAAAAAAAAAAAAAAAAAAAAAAAAAAAAAAAAAAAAAAAAAAAAAAAA AAAAAAAAAAAAAAAAAAAAAAAAAAAAAAAAAAAAAAAA-3′
**eGFP 128 poly A**	5′ GGGAGAGAUAUCACGCGUUCUAGAGCUAGCGCUACCGGACUCAGAUCUCGAGCUCA AGCUUCGAAUUCUGCAGUCGACGGUACCGCGGGCCCGGGAUCCACCGGUCGCCAC CAUGGUGAGCAAGGGCGAGGAGCUGUUCACCGGGGUGGUGCCCAUCCUGGUCGAG CUGGACGGCGACGUAAACGGCCACAAGUUCAGCGUGUCCGGCGAGGGCGAGGGCG AUGCCACCUACGGCAAGCUGACCCUGAAGUUCAUCUGCACCACCGGCAAGCUGCCC GUGCCCUGGCCCACCCUCGUGACCACCCUGACCUACGGCGUGCAGUGCUUCAGCCG CUACCCCGACCACAUGAAGCAGCACGACUUCUUCAAGUCCGCCAUGCCCGAAGGCU ACGUCCAGGAGCGCACCAUCUUCUUCAAGGACGACGGCAACUACAAGACCCGCGCC GAGGUGAAGUUCGAGGGCGACACCCUGGUGAACCGCAUCGAGCUGAAGGGCAUCG ACUUCAAGGAGGACGGCAACAUCCUGGGGCACAAGCUGGAGUACAACUACAACAGC CACAACGUCUAUAUCAUGGCCGACAAGCAGAAGAACGGCAUCAAGGUGAACUUCAA GAUCCGCCACAACAUCGAGGACGGCAGCGUGCAGCUCGCCGACCACUACCAGCAGA ACACCCCCAUCGGCGACGGCCCCGUGCUGCUGCCCGACAACCACUACCUGAGCACC CAGUCCGCCCUGAGCAAAGACCCCAACGAGAAGCGCGAUCACAUGGUCCUGCUGGA GUUCGUGACCGCCGCCGGGAUCACUCUCGGCAUGGACGAGCUGUACAAGUAAAGC GGCCGCAGCUCGCUUUCUUGCUGUCCAAUUUCUAUUAAAGGUUCCUUUGUUCCCUA AGUCCAACUACUAAACUGGGGGAUAUUAUGAAGGGCCUUGAGCAUUUGGAUUCUG CCUAAUAAAAAACAUUUAUUUUCAUUGCGUUUAGCUCGCUUUCUUGCUGUCCAAUU UCUAUUAAAGGUUCCUUUGUUCCCUAAGUCCAACUACUAAACUGGGGGAUAUUAUG AAGGGCCUUGAGCAUUUGGAUUCUGCCUAAUAAAAAACAUUUAUUUUCAUUGAAAA AAAAAAAAAAAAAAAAAAAAAAAAAAAAAAAAAAAAAAAAAAAAAAAAAAAAAAAAAA

	AAAAAAAAAAAAAAAAAAAAAAAAAAAAAAAAAAAAAAAAAAAAAAAAAAAAAAAAAAAA<br>AAAAAA-3′
**Rheb_eGFP**	5′-<br>GGGGAAUUGUGAGCGGAUAACAAUUCCCCUCUAGAAAUAAUUUUGUUUAACUUUAA<br>GAAGGAGAUAUACCAUGGGCAGCAGCCAUCAUCAUCAUCAUCACAGCAGCGGCCUG<br>GUGCCGCGCGGCAGCCAUAUGGCGAUGGUGAGCAAGGGCGAGGAGCUGUUCACCG<br>GGGUGGUGCCCAUCCUGGUCGAGCUGGACGGCGACGUAAACGGCCACAAGUUCAG<br>CGUGUCCGGCGAGGGCGAGGGCGAUGCCACCUACGGCAAGCUGACCCUGAAGUUC<br>AUCUGCACCACCGGCAAGCUGCCCGUGCCCUGGCCCACCCUCGUGACCACCCUGAC<br>CUACGGCGUGCAGUGCUUCAGCCGCUACCCCGACCACAUGAAGCAGCACGACUUCU<br>UCAAGUCCGCCAUGCCCGAAGGCUACGUCCAGGAGCGCACCAUCUUCUUCAAGGAC<br>GACGGCAACUACAAGACCCGCGCCGAGGUGAAGUUCGAGGGCGACACCCUGGUGA<br>ACCGCAUCGAGCUGAAGGGCAUCGACUUCAAGGAGGACGGCAACAUCCUGGGGCA<br>CAAGCUGGAGUACAACUACAACAGCCACAACGUCUAUAUCAUGGCCGACAAGCAGA<br>AGAACGGCAUCAAGGUGAACUUCAAGAUCCGCCACAACAUCGAGGACGGCAGCGUG<br>CAGCUCGCCGACCACUACCAGCAGAACACCCCCAUCGGCGACGGCCCCGUGCUGCU<br>GCCCGACAACCACUACCUGAGCACCCAGUCCAAGCUGAGCAAAGACCCCAACGAGAA<br>GCGCGAUCACAUGGUCCUGCUGGAGUUCGUGACCGCCGCCGGGAUCACUCUCGGC<br>AUGGACGAGCUGUACAAGGGAUCCAUGCCGCAGUCCAAGUCCCGGAAGAUCGCGAU<br>CCUGGGCUACCGGUCUGUGGGGAAAUCCUCAUUGACGAUUCAAUUUGUUGAAGGC<br>CAAUUUGUGGACUCCUACGAUCCAACCAUAGAAAACACUUUUACAAAGUUGAUCAC<br>AGUAAAUGGACAAGAAUAUCAUCUUCAACUUGUAGACACAGCCGGGCAAGAUGAAU<br>AUUCUAUCUUUCCUCAGACAUACUCCAUAGAUAUUAAUGGCUAUAUUCUUGUGUAU<br>UCUGUUACAUCAAUCAAAAGUUUUGAAGUGAUUAAAGUUAUCCAUGGCAAAUUGUU<br>GGAUAUGGUGGGGAAAGUACAAAUACCUAUUAUGUUGGUUGGGAAUAAGAAAGAC<br>CUGCAUAUGGAAAGGGUGAUCAGUUAUGAAGAAGGGAAAGCUUUGGCAGAAUCUU<br>GGAAUGCAGCUUUUUUGGAAUCUUCUGCUAAAGAAAAUCAGACUGCUGUGGAUGU<br>UUUUCGAAGGAUAAUUUUGGAGGCAGAAAAAAUGGACGGGGCAGCUUCACAAGGC<br>AAGUCUUCAUGCUCGGUGAUGUGAGCGGCCGCAC-3′

Synthetic procedures

Guanosine 5′-diphosphate imidazolide (**8**) was synthesized from commercially available Guanosine 5′-diphosphate disodium salt hydrate [CAS: 43139-22-6], supplier (Biosynth-Carbosynth, product code: NG08963). Procedure of Jemielity *et al.*[8]: Guanosine 5′-diphosphate imidazolide was synthesized by changing guanosine 5′-diphosphate Na^{+} salt (1.11 g, 1.49 mmol, 1 eq.) into the TEA salt on Dowex 50WX8 resin and then mixing it with imidazole (509 mg, 7.47 mmol, 5 eq.) and 2,2′-dithiodipyridine (657 mg, 2.98 mmol, 2 eq.) in anhydrous DMF (15 mL) and TEA (207 µL). Triphenylphosphine (782 mg, 2.98 mmol, 2 eq.) was added, and the mixture was stirred at room temperature overnight. The mixture was poured in a flask containing anhydrous sodium perchlorate (1.2 g) dissolved in 60 mL of dry acetone. After cooling at 4 °C for 2 h, the precipitate was filtered, washed twice with 15 mL of cold acetone, and dried in a vacuum desiccator over P_4O_{10} (yield: 725 mg, 91%).

HRMS (ESI-) m/z: calculated for $C_{13}H_{17}N_7O_{10}P_2$ $[M-H]^-$: 492.04394 found, 492.04246

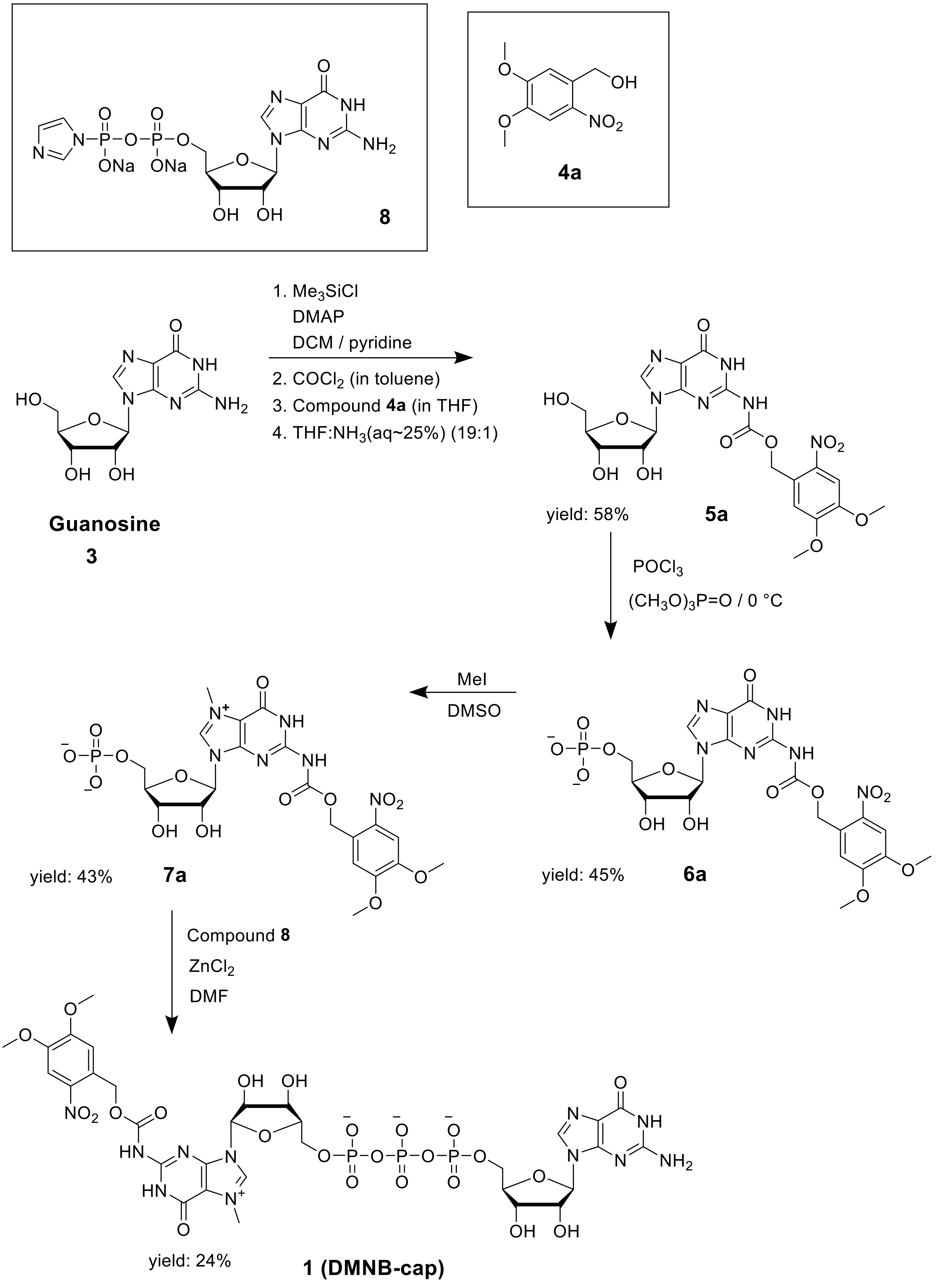

8
4a
1. Me3SiCl
DMAP
DCM / pyridine
2. COCl2 (in toluene)
3. Compound 4a (in THF)
4. THF:NH3(aq~25%) (19:1)
Guanosine
3
yield: 58%
5a
POCl3
(CH3O)3P=O / 0 °C
MeI
DMSO
yield: 43%
7a
yield: 45%
6a
Compound 8
ZnCl2
DMF
yield: 24%
1 (DMNB-cap)

N^2-(4,5-Dimethoxy-2-nitrobenzyl)oxycarbonylguanosine (5a)

Guanosine (**3**) (850 mg, 3 mmol, 1 eq.) and 4-dimethylaminopyridine (40 mg, 0.33 mmol, 0.1 eq.) were suspended in dry DCM (60 mL) in a round bottom schlenk flask under argon atmosphere. The mixture was cooled by an ice-water bath and dry pyridine (10 mL) was added subsequently. Trimethylsilyl chloride (2.4 mL, 18.9 mmol, 6.3 eq.) was added dropwise and the reaction mixture was stirred for 2 hours. Phosgene in toluene (3 mL of 15wt% solution = roughly 4.2 mmol, 1.4 eq.) was added dropwise at 0 °C. The formation of a yellow precipitate was observed. The reaction mixture was stirred for futher 30 minutes. Subsequently, a solution of 4,5-dimethoxy-2-nitrobenzyl alcohol (**4a**) (2.4 g, 11.3 mmol, 3.8 eq.) in dry THF (52 mL) was added and reaction mixture was stirred overnight to gradually warm up to room temperature. The reaction mixture was mixed with chloroform (100 mL) and demineralized water (100 mL). The organic layer was extracted with demineralized water (3x 100 mL) and the solvent was evaporated under reduced pressure. The obtained brown oily solid was dissolved in the THF (50 mL) and aqueous ammonia (2.5 mL of 25% solution) was added. The mixture was stirred vigorously overnight to form a white-beige precipitate. The suspended precipitate was centrifuged (3220 rcf, 4 °C, 10 min), the supernatant was removed and the precipitate was freeze-dried to obtain compound **5a** as a white-beige solid (916 mg, 1.75 mmol, yield: 58%).

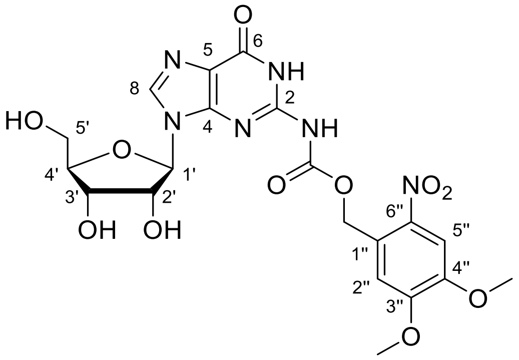

^{1}H NMR (600 MHz, DMSO-*d6*): δ 8.04 [s, 1H, H-8]; 7.72 [s, 1H, H-5″]; 7.33 [s, 1H, H-2″]; 5.75 [d, 1H, *J* = 6.1 Hz, H-1′]; 5.46 [s, 2H, $\underline{CH_2}$-Ar]; 4.49 [dd, 1H, *J* = 6.1, 5.0 Hz, H-2′]; 4.11 [dd, 1H, *J* = 5.0, 3.2 Hz, H-3′]; 3.93

[s, 3H, O-CH_3]; 3.90 [q, 1H, J = 3.6 Hz, H-4′]; 3.88 [s, 3H, O-CH_3]; 3.63 [dd, 1H, H-5′^{a}]; 3.52 [dd, 1H, H-5′^{b}]

^{13}C NMR (151 MHz, DMSO-*d6*): δ 156.39 [C-6]; 153.45 [-NH-CO-OAr]; 150.35 [C-4]; 147.64 [Ar]; 139.15 [Ar]; 137.07 [C-8]; 127.92 [Ar]; 119.08 [C-5]; 110.59 [Ar]; 108.09 [Ar]; 87.15 [C-1′]; 85.63 [C-4′]; 73.62 [C-2′]; 70.53 [C-3′]; 63.27 [-CH_2-Ar]; 61.55 [C-5′]; 56.34 [O-CH_3]; 56.08 [O-CH_3]

HRMS (ESI+) m/z: calculated for $C_{20}H_{22}N_6O_{11}$ $[M+H]^+$: 523.14193, found 523.14336.

N^2-(4,5-Dimethoxy-2-nitrobenzyl)oxycarbonylguanosine 5′-monophosphate (6a)

The guanosine derivative **5a** (313 mg, 599 µmol, 1 eq.) was suspended in dry trimethyl phosphate (TMP) (5 mL). The solution was cooled to 0 °C and freshly distilled phosphoryl chloride (73 µL, 119 mg, 779 µmol, 1.3 eq.) was added slowly. The reaction mixture was stirred for 1 hour. Subsequently, a precooled solution of TEAB (10 mL, 1 M) was added slowly. The resulting solution (pH ~ 7, according to pH paper) was extracted with MTBE (10x 30 mL) to remove the majority of TMP. The aqueous phase was concentrated under reduced pressure. The residue was dissolved in TEAB buffer (1 mL, 1 M) and purified by flash chromatography (C18-aq 150 g; eluent TEAB (0.1 M)/CH_3CN, gradient 0-50%) and freeze-dried to obtain 217 mg (yield: 45%) of compound **6a** as a fluffy lyophilizate as bis(triethylammonium) salt.

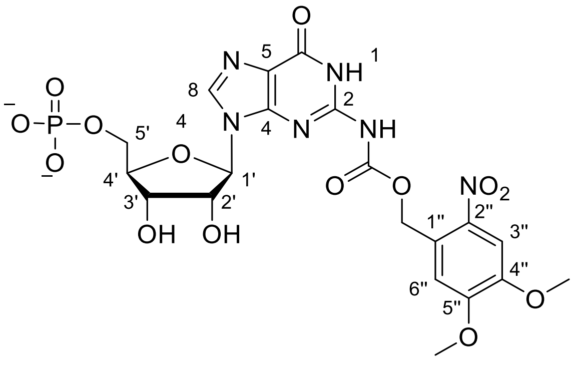

^{1}H NMR (500 MHz, D_2O): δ 8.19 [s, 1H, H-8], 7.07 [s, 1H, H-3′′], 6.94 [s, 1H, H-6′′], 5.76 [d, *J* = 4.3 Hz, 1H, H-1′], 5.16 [t, *J* = 9.7 Hz, 2H, $\underline{CH_2}$-Ar], 4.54 [t, *J* = 4.6 Hz, 1H, H-2′], 4.47 [t, *J* = 5.0 Hz, 1H, H-3′], 4.30 [q, *J* = 4.1 Hz, 1H, H-4′], 4.04 [ddt, *J* = 26.3, 11.3, 3.6 Hz, 2H, H-5′], 3.86 [s, 3H, $\underline{CH_3}$-5′′], 3.44 [s, 3H, $\underline{CH_3}$-4′′].

^{13}C NMR (126 MHz, D_2O): δ 156.90 [C-6 or C-2 or -NH-$\underline{CO}$-Oar], 156.90 [C-6 or C-2 or -NH-$\underline{CO}$-Oar], 153.34 [C-5′′], 149.76 [C-6 or C-2 or -NH-$\underline{CO}$-Oar], 149.41 [C-4], 146.55 [C-4′′], 138.38 [C-8], 137.07 [C-2′′], 126.75 [C-1′′], 117.97 [C-5], 107.76 [C-6′′], 107.03 [C-3′′], 87.61 [C-1′], 83.78 [C-2′], 74.72 [C-3′], 70.26 [C-4′], 65.22 [$\underline{CH_2}$-Ar], 63.48 [C-5′], 56.58 [O-$\underline{CH_3}$-5′′], 55.78[O-$\underline{CH_3}$-4′′].

HRMS (ESI-) m/z: calculated for $C_{20}H_{23}N_6O_{14}P_1$ $[M-H]^-$: 601.09371, found 601.09237.

***N*²-(4,5-Dimethoxy-2-nitrobenzyl)oxycarbonyl-7-methylguanosine 5′-monophosphate (7a)**

Bis(triethylammonium) salt of compound **6a** (392 mg, 487 µmol, 1 eq.) was dissolved in dry DMSO (3.5 mL) in a Schlenk flask (10 mL) under argon atmosphere. Iodomethane (605 µL, 1.38 g, 9.7 mmol, 20 eq.) was added and the reaction mixture was stirred for 4 hours. Then, the resulting solution was mixed with cooled TEAB (2 mL, 1 M). After five minutes, the mixture was purified by flash chromatography (C18-aq 150 g; eluent TEAB (0.1 M)/CH_3CN, gradient 0-50%). Fractions containing the pure product were pooled and freeze-dried to obtain 170 mg (yield: 43%) of compound **7a** as a fluffy lyophilizate.

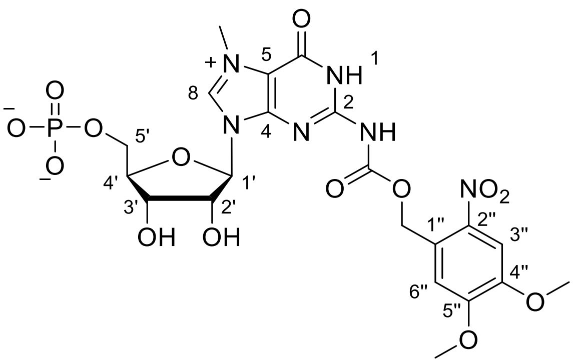

^{1}H NMR (600 MHz, D_2O): δ 7.44 (s, 1H, H-3′′), 6.90 (s, 1H, H-6′′), 5.88 (d, *J* = 3.8 Hz, 1H, H-1′), 5.19 (q, *J* = 15.3 Hz, 2H, -$\underline{CH_2}$-Ar), 4.54–4.50 (m, 1H, H-2′), 4.47 (t, *J* = 4.9 Hz, 1H, H-3′), 4.40–4.37 (m, 1H, H-4′), 4.19–4.14 (m, 1H, H-5′^{a}), 4.08 (s, 3H, 7-$\underline{CH_3}$), 4.07–4.02 (m, 1H, H-5′^{b}), 3.88 (s, 3H, 5′′-O-$\underline{CH_3}$), 3.78 (s, 3H, 4′′-O-$\underline{CH_3}$).

^{13}C NMR (151 MHz, D_2O): δ 153.35 [C-5′′], 153.03 [-NH-$\underline{CO}$-OCH_2Ar], 148.12 [C-4], 146.88 [C-4′′], 137.89 [C-2′′], 135.62 [C-8], 127.21 [C-1′′], 111.27 [C-5], 108.73 [C-6′′], 107.67 [C-3′′], 89.76 [C-1′], 84.92 [C-4′], 75.60 [C-2′], 69.69 [C-3′], 64.43 [-$\underline{CH_2}$-Ar], 62.92 [C-5′], 56.56 [5′′-$\underline{CH_3}$], 56.12 [4′′-$\underline{CH_3}$], 35.91 [7-$\underline{CH_3}$].

^{31}P NMR (243 MHz, D_2O): δ 2.59

HRMS (ESI+) m/z: calculated for $C_{21}H_{26}N_6O_{14}P_1^+$ $[M]^+$: 617.12391, found 617.12424.

P1-(N^2-(4,5-Dimethoxy-2-nitrobenzyl)oxycarbonyl-7-methylguanosin-5'-yl)-P3-guanosin-5'-yl triphosphate (1) (DMNB-cap)

$ZnCl_2$ (645 mg, 4.73 mmol, 43 eq.) was heated (170 °C) *in vacuo* for 3 hours in a Schlenk flask (10 mL) and then overlaid with argon and cooled to room temperature. Subsequently, the guanosine 5′-diphosphate derivative **8** (177 mg, 330 µmol, 3 eq.) was added. The bis(triethylammonium) salt of compound **7a** (90 mg, 110 µmol, 1 eq.) was dissolved in dry DMF (3 mL) under argon atmosphere and added to the reaction vessel. The resulting mixture was stirred 23 hours at room temperature. Subsequently, ddH_2O (1 mL) and an EDTA solution (1.5 mL, 0.5 M) were added. The reaction mixture was purified by flash chromatography (C18-aq 150 g; eluent TEAB (0.1 M)/CH_3CN, gradient 0-50%). The appropriate fractions were pooled, freeze-dried, dissolved in ddH_2O (2 mL) and purified on a POROS-50HQ column (eluent ddH_2O/TEAB (1 M), gradient 0-100%). Fractions containing the pure product were pooled

and freeze-dried to obtain 29.6 mg (yield: 24%) of compound **1** as a yellowish solid lyophilizate.

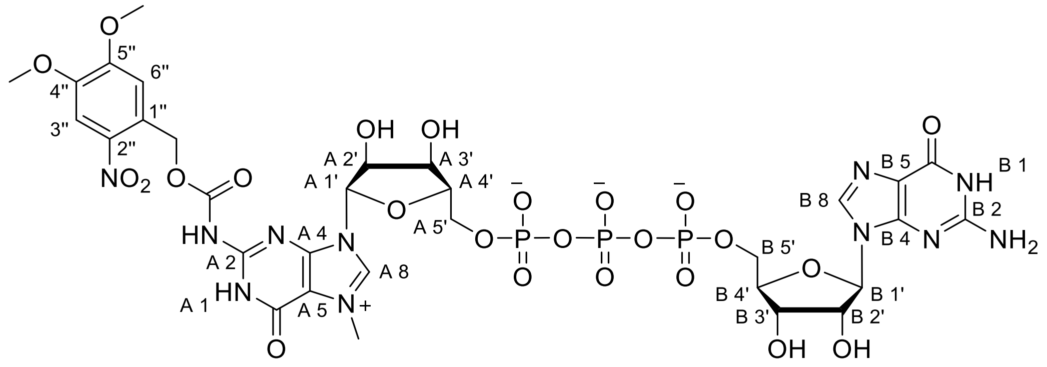

^{1}H NMR (600 MHz, D_2O): δ 7.91 [s, 1H, H-B8], 7.53 [s, 1H, H-6′′ or H-3′′], 7.06 [s, 1H, H-6′′ or H-3′′], 5.91 [s, 1H, H-A1′], 5.70 [d, *J* = 5.7 Hz, 1H, H-B1′], 5.27 [s, 2H, -<u>CH2</u>-Ar], 4.63 [s, 1H, H-A2′], 4.55 [s, 1H, H-B2′], 4.49 [s, 1H, H-A3′], 4.43 [d, *J* = 5.8 Hz, 2H, H-A4′ and H-B3′], 4.38 [d, *J* = 11.1 Hz, 1H, H-A5′ or H-B5′], 4.31 – 4.20 [m, 4H, H-A5′ and H-B5′ and H-B4′], 4.14 [s, 3H, -<u>CH3</u>], 3.90 [s, 3H, -<u>CH3</u>], 3.84 [s, 3H, -<u>CH3</u>].

^{13}C NMR (151 MHz, D_2O): δ 151.18 [C-B4], 136.74 [C-B8], 115.61 [C-B5], 109.02 [C-6′′ or C-3′′], 107.82 [C-6′′ or C-3′′], 90.71 [C-A1′], 86.76 [C-B1′], 84.52 [C-A4′], 83.45 [C-B4′], 74.99 [C-A2′], 73.97 [C-B2′], 70.24 [C-B3′], 69.61 [C-A3′], 65.51 [C-A5′ or C-B5′], 65.14 [-<u>CH2</u>-Ar], 64.57 [C-A5′ or C-B5′], 57.04 [-<u>CH3</u>], 56.19 [-<u>CH3</u>], 36.27 [-<u>CH3</u>].

^{31}P NMR (243 MHz, D_2O): δ -11.43 [dd, *J* = 34.7, 18.9 Hz, 2P], -22.85 [t, *J* = 19.2 Hz, 1P].

HRMS (ESI-) m/z: calculated for $C_{31}H_{39}N_{11}O_{24}P_3$ $[M-2H]^-$: 1040.12312, found 1040.12002.

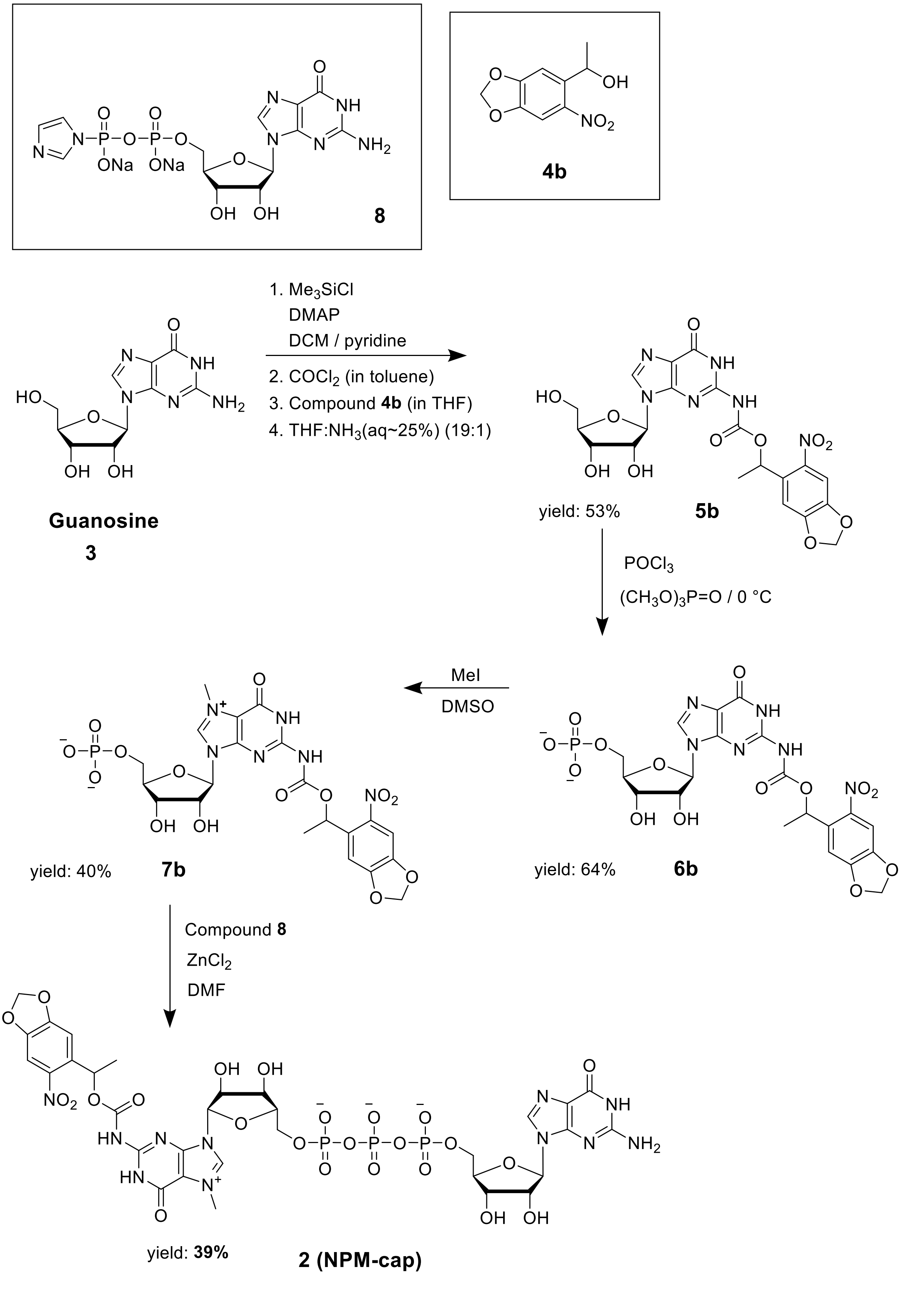
8
4b
1. Me3SiCl
DMAP
DCM / pyridine
2. COCl2 (in toluene)
3. Compound 4b (in THF)
4. THF:NH3(aq~25%) (19:1)
Guanosine
3
yield: 53%
5b
POCl3
(CH3O)3P=O / 0 °C
MeI
DMSO
yield: 64%
6b
yield: 40%
7b
Compound 8
ZnCl2
DMF
yield: 39%
2 (NPM-cap)

1-(6-Nitrobenzo[*d*][1,3]dioxol-5-yl)ethanol (4b)

Following literature procedure of Feng *et al.*[9]: 6-Nitropiperonal (**9**) (5.03 g, 25.8 mmol, 1 eq.) was dissolved in dry DCM (200 mL) under argon. The mixture was cooled by an ice-water bath for 10 minutes. A solution of Me_3Al (25 mL, 2 M in toluene) was added dropwise. The reaction mixture was stirred for 1.5 hours and tested by TLC (6:4 / cyclohexane:EtOAc). After full conversion of the starting material, the reaction was quenched by a solution of NaOH (200 mL, 1 M). The mixture was extracted with DCM (2x 150 mL)/water (1x 150 mL). The combined organic phases were washed with dH_2O (2x 200 mL). The organic solvent was evaporated under reduced pressure and the raw material was purified via flash chromatography (330 g SiO_2 column, gradient 80:20 cyclohexane:DCM to 55:40:5 cyclohexane:DCM:MeOH) to obtain the product **4b** as an orange-beige solid (4.36 g, 20.6 mmol, yield: 80%).

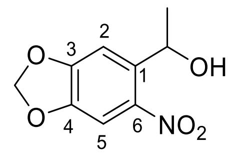

^{1}H NMR (500 MHz, DMSO-*d6*): δ 7.52 [s, 1H, H-5]; 7.27 [s, 1H, H-2]; 6.20 [dd, 2H, *J* = 13.2, 1.0 Hz, O-$\underline{CH_2}$-O]; 5.46 [d, 1H, *J* = 4.4 Hz, -OH]; 5.15 [qd, 1H, *J* = 6.3, 4.4 Hz, $\underline{CH}$-CH_3]; 1.34 [d, 3H, *J* = 6.3 Hz, CH-$\underline{CH_3}$].

^{13}C NMR (126 MHz, DMSO-*d6*): δ 151.82; 146.31; 140.64; 140.18; 106.04; 104.23; 103.12; 63.96; 26.32; 25.06.

HRMS (ESI+) m/z: calculated for $C_9H_9NO_5$ $[M+Na]^+$: 234.03729, found 234.03716.

***N*²-(1-(6-Nitrobenzo[*d*][1,3]dioxol-5-yl)ethyl)oxycarbonylguanosine (5b)**

Guanosine (**3**) (850 mg, 3 mmol, 1 eq.) and 4-dimethylaminopyridine (40 mg, 0.33 mmol, 0.1 eq.) were suspended in dry DCM (60 mL) in a round bottom schlenk flask under argon atmosphere. The mixture was

cooled by an ice-water bath and dry pyridine (10 mL) was added subsequently. Trimethylsilyl chloride (2.4 mL, 18.9 mmol, 6.3 eq.) was added dropwise and the reaction mixture was stirred for 2 hours. Phosgene in toluene (3 mL of 15wt% solution = roughly 4.2 mmol, 1.4 eq.) was added dropwise at 0 °C. The formation of a yellow precipitate was observed. The reaction mixture was stirred for further 30 minutes to suspend/dissolve the precipitate. Subsequently, a solution of 1-(4,5-methylenedioxy-2-nitrophenol) ethan-2-ol (**4b**) (2.04 g, 9.7 mmol, 3.2 eq.) in dry THF (20 mL) was added and the reaction mixture was stirred overnight to gradually warm up to room temperature. The reaction mixture was mixed with chloroform (100 mL) and demineralized water (100 mL). The organic layer was extracted with demineralized water (3x 100 mL) and the solvent was evaporated under reduced pressure. The obtained brown oily solid was dissolved in the THF (50 mL) and aqueous ammonia (2.5 mL of 25% solution) was added. The mixture was stirred overnight to form a dark-brown oily precipitate. The liquid was evaporated by a gentle stream of nitrogen applied via a long needle to the liquid (4 hours). The solid residue was dissolved in DMSO (35 mL), transferred onto silica gel (25 mL), freeze-dried and used as a solid load sample for flash chromatography (column: 25 µm-HC silica-gel 80 g; eluent $CHCl_3$/MeOH, gradient 5-30%). The solvent of the pure fraction was evaporated under reduced pressure to obtain compound **5b** as a white-beige solid (829 mg, 1.03 mmol, yield: 53%).

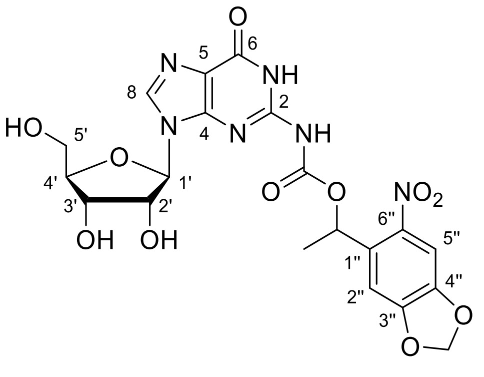

^{1}H NMR (600 MHz, DMSO-*d6*): δ 8.21 [d, 1H, *J* = 2.5 Hz, H-8]; 7.64 [s, 1H, H-5′′]; 7.24 [s, 1H, H-2′′]; 6.26–6.24 [m, 2H, O-$\underline{CH_2}$-O]; 6.25–6.22 [m, 1H,

CH-CH_3]; 5.77 [dd, 1H, *J* = 5.9, 1.3 Hz, H-1′]; 5.44 [bs, 1H, HO-C2′]; 5.15 [bs, 1H, HO-C3′]; 5.02–4.97 [m, 1H, HO-C5′]; 4.45 [dt, 1H, *J* = 14.2, 5.5 Hz, H-2′]; 4.13 [bs, 1H, H-3′]; 3.65–3.60 [m, 1H, H-5′[a]]; 3.56–3.51 [m, 1H, H-5′[b]]; 1.62 [d, 3H, *J* = 6.4 Hz, CH-CH_3]

^{13}C NMR (151 MHz, DMSO-*d6*): δ 155.07 [C-6]; 152.40 [-NH-CO-OCH_2Ar]; 152.39 [C-3″]; 149.05 [C-4]; 147.35 [C-4″]; 141.15 and 141.13 [C-6″]; 137.78 and 137.71 [C-8]; 133.68 and 133.65 [C-1″]; 119.92 and 119.89 [C-5]; 105.77 [C-2″]; 104.78 [C-5″]; 103.65 [O-CH_2-O]; 86.73 and 86.68 [C-1′]; 85.45 and 85.43 [C-4′]; 73.88 and 73.77 [C-2′]; 70.31 [C-3′]; 69.69 [CH-CH_3]; 61.29 and 61.28 [C-5′]; 21.56 [CH-CH_3].

HRMS (ESI+) m/z: calculated for $C_{20}H_{20}N_6O_{11}$ $[M+H]^+$: 521.12628, found 521.12627.

***N*²-(1-(6-Nitrobenzo[*d*][1,3]dioxol-5-yl)ethyl)oxycarbonylguanosine 5′-monophosphate (6b)**

The guanosine derivative **5b** (221 mg, 425 µmol, 1 eq.) was suspended in dry trimethyl phosphate (TMP) (4 mL) in a Schlenk flask (20 mL) under argon atmosphere. The solution was cooled to 0 °C and freshly distilled phosphoryl chloride (52 µL, 85 mg, 552 µmol, 1.3 eq.) was added slowly. The reaction mixture was stirred for 1 hour and a precooled solution of TEAB (6 mL, 1 M) was added slowly. The resulting solution (pH ~ 7, according to pH paper) was extracted with MTBE (10x 20 mL) to remove the majority of TMP. The aqueous phase was concentrated under reduced pressure. The residue was dissolved in TEAB buffer (1 mL, 1 M), purified by flash chromatography (C18-aq 150 g; eluent TEAB (0.1 M)/CH_3CN, gradient 0-50%) and freeze-dried to obtain 218 mg (yield: 64%) of compound **6b** as bis(triethylammonium) salt.

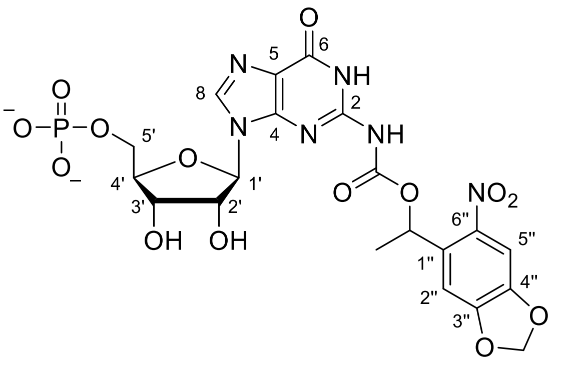

^{1}H NMR (500 MHz, D_2O): δ 8.31 (d, *J* = 5.9 Hz, 1H, H-8), 7.21 (d, *J* = 2.9 Hz, 1H, H-5″), 7.01 (s, 1H, H-2″), 6.19 (dq, *J* = 12.8, 6.3 Hz, 1H, <u>CH</u>-CH_3), 6.01 (dd, *J* = 16.3, 2.7 Hz, 2H, O-<u>CH_2</u>-O), 5.94 (dd, *J* = 14.7, 5.3 Hz, 1H, H-1′), 4.67 (td, *J* = 5.2, 3.2 Hz, 1H, H-2′), 4.47 (q, *J* = 3.8 Hz, 1H, H-3′), 4.33–4.21 (m, 1H, H-4′), 4.04 (h, *J* = 7.7, 7.2 Hz, 2H, H-5′), 1.54 (d, *J* = 6.4 Hz, 3H, CH-<u>CH_3</u>].

^{13}C NMR (126 MHz, D_2O): δ 153.89 [-NH-<u>CO</u>-OCH_2Ar], 152.77 [C-3″], 149.47 [C-4], 147.19 [C-4″], 140.48 [C-6″], 139.23 [C-8], 134.27 [C-1″], 119.09 [C-5], 105.74 [C-2″], 104.73 [C-5″], 103.90 [O-<u>CH_2</u>-O], 87.44[C-1′] 84.33 [C-4′], 74.72 [C-2′], 71.39 [<u>CH</u>-CH_3], 70.58 [C-3′], 63.80 [C-5′], 20.79 [CH-<u>CH_3</u>].

^{31}P NMR (202 MHz, D_2O): δ 2.88.

HRMS (ESI-) m/z: calculated for $C_{20}H_{21}N_6O_{14}P_1$ $[M-H]^-$: 599.07806, found 599.07705.

***N*²-(1-(6-Nitrobenzo[*d*][1,3]dioxol-5-yl)ethyl)oxycarbonyl-7-methylguanosine 5′-monophosphate (7b)**

The bis(triethylammonium) salt of compound **6b** (166 mg, 207 µmol, 1 eq.) was dissolved in dry DMSO (2 mL) in a Schlenk flask (10 mL) under argon atmosphere. Iodomethane (258 µL, 588 mg 4.14 mmol, 20 eq.) was added and the reaction mixture was stirred for 6 hours. Then, the resulting solution was mixed with a cooled TEAB buffer (1.5 mL, 1 M). After five minutes, the mixture was purified by flash chromatography (C18-aq 150 g; eluent TEAB

(0.1 M)/CH_3CN, gradient 0-50%). The pure fraction was freeze-dried to obtain 68 mg (yield: 40%) of compound **7b** as a yellowish lyophilizate.

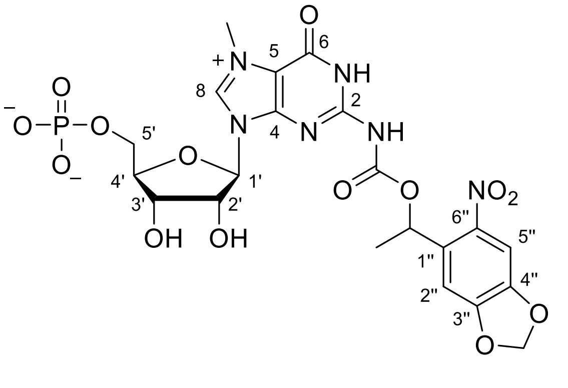

^{1}H NMR (500 MHz, D_2O): δ 7.44 [d, J = 3.6 Hz, 1H, H-5″], 7.10 [s, 1H, H-2″], 6.28–6.20 [m, 1H, CH-CH_3], 6.17 [d, J = 3.5 Hz, 1H, H-1′], 6.13 [ddd, J = 7.0, 4.1, 1.9 Hz, 2H, O-CH_2-O], 4.61 [ddd, J = 11.7, 4.9, 3.7 Hz, 1H, H-2′], 4.49 [dt, J = 8.1, 5.0 Hz, 1H, H-3′], 4.38 [td, J = 5.6, 2.5 Hz, 1H, H-4′], 4.18 [s, 3H, 7-CH_3], 4.17–4.12 [m, 1H, H-5′], 4.06–3.99 [m, 1H, H-5′], 1.61 [d, J = 6.4 Hz, 3H, CH-CH_3].

^{13}C NMR (126 MHz, D_2O): δ 153.32 [-NH-CO-OCH_2Ar], 152.90 [C-3″], 147.22 [C-4″], 140.81 [C-6″], 135.20 [C-1″], 111.94 [C-5], 105.82 [C-2″], 105.00 [C-5″], 103.72 [O-CH_2-O], 89.60 [C-1′], 84.77 [C-4′], 75.52 [C-2′], 70.35 [CH-CH_3], 69.47 [C-3′], 62.62 [C-5′], 36.00 [7-CH_3], 20.79 [CH-CH_3].

^{31}P NMR (202 MHz, D_2O): δ 3.34.

HRMS (ESI-) m/z: calculated for $C_{21}H_{24}N_6O_{14}P_1$ $[M-2H]^-$: 613.09371, found 613.09254.

P1-(N^2-(1-(6-Nitrobenzo[*d*][1,3]dioxol-5-yl)ethyl)oxycarbonyl-7-methylguanosin-5'-yl)-P3-guanosin-5'-yl triphosphate (2) (NPM-cap)

$ZnCl_2$ (487 mg, 3.57 mmol, 43 eq.) was heated (170 °C) *in vacuo* for 3 hours in a Schlenk flask (10 mL) and then overlaid with argon and cooled to room temperature. Subsequently, guanosine 5′-diphosphate derivative **8** (134 mg, 249 μmol, 3 eq.) was added. The bis(triethylammonium) salt of

compound **7b** (68 mg, 83 µmol, 1 eq.) was dissolved in dry DMF (2 mL) under argon atmosphere and added to the reaction vessel containing the other components. The resulting mixture was stirred for 32 hours at room temperature. Subsequently, ddH_2O (1 mL) and an EDTA solution (1.5 mL, 0.5 M) were added. The reaction mixture was purified by flash chromatography (C18-aq 150 g; eluent TEAB (0.1 M)/CH_3CN, gradient 0-50%). Fractions containing the product were pooled, freeze-dried, dissolved in ddH_2O (2 mL) and purified on a POROS-50HQ column (eluent ddH_2O/TEAB (1 M), gradient 0-100%). The product containing fractions were pooled and freeze-dried to obtain 43 mg (yield: 39%) of compound **2** as a yellowish solid lyophilizate.

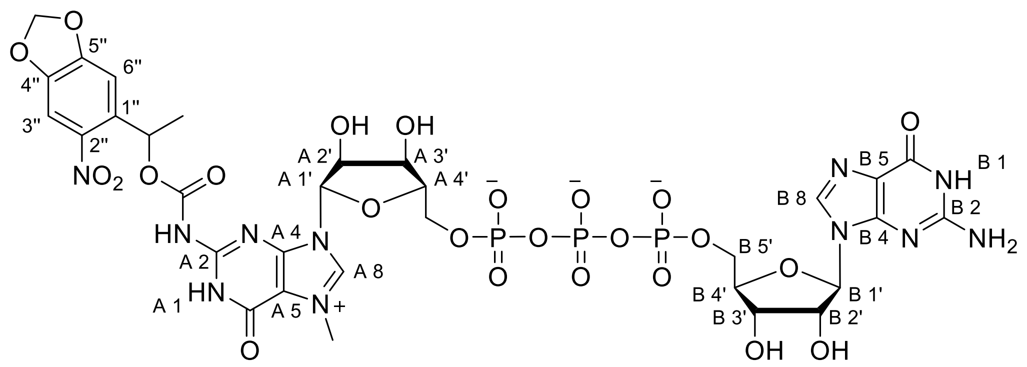

^{1}H NMR (600 MHz, D_2O): δ 7.99 [d, *J* = 2.9 Hz, 1H, H-B8], 7.42 [d, *J* = 10.0 Hz, 1H, H-3′′], 7.13 [d, *J* = 3.6 Hz, 1H, H-6′′], 6.26 – 6.21 [m, 1H, <u>CH</u>-CH_3], 6.11 [d, *J* = 7.4 Hz, 2H, O-<u>CH_2</u>-O], 6.04 [dd, *J* = 14.3, 3.4 Hz, 1H, H-A1′], 5.77 [dd, *J* = 5.9, 3.4 Hz, 1H, H-B1′], 4.62 [q, *J* = 5.5 Hz, 1H, H-B2′], 4.55 [dd, *J* = 10.7, 6.5 Hz, 1H, H-A2′], 4.49 – 4.42 [m, 2H, H-A3′ and H-B3′], 4.41 – 4.37 [m, 2H, H-A4′ and H-A5′], 4.33 – 4.22 [m, 4H, H-A5′ and H-B4′ and H-B5′], 4.12 [d, *J* = 2.8 Hz, 3H, 7-<u>CH_3</u>], 1.61 [dd, *J* = 6.6, 2.5 Hz, 3H, CH-<u>CH_3</u>].

^{13}C NMR (151 MHz, D_2O): δ 153.25 [-NH-<u>CO</u>-OCH_2Ar], 152.95 or 152.92 [C-5′′ or C-4′′], 151.43 [C-B4], 147.18 or 147.17 [C-5′′ or C-4′′], 140.61 [C-2′′], 137.11 [C-B8], 135.16 [C-1′′], 115.86 [C-B5], 111.64 [C-A5], 105.81 [C-6′′], 104.95 [C-3′′], 103.75[O-<u>CH_2</u>-O], 89.87 [C-A1′], 86.70 [C-B1′], 83.93 [C-A4′], 83.71 or 83.65 [C-B4′], 75.20 [C-A2′], 74.04 or 74.00

[C-B2′], 70.48 [CH-CH_3], 70.43 [C-B3′], 69.13 [C-A3′], 65.51 [C-A5′], 64.39 [C-B5′], 36.10 [7-CH_3], 20.92 [CH-CH_3].

^{31}P NMR (243 MHz, D_2O): δ -11.41 (t, J = 18.2 Hz, 2P), -22.85 (t, J = 19.1 Hz, 1P).

HRMS (ESI-) m/z: calculated for $C_{31}H_{37}N_{11}O_{24}P_3$ $[M-2H]^-$: 1038.10747, found 1038.10319.

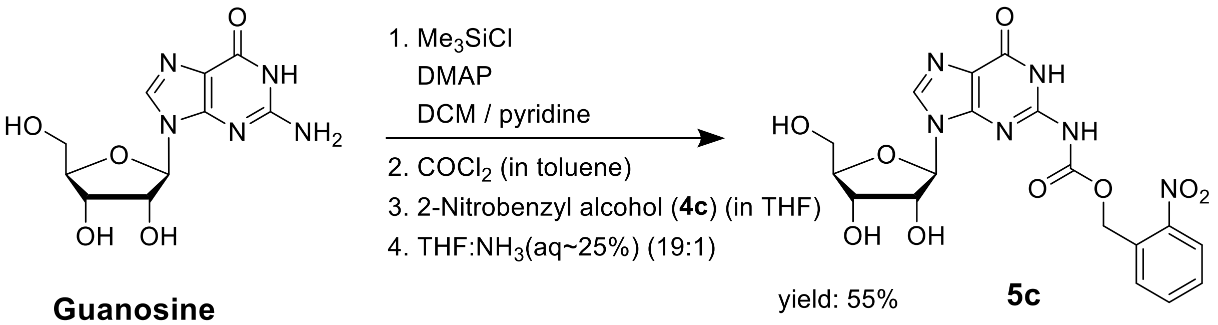

Guanosine
3

***N*²-(2-Nitrobenzyl)oxycarbonylguanosine (5c)**

Guanosine (**3**) (850 mg, 3 mmol, 1 eq.) and 4-dimethylaminopyridine (40 mg, 0.33 mmol, 0.1 eq.) were suspended in dry DCM (60 mL) in the round bottom schlenk flask under argon atmosphere. The mixture was cooled by an ice-water bath and the dry pyridine (10 mL) was added subsequently. Trimethylsilyl chloride (2.4 mL, 18.9 mmol, 6.3 eq.) was added dropwise and the reaction mixture was stirred for 2 hours. Phosgene in toluene (3 mL of 15wt% solution = roughly 4.2 mmol, 1.4 eq.) was added dropwise at 0 °C. The formation of a yellow precipitate was observed. The Reaction mixture was stirred for further 30 minutes. Subsequently, a solution of 2-nitrobenzyl alcohol (**4c**) (1.8 g, 11.8 mmol, 3.9 eq.) in dry THF (10 mL) was added and reaction mixture was stirred overnight to gradually warm up to room temperature. The reaction mixture was mixed with

chloroform (60 mL) and demineralized water (80 mL). The organic layer was extracted with demineralized water (3x 80 mL) and the solvent was evaporated under reduced pressure. The obtained brown oily solid was dissolved in the THF (50 mL) and aqueous ammonia (2.5 mL of 25% solution) was added. The mixture was stirred vigorously overnight to form a white-beige precipitate. The suspended precipitate was centrifuged (3220 rcf, 4 °C, 10 min), the supernatant was removed and the precipitate was freeze-dried to obtain compound **5c** as a white-beige solid (749 mg, 1.6 mmol, yield: 54%).

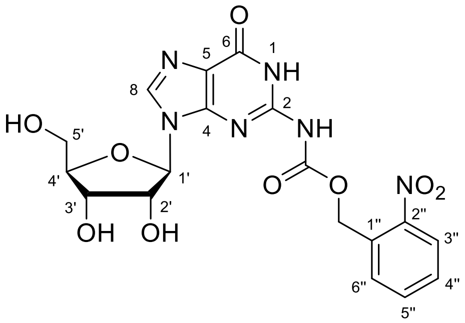

^{1}H NMR (500 MHz, DMSO-d_6): 8.14 [dd, 1H, J = 8.2, 1.2 Hz, H-3″]; 8.07 [s, 1H, H-8]; 7.83-7.86 [m, 2H, H-4″, H-6″]; 7.61 [ddd, 1H, J = 8.2, 7.0, 1,8 Hz, H-5″]; 5.75 [d, 1H, J = 6.1 Hz, H-1′]; 5.51 [d, 2H, J = 2.2 Hz, -<u>CH₂</u>-Ar]; 4.50 [dd, 1H, J = 6.1, 5.0 Hz, H-2′]; 4.12 [dd, 1H, J = 5.0, 3.2 Hz, H-3′] ; 3.91 [q, 1H, J = 3.6 Hz, H-4′]; 3.63 [dd, 1H, J = 12.1, 3.8 Hz, H-5′^{a}]; 3.53 [dd, 1H, J = 12.1, 3.8 Hz, H-5′^{b}]

^{13}C NMR (126 MHz, DMSO-d_6): 157.58 [-NH-CO-OCH$_2$Ar]; 156.12 [C-6]; 151.59 [C-2]; 150.15 [C-4]; 147.06 [C-2″]; 137.32 [C-8]; 134.21 [C-4″]; 132.71 [C-1″]; 128.97 and 128.95 [C-6″, C-5″]; 124.78 [C-3″]; 119.24 [C-5]; 87.20 [C-1′]; 85.67 [C-4′]; 73.63 [C-2′]; 70.54 [C-3′]; 63.11 [-CH$_2$-Ar]; 61.56 [C-5′]

HRMS (ESI+) m/z: calculated for $C_{18}H_{18}N_6O_9$ $[M+Na]^+$: 485.10275, found 485.1029.

6-Nitrobenzo[*d*][1,3]dioxol-5-yl-methanol (4d)

Following literature procedure of Saunthwal *et al.*[10]: 6-Nitropiperonal (**9**) (3.2 g, 16.4 mmol, 1 eq.) was dissolved in THF (80 mL) and ddH_2O (4 mL), sodium tetrahydridoborate (560 mg, 14.8 mmol, 0.9 eq.) was added and the reaction mixture was stirred at room temperature for 1 h. After extraction with ethyl acetate (3×150 mL) the solvent was removed under reduced pressure and the residue was purified by flash chromatography (cyclohexane:ethyl acetate 80:20 to 50:50). The product was obtained as yellow solid (3.16 g, 16.0 mmol, 98 %).

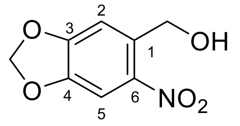

^{1}H NMR (500 MHz, DMSO-d_6): δ 7.65 [s, 1H, H-5], 7.29 [d, J = 0.9 Hz, 1H, H-2], 6.22 [s, 2H, O-<u>CH₂</u>-O], 5.56 [t, J = 5.4 Hz, 1H, O<u>H</u>], 4.77 – 4.75 [m, 2H, <u>CH₂</u>-OH].

^{13}C NMR (126 MHz, DMSO-d_6): δ 152.35, 146.29, 140.05, 136.88, 106.58, 104.87, 103.26, 60.21.

HRMS (ESI+) m/z: calculated for $C_7H_8NO_5$ $[M+Na]^+$: 220.02164, found 220.02141.

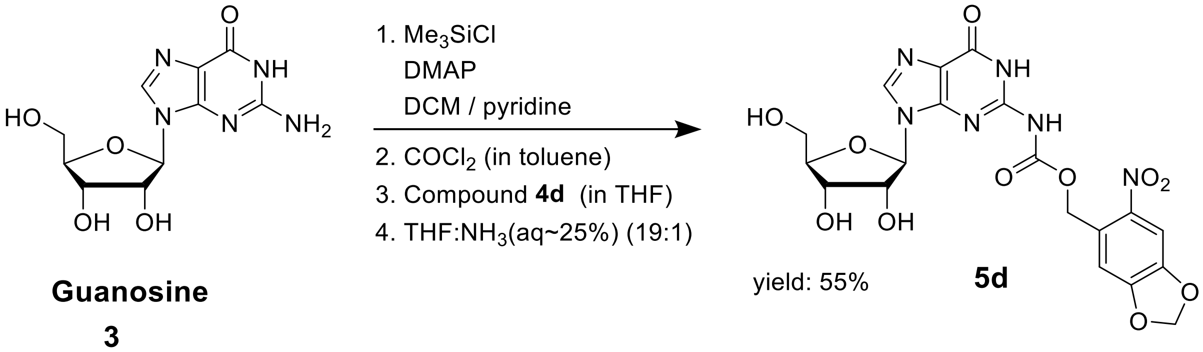

Guanosine
3

5d

***N*²-(6-nitrobenzo[*d*][1,3]dioxol-5-yl)methyl)oxycarbonylguanosine (5d)**

Guanosine (**3**) (850 mg, 3 mmol, 1 eq.) and 4-dimethylaminopyridine (40 mg, 0.33 mmol, 0.1 eq.) were suspended in dry DCM (60 mL) in a round bottom schlenk flask under argon atmosphere. The mixture was cooled by an ice-water bath and the dry pyridine (10 mL) was added subsequently. Trimethylsilyl chloride (2.4 mL, 18.9 mmol, 6.3 eq.) was added dropwise and the reaction mixture was stirred for 2 hours. Phosgene in toluene (3 mL of 15wt% solution = roughly 4.2 mmol, 1.4 eq.) was added dropwise at 0 °C. The formation of a yellow precipitate was observed. The Reaction mixture was stirred for further 30 minutes. Subsequently, a solution of **4d** (2.33 g, 11.8 mmol, 3.9 eq.) in dry THF (10 mL) was added and reaction mixture was stirred overnight to gradually warm up to room temperature. The reaction mixture was mixed with chloroform (60 mL) and demineralized water (80 mL). The organic layer was extracted with demineralized water (3x 80 mL) and the solvent was evaporated under reduced pressure. The obtained brown oily solid was dissolved in the THF (50 mL) and aqueous ammonia (2.5 mL of 25% solution) was added. The mixture was stirred vigorously overnight to form a white-beige precipitate. The suspended precipitate was centrifuged (3220 rcf, 4 °C, 10 min), the supernatant was removed and the precipitate was freeze-dried to obtain compound **5d** as a white-beige solid (840 mg, 1.66 mmol, yield: 55%).

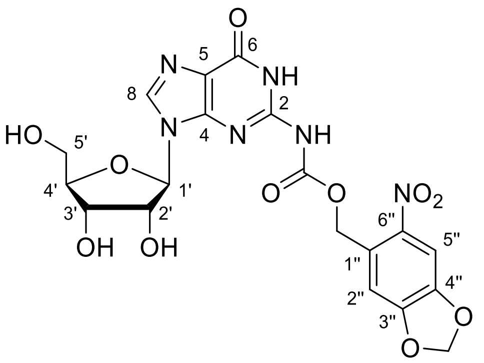

^{1}H NMR (500 MHz, DMSO-d_6): δ 8.24 [s, 1H, H-8]; 7.77 [s, 1H, H-5″]; 7.38 [s, 1H, H-2″]; 6.28 [s, 2H, O-CH_2-O]; 5.79 [d, 1H, *J* = 5.8 Hz, H-1′]; 5.53 [s, 2H, O-$\underline{CH_2}$-Ar]; 5.49 – 5.42 [m, 1H, -OH-2′]; 5.16 [d, 1H, *J* = 4.6 Hz, -OH-3′]; 5.00 [t, 1H, *J* = 5.4 Hz,-OH-5′]; 4.46 [d, 1H, *J* = 5.1 Hz, H-2′];

4.13 [d, 1H, *J* = 4.2 Hz, H-3′]; 3.90 [q, 1H, *J* = 4.0 Hz, H-4′]; 3.64 [dt, 1H, *J* = 11.8, 4.8 Hz, H-5′[a]]; 3.58 - 3.51 [m, 1H, H-5′[b]].

^{13}C NMR (126 MHz, DMSO-d_6): δ 155.08 [C-6]; 154.01 [-NH-<u>CO</u>-OCH$_2$Ar]; 152.55 [C-3″]; 149.08 [C-4]; 147.37 [C-4″]; 147.25 [C-2″]; 140.85 [C-6″]; 137.72 [C-8]; 128.79 [C-1″]; 119.91 [C-5]; 107.58 [C-2″]; 105.39 [C-5″]; 103.73 [O-CH$_2$-O]; 86.65 [C-1′]; 85.41 [C-4′]; 73.88 [C-2′]; 70.29 [C-3′]; 64.28 [O-<u>CH$_2$</u>-Ar]; 61.24 [C-5′].

HRMS (ESI+) m/z: calculated for $C_{19}H_{18}N_6O_{11}$ $[M+Na]^+$: 529.09258 found 529.09266

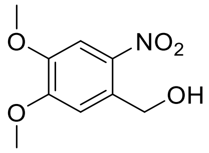

4a

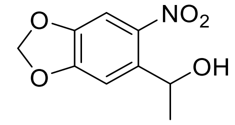

4b

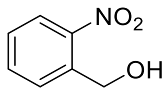

4c

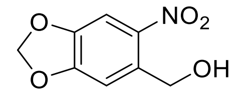

4d

Supplementary Figure 22. o-Nitrobenzyl alcohol derivatives used in this study.

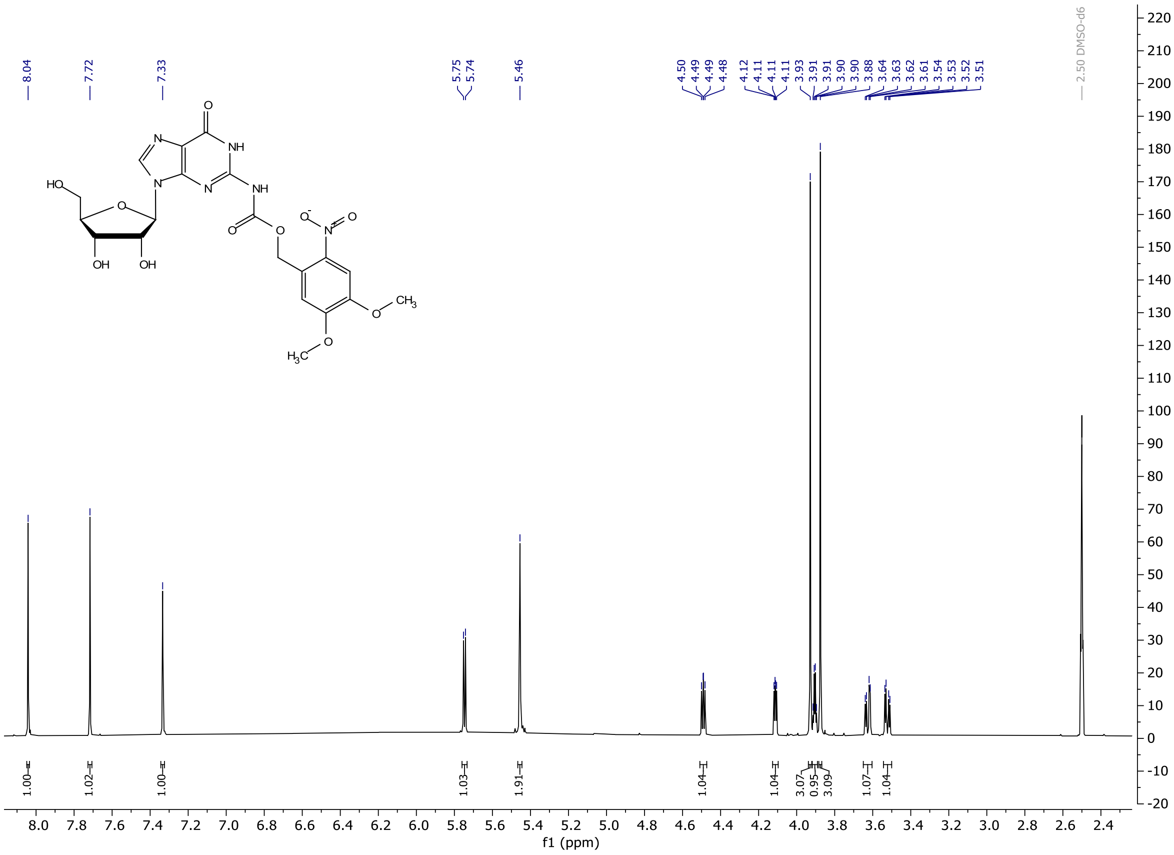

Supplementary Figure 23. ^{1}H-NMR spectrum of compound 5a.

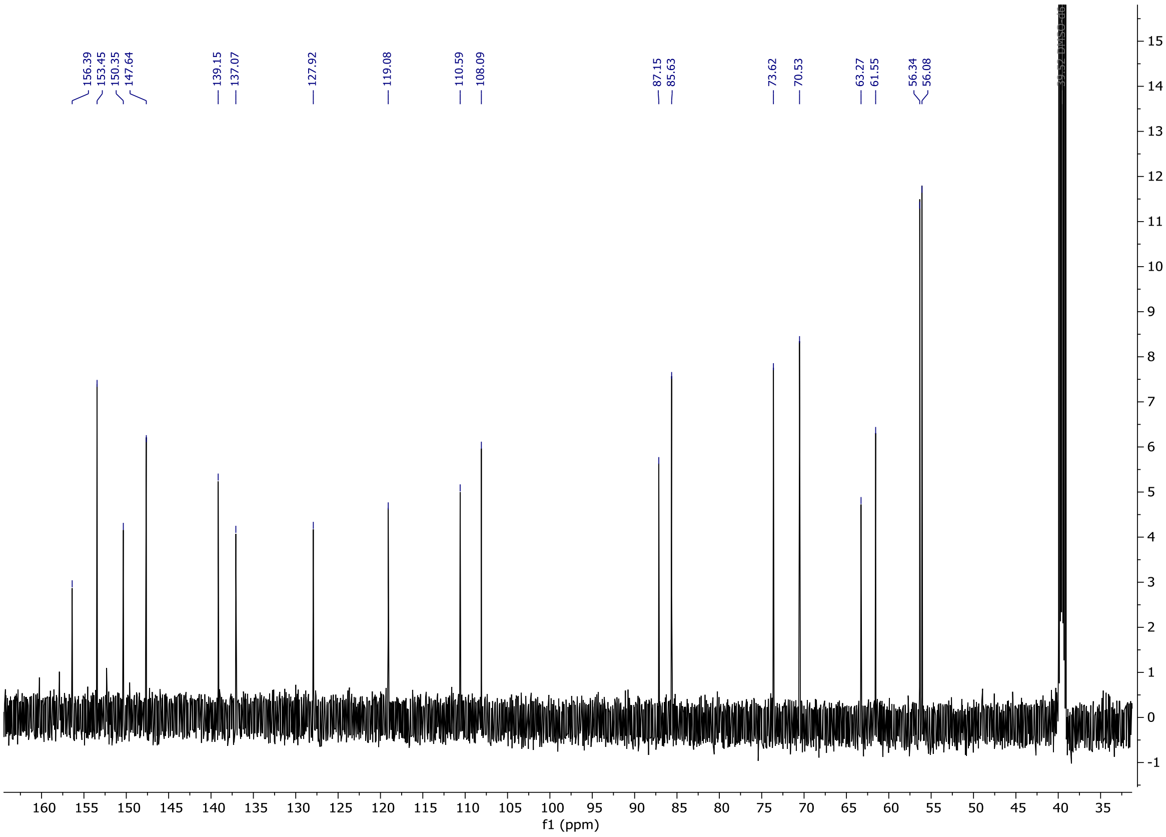

Supplementary Figure 24. ^{13}C-NMR spectrum of compound 5a.

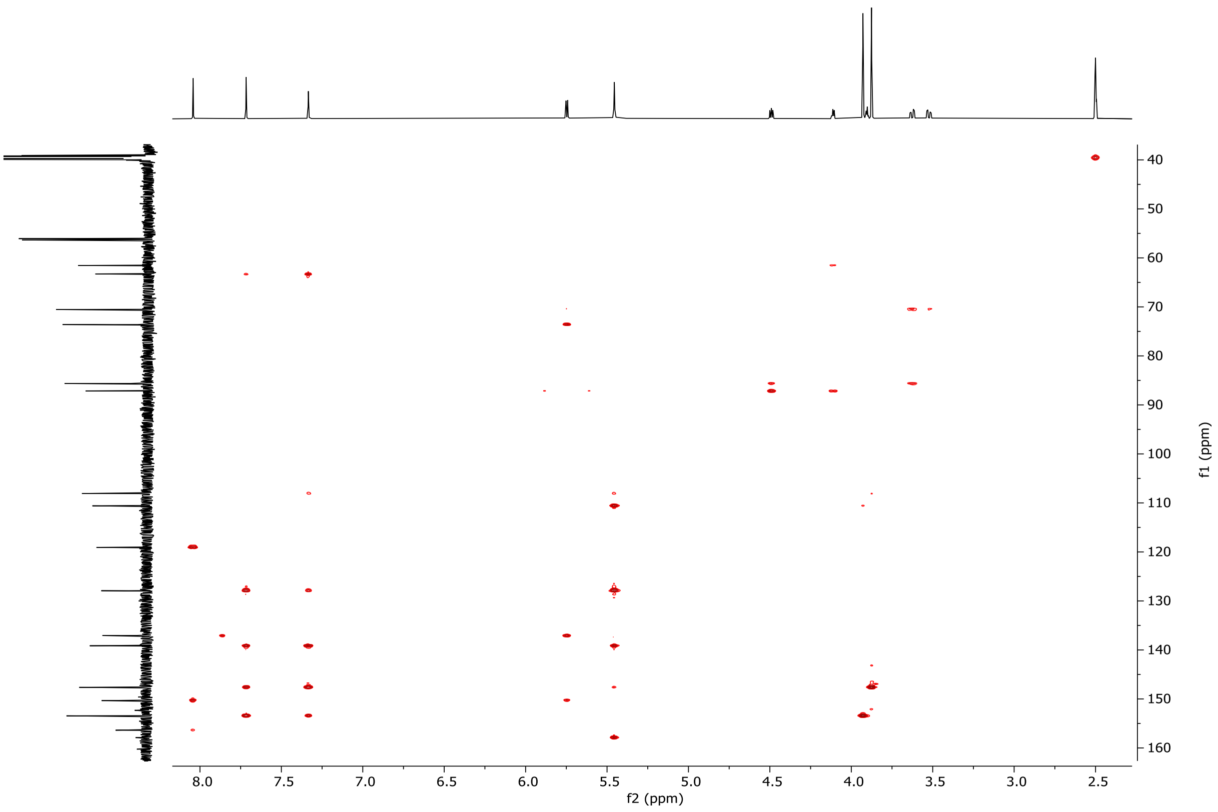

Supplementary Figure 25. HMBC spectrum of compound 5a.

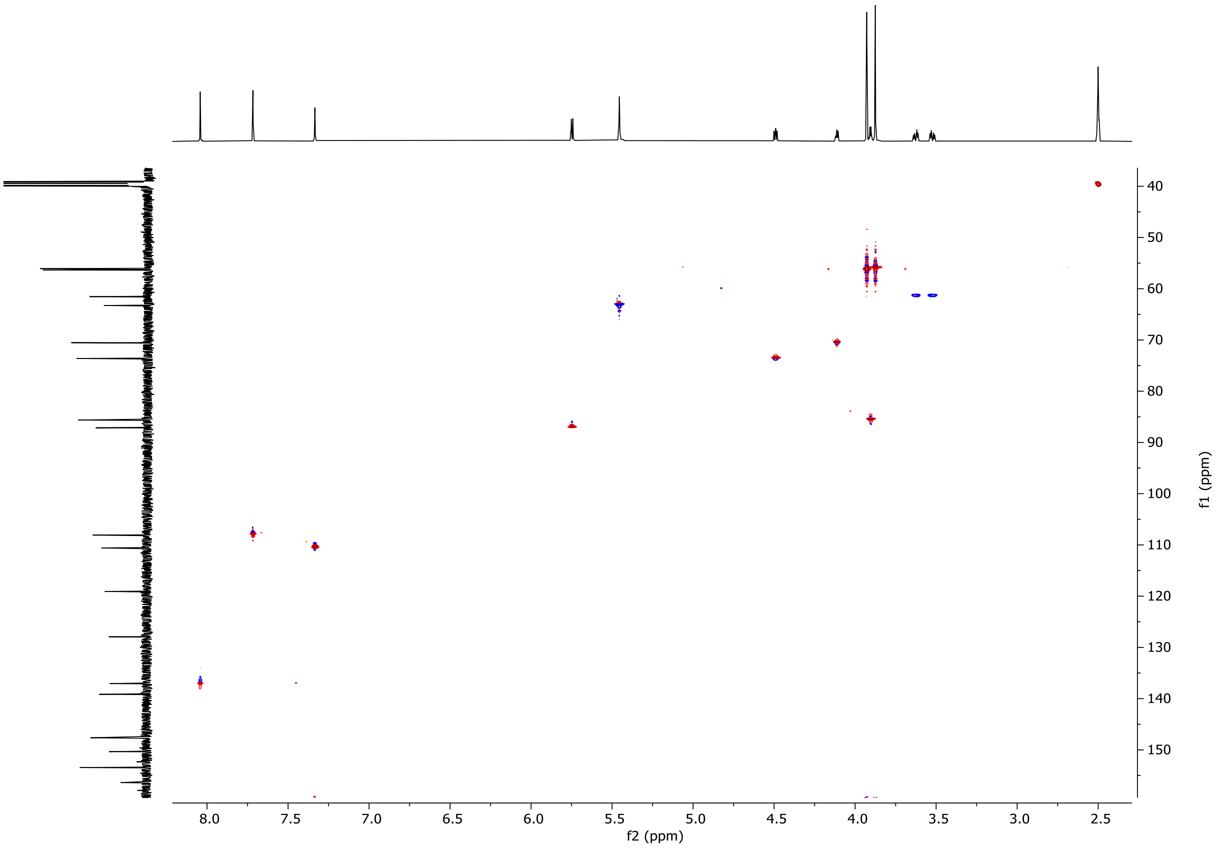

Supplementary Figure 26. HSQC spectrum of compound 5a.

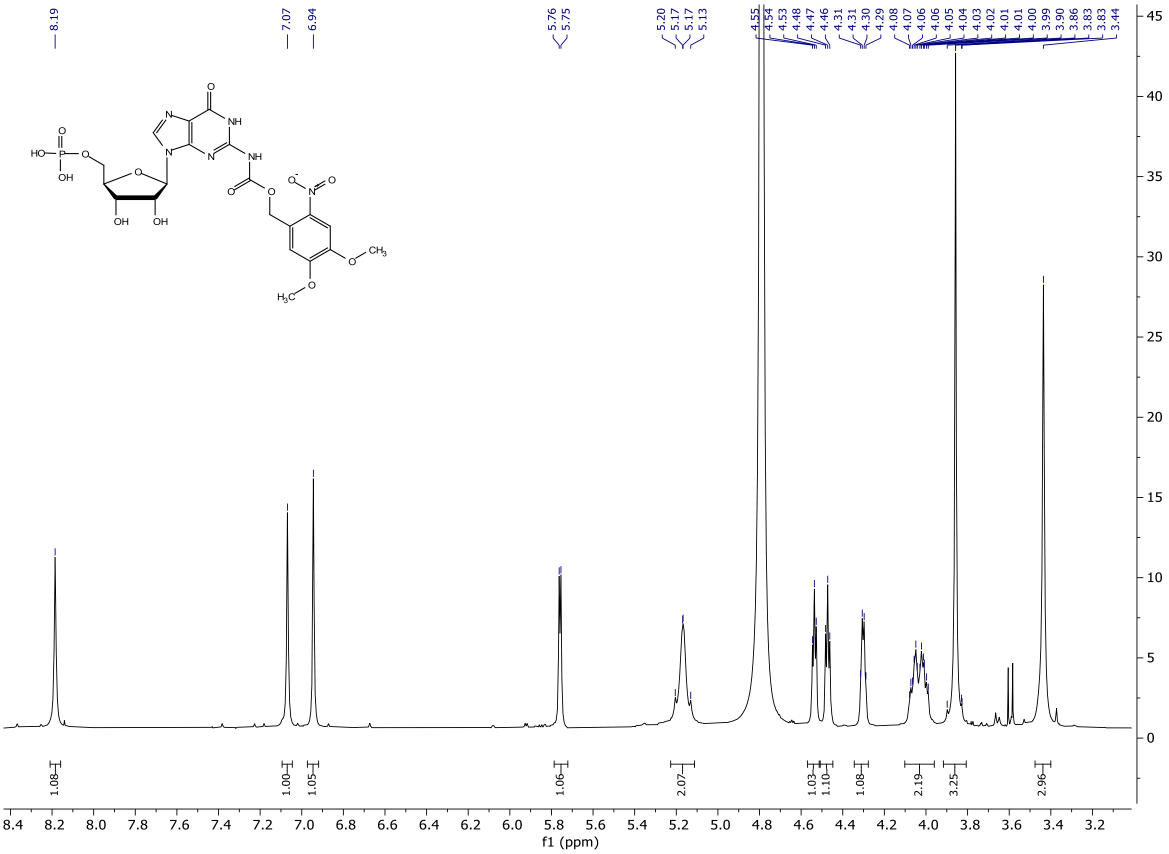

Supplementary Figure 27. ^{1}H-NMR spectrum of compound 6a.

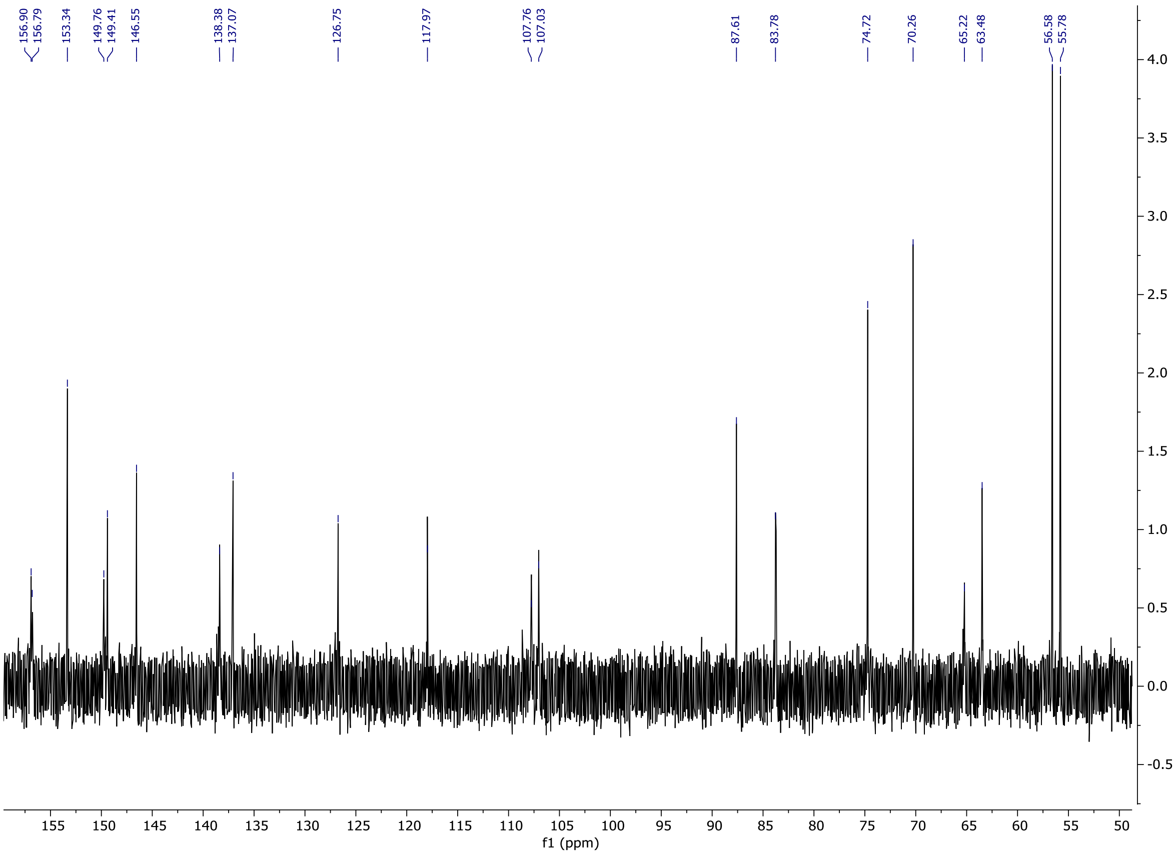

Supplementary Figure 28. ^{13}C-NMR spectrum of compound 6a.

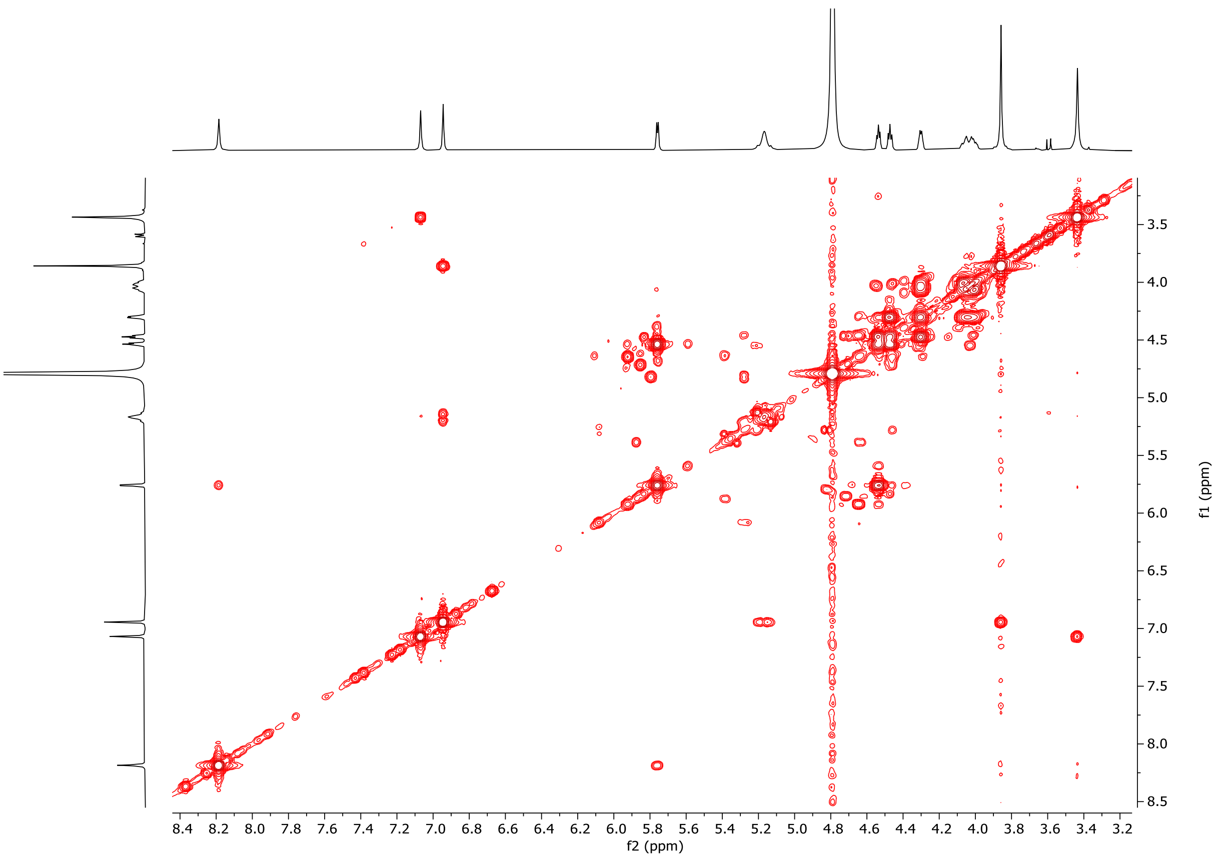

Supplementary Figure 29. COSY spectrum of compound 6a.

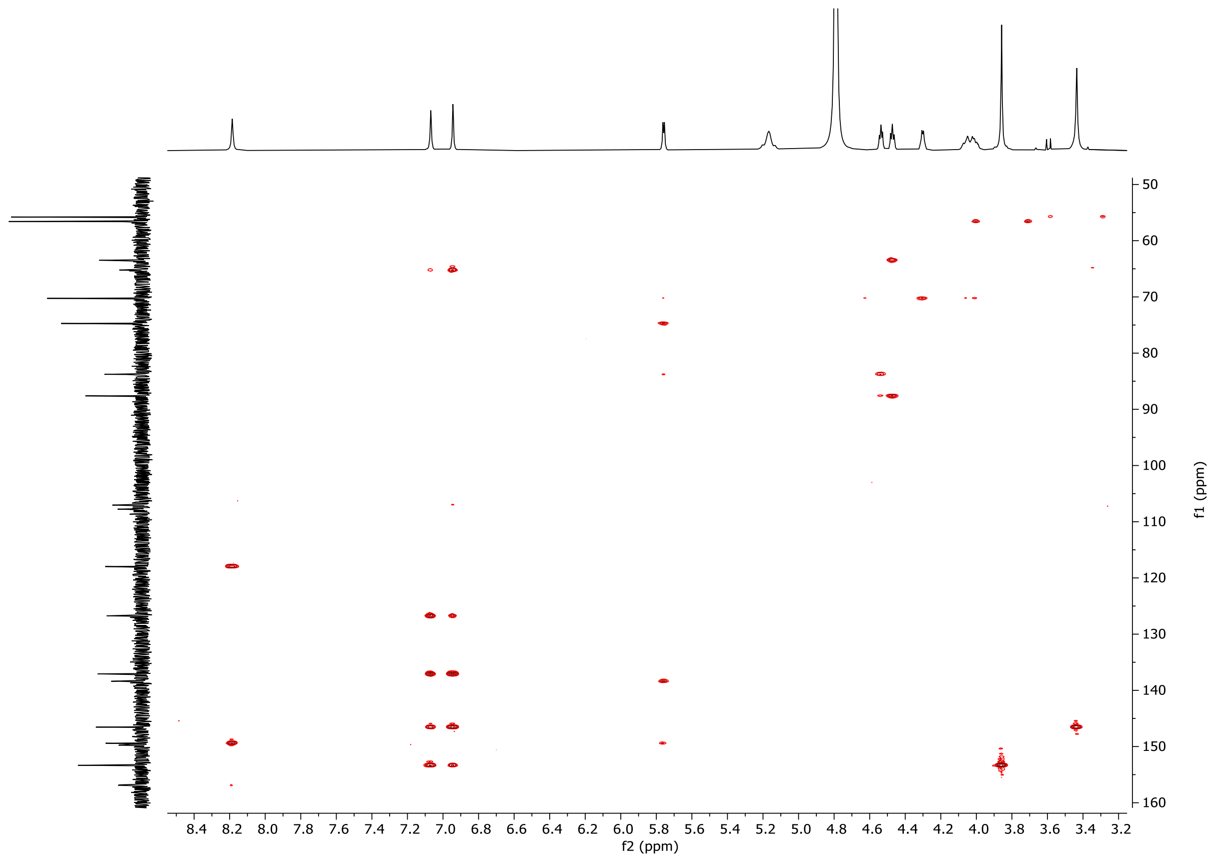

Supplementary Figure 30. HMBC spectrum of compound 6a.

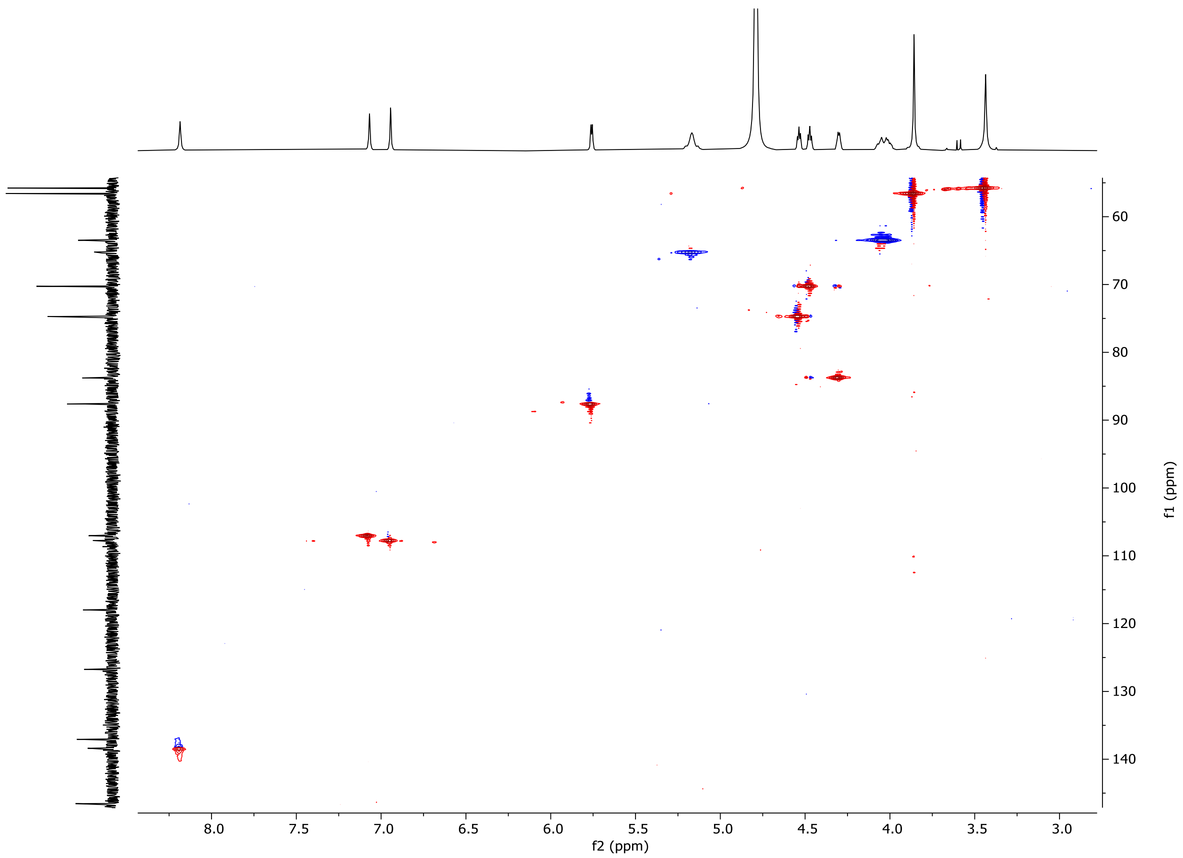

Supplementary Figure 31. HSQC spectrum of compound 6a.

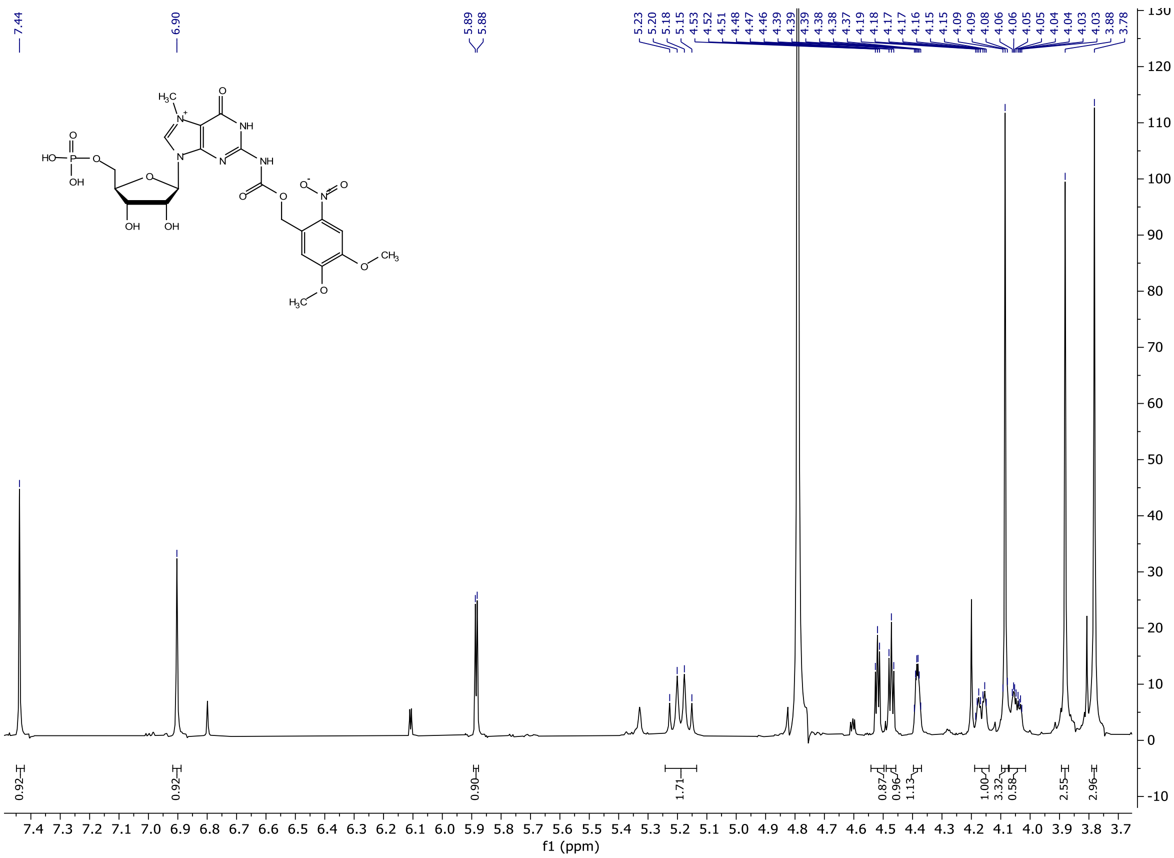

Supplementary Figure 32. ^{1}H-NMR spectrum of compound 7a.

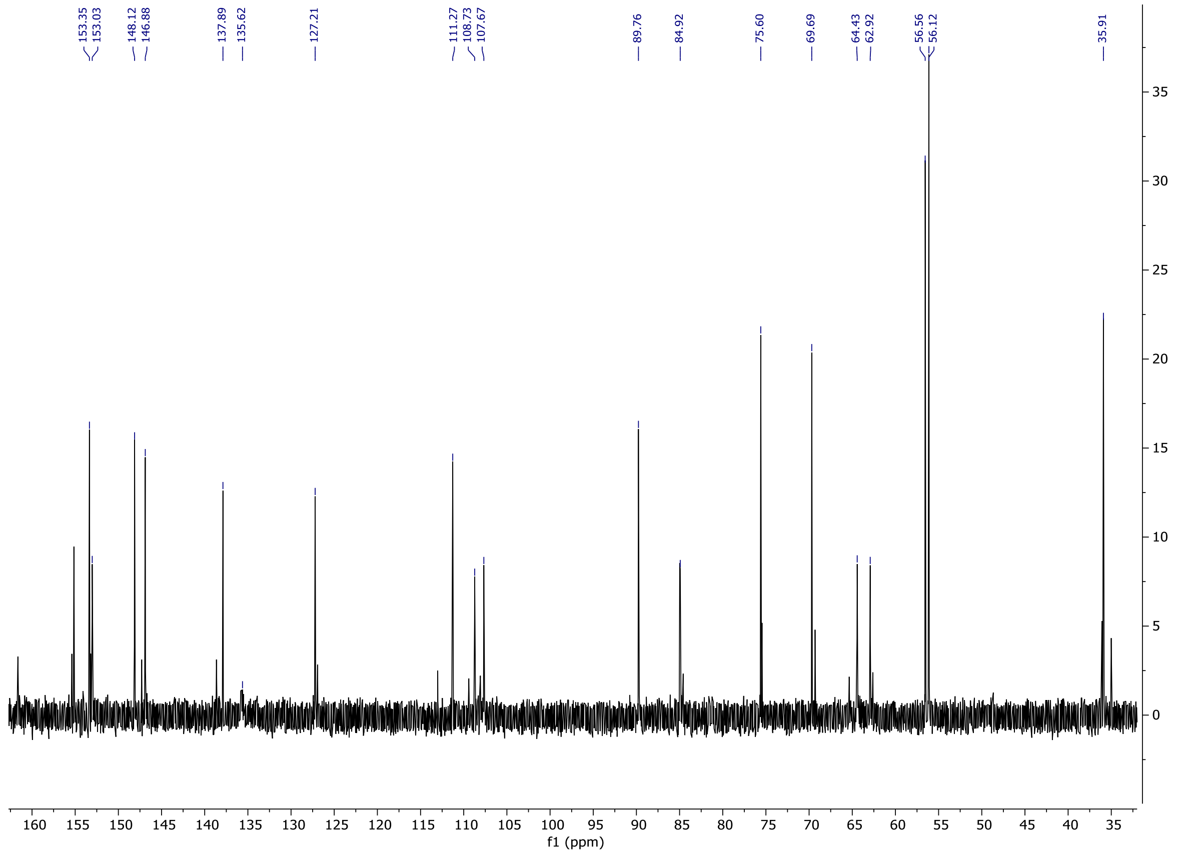

Supplementary Figure 33. ^{13}C-NMR spectrum of compound 7a.

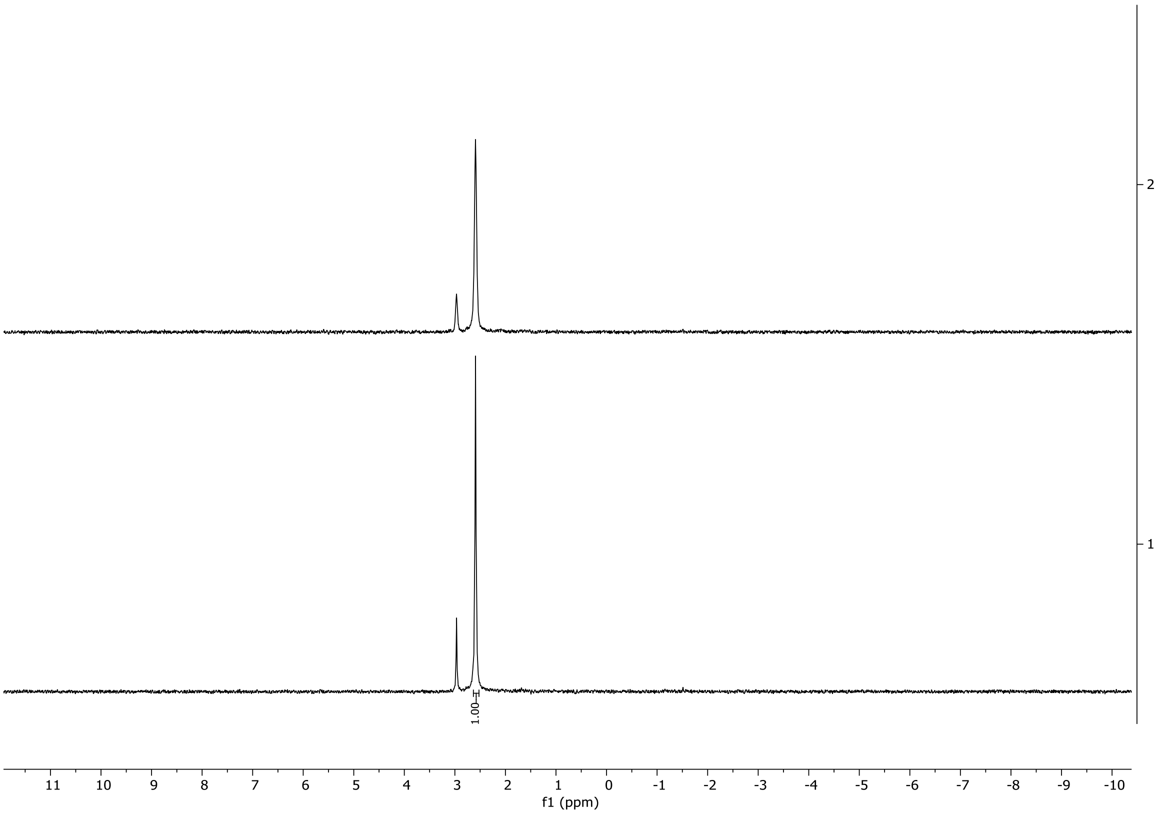

Supplementary Figure 34. ^{31}P-NMR spectrum of compound 7a.

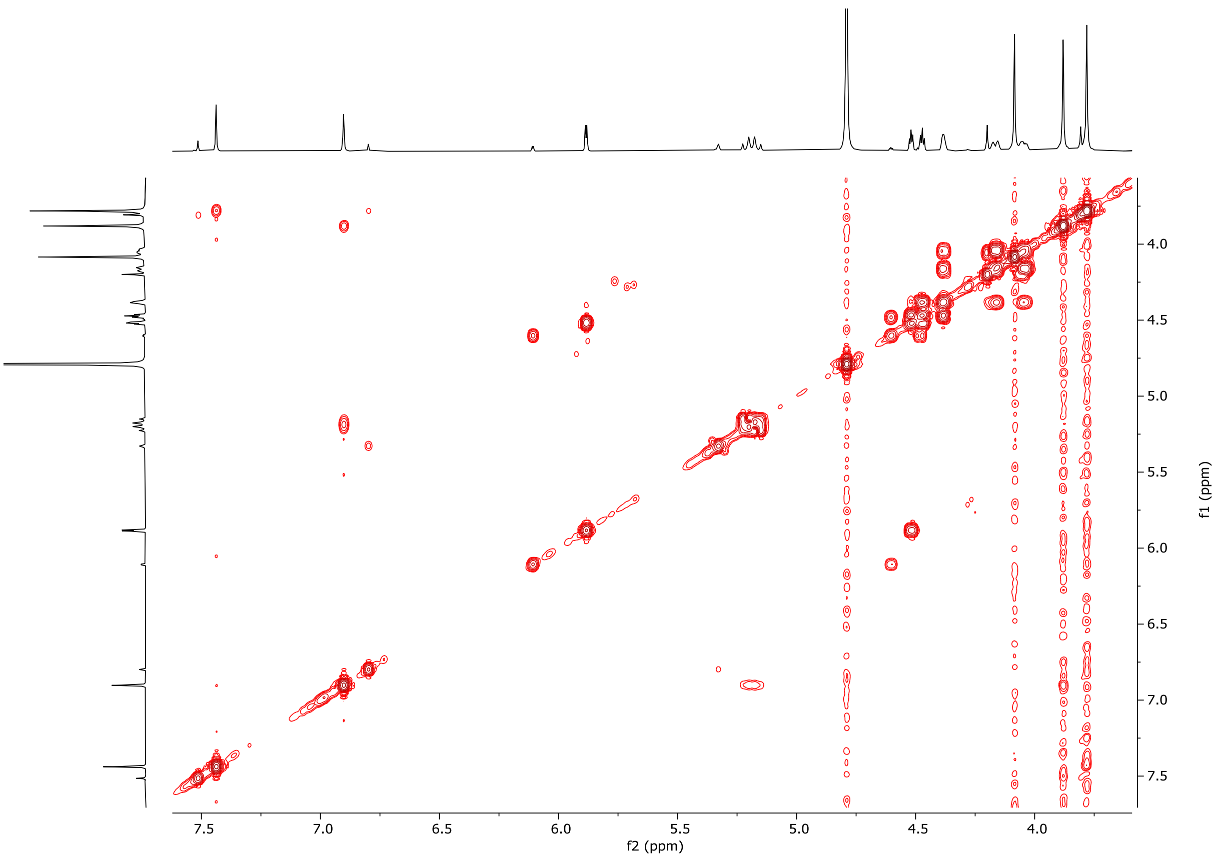

Supplementary Figure 35. COSY spectrum of compound 7a.

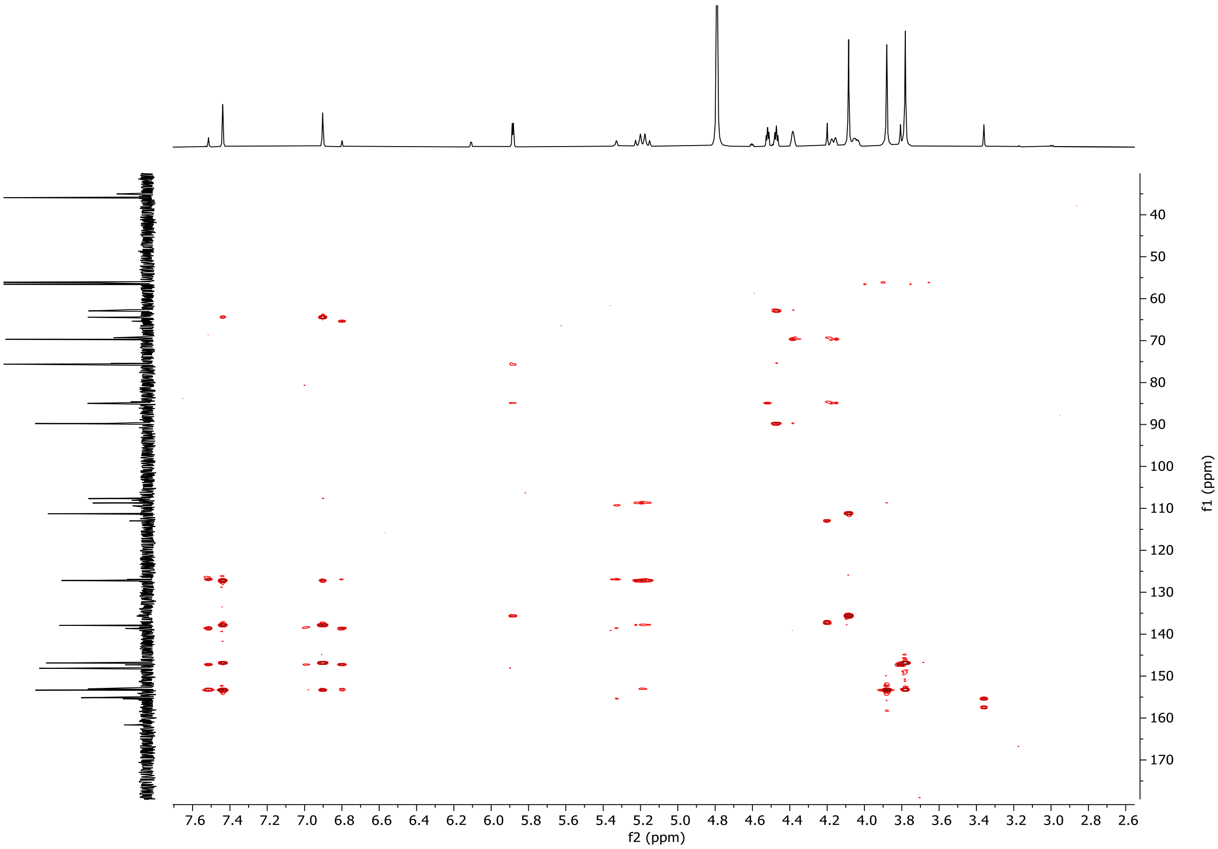

Supplementary Figure 36. HMBC spectrum of compound 7a.

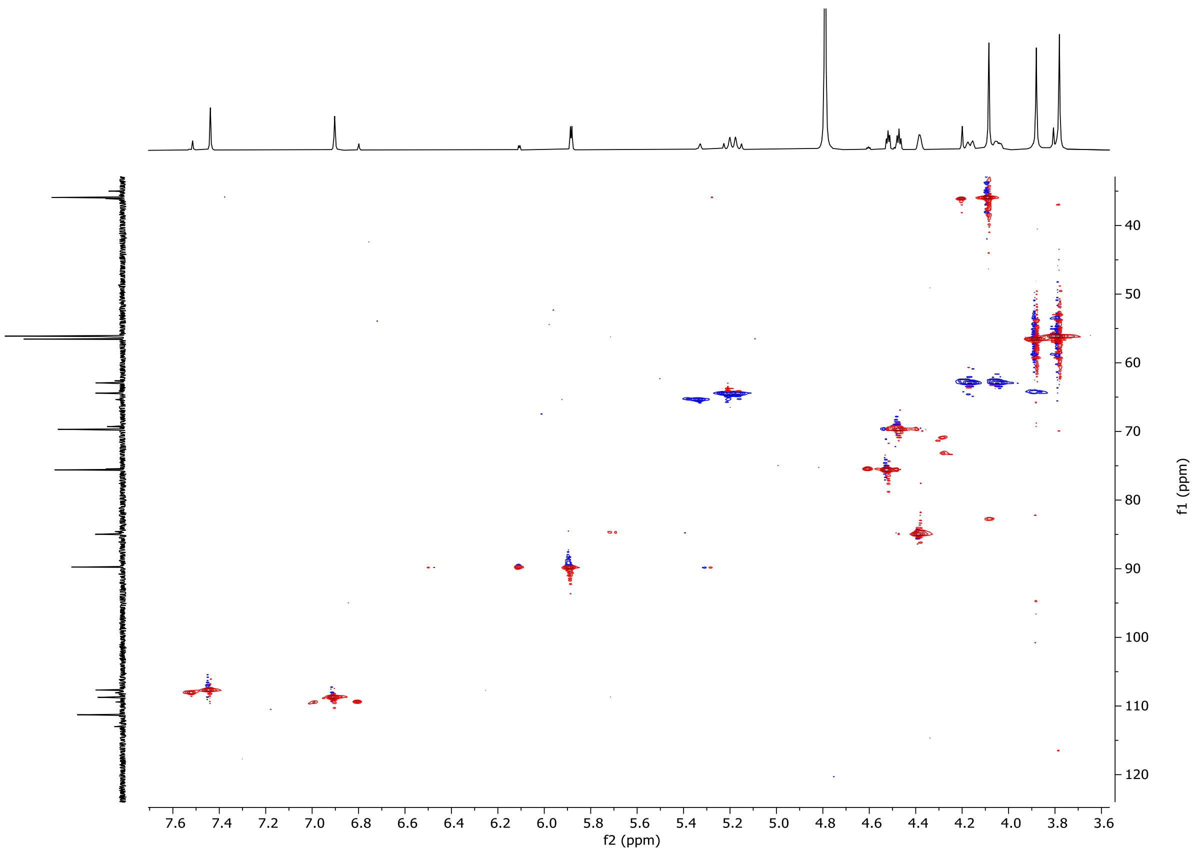

Supplementary Figure 37. HSQC spectrum of compound 7a.

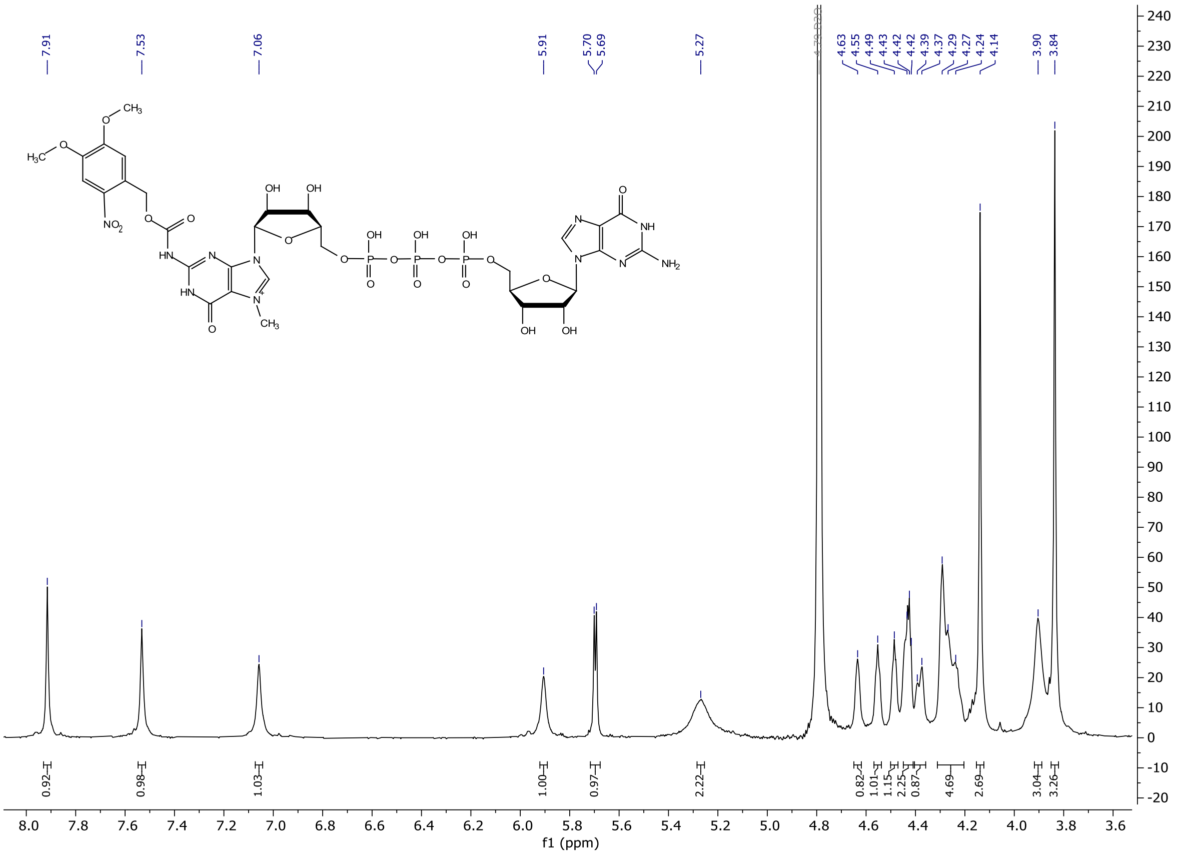

Supplementary Figure 38. ^{1}H-NMR spectrum of compound 1.

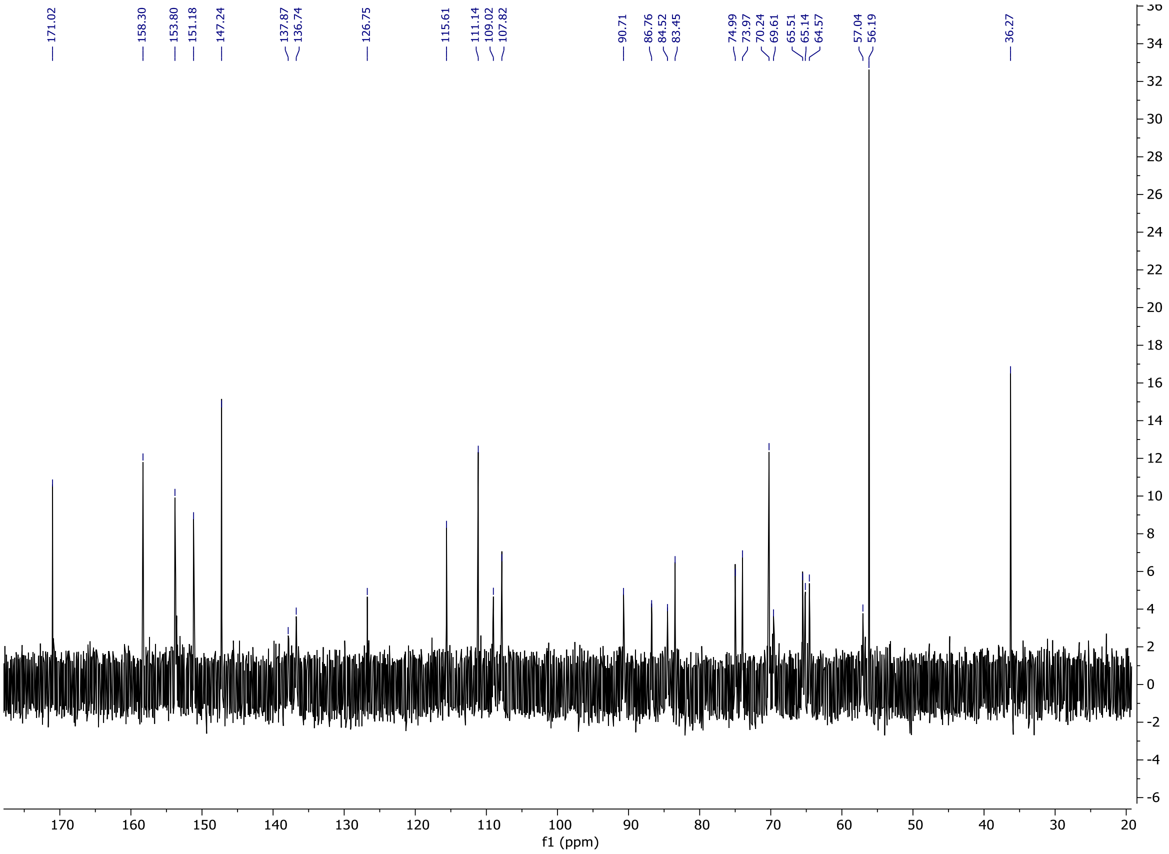

Supplementary Figure 39. ^{13}C-NMR spectrum of compound 1.

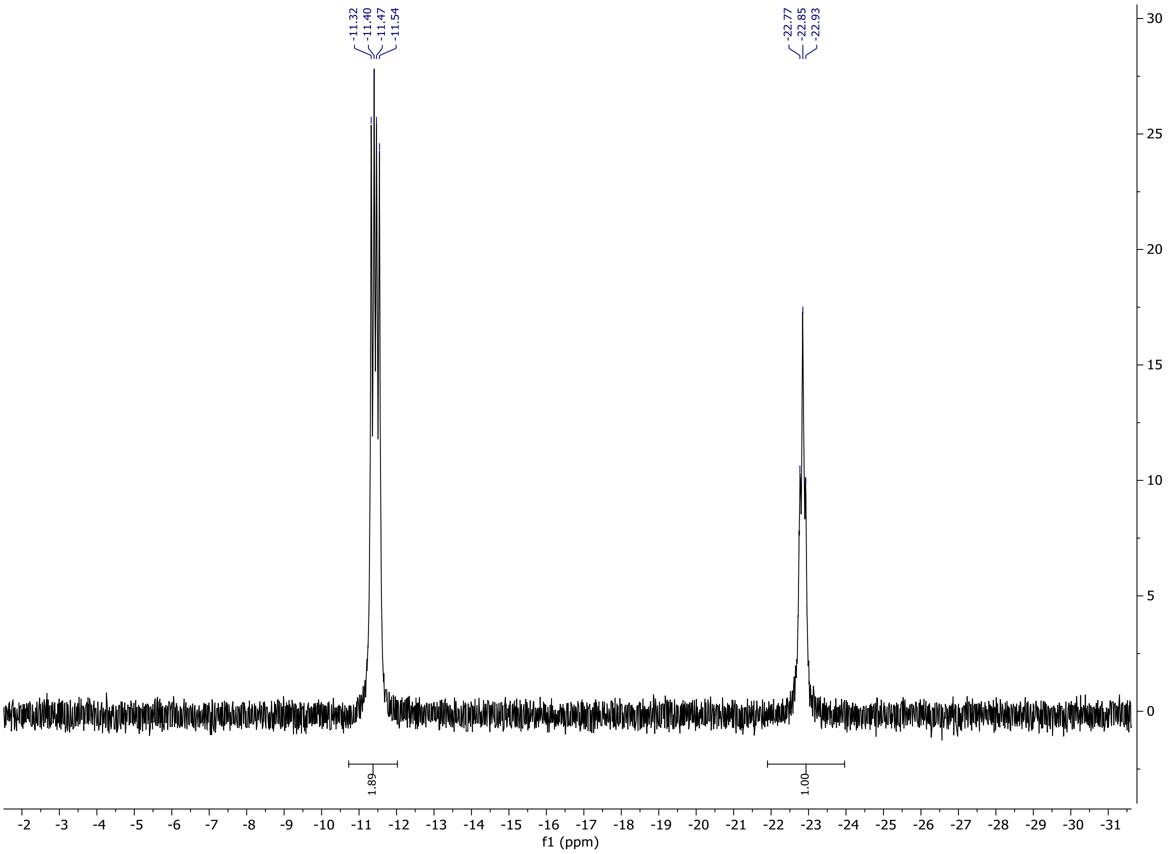

Supplementary Figure 40. ^{31}P-NMR spectrum of compound 1.

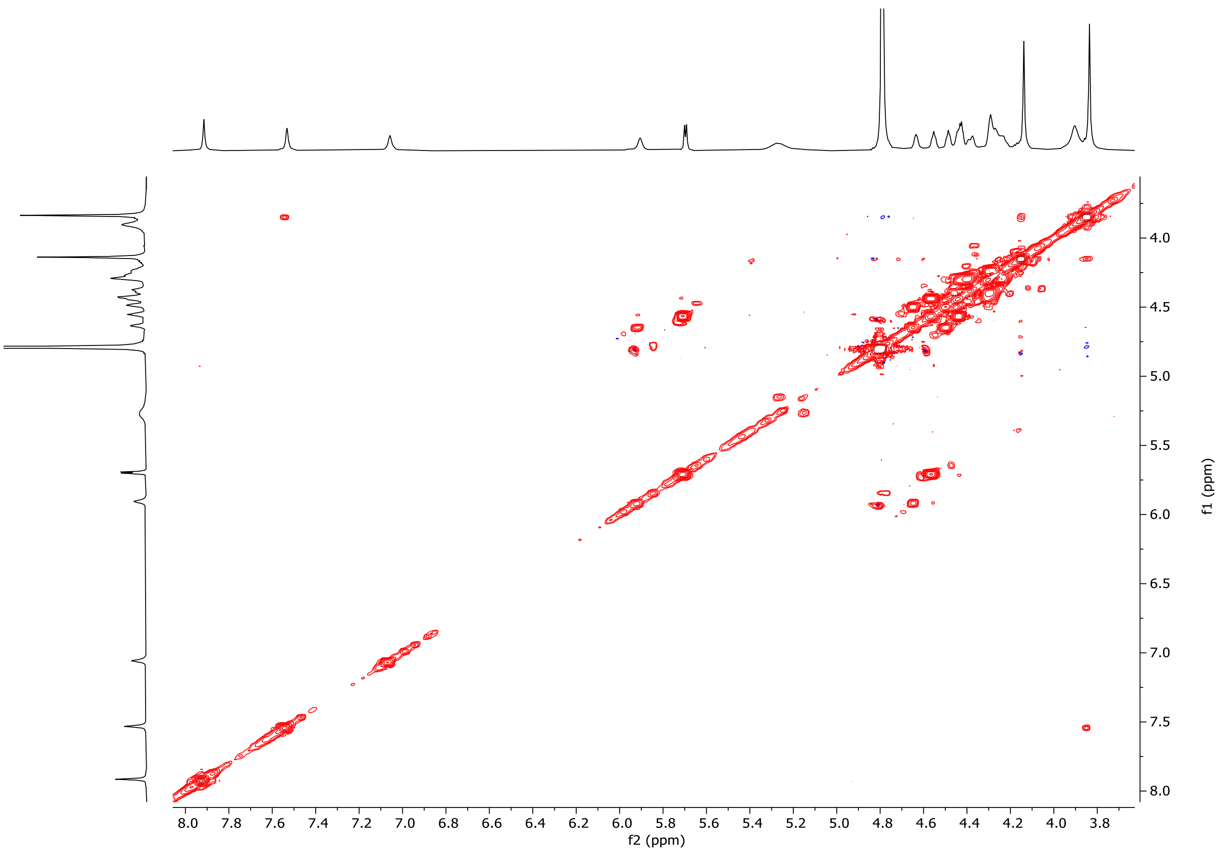

Supplementary Figure 41. COSY spectrum of compound 1.

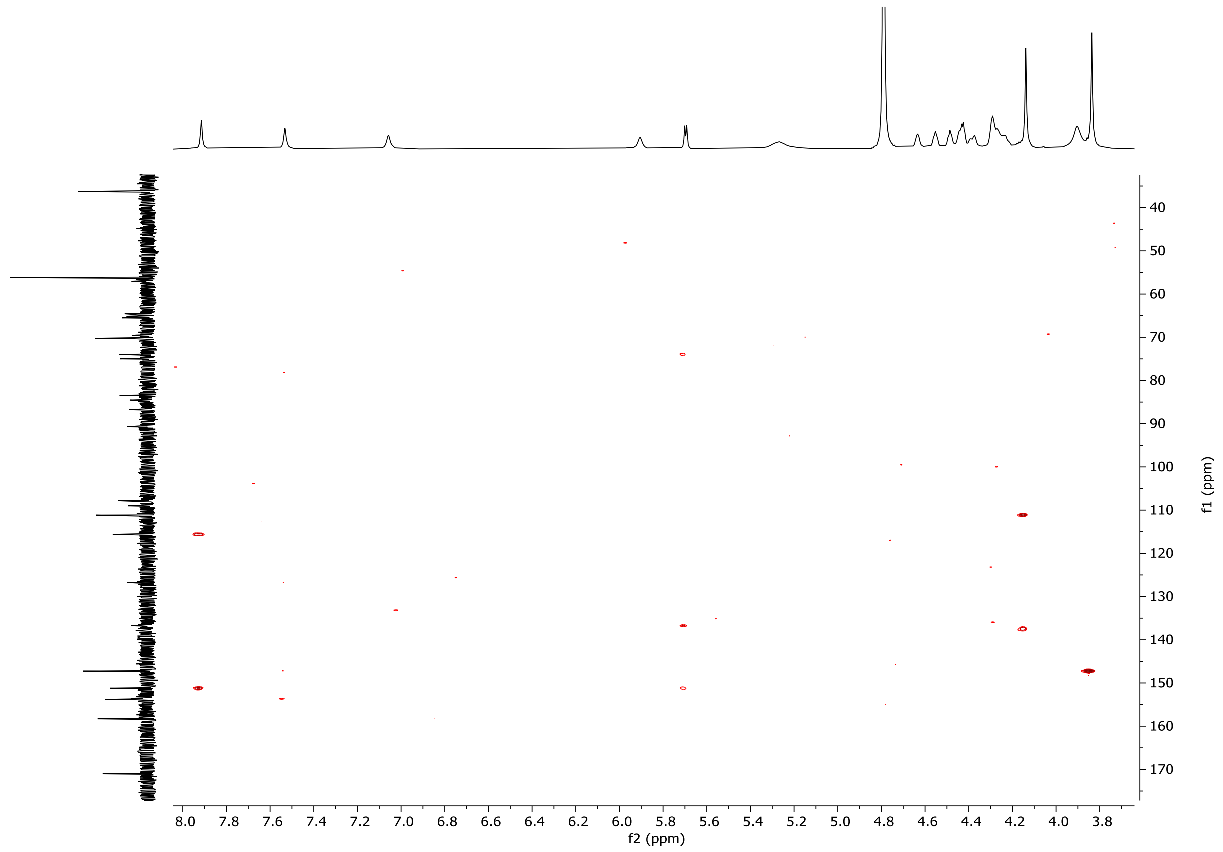

Supplementary Figure 42. HMBC spectrum of compound 1.

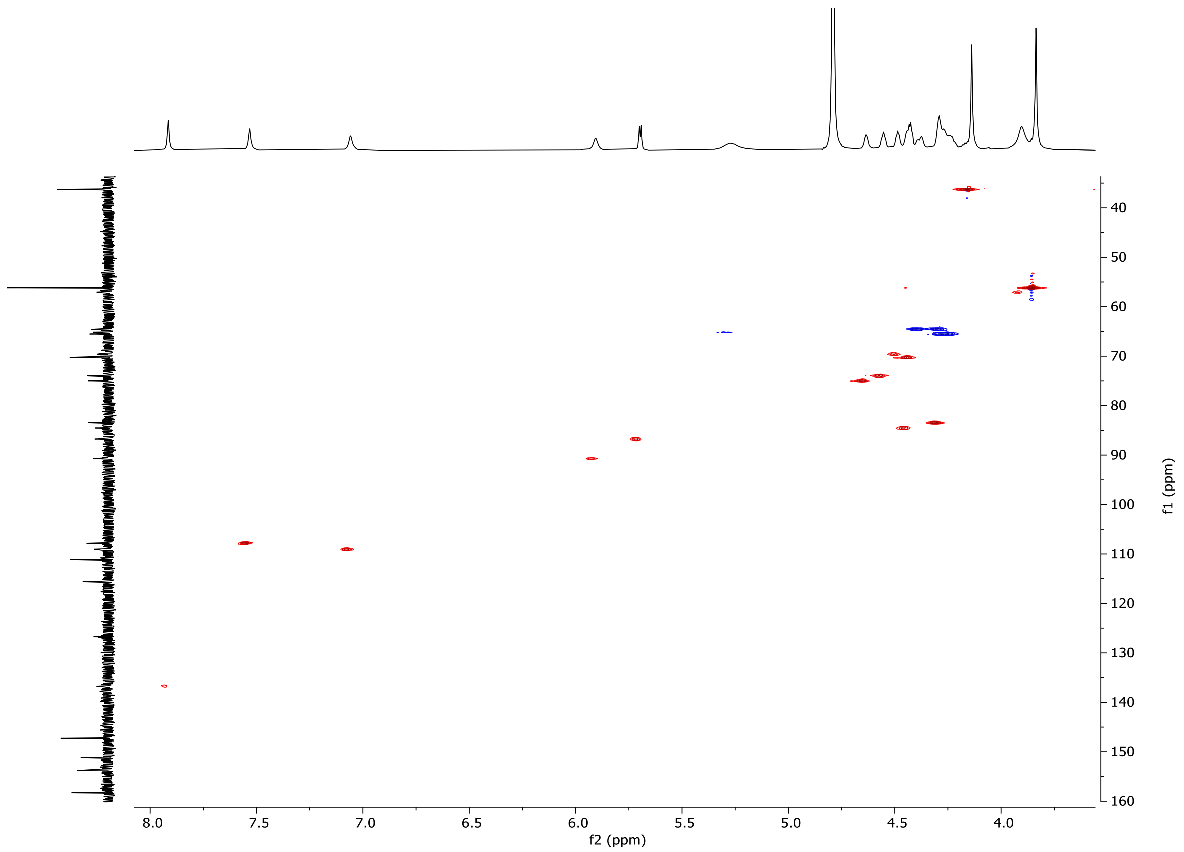

Supplementary Figure 43. HSQC spectrum of compound 1.

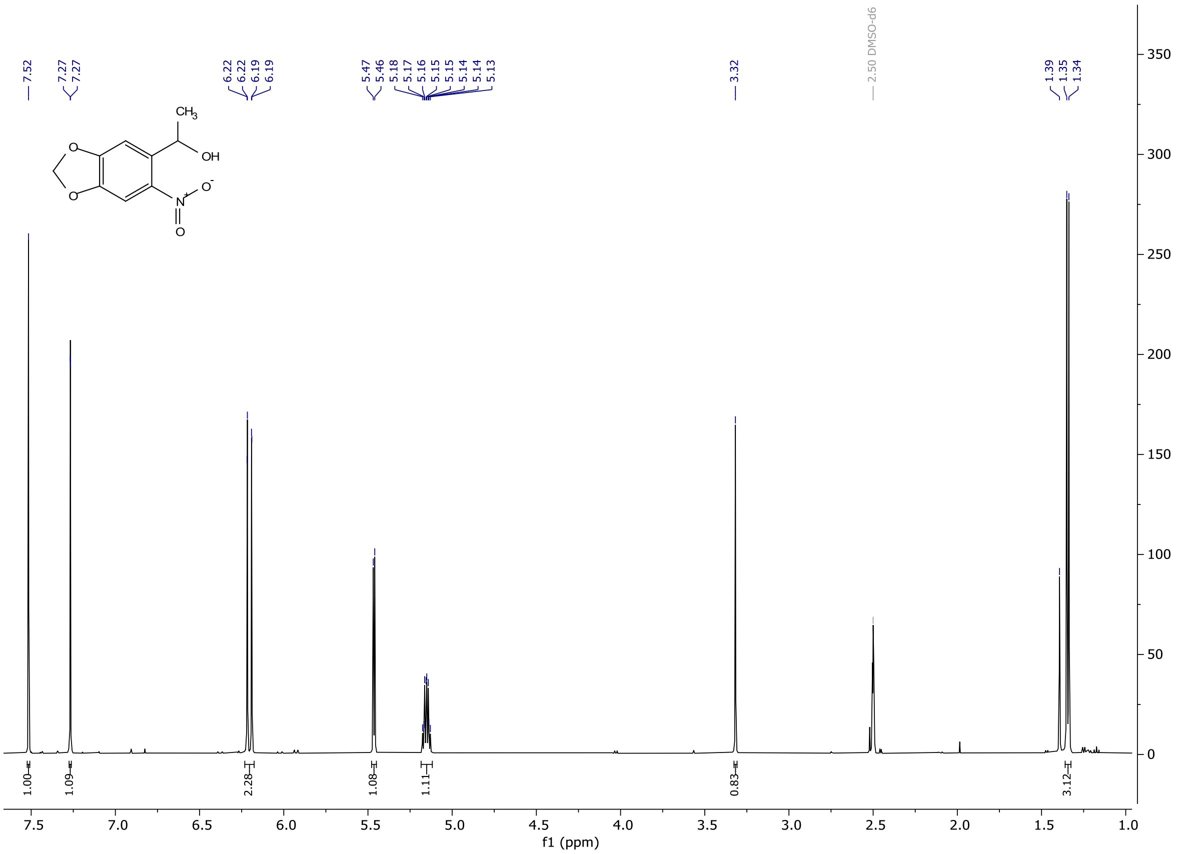

Supplementary Figure 44. ^{1}H-NMR spectrum of compound 4b.

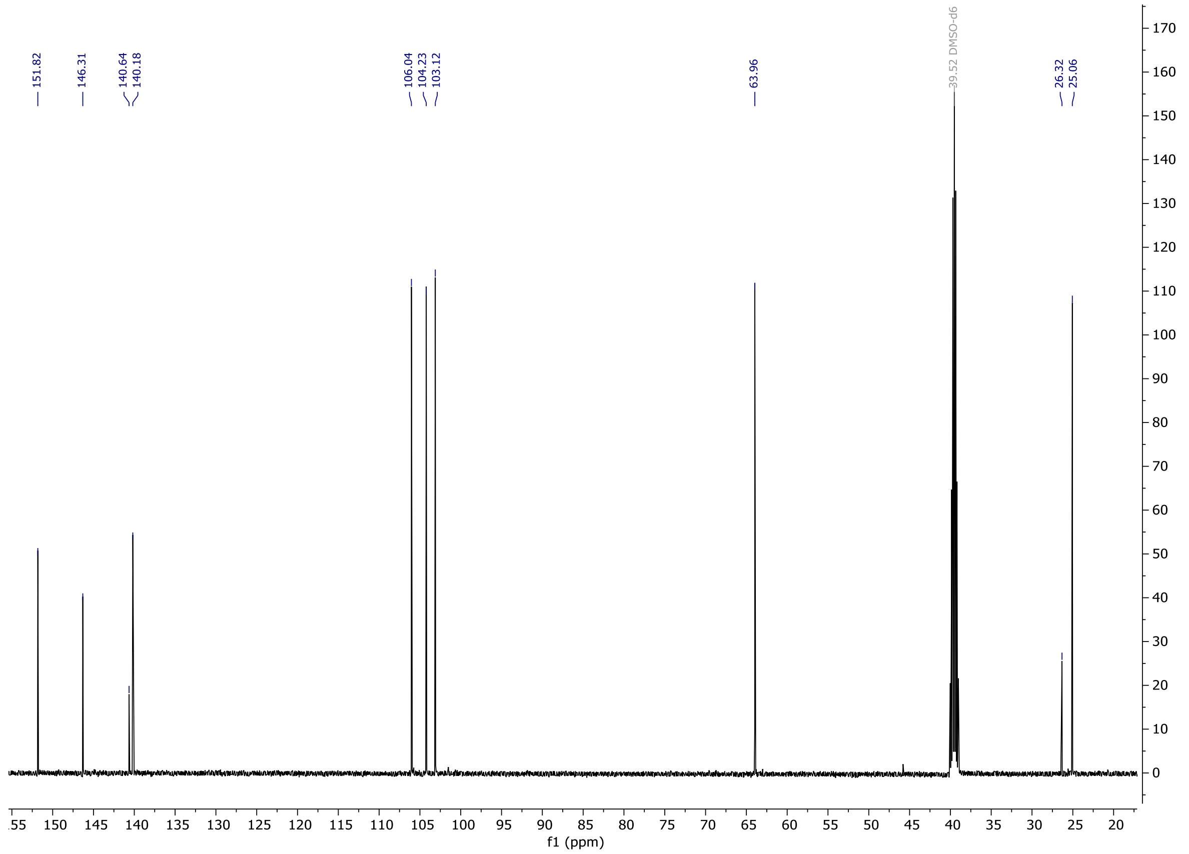

Supplementary Figure 45. ***^{13}C-NMR spectrum of compound 4b.***

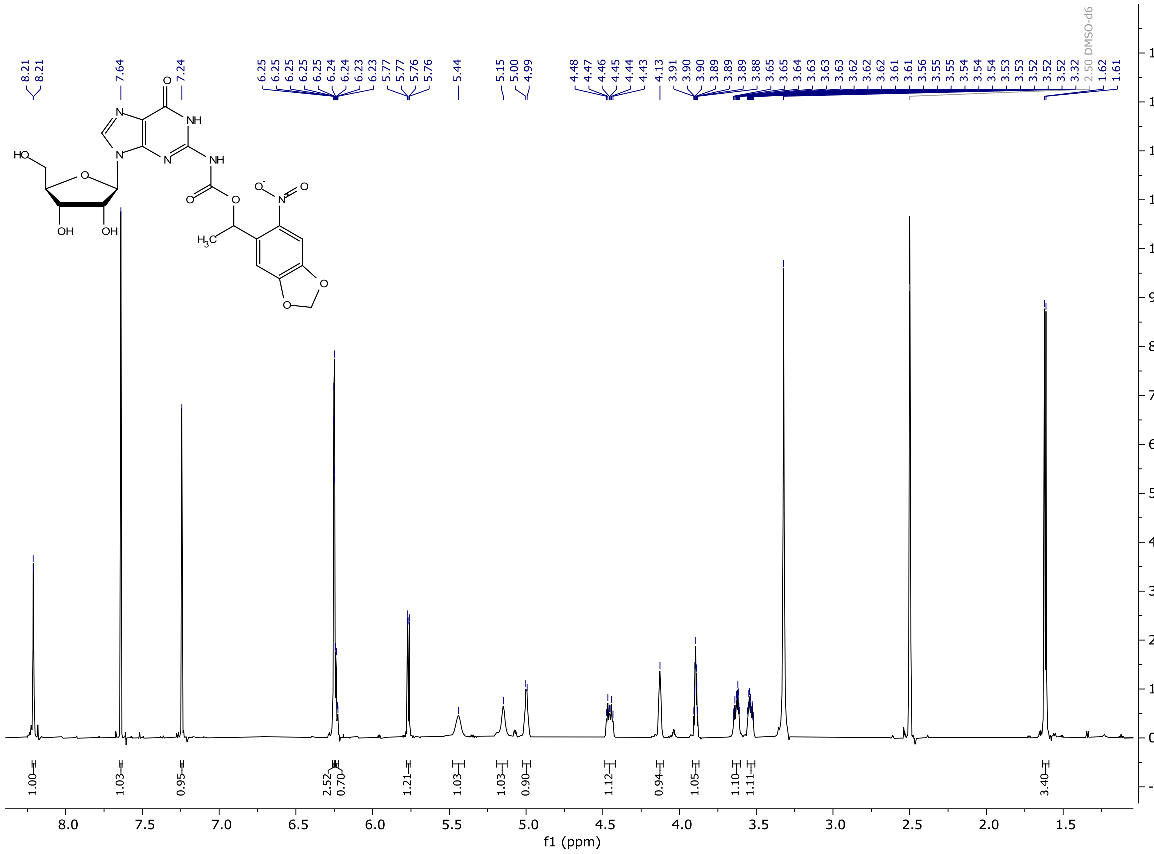

Supplementary Figure 46. **^{1}H-NMR spectrum of compound 5b.**

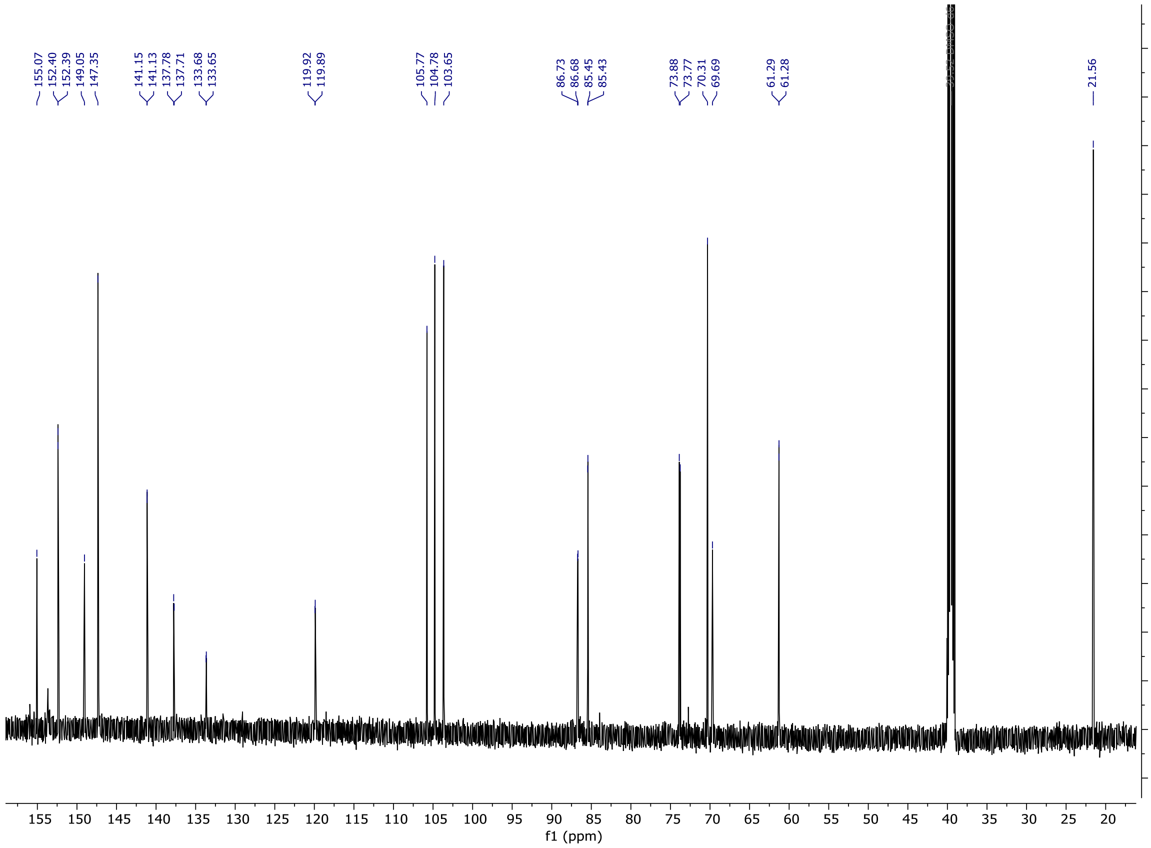

Supplementary Figure 47. ^{13}C-NMR spectrum of compound 5b.

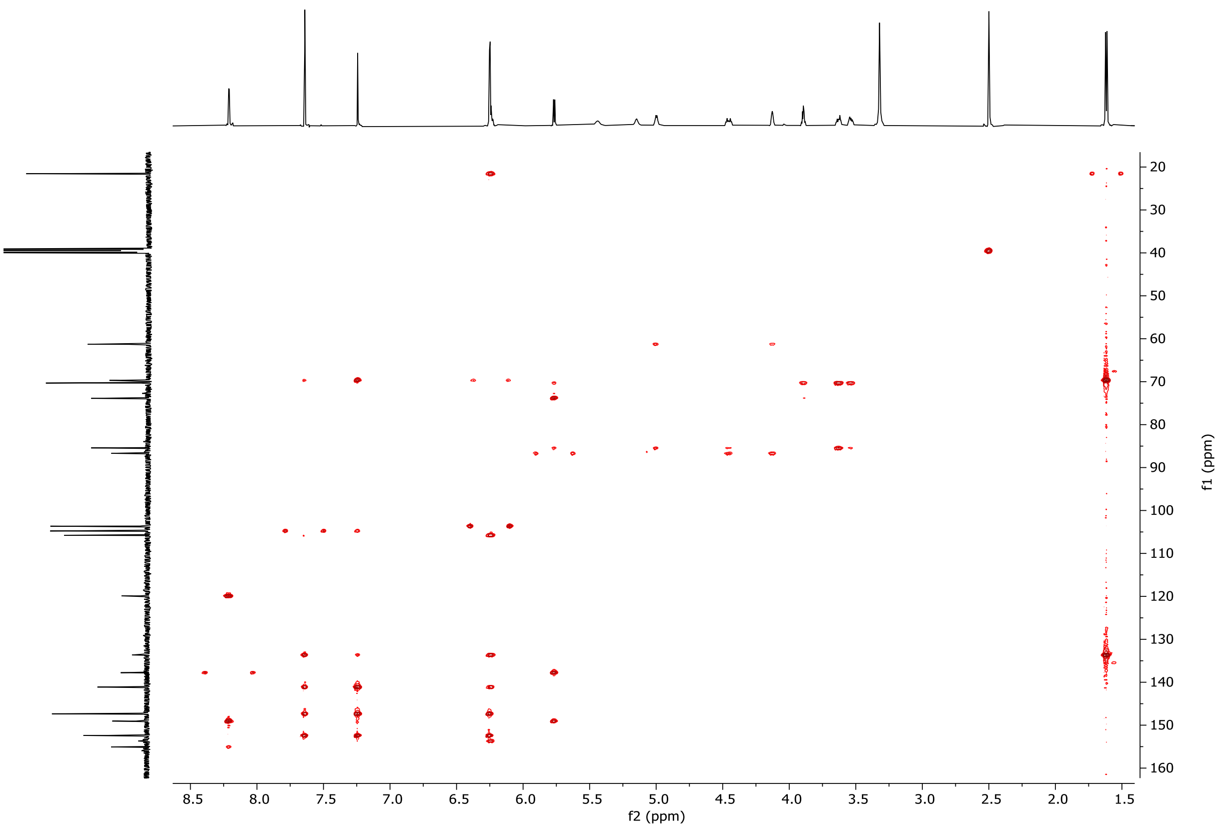

Supplementary Figure 48. HMBC spectrum of compound 5b.

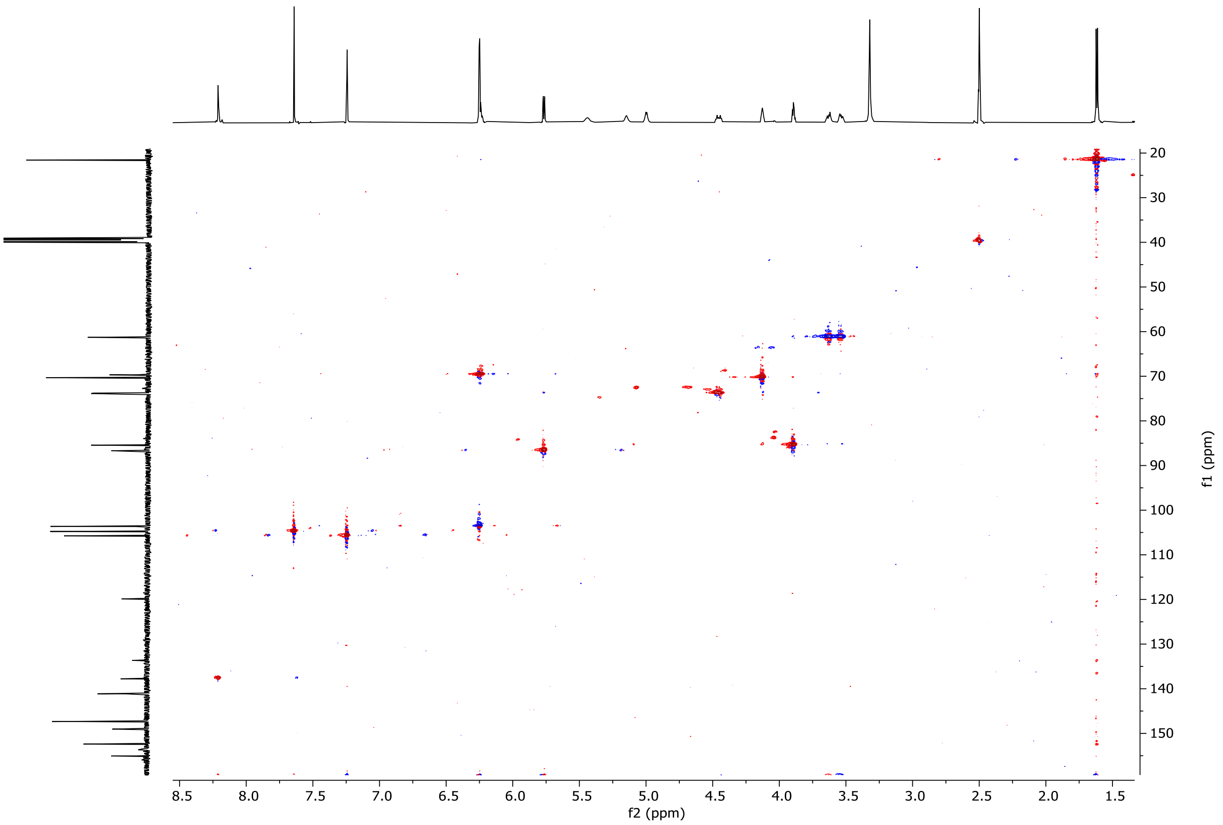

Supplementary Figure 49. HSQC spectrum of compound 5b.

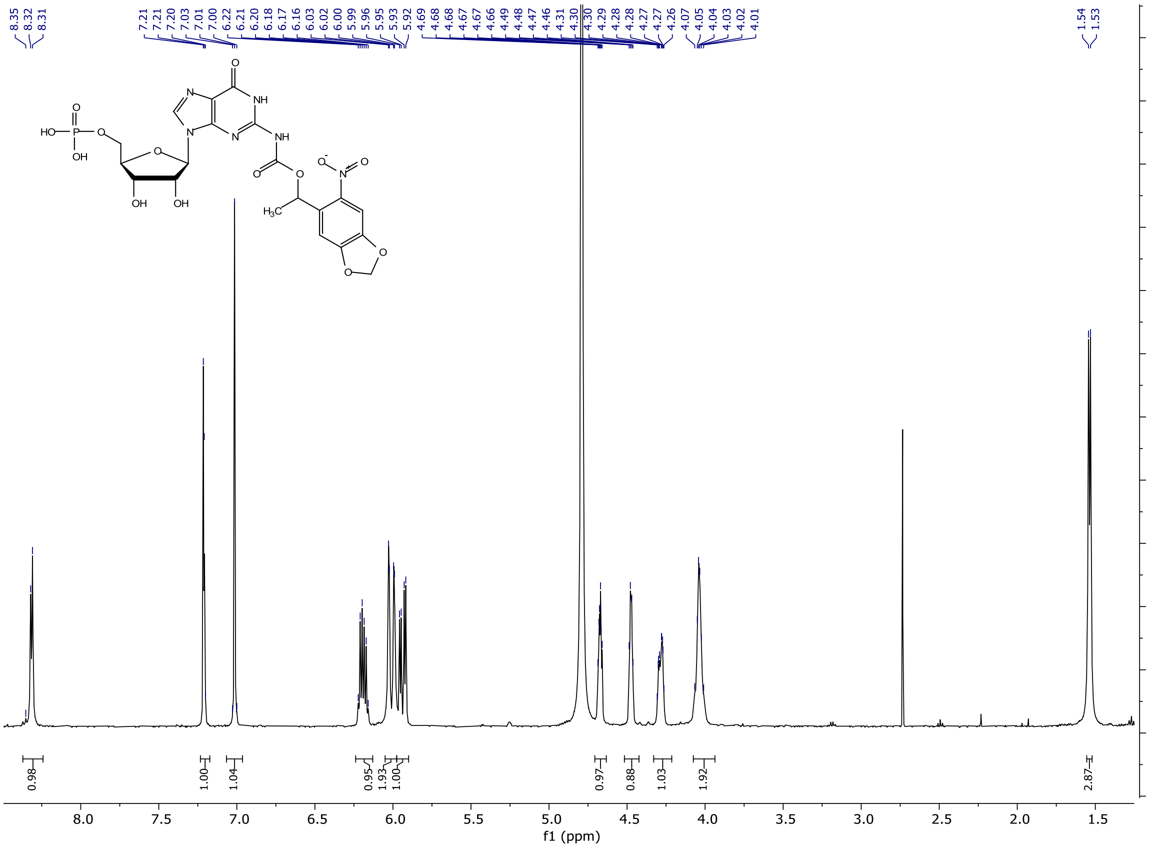

Supplementary Figure 50. ^{1}H-NMR spectrum of compound 6b.

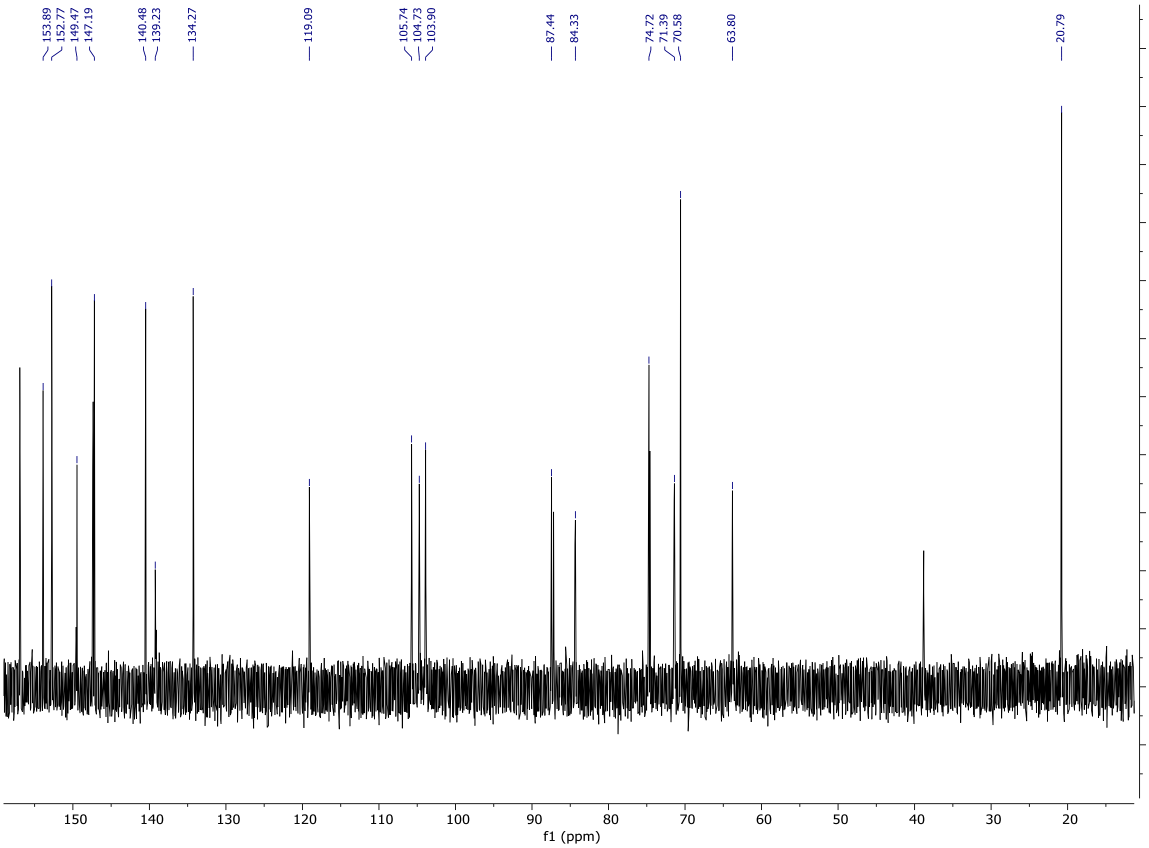

Supplementary Figure 51. ^{13}C-NMR spectrum of compound 6b.

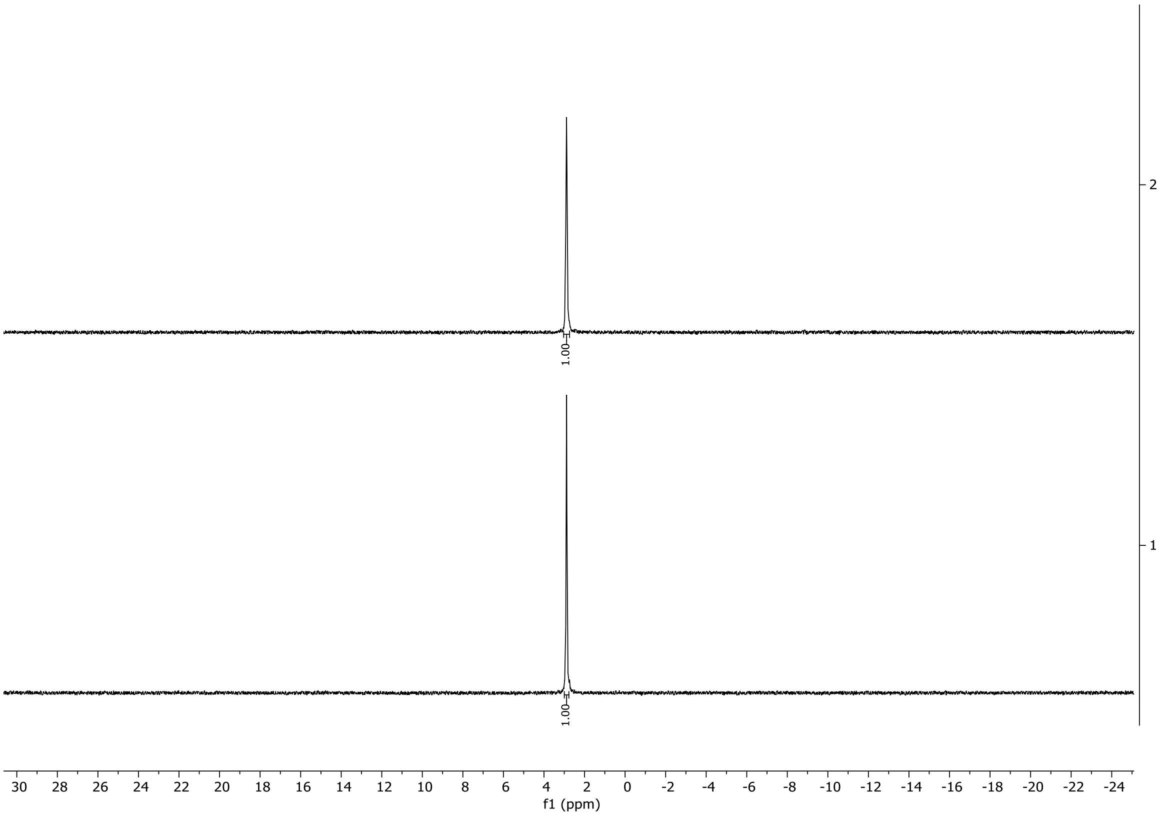

Supplementary Figure 52. ^{31}P-NMR spectrum of compound 6b.

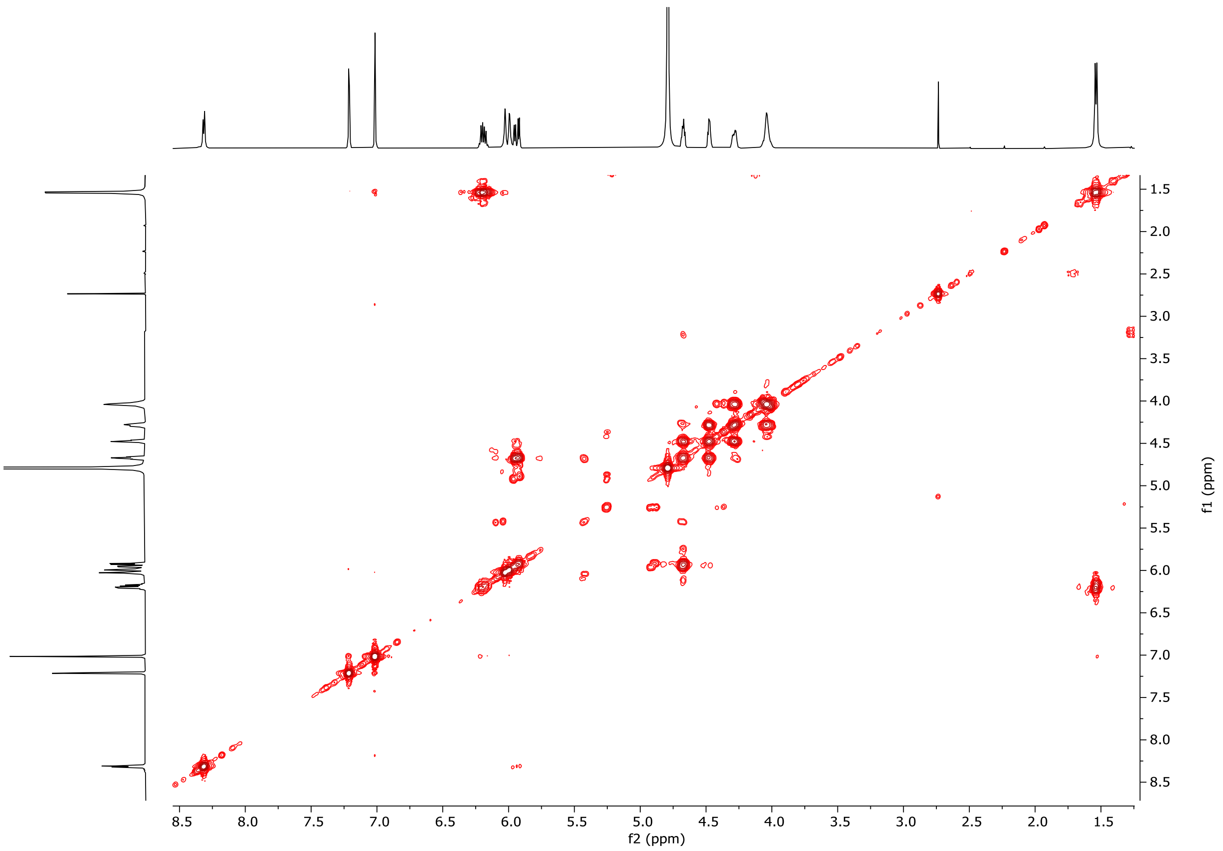

Supplementary Figure 53. COSY spectrum of compound 6b.

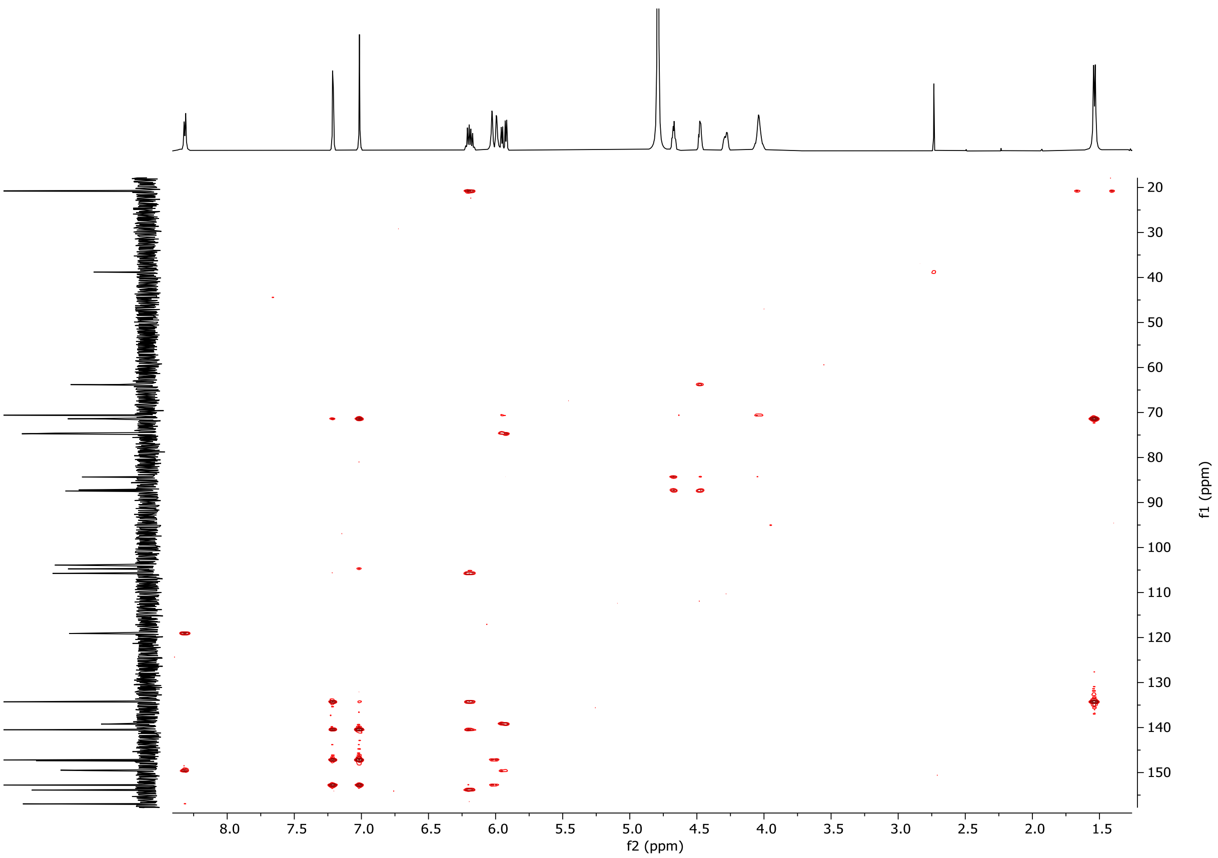

Supplementary Figure 54. HMBC spectrum of compound 6b.

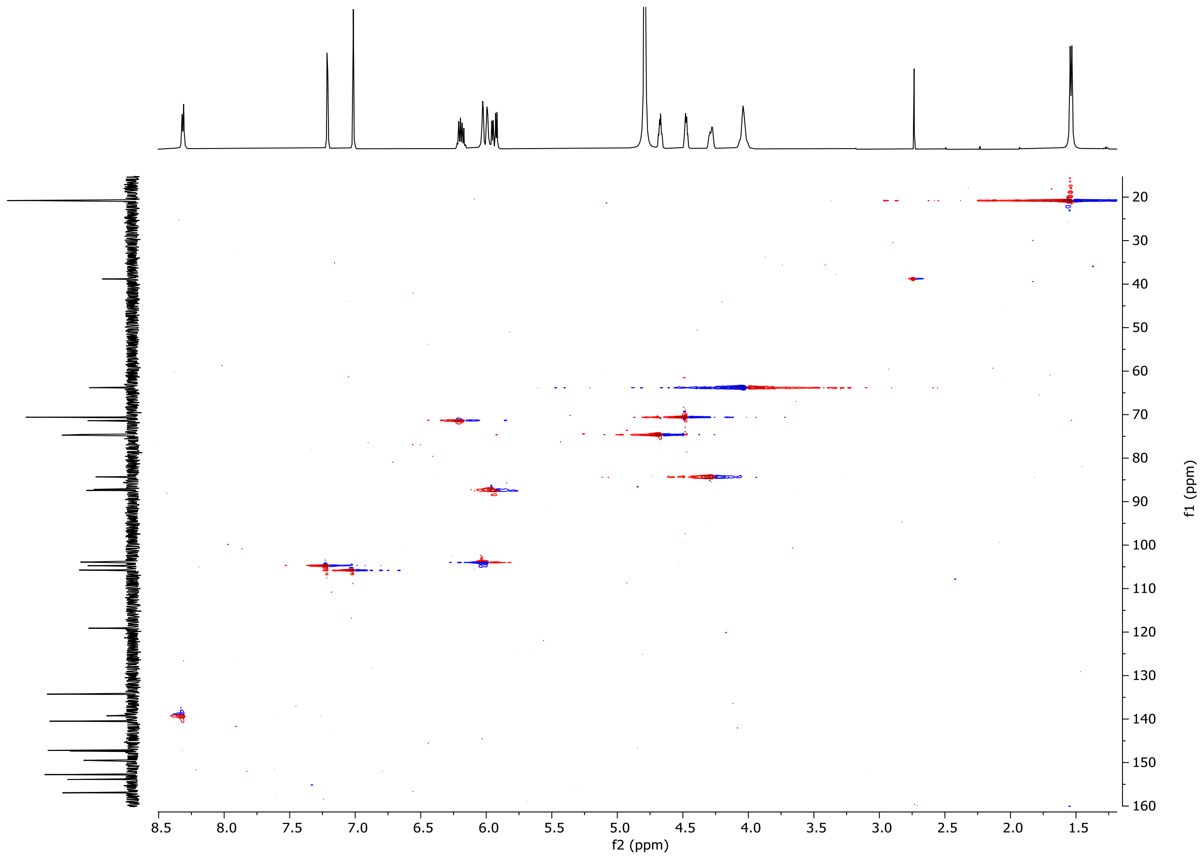

Supplementary Figure 55. HSQC spectrum of compound 6b.

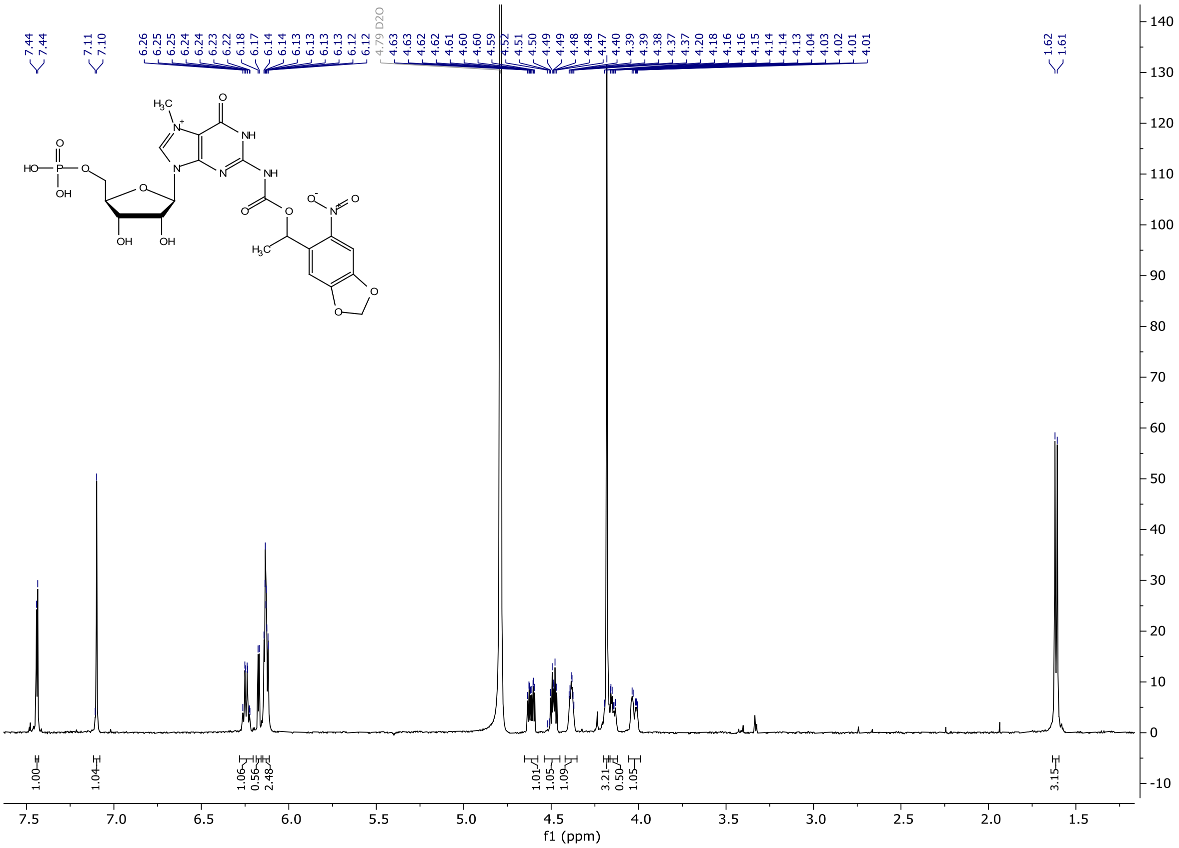

Supplementary Figure 56. ^{1}H-NMR spectrum of compound 7b.

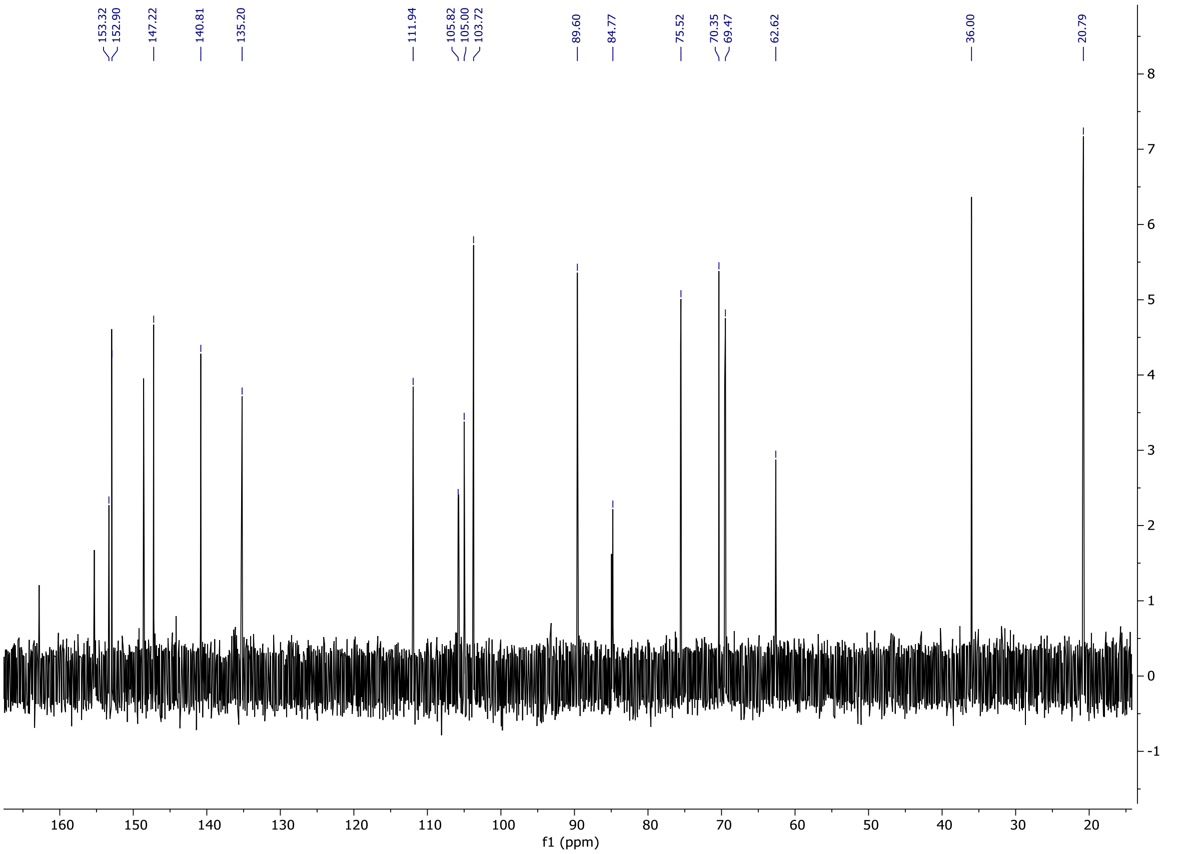

Supplementary Figure 57. ^{13}C-NMR spectrum of compound 7b.

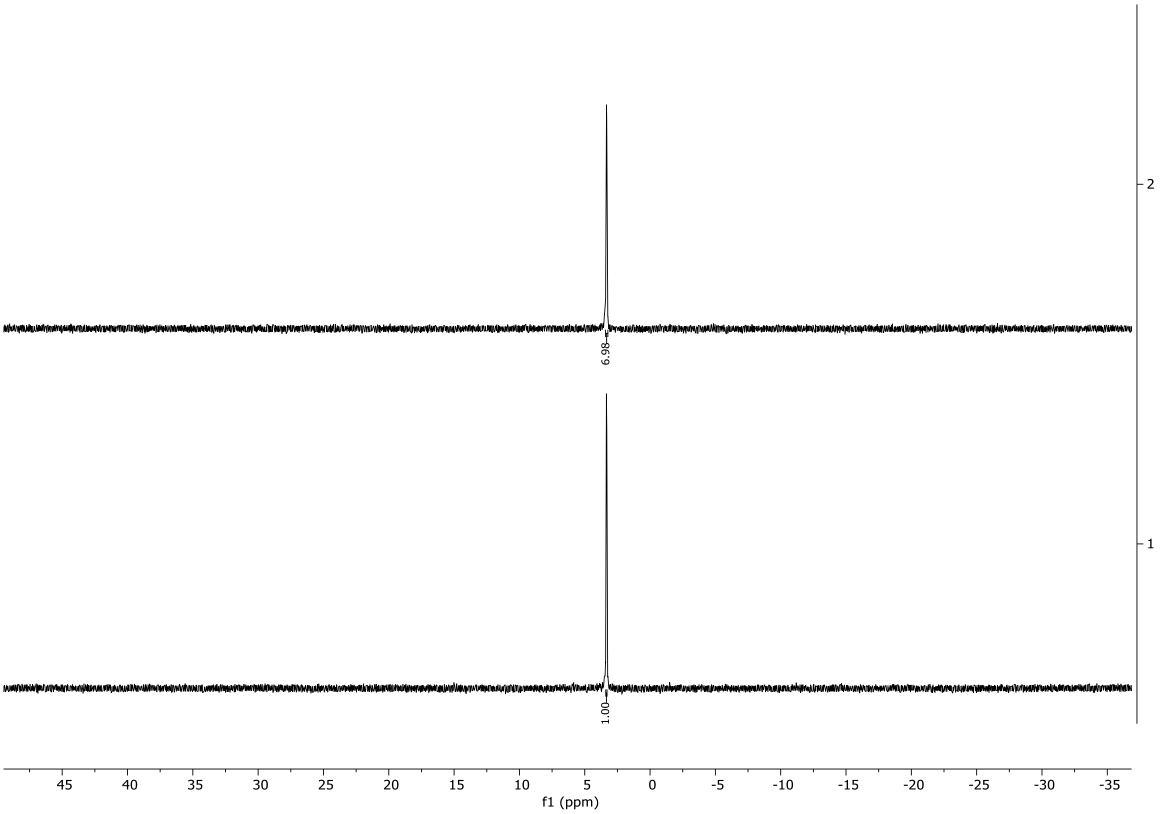

Supplementary Figure 58. ^{31}P-NMR spectrum of compound 7b.

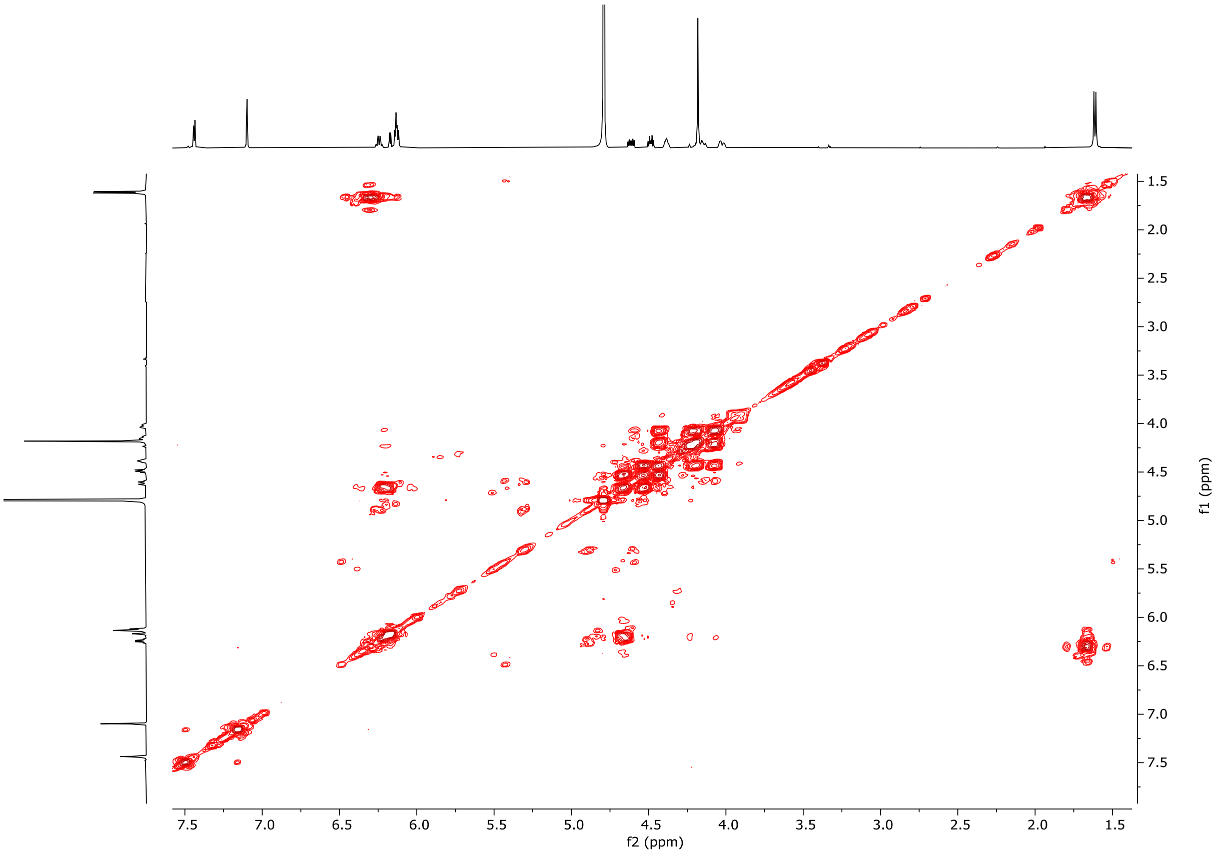

Supplementary Figure 59. COSY spectrum of compound 7b.

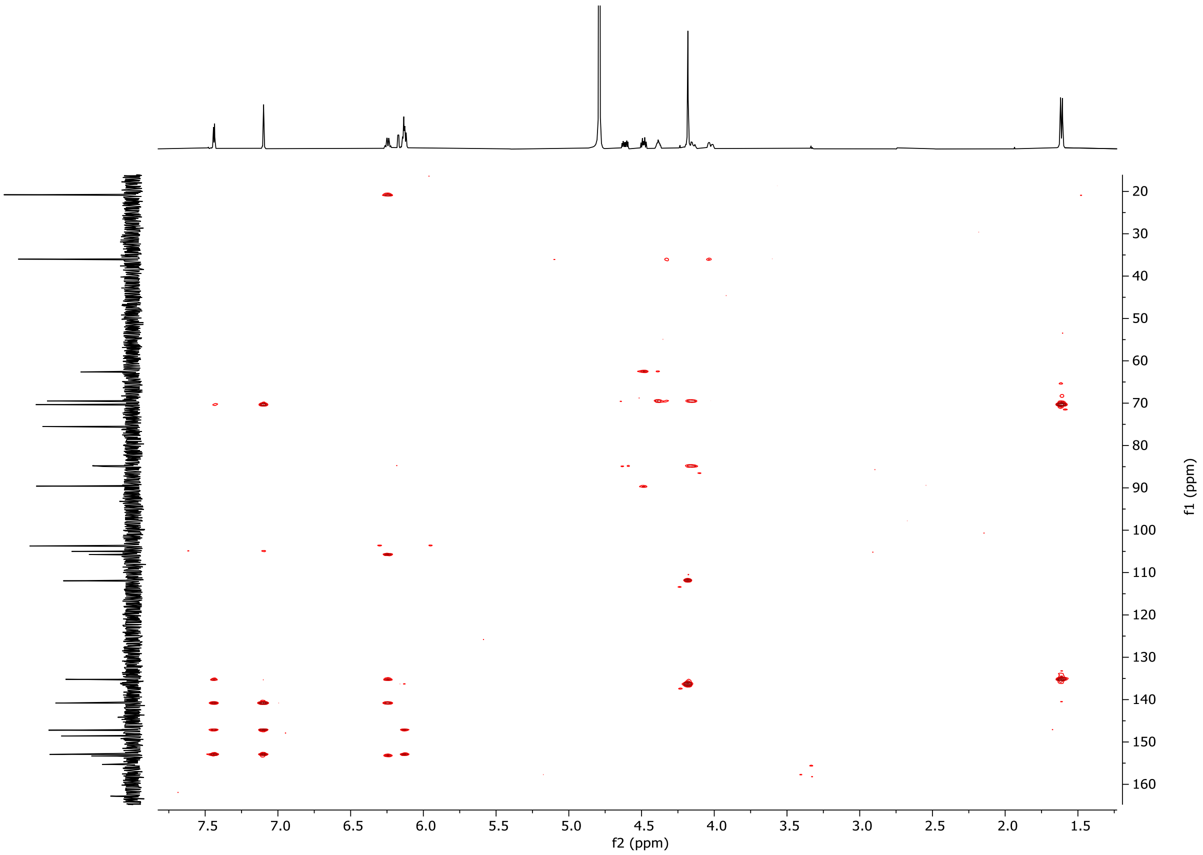

Supplementary Figure 60. HMBC spectrum of compound 7b.

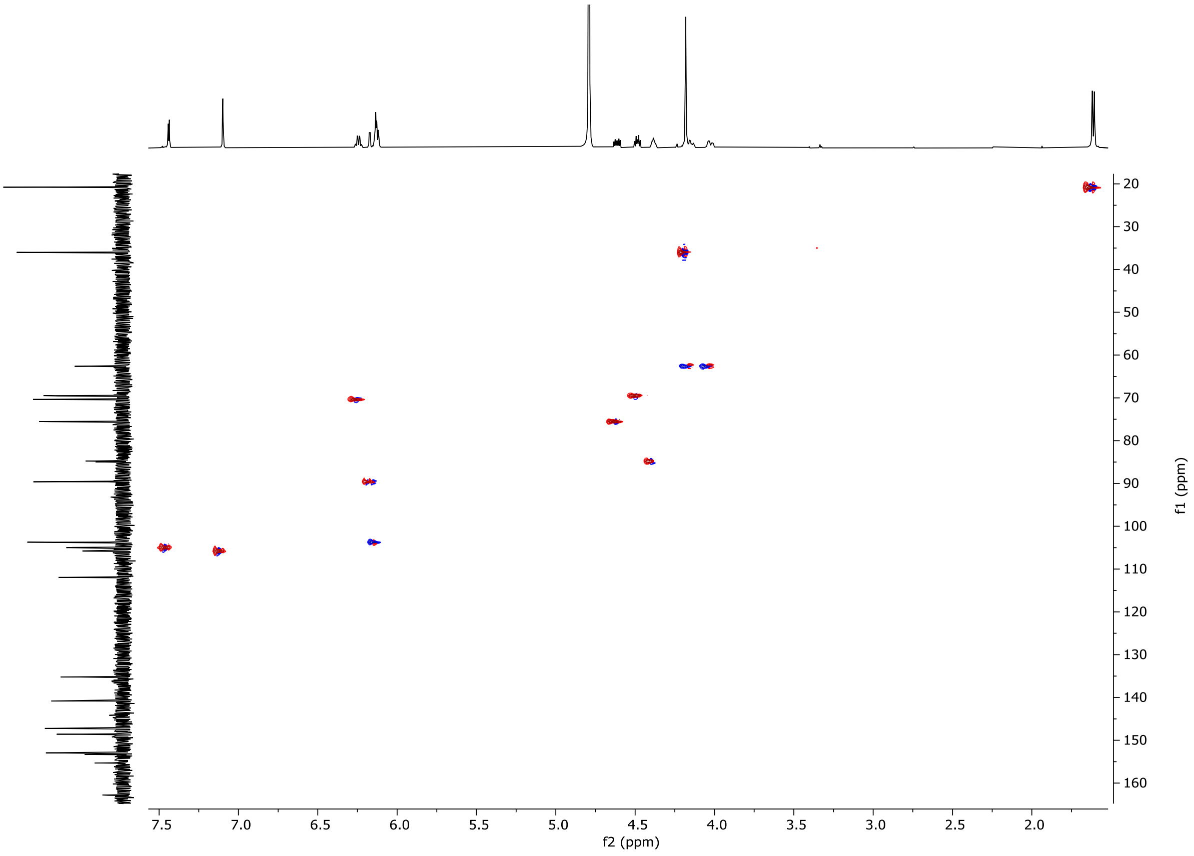

Supplementary Figure 61. HSQC spectrum of compound 7b.

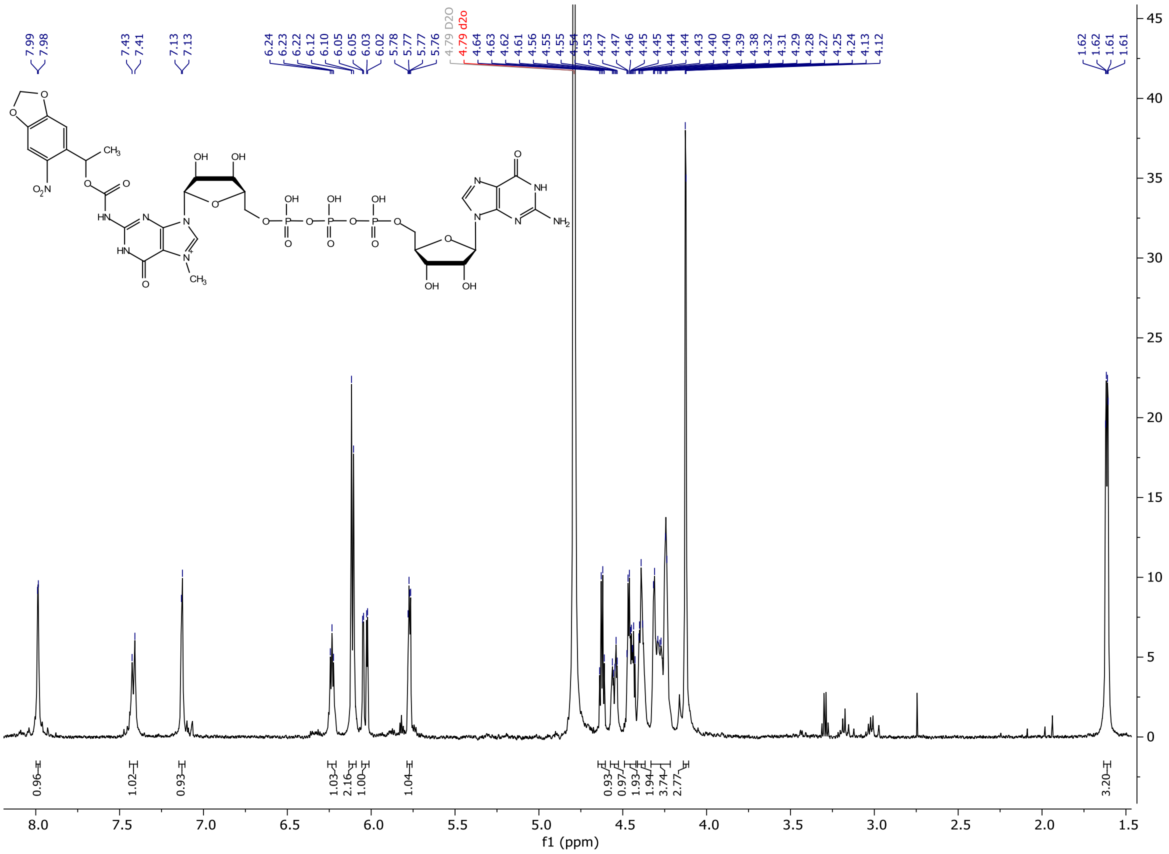

Supplementary Figure 62. ^{1}H-NMR spectrum of compound 2.

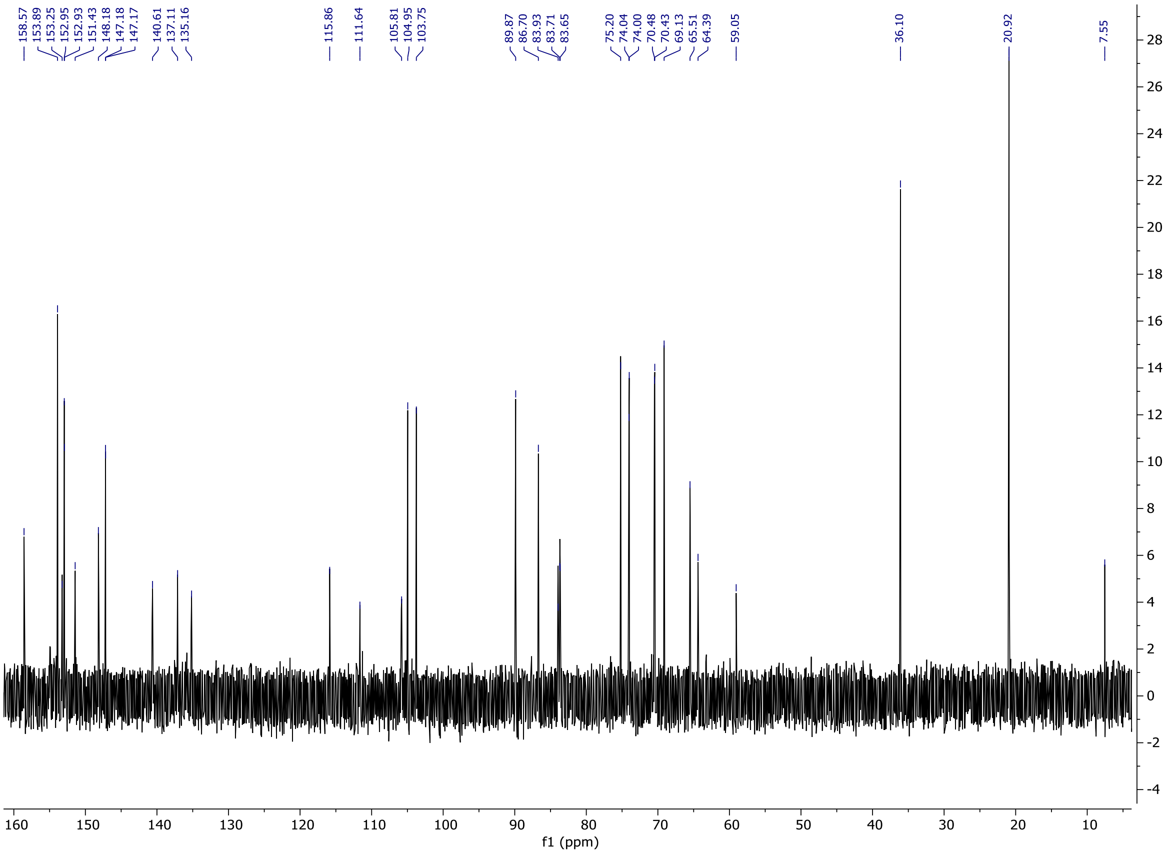

Supplementary Figure 63. ^{13}C-NMR spectrum of compound 2.

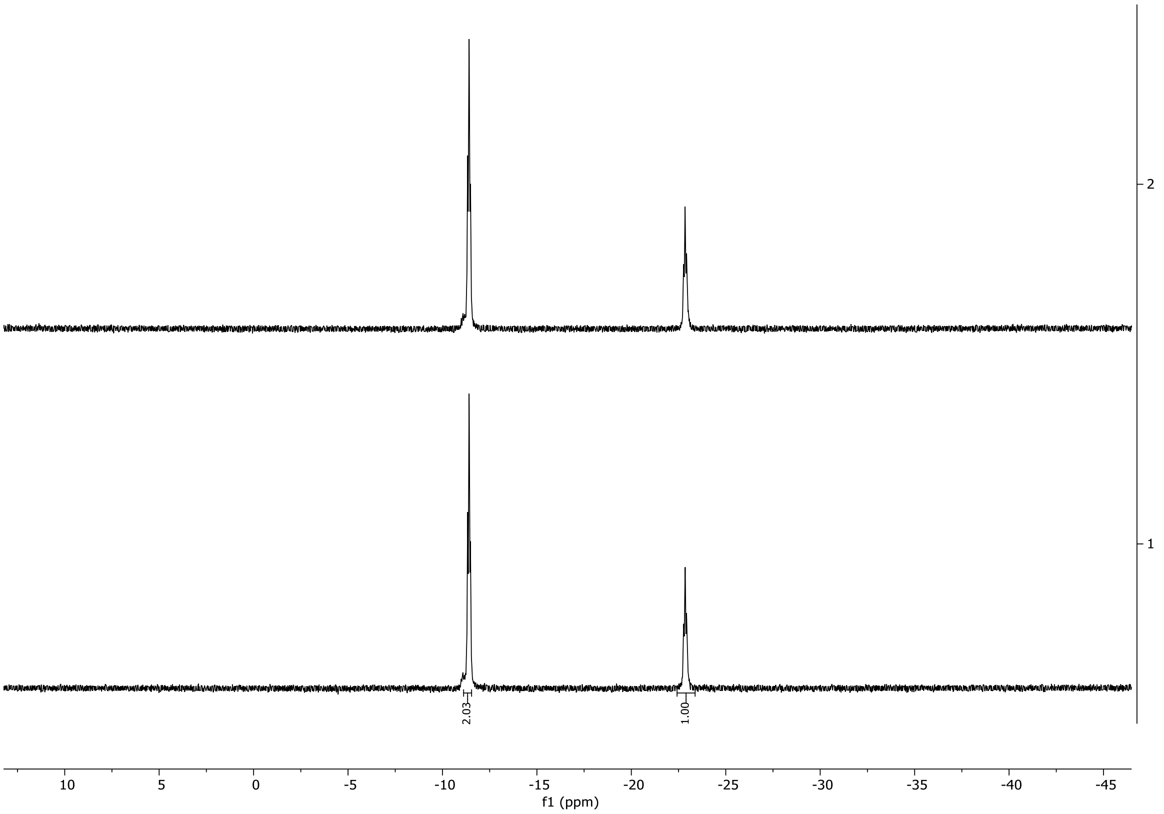

Supplementary Figure 64. ^{31}P-NMR spectrum of compound 2.

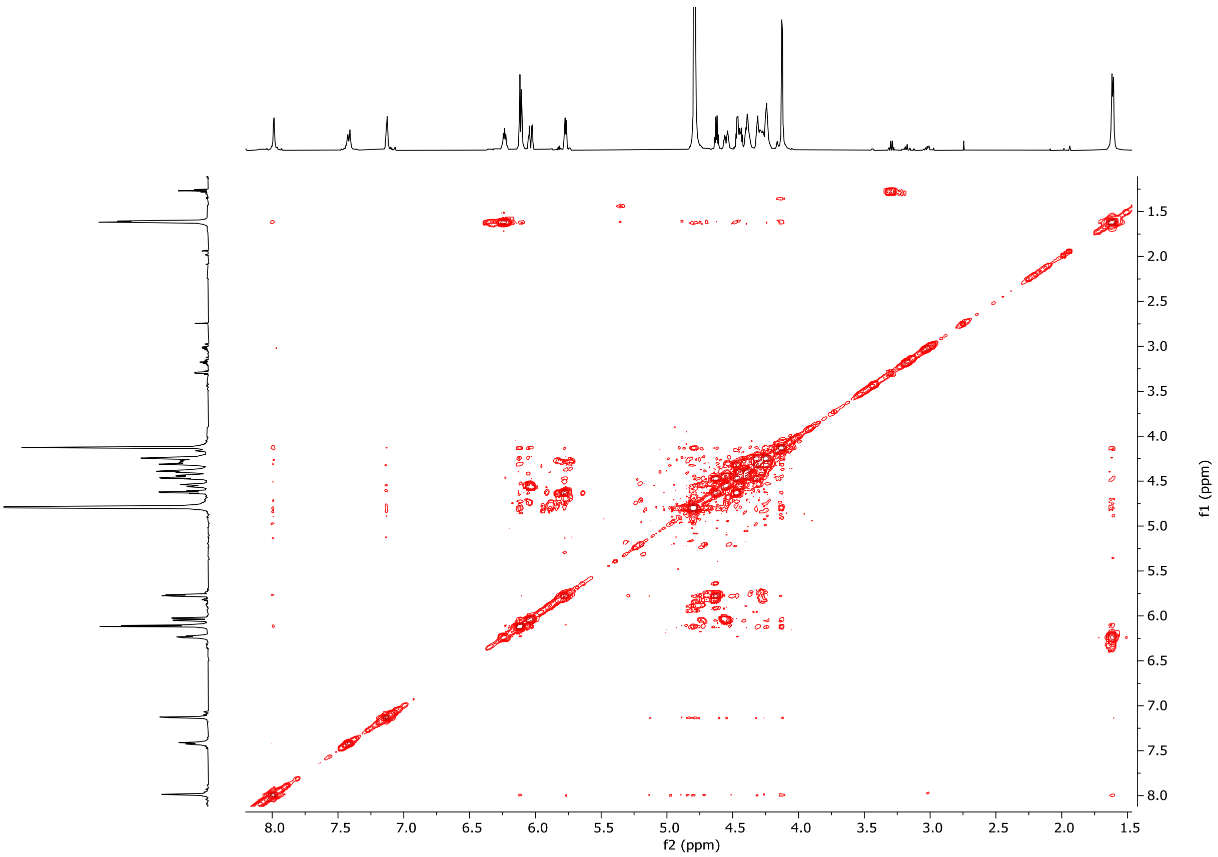

Supplementary Figure 65. COSY spectrum of compound 2.

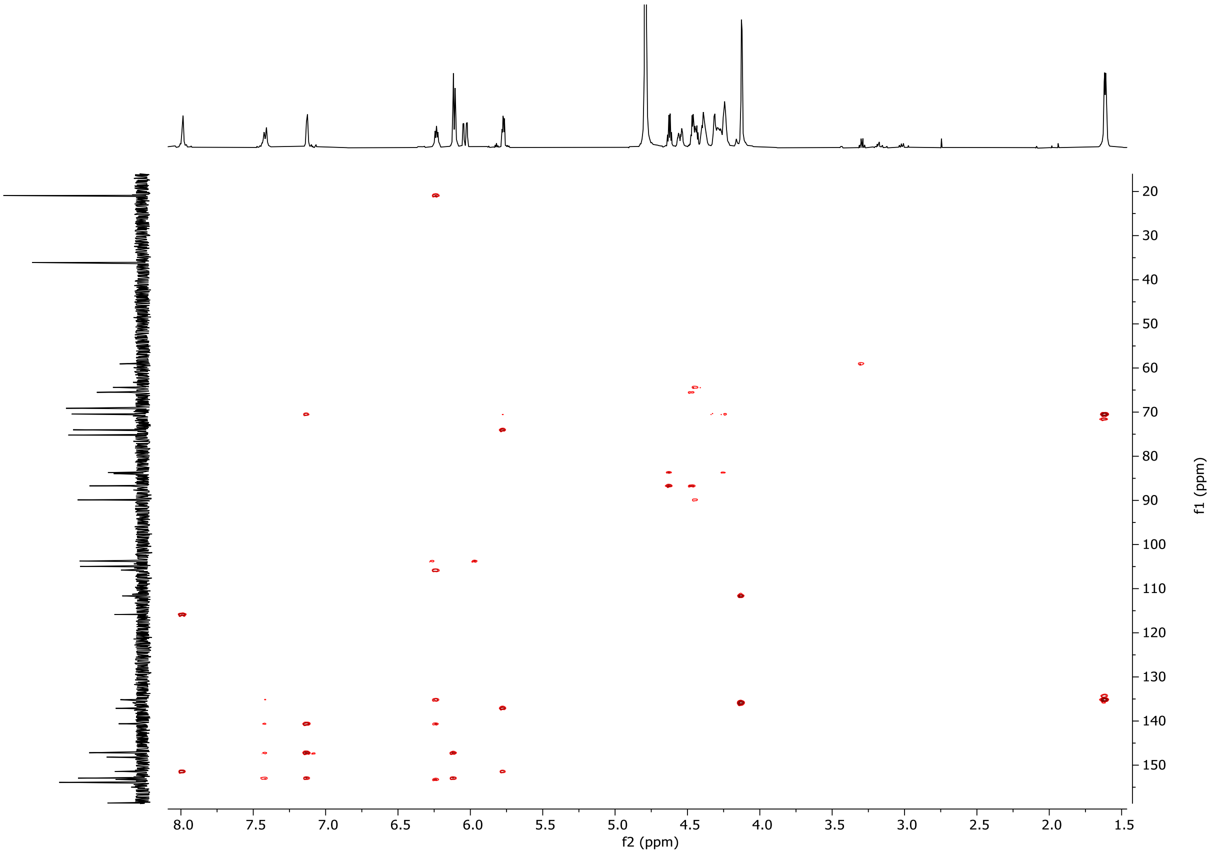

Supplementary Figure 66. HMBC spectrum of compound 2.

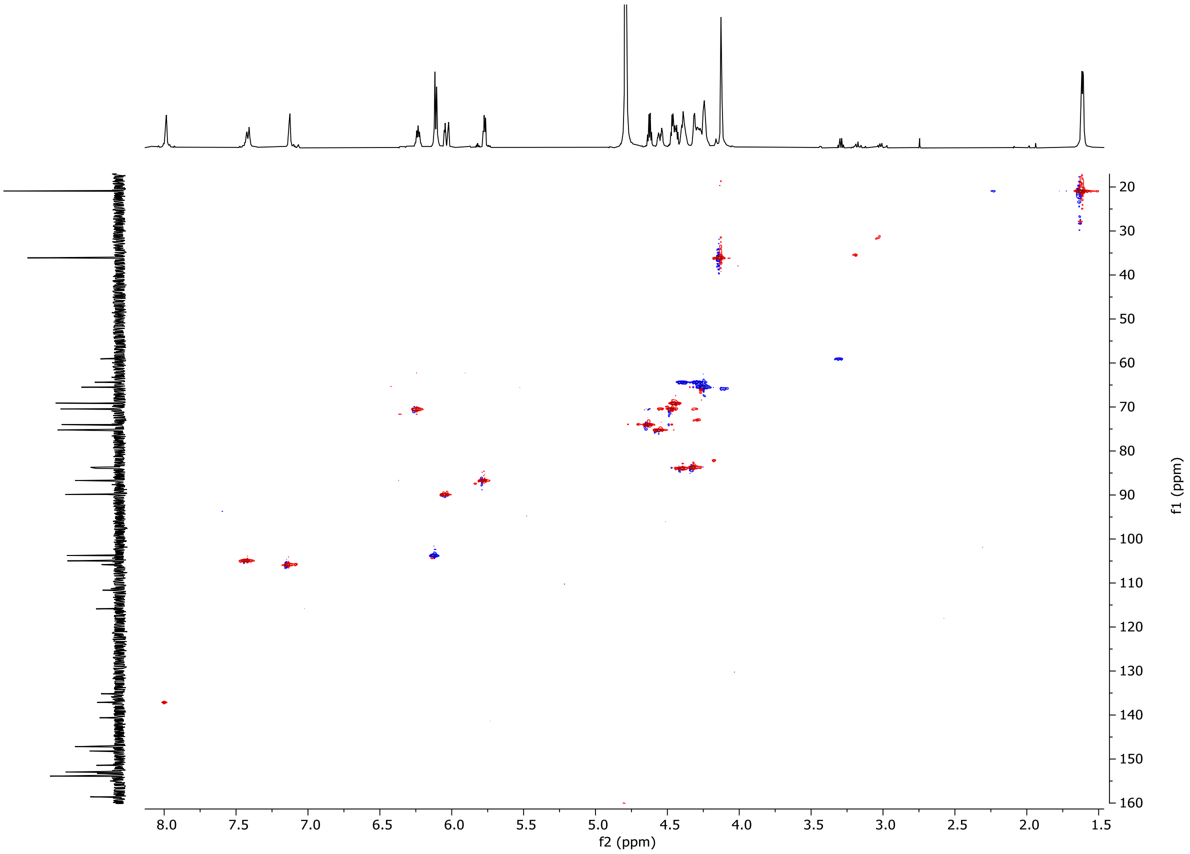

Supplementary Figure 67. HSQC spectrum of compound 2.

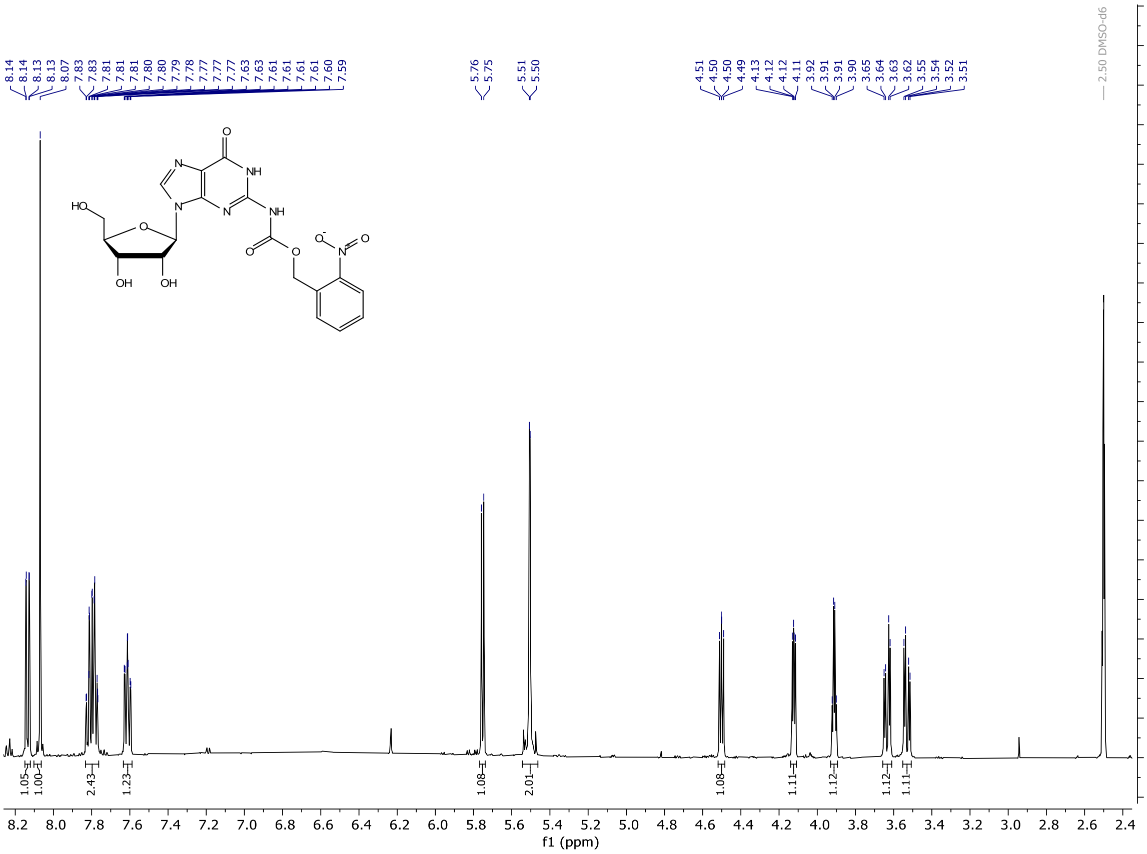

Supplementary Figure 68. **^{1}H-NMR spectrum of compound 5c.**

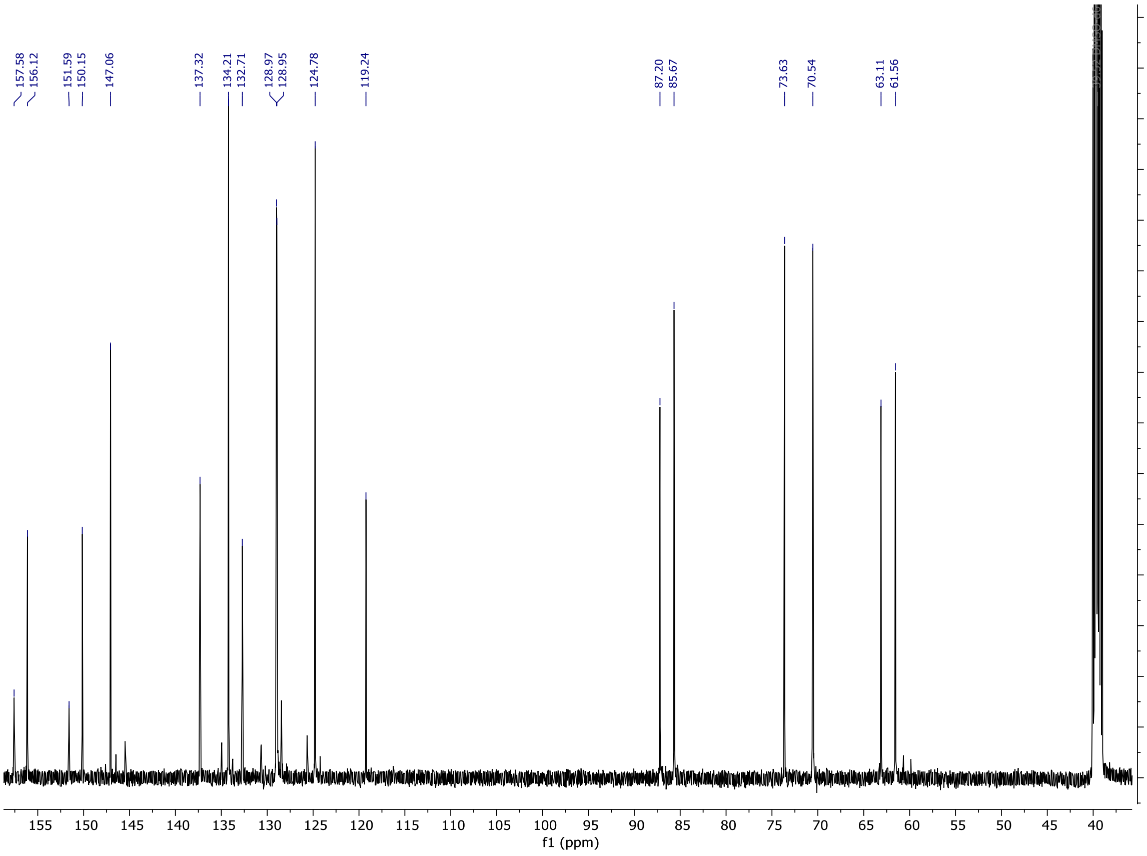

Supplementary Figure 69. **^{13}C-NMR spectrum of compound 5c.**

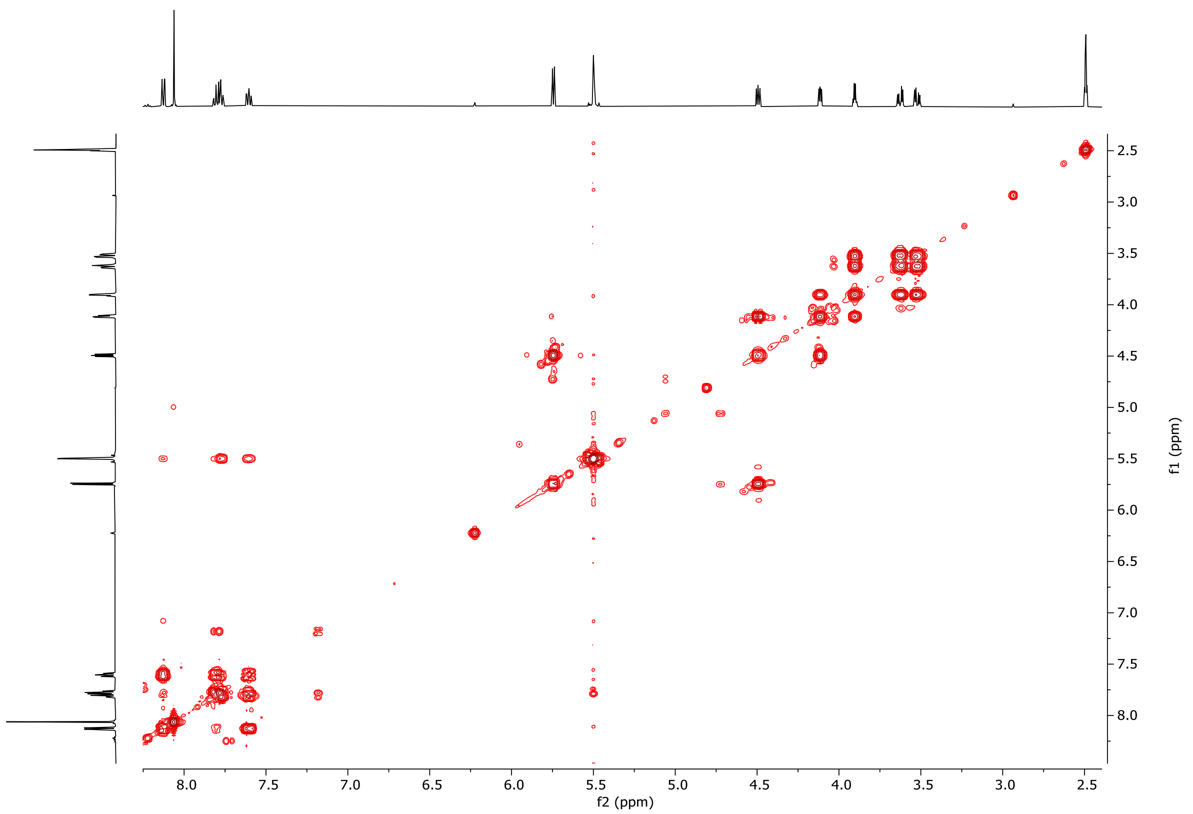

Supplementary Figure 70. COSY spectrum of compound 5c.

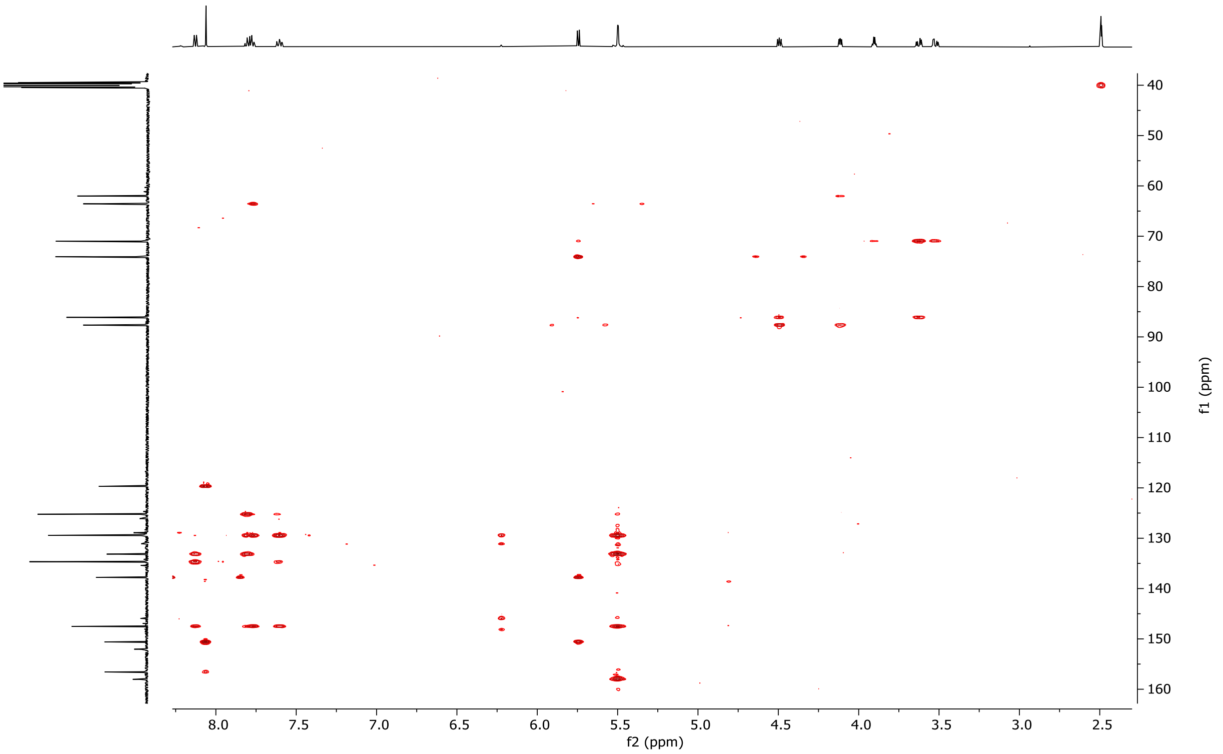

Supplementary Figure 71. HMBC spectrum of compound 5c.

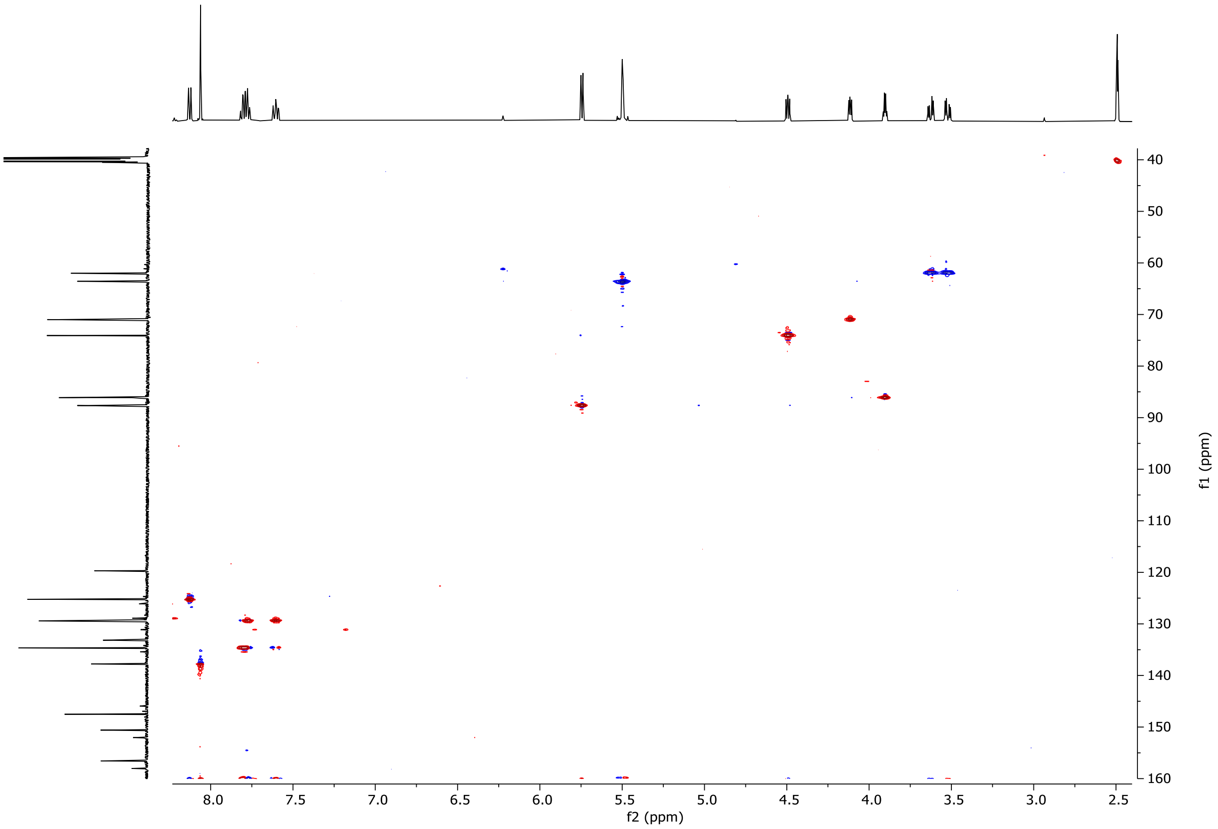

Supplementary Figure 72. HSQC spectrum of compound 5c.

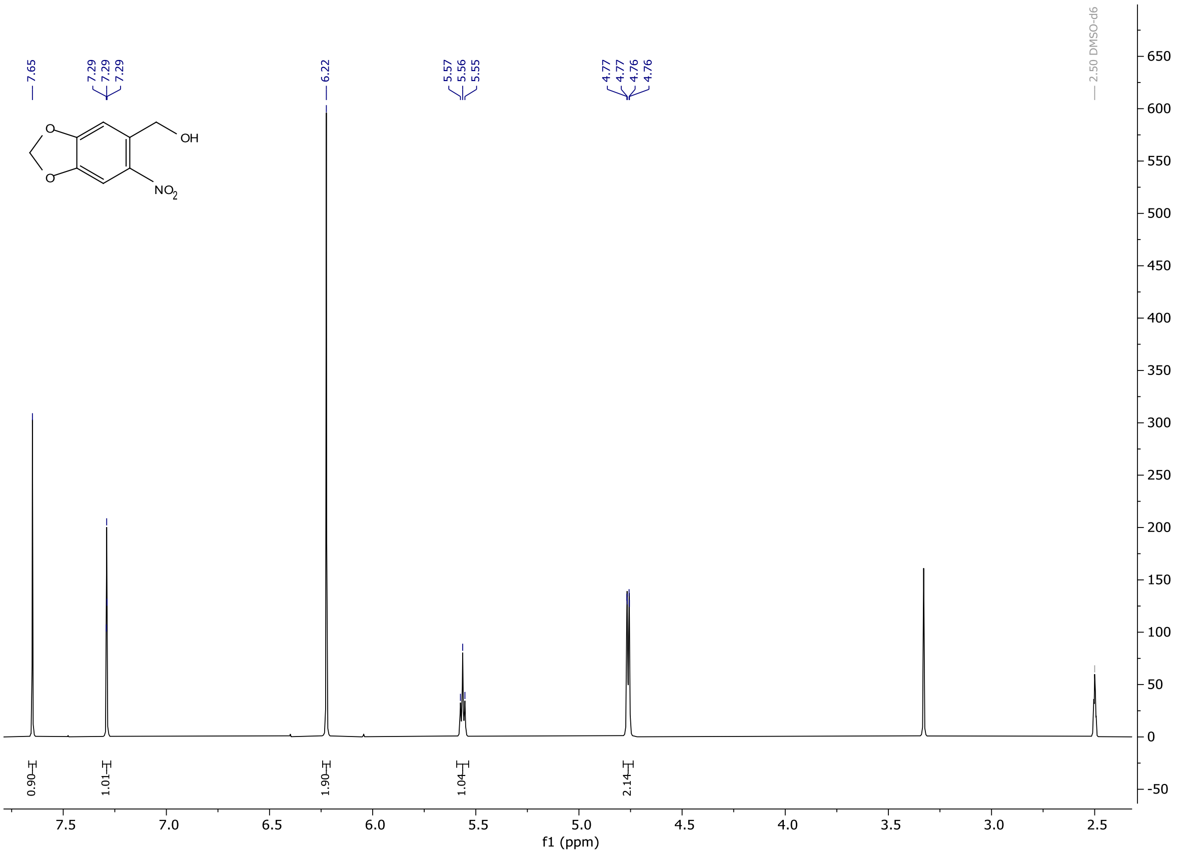

Supplementary Figure 73. ^{1}H-NMR spectrum of compound 4d.

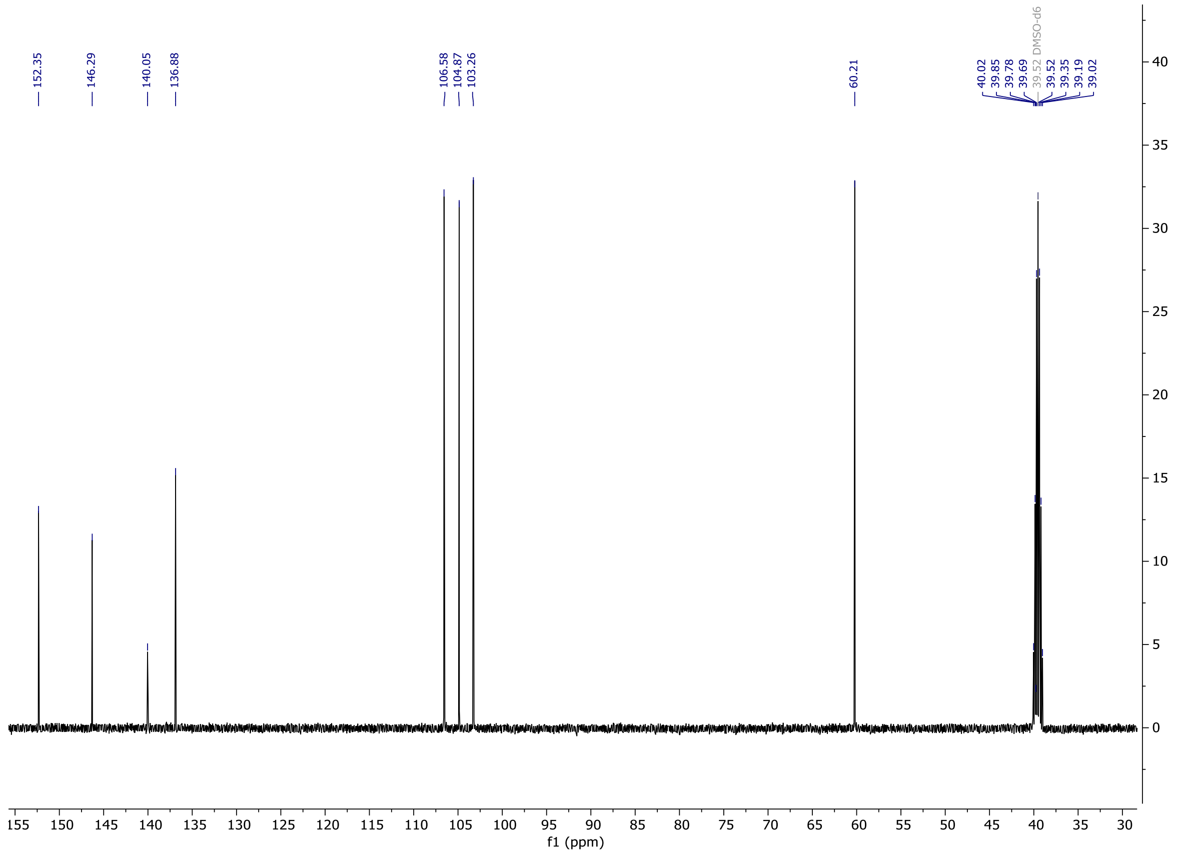

Supplementary Figure 74. ^{13}C-NMR spectrum of compound 4d.

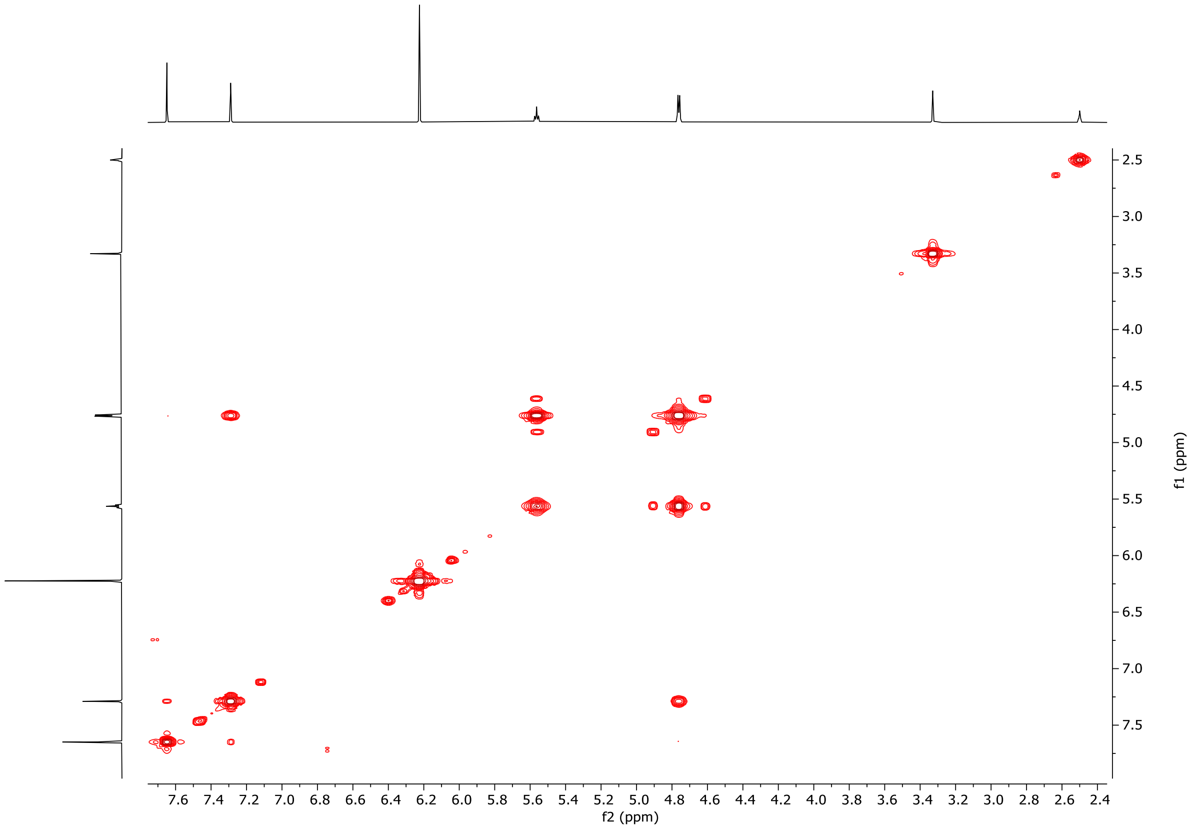

Supplementary Figure 75. COSY spectrum of compound 4d.

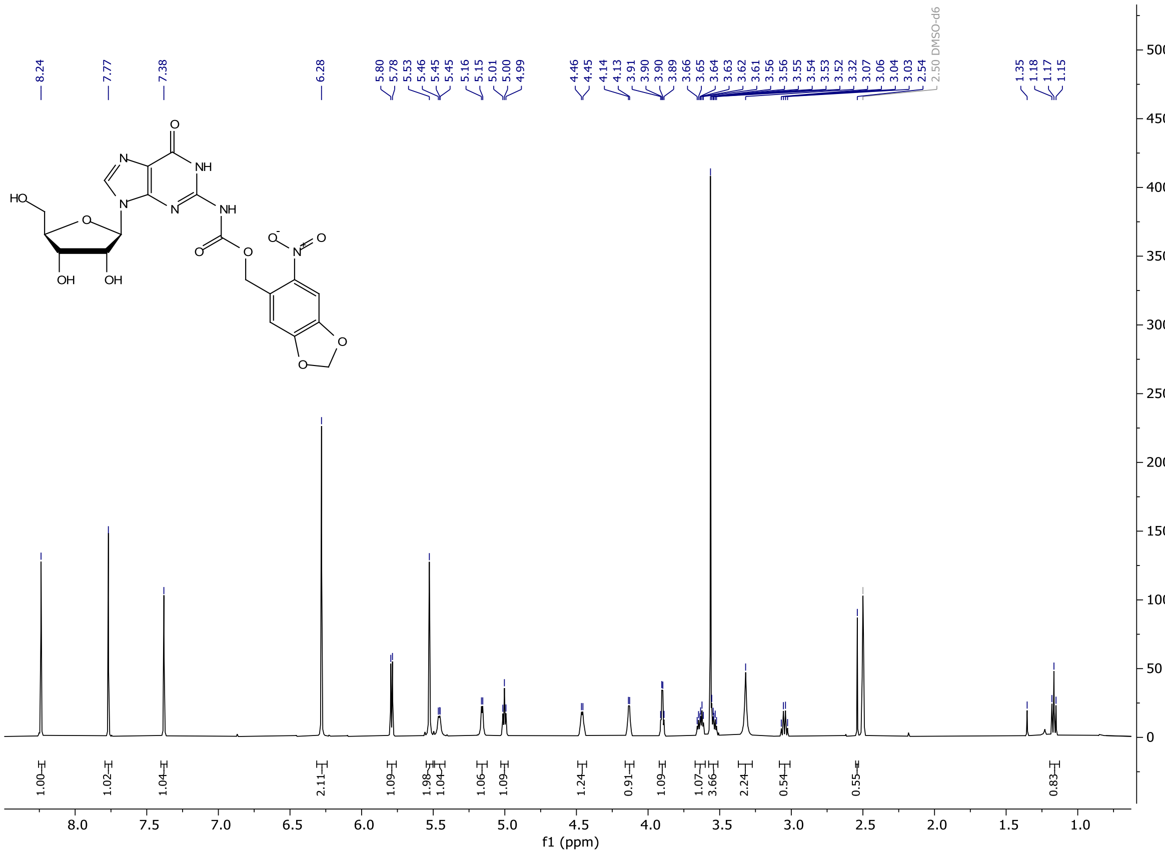

Supplementary Figure 76. ^{1}H-NMR spectrum of compound 5d.

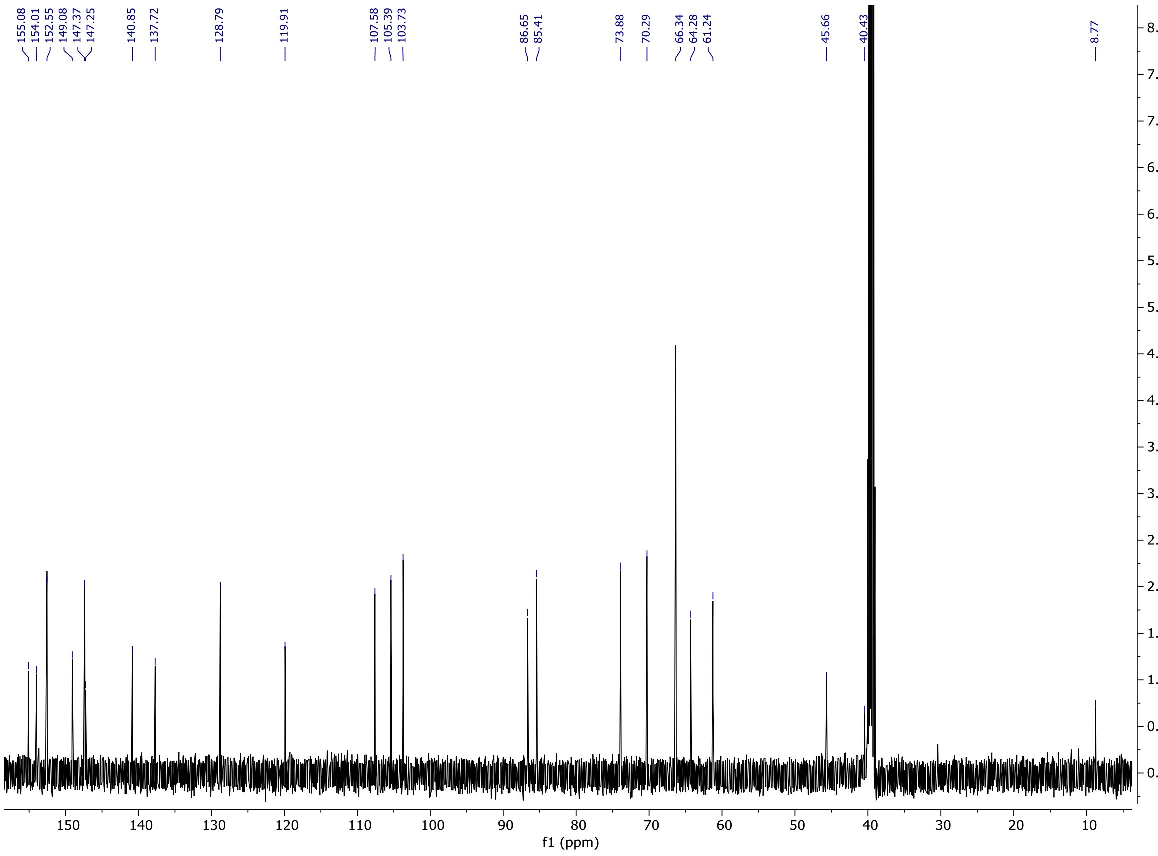

Supplementary Figure 77: ^{13}C-NMR spectrum of compound 5d.

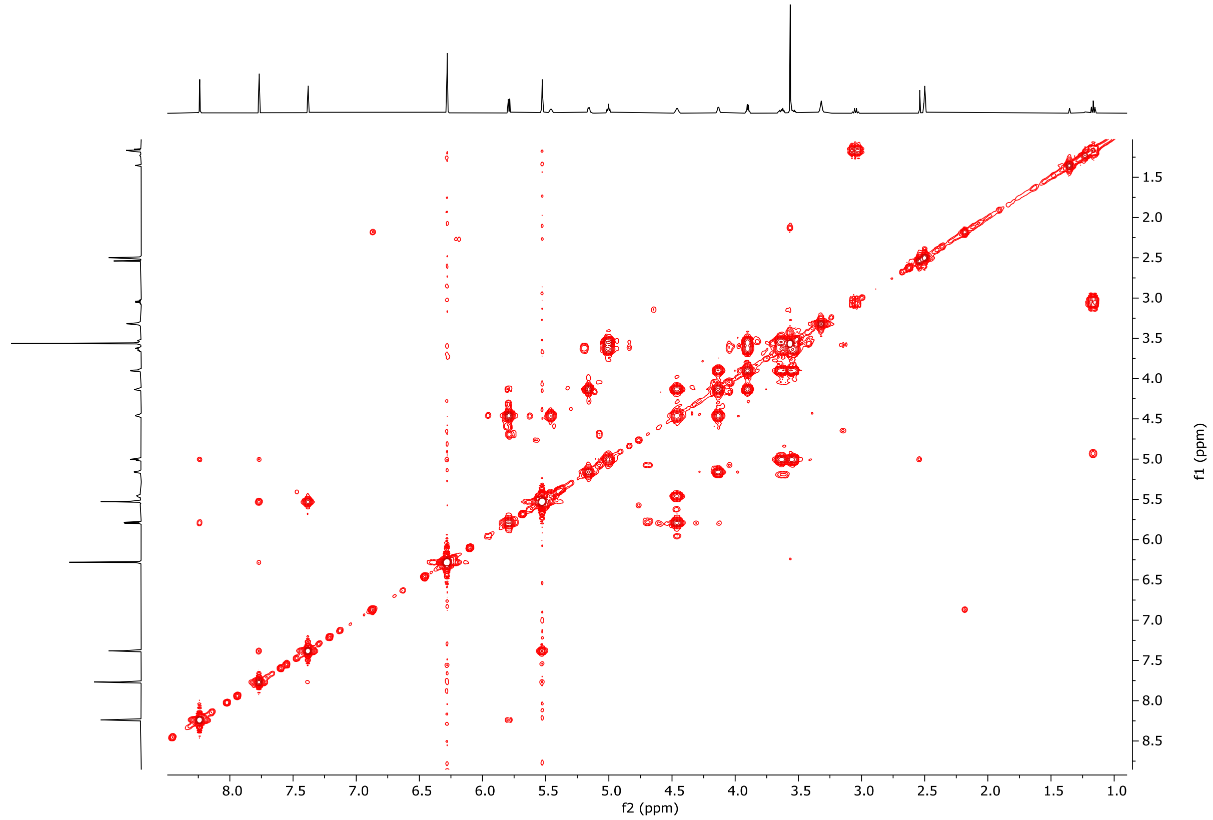

Supplementary Figure 78. COSY spectrum of compound 5d.

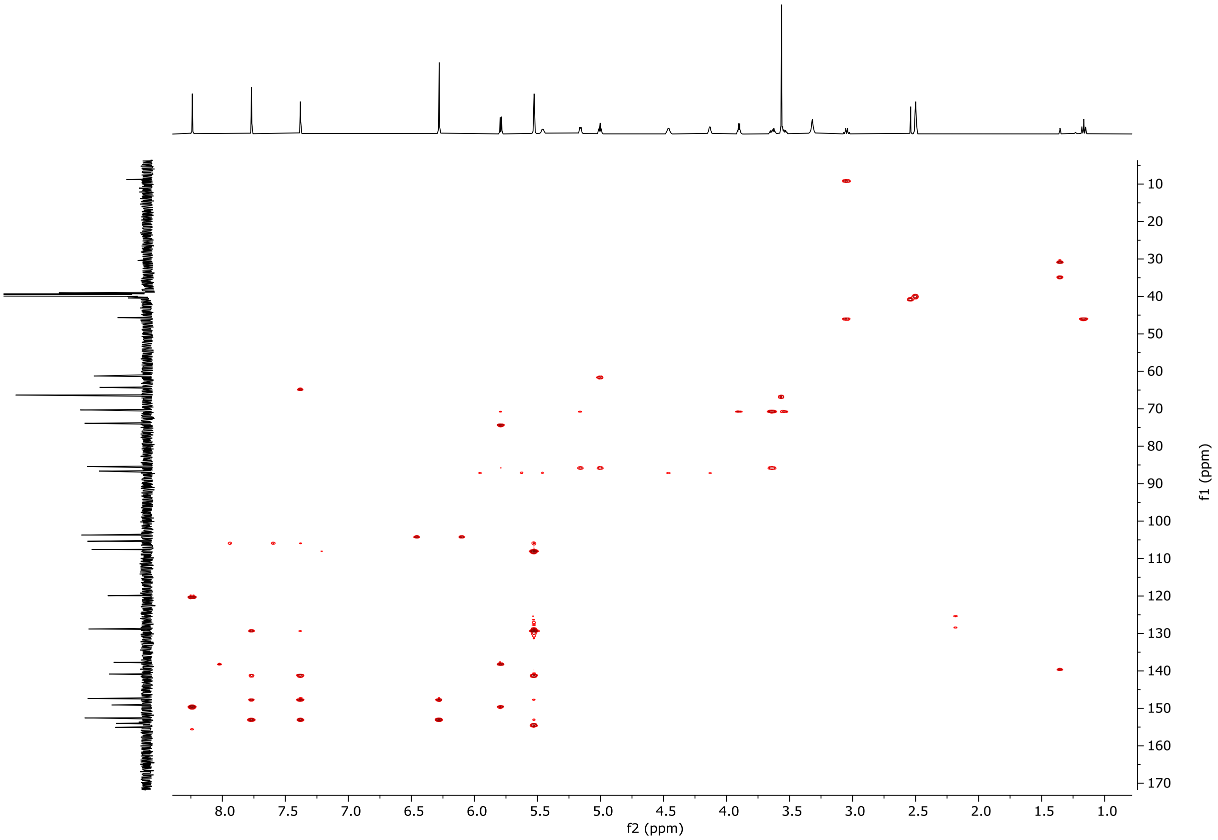

Supplementary Figure 79. HMBC spectrum of compound 5d.

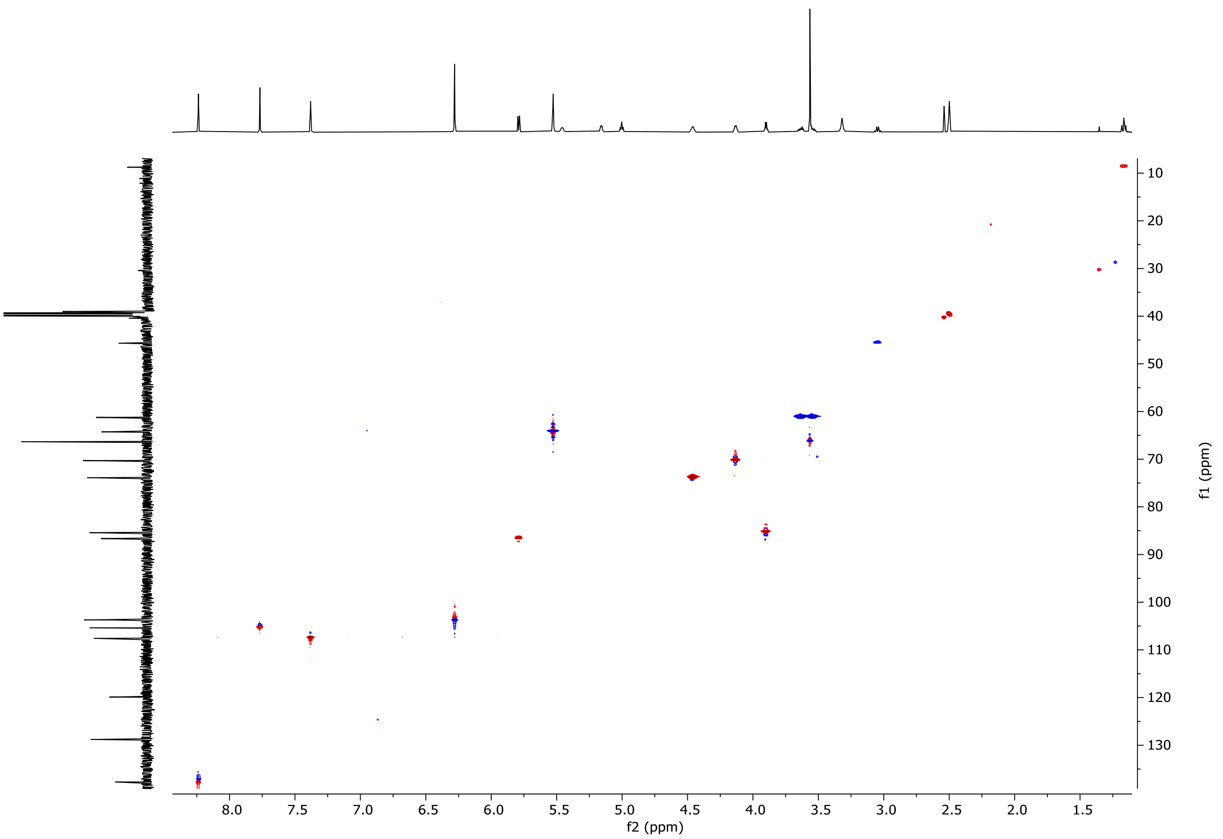

Supplementary Figure 80. HSQC spectrum of compound 5d.

References

1 Anhäuser, L. *et al.* A Benzophenone-Based Photocaging Strategy for the N7 Position of Guanosine. *Angew. Chem., Int. Ed. Engl.* **59**, 3161-3165 (2020).

2 Liu, W. *et al.* Structural basis for nematode eIF4E binding an m(2,2,7)G-Cap and its implications for translation initiation. *Nucleic Acids Res* **39**, 8820-8832 (2011).

3 Arndt, N., Ross-Kaschitza, D., Kojukhov, A., Komar, A. A. & Altmann, M. Properties of the ternary complex formed by yeast eIF4E, p20 and mRNA. *Sci. Rep.* **8**, 6707 (2018).

4 Jemielity, J. *et al.* Novel "anti-reverse" cap analogs with superior translational properties. *RNA* **9**, 1108-1122 (2003).

5 Feng, C., Li, Y. & Spitale, R. C. Photo-controlled cell-specific metabolic labeling of RNA. *Org. Biomol. Chem.* **15**, 5117-5120 (2017).

6 Saunthwal, R. K., Patel, M. & Verma, A. K. Metal- and Protection-Free [4 + 2] Cycloadditions of Alkynes with Azadienes: Assembly of Functionalized Quinolines. *Org. Lett.* **18**, 2200-2203 (2016).
