# Supplementary material for: Photocaged 5′ cap analogues for optical control of mRNA translation in cells
Source: Nat Chem. 2022 Jun 20;14(8):905–13. doi: 10.1038/s41557-022-00972-7 (PMC7613264; doi:10.1038/s41557-022-00972-7)
Supplement: Supplementary file 2 — Reporting Summary [file 41557_2022_972_MOESM2_ESM.pdf]

nature research

Corresponding author(s): Andrea Rentmeister

Last updated by author(s): Apr 1, 2022

Reporting Summary

Nature Research wishes to improve the reproducibility of the work that we publish. This form provides structure for consistency and transparency in reporting. For further information on Nature Research policies, see our Editorial Policies and the Editorial Policy Checklist.

Statistics

For all statistical analyses, confirm that the following items are present in the figure legend, table legend, main text, or Methods section.

n/a	Confirmed	
☐	☒	The exact sample size (*n*) for each experimental group/condition, given as a discrete number and unit of measurement
☐	☒	A statement on whether measurements were taken from distinct samples or whether the same sample was measured repeatedly
☐	☒	The statistical test(s) used AND whether they are one- or two-sided<br>*Only common tests should be described solely by name; describe more complex techniques in the Methods section.*
☒	☐	A description of all covariates tested
☐	☒	A description of any assumptions or corrections, such as tests of normality and adjustment for multiple comparisons
☐	☒	A full description of the statistical parameters including central tendency (e.g. means) or other basic estimates (e.g. regression coefficient) AND variation (e.g. standard deviation) or associated estimates of uncertainty (e.g. confidence intervals)
☐	☒	For null hypothesis testing, the test statistic (e.g. *F*, *t*, *r*) with confidence intervals, effect sizes, degrees of freedom and *P* value noted<br>*Give P values as exact values whenever suitable.*
☒	☐	For Bayesian analysis, information on the choice of priors and Markov chain Monte Carlo settings
☒	☐	For hierarchical and complex designs, identification of the appropriate level for tests and full reporting of outcomes
☒	☐	Estimates of effect sizes (e.g. Cohen's *d*, Pearson's *r*), indicating how they were calculated

Our web collection on statistics for biologists contains articles on many of the points above.

Software and code

Policy information about availability of computer code

Data collection	Leica Application Suite X (3.5.6.21594), CxP Analysis Software, CFX Manager Software V 3.1 (Bio-Rad), Qualitative Navigator B.08.00, Agilent ChemStation for LC 3D systems Rev. B.04.03, Agilent OpenLab CDS ChemStation Edition Rev. C.01.10
Data analysis	Origin (2016b), Origin (2021b), Origin (2019b), GraphPad Prism 7, ImageJ (Version 20160205), MestReNova 14, LasX (Version), Excel 2016, PerkinElmer ChemDraw 20.1.

For manuscripts utilizing custom algorithms or software that are central to the research but not yet described in published literature, software must be made available to editors and reviewers. We strongly encourage code deposition in a community repository (e.g. GitHub). See the Nature Research guidelines for submitting code & software for further information.

Data

Policy information about availability of data

All manuscripts must include a data availability statement. This statement should provide the following information, where applicable:

- Accession codes, unique identifiers, or web links for publicly available datasets
- A list of figures that have associated raw data
- A description of any restrictions on data availability

The data generated or analyzed during this study are included in this article (and its supplementary information files). Source Data for Figs. 3–6 and Extended Data Figures 1-4 are provided with the paper. Protein structures and models used for the figures are available under the accession codes: 1EJ1.

Field-specific reporting

Please select the one below that is the best fit for your research. If you are not sure, read the appropriate sections before making your selection.

☒ Life sciences ☐ Behavioural & social sciences ☐ Ecological, evolutionary & environmental sciences

For a reference copy of the document with all sections, see nature.com/documents/nr-reporting-summary-flat.pdf

Life sciences study design

All studies must disclose on these points even when the disclosure is negative.

Sample size	The entire population of transfected cells were subjected to different assays and analyses. For all experiments, cells were seeded one day prior to transfection. HEK and HeLa cells were seeded as follows for the downstream assays: 200,000 cells were seeded in a 12-well plate, 150,000 cells in a 24-well plate, or 30,000 cells in a 96-well plate, respectively. Based on the doubling time of the different cell lines, an estimation of 800,000 cells per condition were harvested for microscopy, flow cytometry, or Western Blot analysis. For RTqPCR 400,000 cells/well were harvested, however two wells per condition were used, resulting again in a total cell number of 800,000 cells. For luminescence and MTT assays in a 96-well plate, an estimation of 120,000 cells were harvested. For the immunogenicity assay an estimation of 600,000 cells were harvested.
Data exclusions	In general, no data was excluded. For MST measurements, outliers were excluded according to the criteria stated in the supplementary information. MST is known to produce occasional outliers. This was handled as follows: 16 data points were measured per binding curve and at least 12 data points were used for each fit.
Replication	All experiments were performed in at least three independent experiments. For the RTqPCR and Luminescence Data, three technical replicates were performed for each independent experiment. For the MTT assay two technical replicates were performed for each independent experiment. All independent experiments and technical replicates showed similar results.
Randomization	Randomization of the experiments in this study was not required, as we measured physical values, i.e. the Luminescence/ Fluorescence/ UV absorption, which is not influenced by the observer. Therefore we deemed independent biological replicates to be sufficient.
Blinding	The persons performing microscopy was unaware of the sample identity. Additional blinding was not possible as experimental conditions (irradiation) were evident from the image data/Luminescence data

Reporting for specific materials, systems and methods

We require information from authors about some types of materials, experimental systems and methods used in many studies. Here, indicate whether each material, system or method listed is relevant to your study. If you are not sure if a list item applies to your research, read the appropriate section before selecting a response.

Materials & experimental systems

n/a	Involved in the study	
☐	☒	Antibodies
☐	☒	Eukaryotic cell lines
☒	☐	Palaeontology and archaeology
☒	☐	Animals and other organisms
☒	☐	Human research participants
☒	☐	Clinical data
☒	☐	Dual use research of concern

Methods

n/a	Involved in the study	
☒	☐	ChIP-seq
☐	☒	Flow cytometry
☒	☐	MRI-based neuroimaging

Antibodies

Antibodies used	Primary antibodies: anti-eGFP mouse monoclonal antibody (Santa Cruz Biotechnology) and anti-Nucleolin mouse monoclonal antibody (Thermo Fisher Scientific)<br>Secondary Antibody: HRP-conjugated secondary antibody (polyclonal rabbit anti-mouse immunoglobulins/HRP; Dako Diagnostica GmbH)
Validation	The Nucleolin Antibody was verified by Knockdown to ensure that the antibody binds to the antigen stated (information gained from the suppliers webpage). The eGFP antibody is a commonly used antibody, which was additionally validated in our study by transfected (eGFP positive) and untransfected (eGFP negative) samples.

Eukaryotic cell lines

Policy information about cell lines

Cell line source(s)	HeLa Cells (Merck), HEK 293T cells (DSMZ), HEK-NF-κB cells (TRON)
Authentication	HeLa cells were authenticated by Merck, HEK293T cells were authenticated by DSMZ-German collection of microorganisms and cell cultures GmBH, HEK-NF-κB cells were authenticated by TRON GmBH (Mainz).
Mycoplasma contamination	All cells were tested negative for Mycoplasma contamination.
Commonly misidentified lines (See ICLAC register)	No commonly misidentified cell lines were used in this study.

Flow Cytometry

Plots

Confirm that:

☒ The axis labels state the marker and fluorochrome used (e.g. CD4-FITC).

☒ The axis scales are clearly visible. Include numbers along axes only for bottom left plot of group (a 'group' is an analysis of identical markers).

☒ All plots are contour plots with outliers or pseudocolor plots.

☒ A numerical value for number of cells or percentage (with statistics) is provided.

Methodology

Sample preparation	One day prior to transfection, 2×10^5 HeLa cells were seeded in a 12-well plate in 1 mL media. Cells were transfected using 1.5 µL Lipofectamine™ MessengerMAX™ (Invitrogen) in Opti-MEM (48.5 µL) and 1 µg eGFP mRNA in Opti-MEM (50 µL). The cells were incubated with the mRNA/ Lipofectamine™ MessengerMAX™ mixture for 4 h at 37 °C in a total volume of 1 mL. The samples were irradiated with 365 nm for 30 sec. Subsequently, the cell media with the transfection agent was replaced with fresh media and the cells were incubated overnight at 37 °C. At 24 h post transfection the cells were harvested with trypsin/EDTA and washed with PBS. The cell suspension was filtered through a 40 µm filter to avoid cell clumps.
Instrument	Beckman Coulter Cytomics FC 500
Software	CxP Analysis Software
Cell population abundance	The entire population of transfected cells were subjected to flow cytometry (about 800.000 cells per sample). During flow cytometry measurement, 10,000 cells of this population were measured per sample.
Gating strategy	The entire population of transfected cells were subjected to flow cytometry. No previous gating of the cells was performed. Untransfected cells were used as negative control (<1% positive cells). The gate for positive cells can be seen in the Figure 5c. Therefore no figure exemplifying the gating strategy is provided.

☐ Tick this box to confirm that a figure exemplifying the gating strategy is provided in the Supplementary Information.
