# Supplementary figures and images for: Photocaged 5′ cap analogues for optical control of mRNA translation in cells
Source: Nat Chem. 2022 Jun 20;14(8):905–13. doi: 10.1038/s41557-022-00972-7 (PMC7613264; doi:10.1038/s41557-022-00972-7)

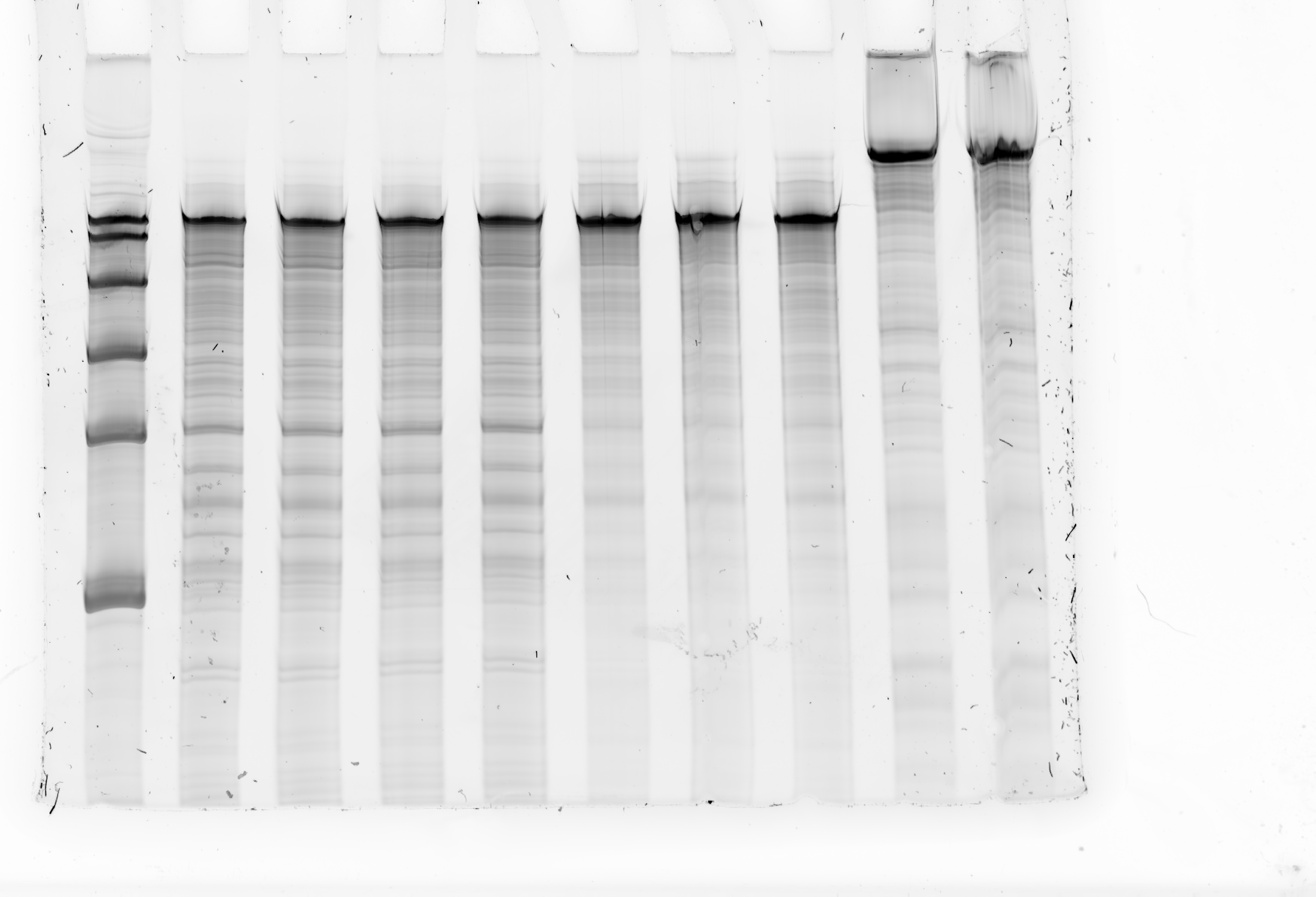

Supplement: Source Data Fig. 4 — Statistical source data and unprocessed gels [file 41557_2022_972_MOESM4_ESM.zip › Fig4_a_irradiated_mRNA.tif]

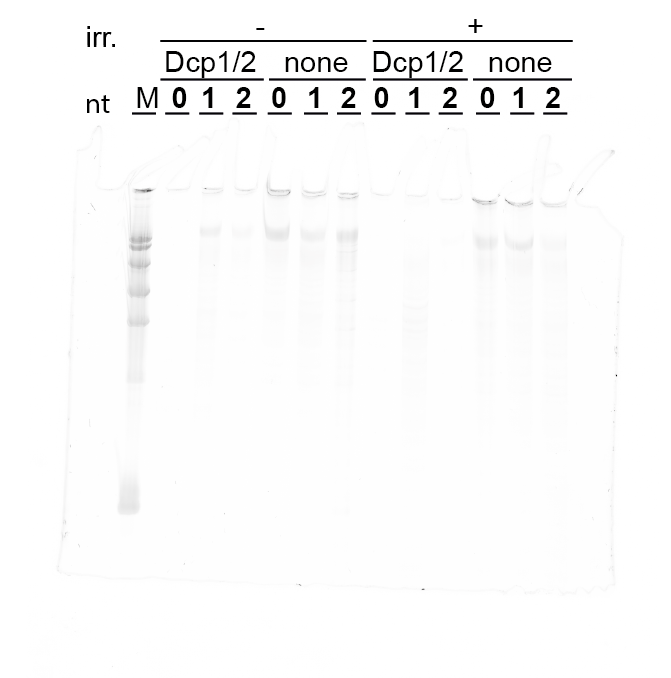

Supplement: Source Data Fig. 4 — Statistical source data and unprocessed gels [file 41557_2022_972_MOESM4_ESM.zip › Uncropped and unprocessed DCP1-2 Digest-01.tif]

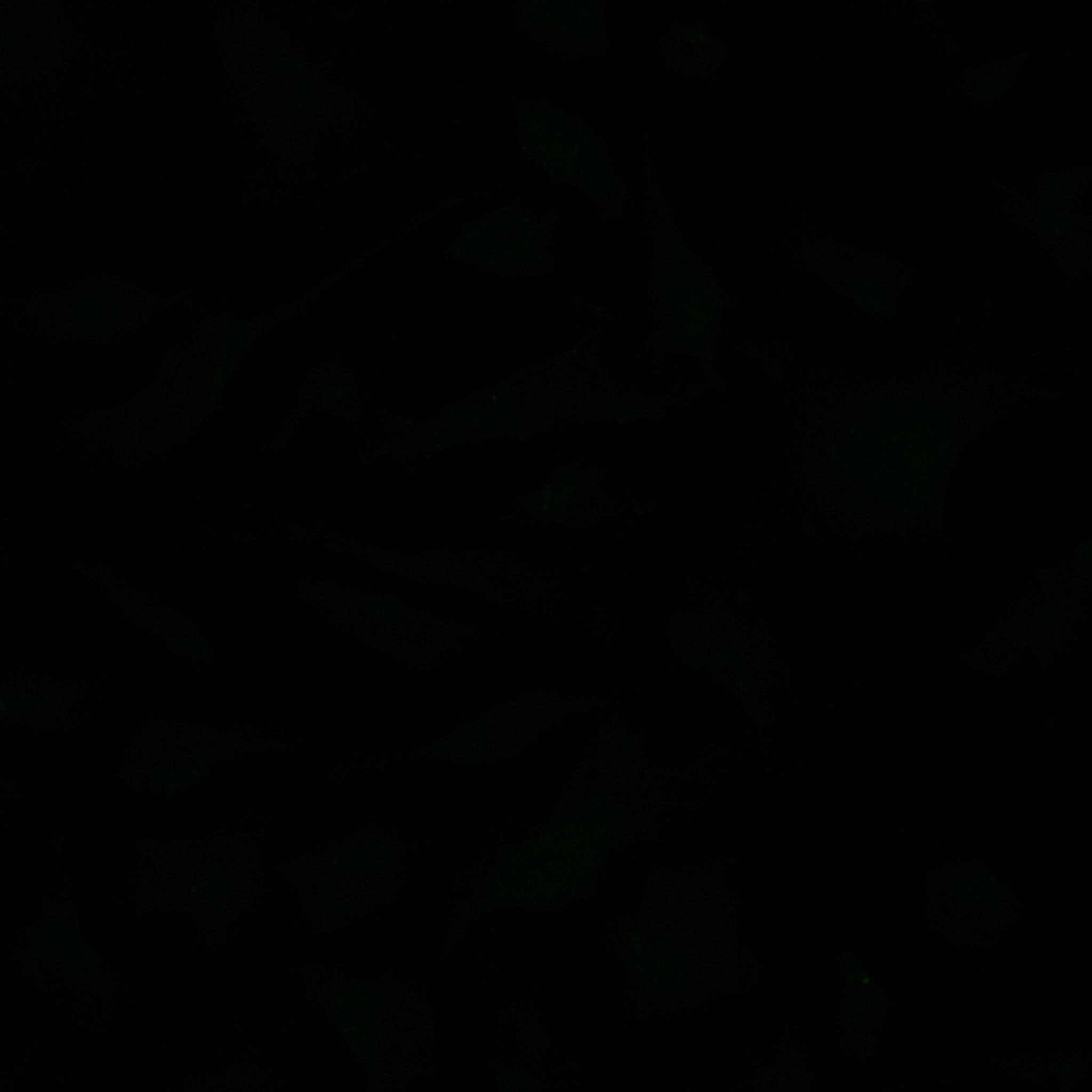

Supplement: Source Data Fig. 5 — Statistical source data and microscopy images [file 41557_2022_972_MOESM5_ESM.zip › Fig5b_ApppG_eGFP.jpg]

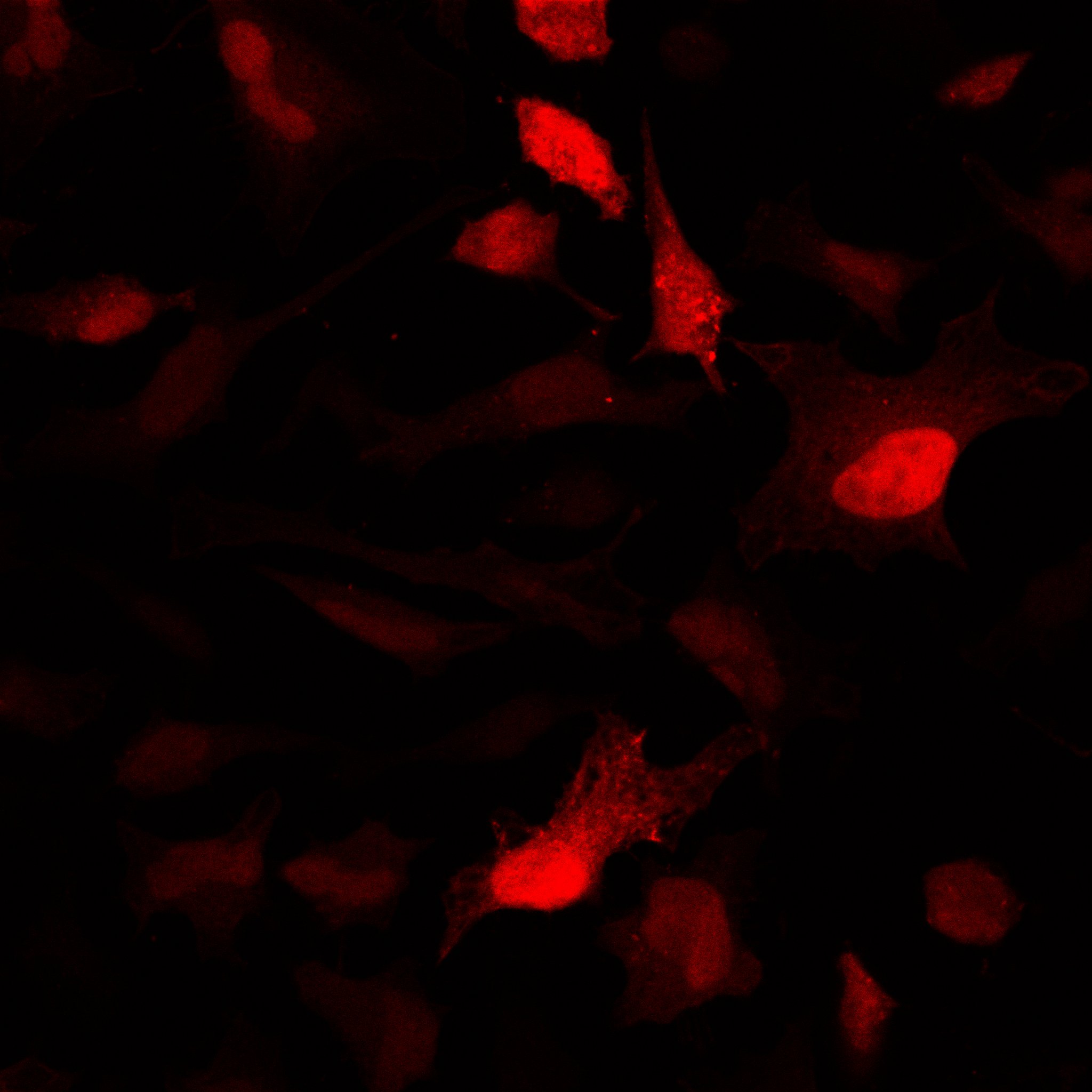

Supplement: Source Data Fig. 5 — Statistical source data and microscopy images [file 41557_2022_972_MOESM5_ESM.zip › Fig5b_ApppG_mScarlet.jpg]

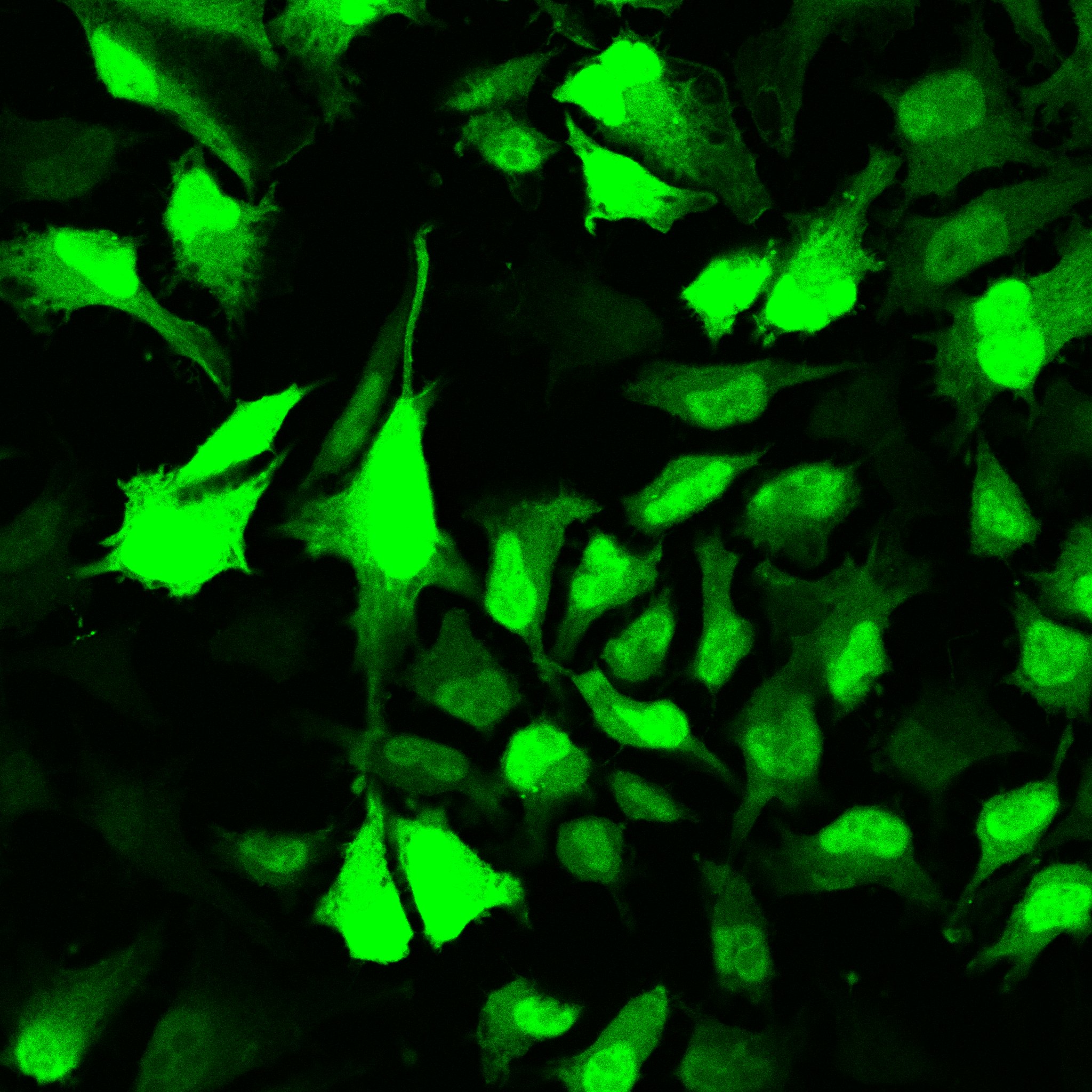

Supplement: Source Data Fig. 5 — Statistical source data and microscopy images [file 41557_2022_972_MOESM5_ESM.zip › Fig5b_m7GpppG_eGFP.jpg]

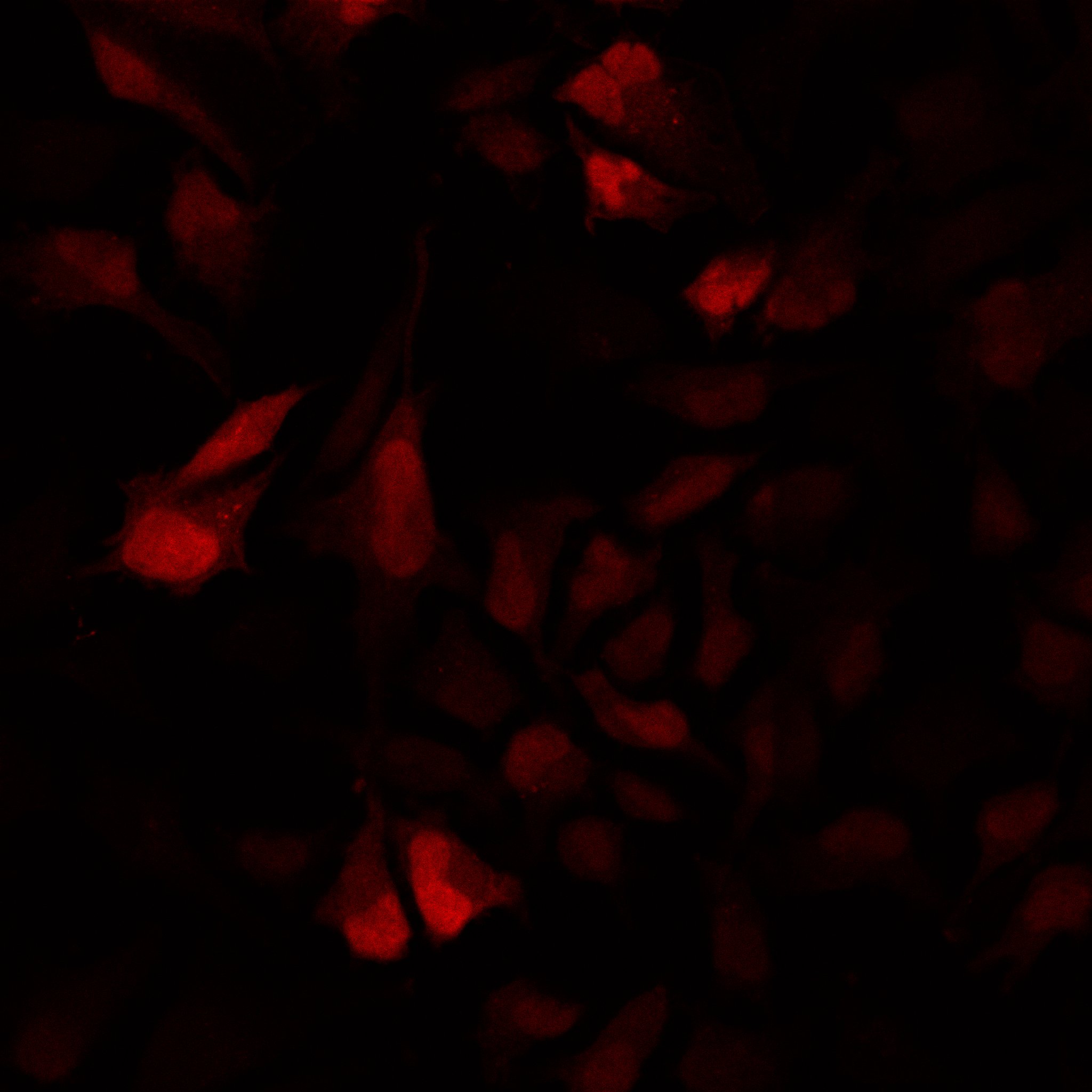

Supplement: Source Data Fig. 5 — Statistical source data and microscopy images [file 41557_2022_972_MOESM5_ESM.zip › Fig5b_m7GpppG_mScarlet.jpg]

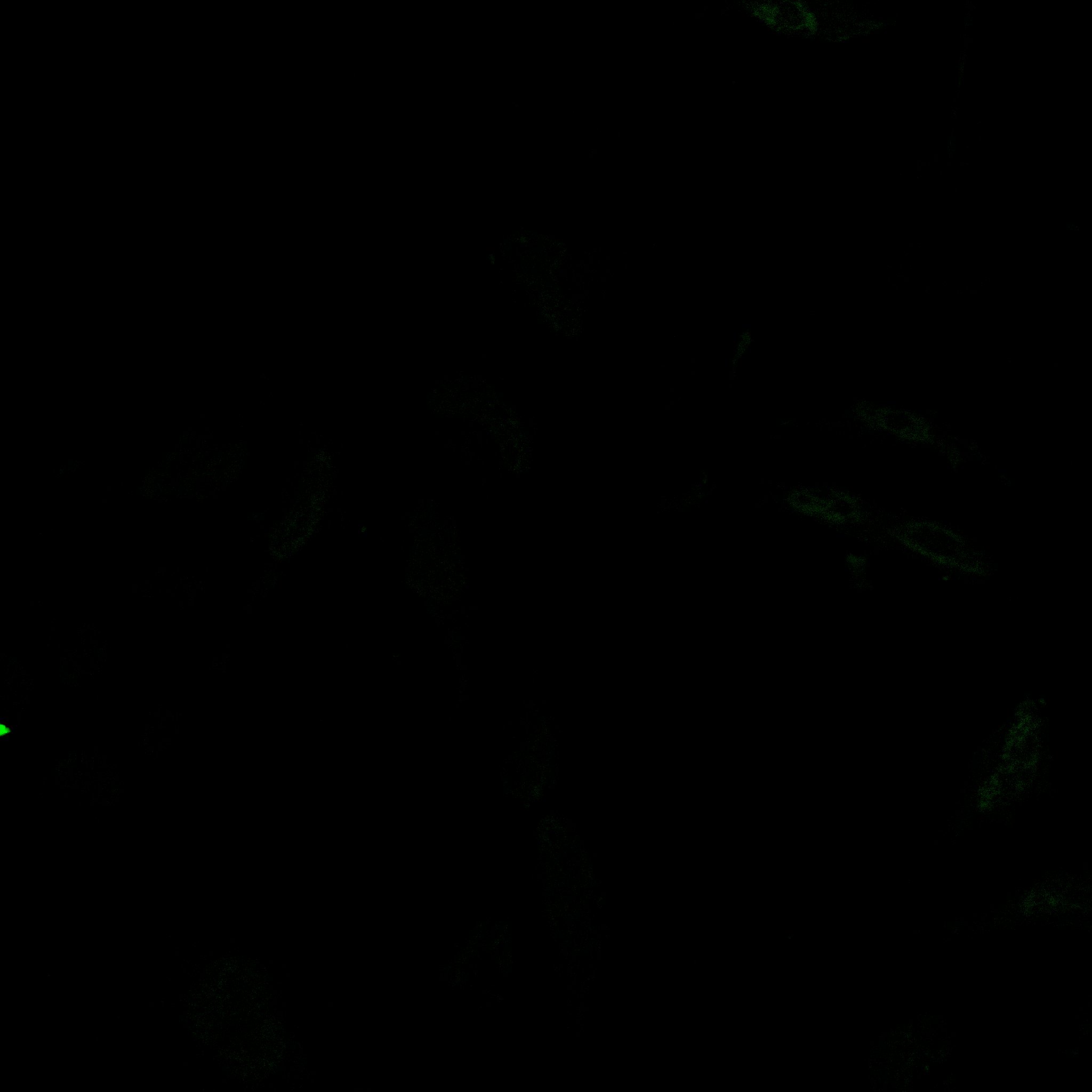

Supplement: Source Data Fig. 5 — Statistical source data and microscopy images [file 41557_2022_972_MOESM5_ESM.zip › Fig5b_NPM_eGFP.jpg]

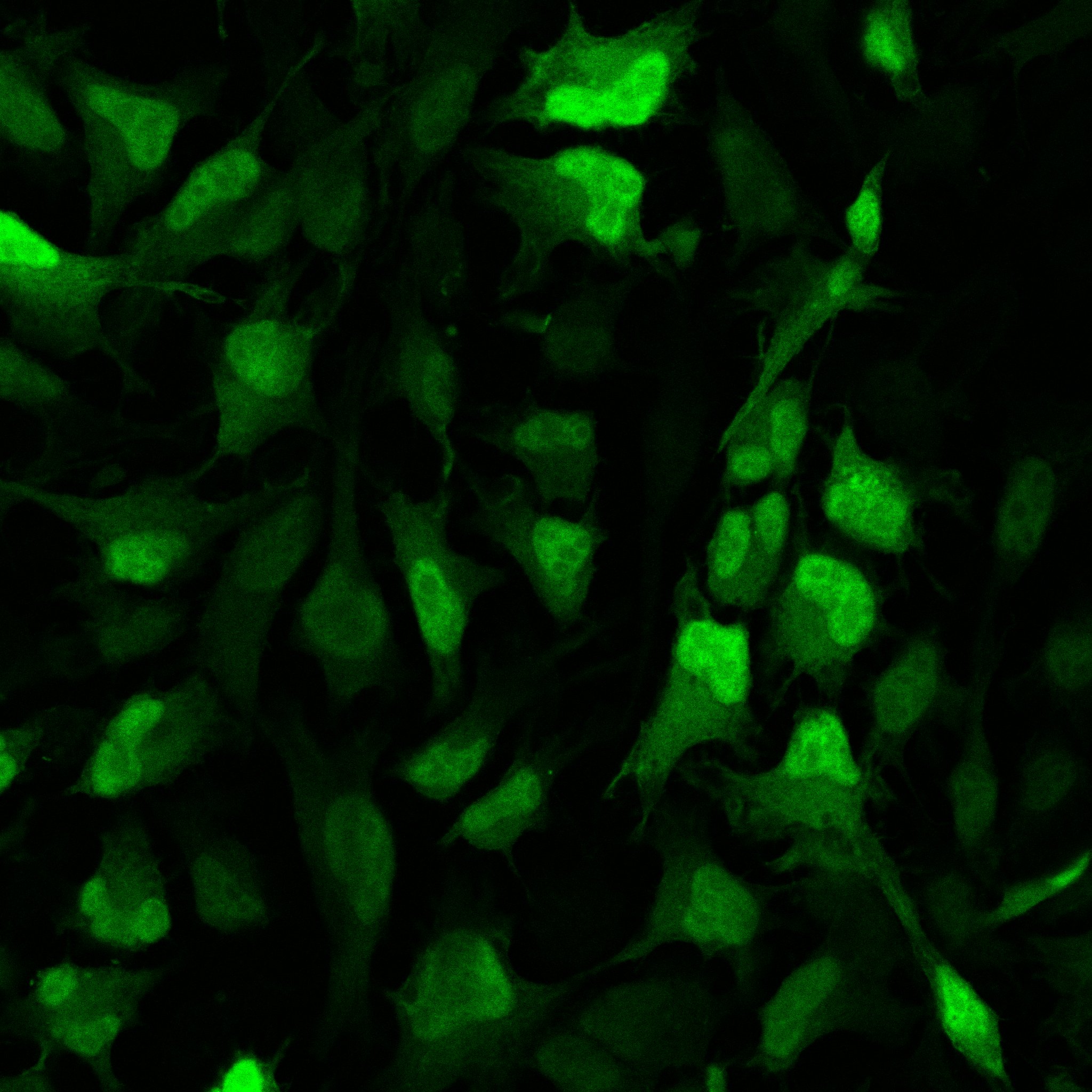

Supplement: Source Data Fig. 5 — Statistical source data and microscopy images [file 41557_2022_972_MOESM5_ESM.zip › Fig5b_NPM_irradiated_in_cells_eGFP.jpg]

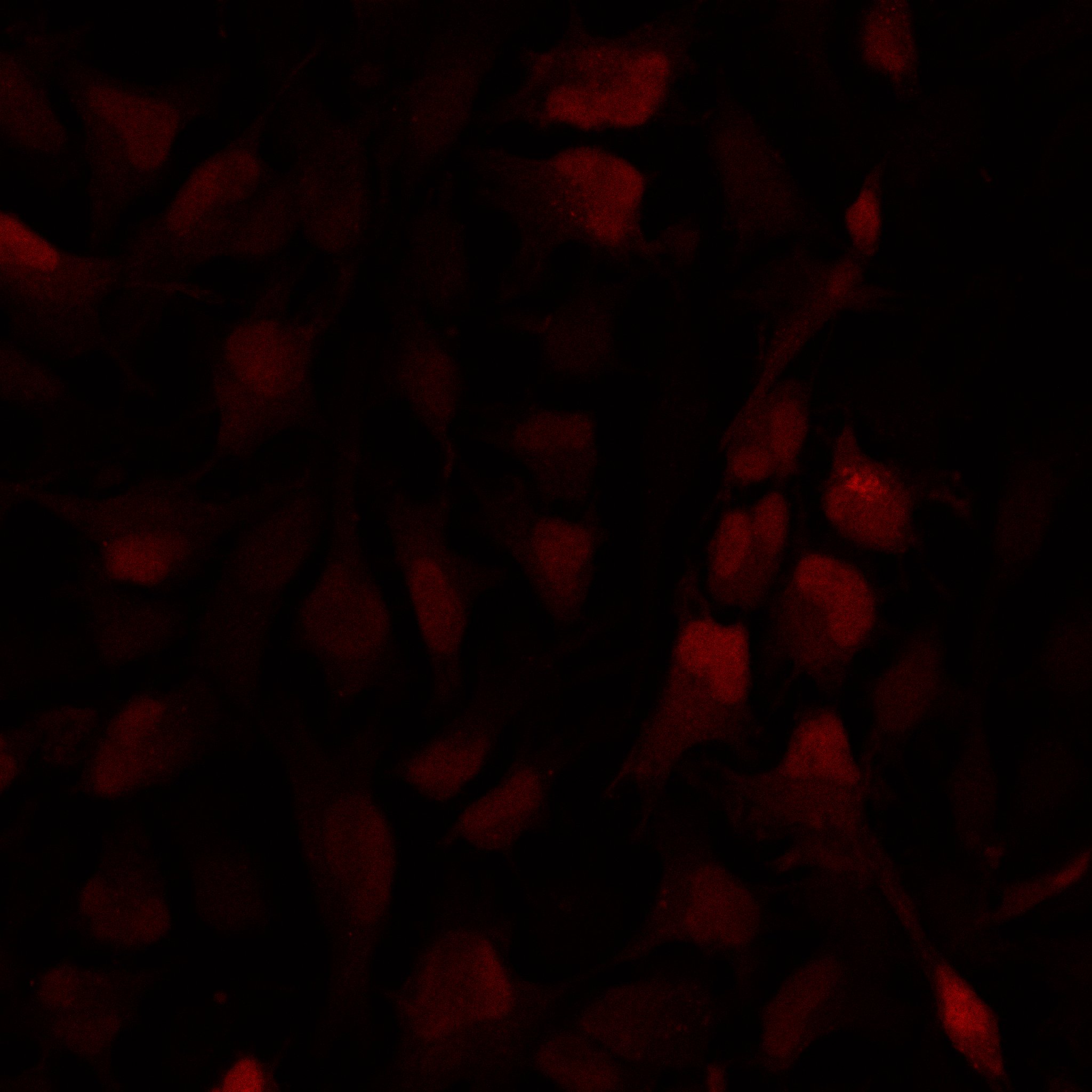

Supplement: Source Data Fig. 5 — Statistical source data and microscopy images [file 41557_2022_972_MOESM5_ESM.zip › Fig5b_NPM_irradiated_in_cells_mScarlet.jpg]

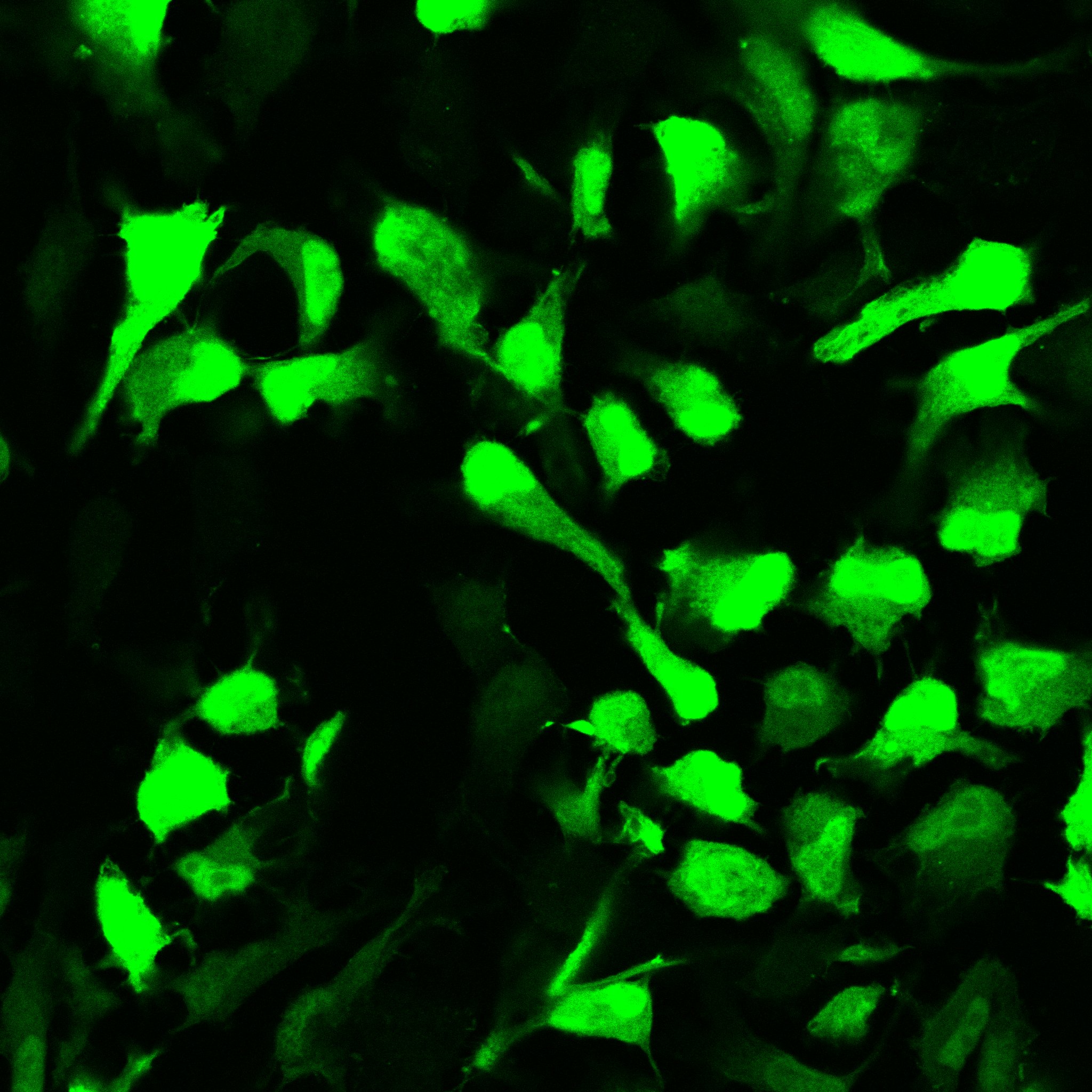

Supplement: Source Data Fig. 5 — Statistical source data and microscopy images [file 41557_2022_972_MOESM5_ESM.zip › Fig5b_NPM_irradiated_mRNA_eGFP.jpg]

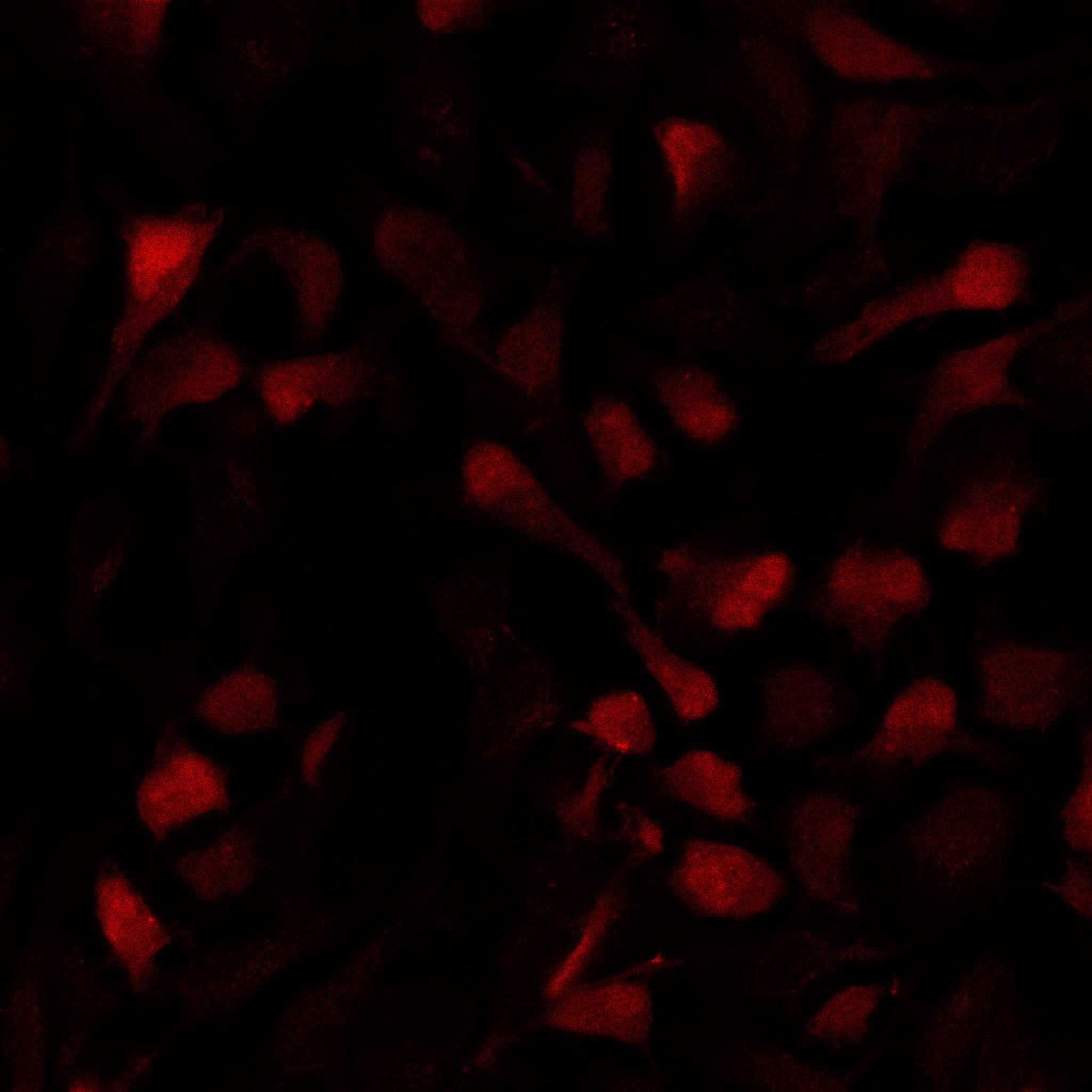

Supplement: Source Data Fig. 5 — Statistical source data and microscopy images [file 41557_2022_972_MOESM5_ESM.zip › Fig5b_NPM_irradiated_mRNA_mScarlet.jpg]

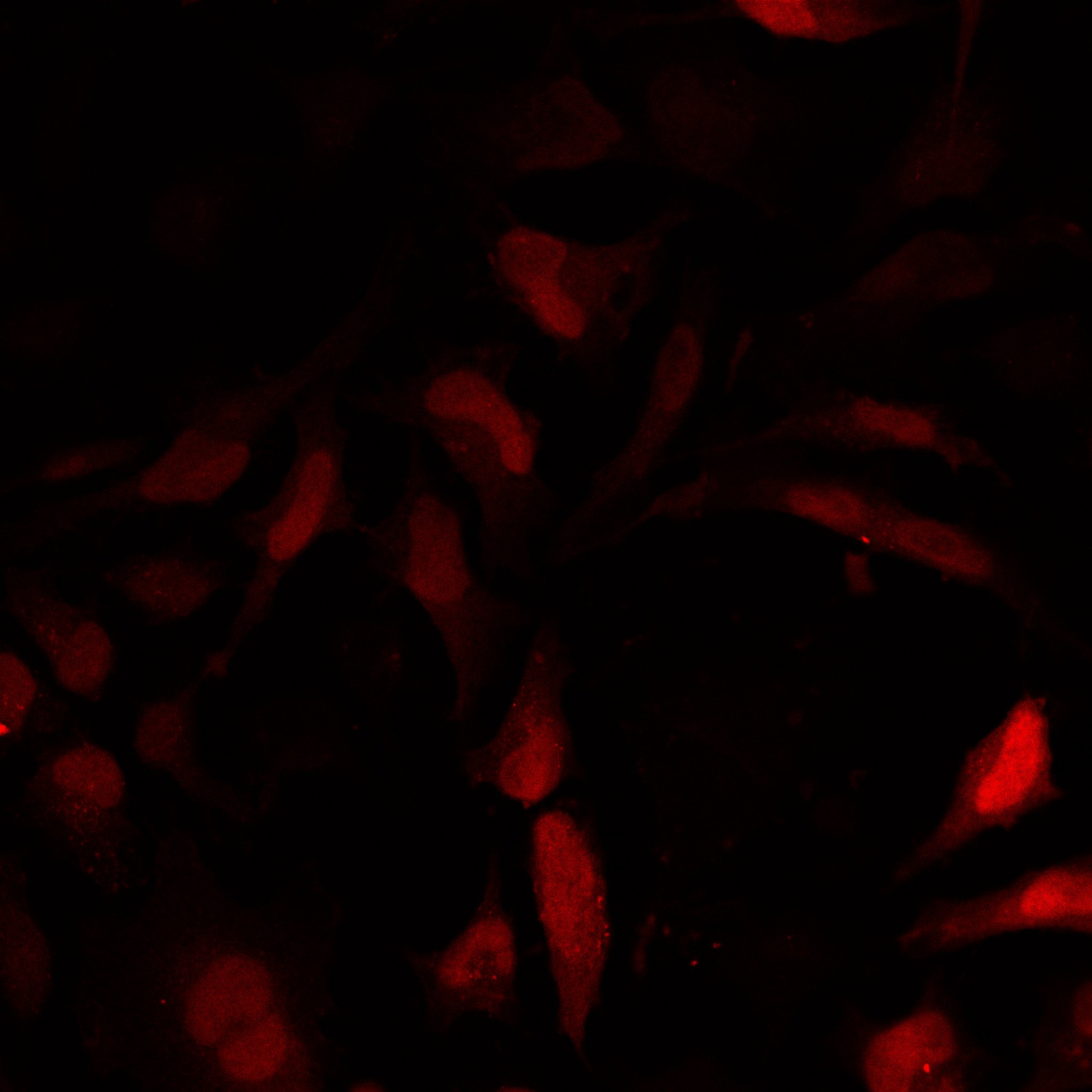

Supplement: Source Data Fig. 5 — Statistical source data and microscopy images [file 41557_2022_972_MOESM5_ESM.zip › Fig5b_NPM_mScarlet.jpg]

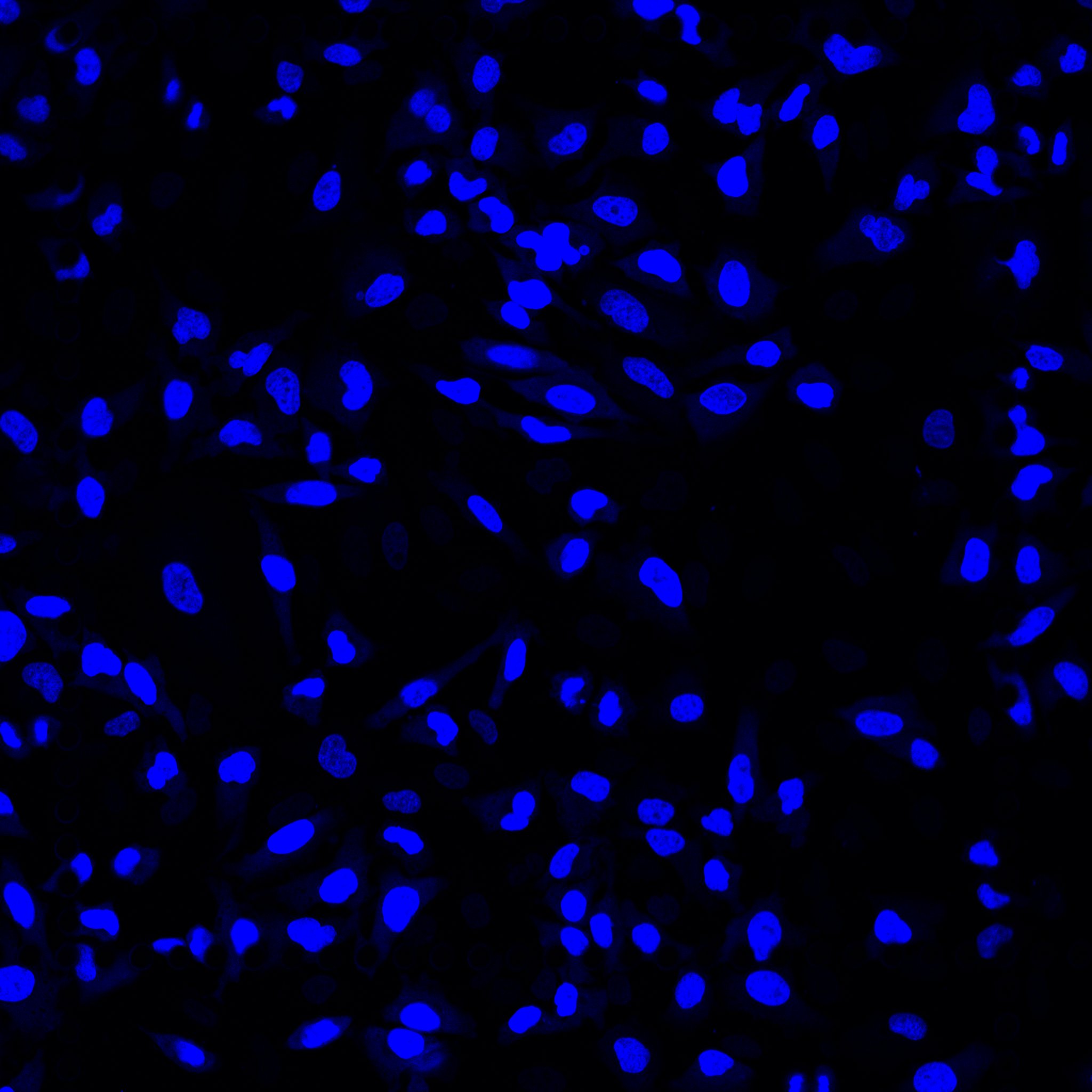

Supplement: Source Data Fig. 6 — Statistical source data and microscopy images [file 41557_2022_972_MOESM6_ESM.zip › Fig6a_DAPI.jpg]

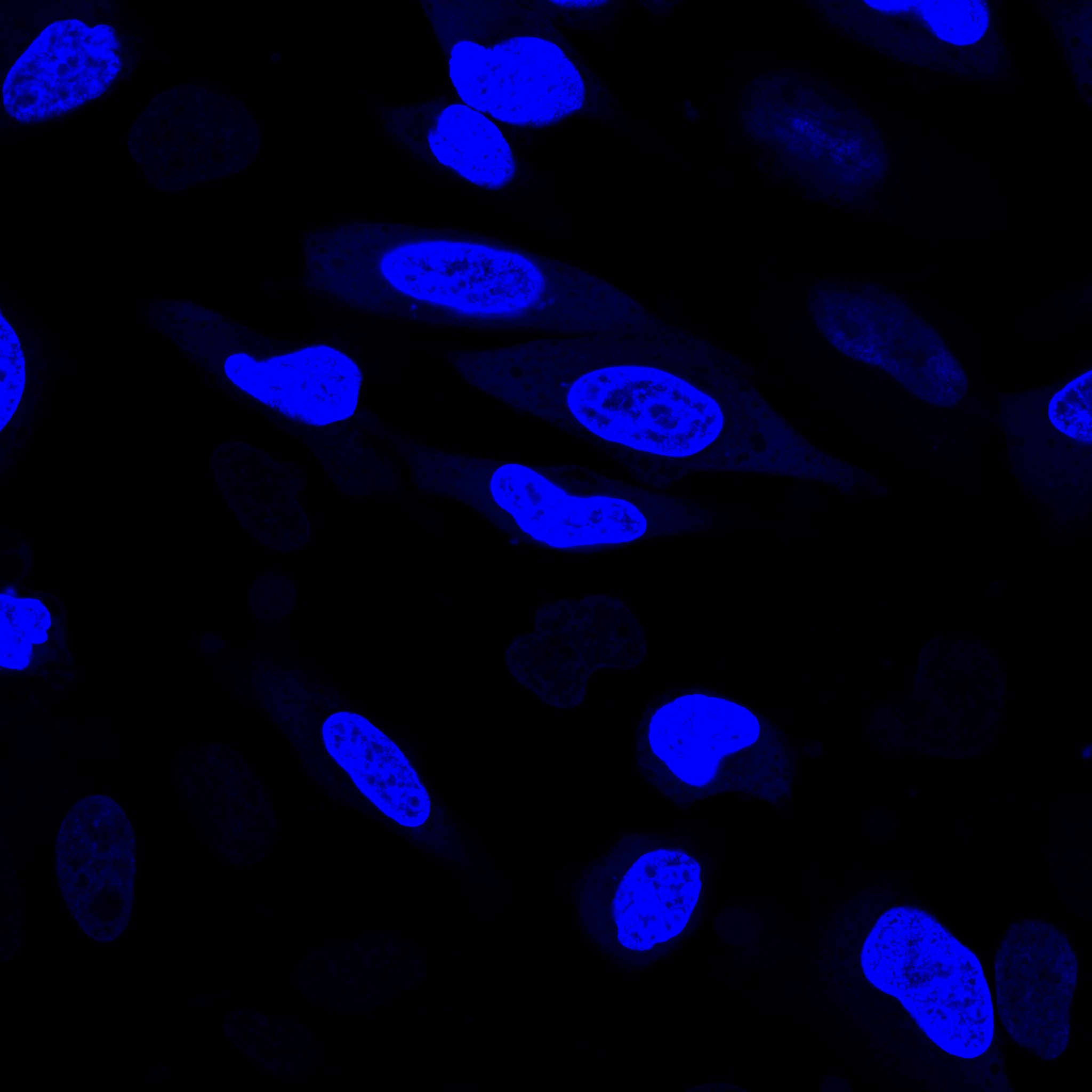

Supplement: Source Data Fig. 6 — Statistical source data and microscopy images [file 41557_2022_972_MOESM6_ESM.zip › Fig6a_DAPI_zoom.jpg]

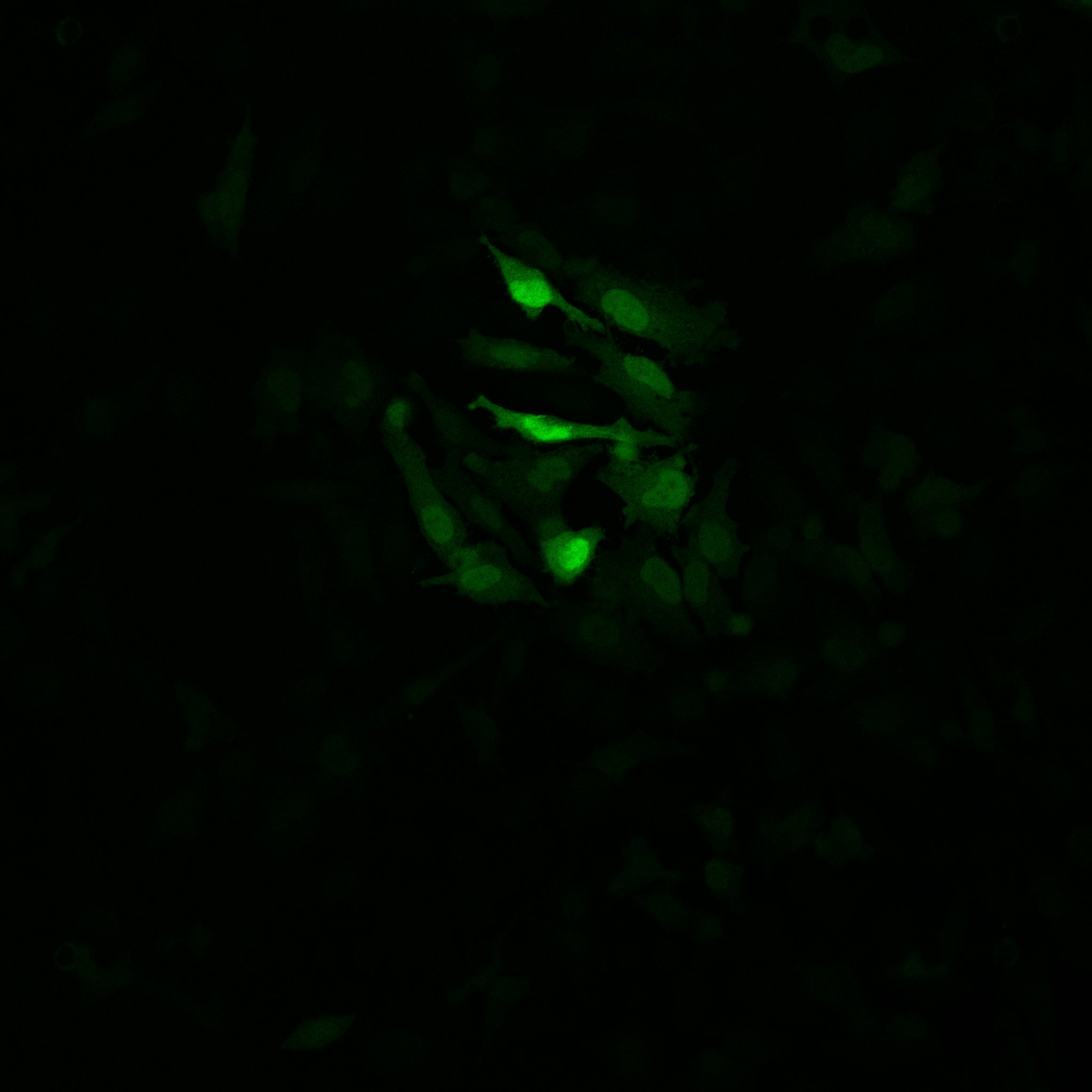

Supplement: Source Data Fig. 6 — Statistical source data and microscopy images [file 41557_2022_972_MOESM6_ESM.zip › Fig6a_eGFP.jpg]

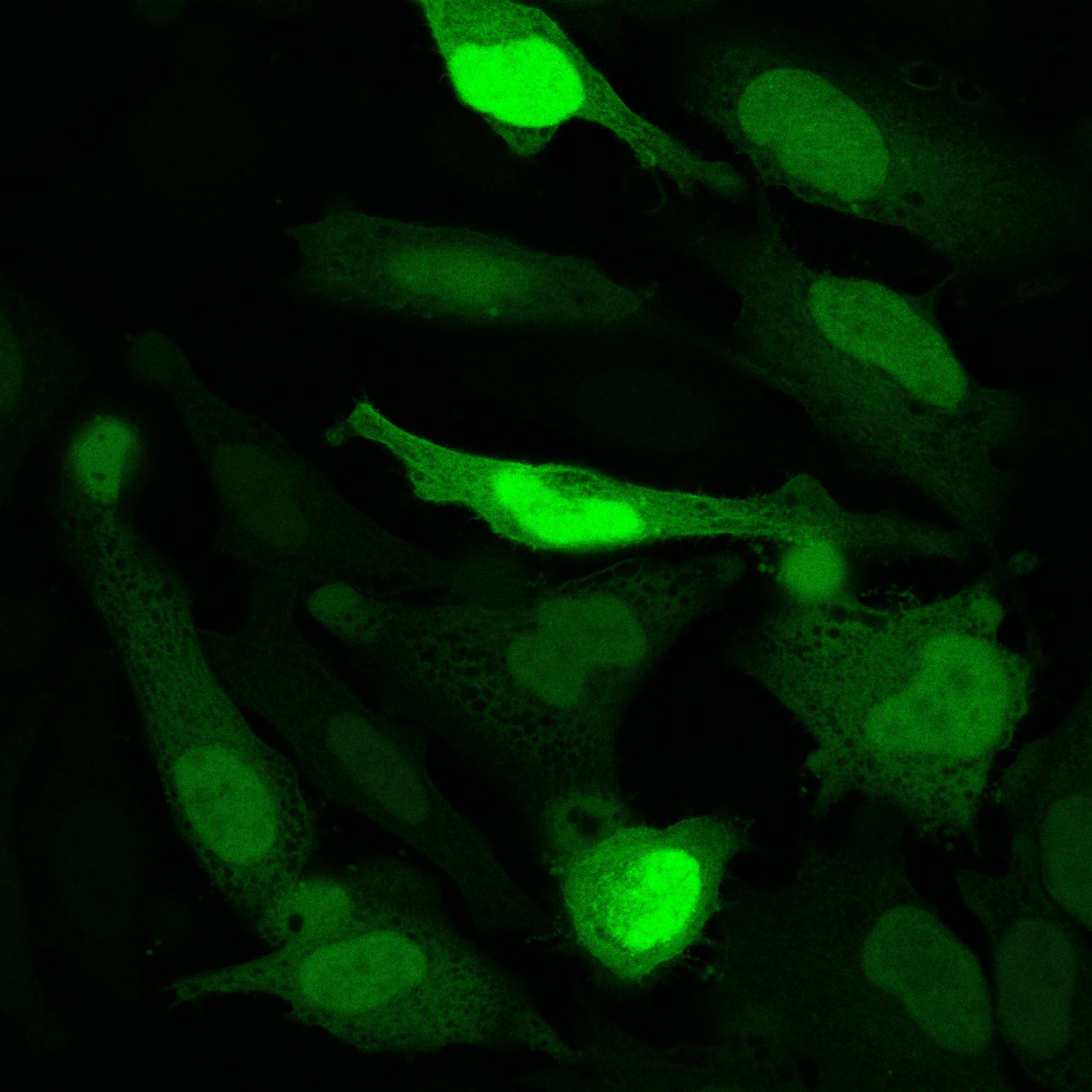

Supplement: Source Data Fig. 6 — Statistical source data and microscopy images [file 41557_2022_972_MOESM6_ESM.zip › Fig6a_eGFP_zoom.jpg]

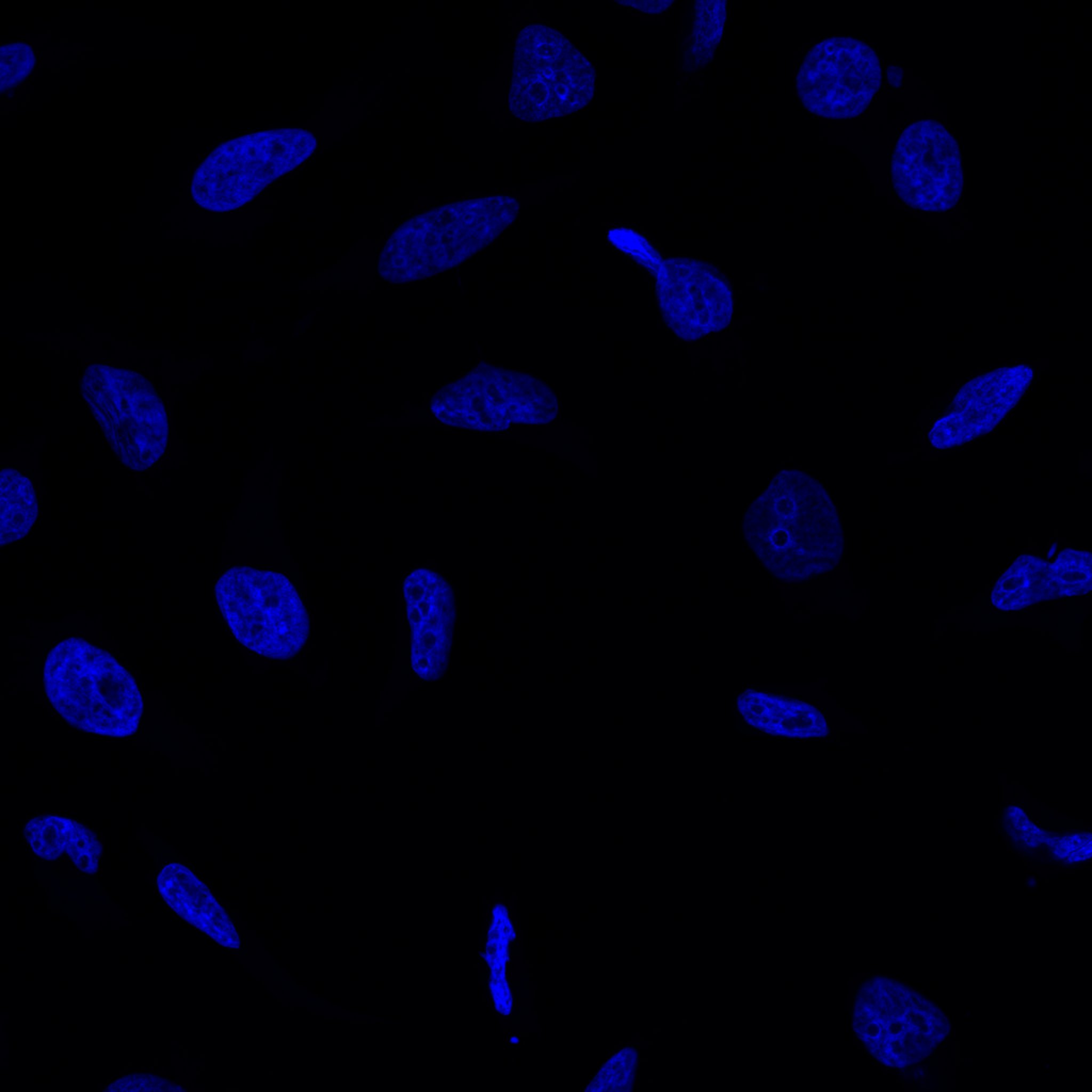

Supplement: Source Data Extended Data Fig. 3 — Microscopy images [file 41557_2022_972_MOESM9_ESM.zip › EDFig3_ApppG_DAPI.jpg]

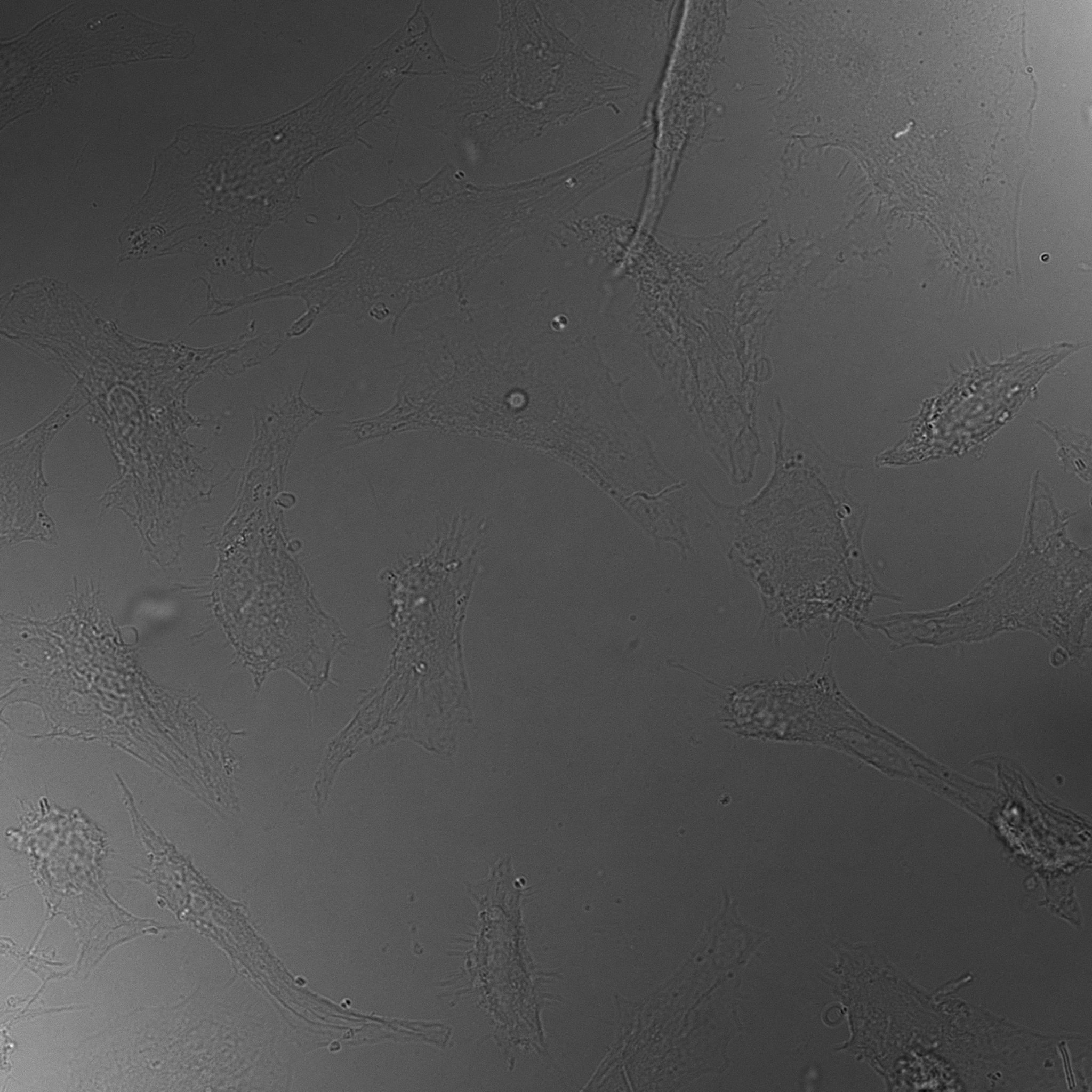

Supplement: Source Data Extended Data Fig. 3 — Microscopy images [file 41557_2022_972_MOESM9_ESM.zip › EDFig3_ApppG_DIC.jpg]

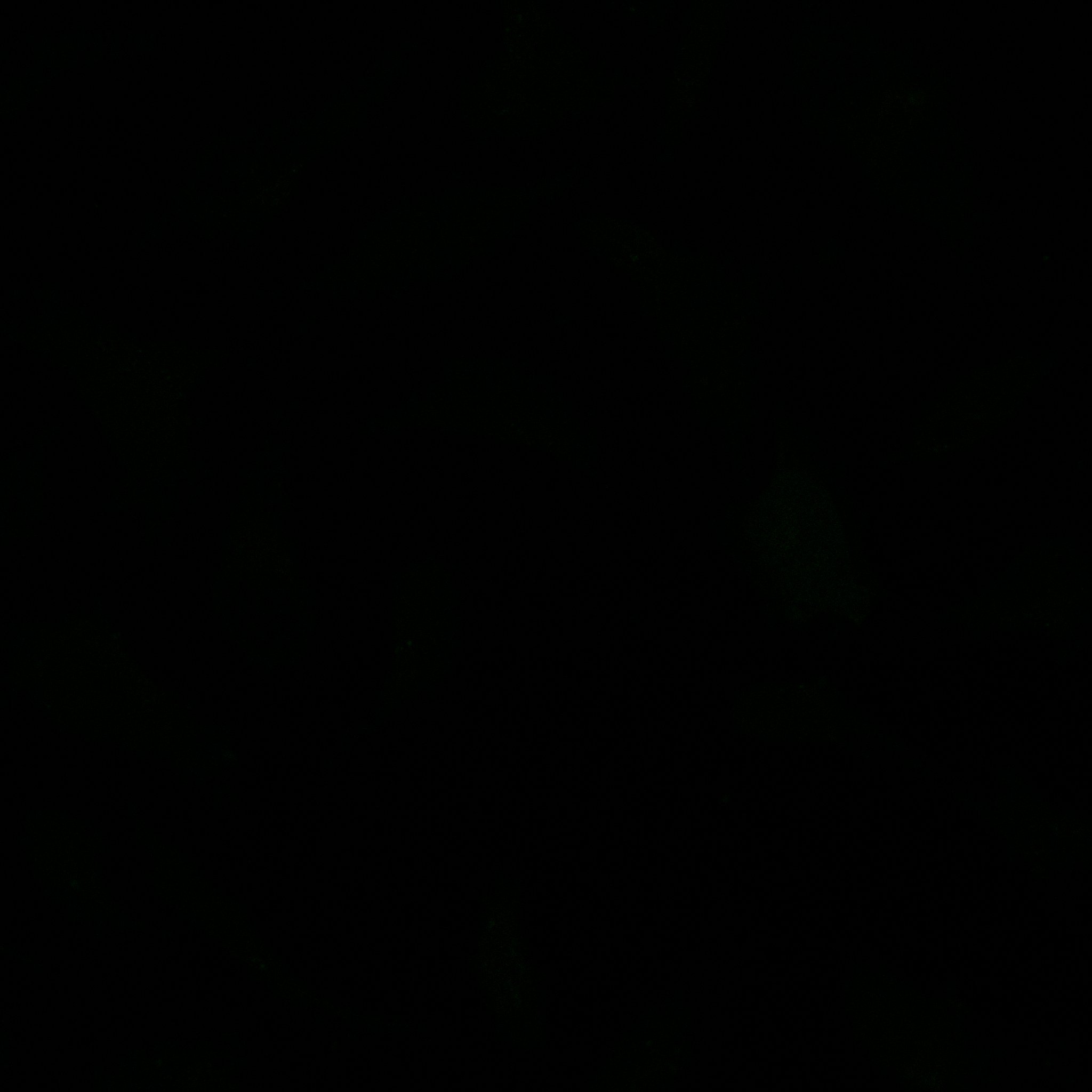

Supplement: Source Data Extended Data Fig. 3 — Microscopy images [file 41557_2022_972_MOESM9_ESM.zip › EDFig3_ApppG_eGFP.jpg]

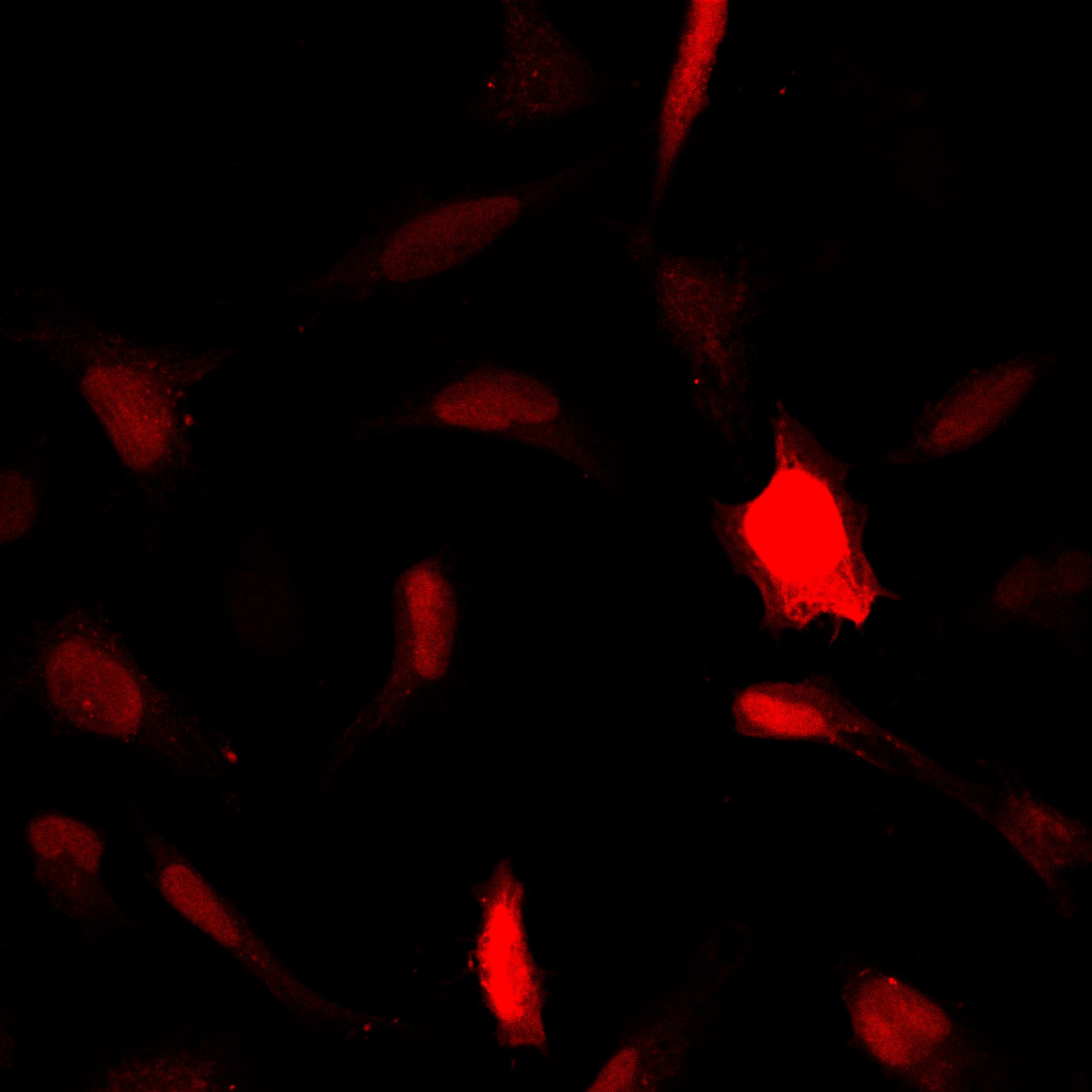

Supplement: Source Data Extended Data Fig. 3 — Microscopy images [file 41557_2022_972_MOESM9_ESM.zip › EDFig3_ApppG_scar.jpg]

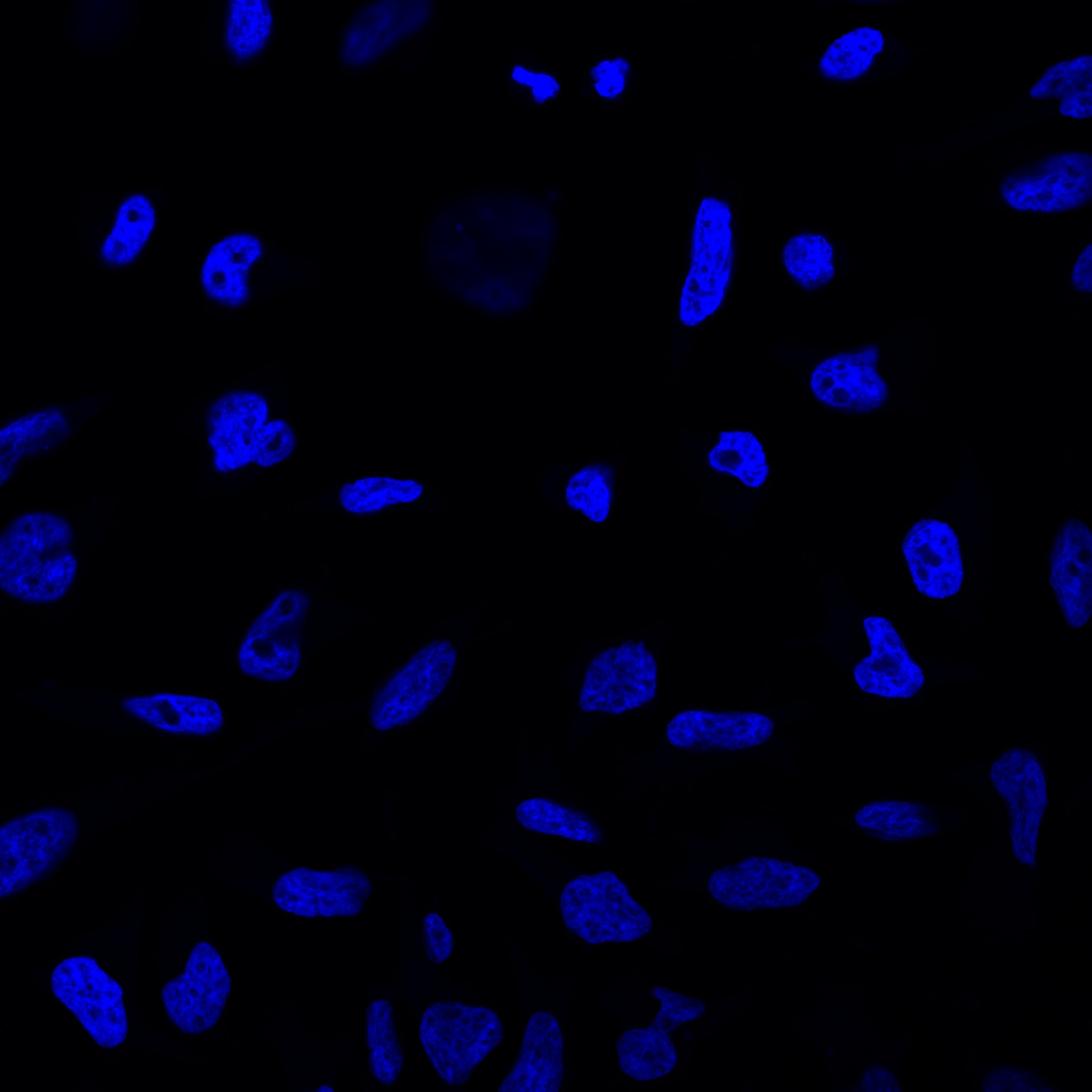

Supplement: Source Data Extended Data Fig. 3 — Microscopy images [file 41557_2022_972_MOESM9_ESM.zip › EDFig3_m7GG_DAPI.jpg]

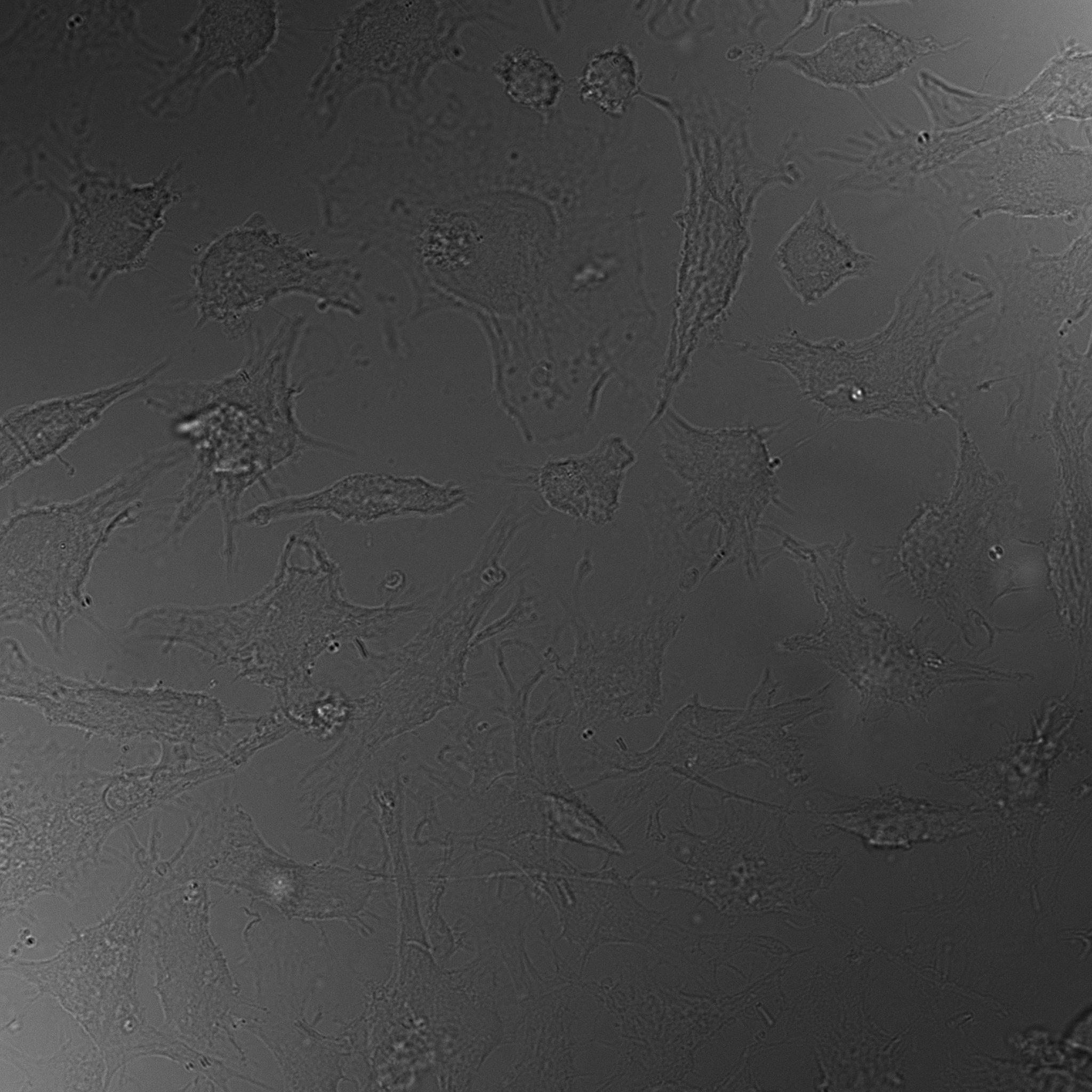

Supplement: Source Data Extended Data Fig. 3 — Microscopy images [file 41557_2022_972_MOESM9_ESM.zip › EDFig3_m7GG_DIC.jpg]

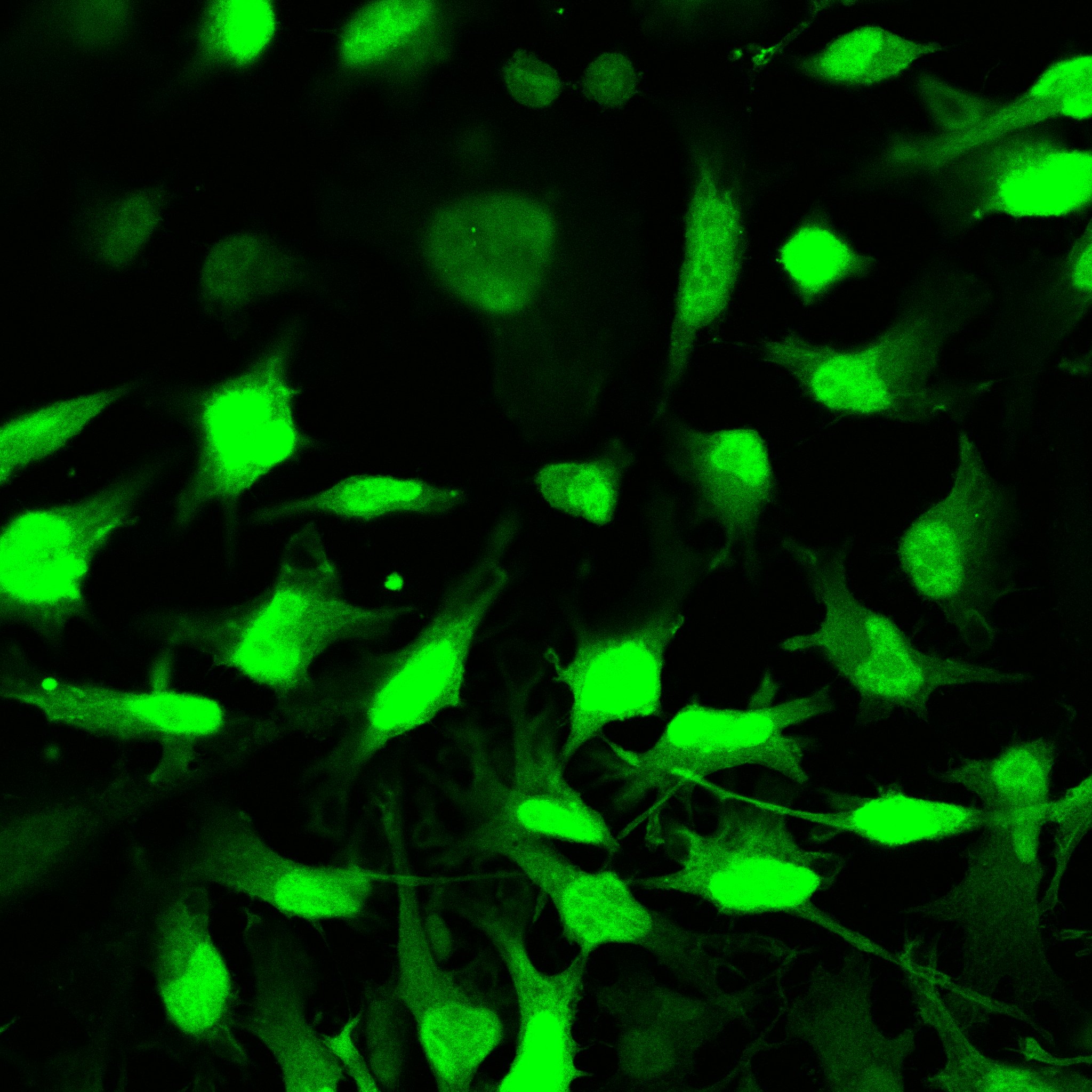

Supplement: Source Data Extended Data Fig. 3 — Microscopy images [file 41557_2022_972_MOESM9_ESM.zip › EDFig3_m7GG_eGFP.jpg]

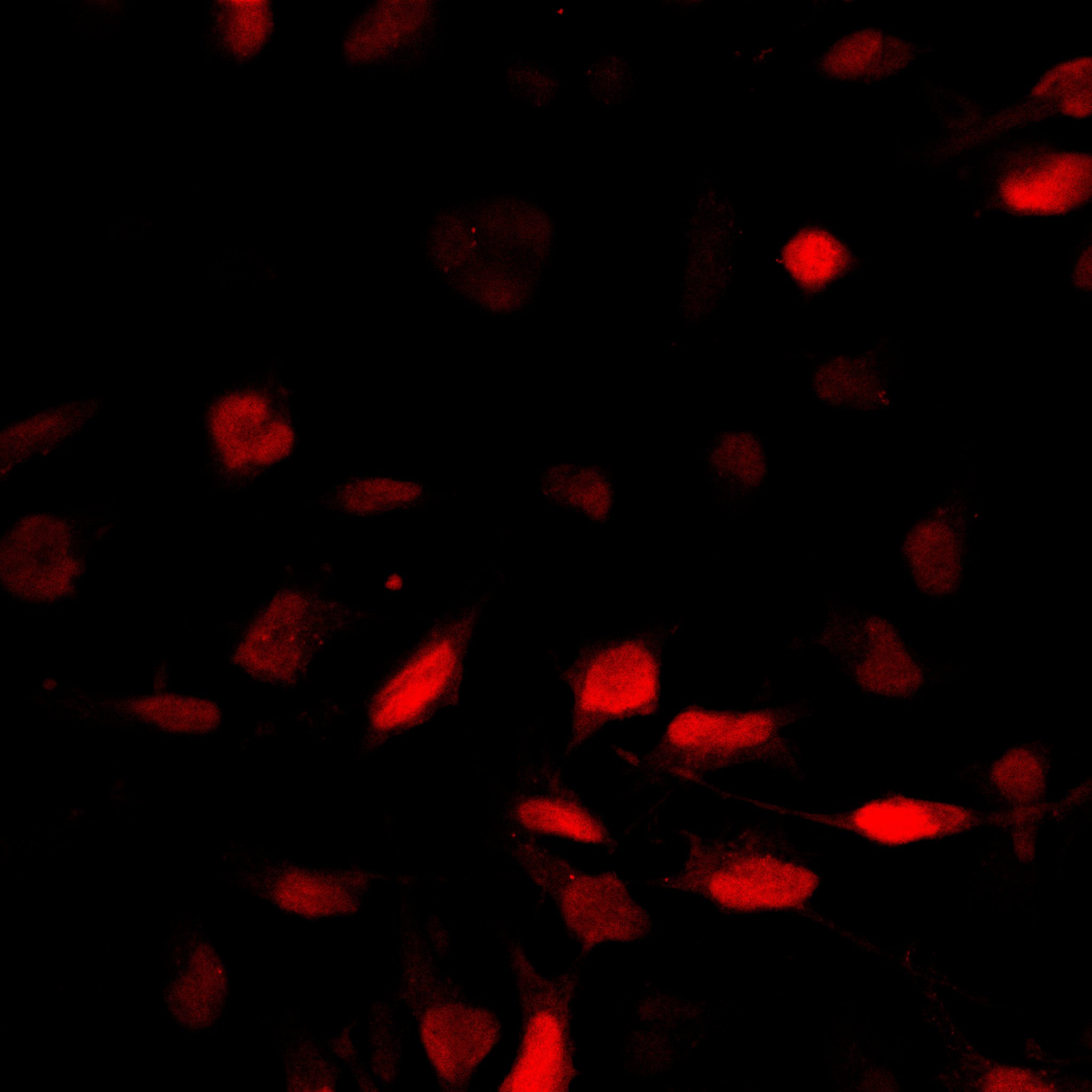

Supplement: Source Data Extended Data Fig. 3 — Microscopy images [file 41557_2022_972_MOESM9_ESM.zip › EDFig3_m7GG_scar.jpg]

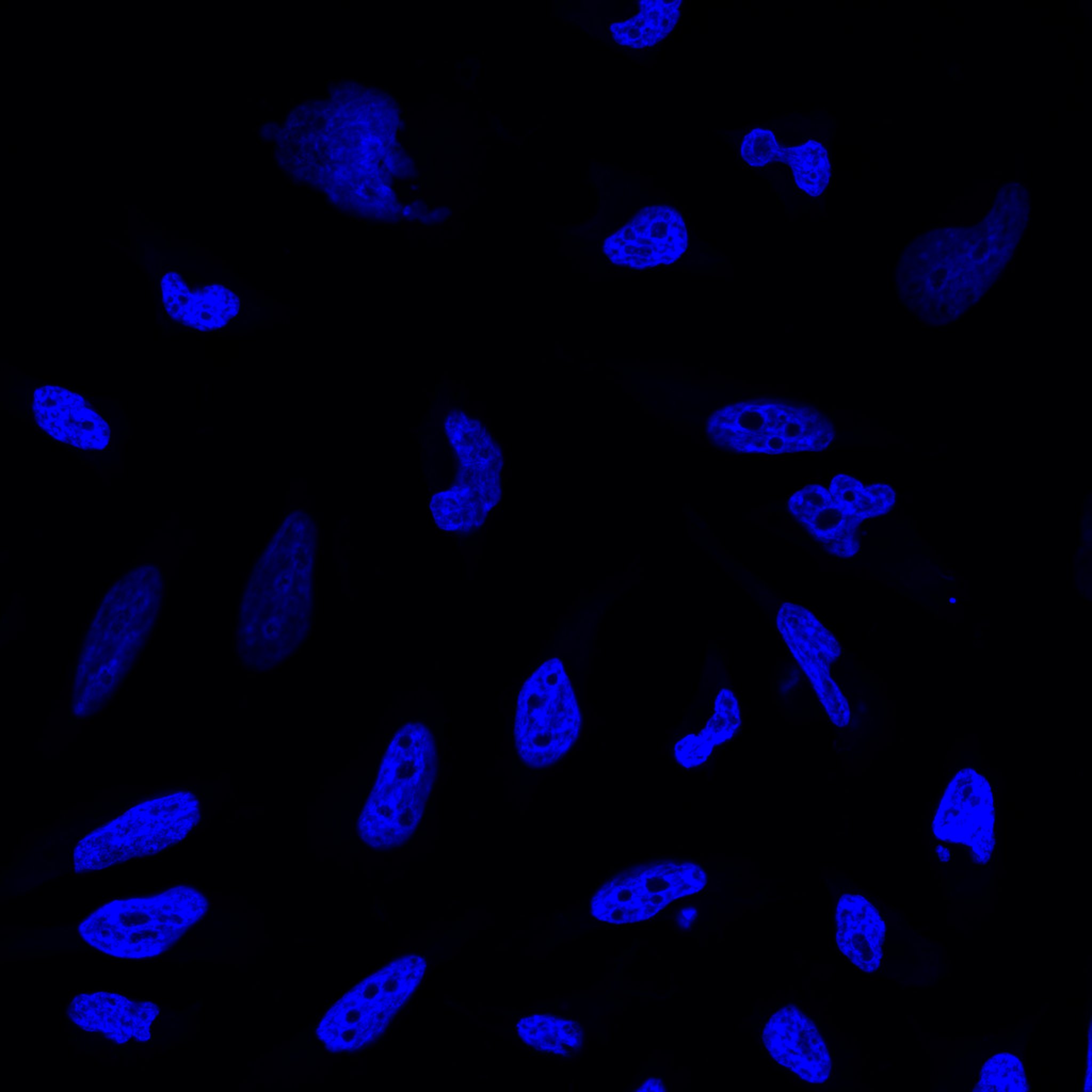

Supplement: Source Data Extended Data Fig. 3 — Microscopy images [file 41557_2022_972_MOESM9_ESM.zip › EDFig3_NPM_405_60s_DAPI.jpg]

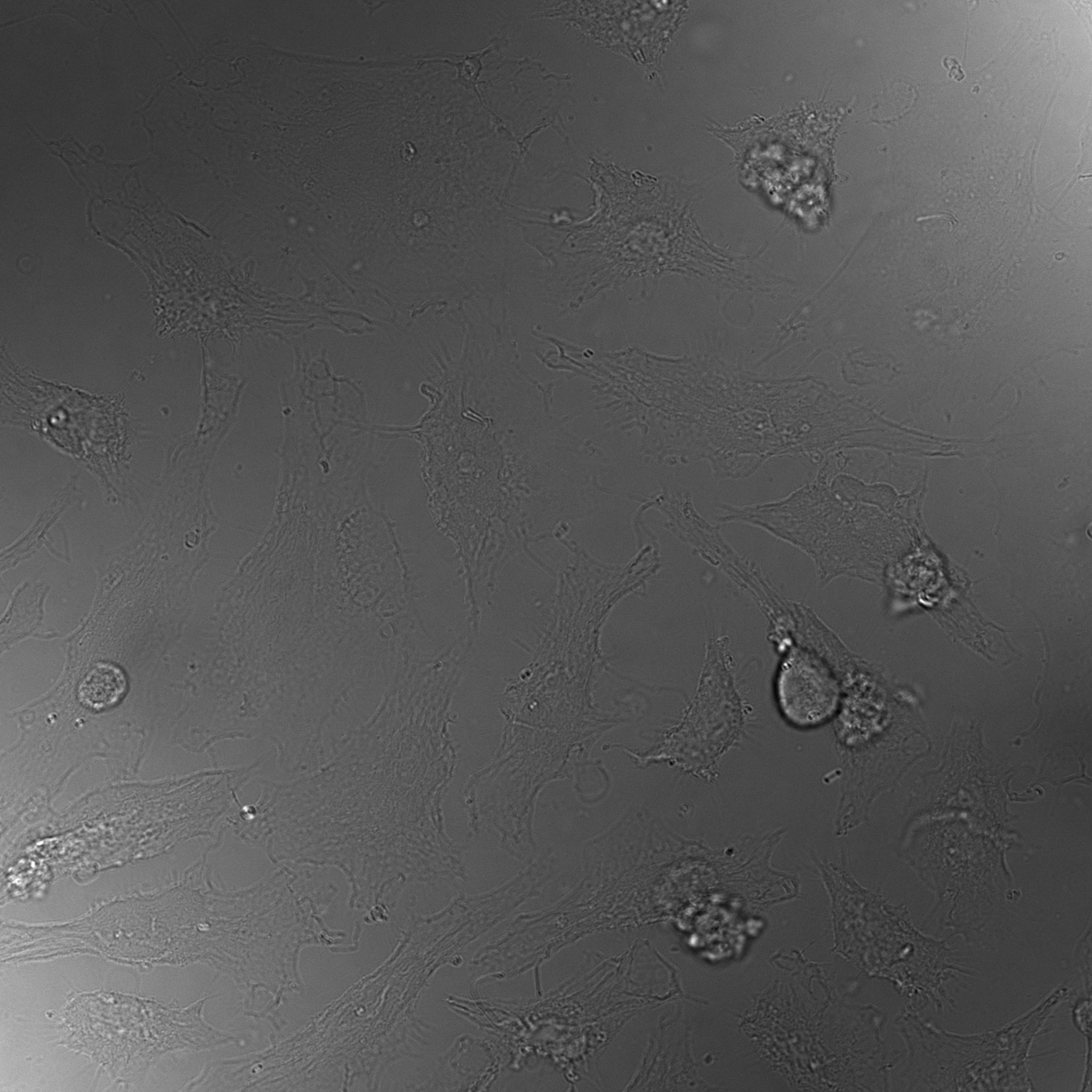

Supplement: Source Data Extended Data Fig. 3 — Microscopy images [file 41557_2022_972_MOESM9_ESM.zip › EDFig3_NPM_405_60s_DIC.jpg]

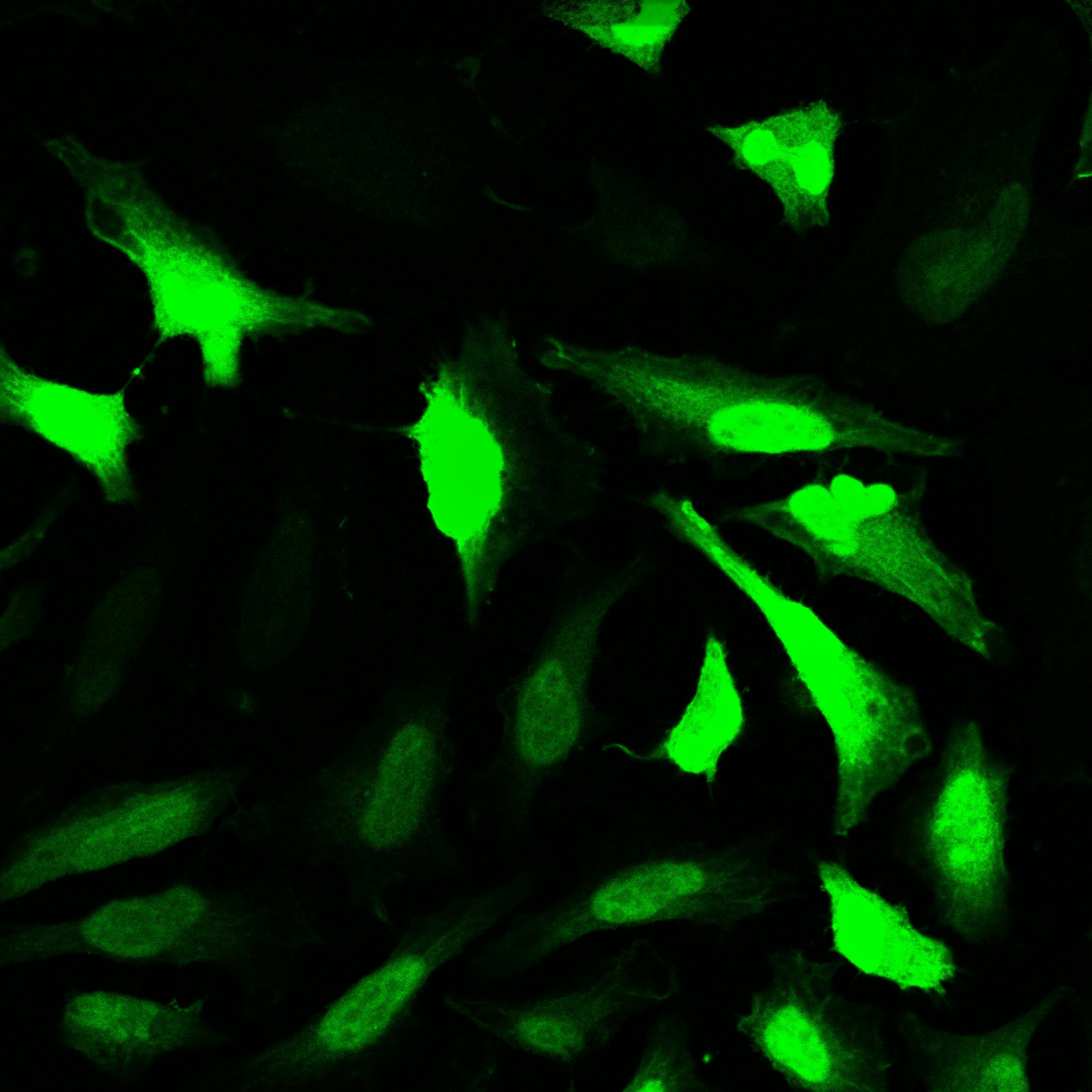

Supplement: Source Data Extended Data Fig. 3 — Microscopy images [file 41557_2022_972_MOESM9_ESM.zip › EDFig3_NPM_405_60s_eGFP.jpg]

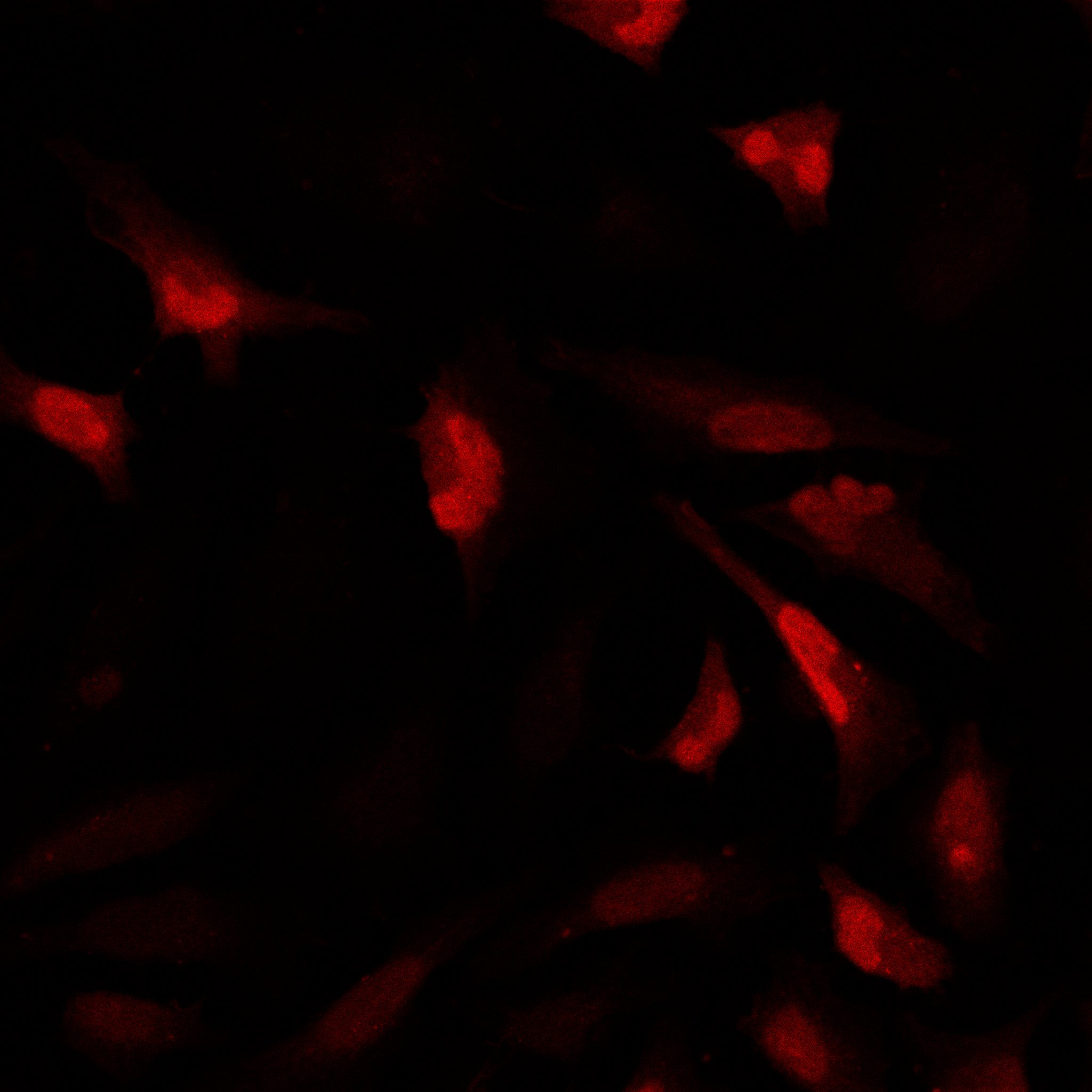

Supplement: Source Data Extended Data Fig. 3 — Microscopy images [file 41557_2022_972_MOESM9_ESM.zip › EDFig3_NPM_405_60s_scar.jpg]

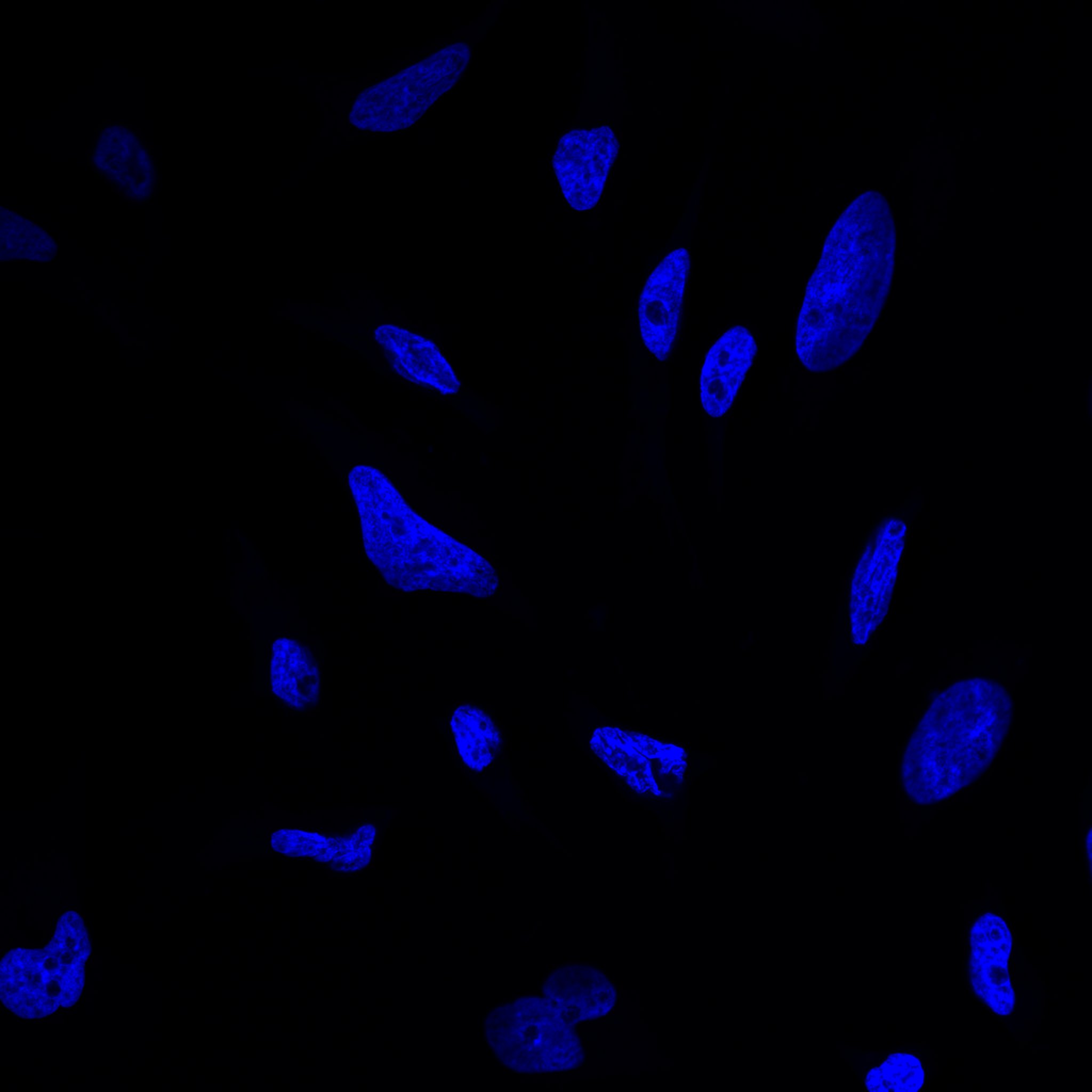

Supplement: Source Data Extended Data Fig. 3 — Microscopy images [file 41557_2022_972_MOESM9_ESM.zip › EDFig3_NPM_420_180s_DAPI.jpg]

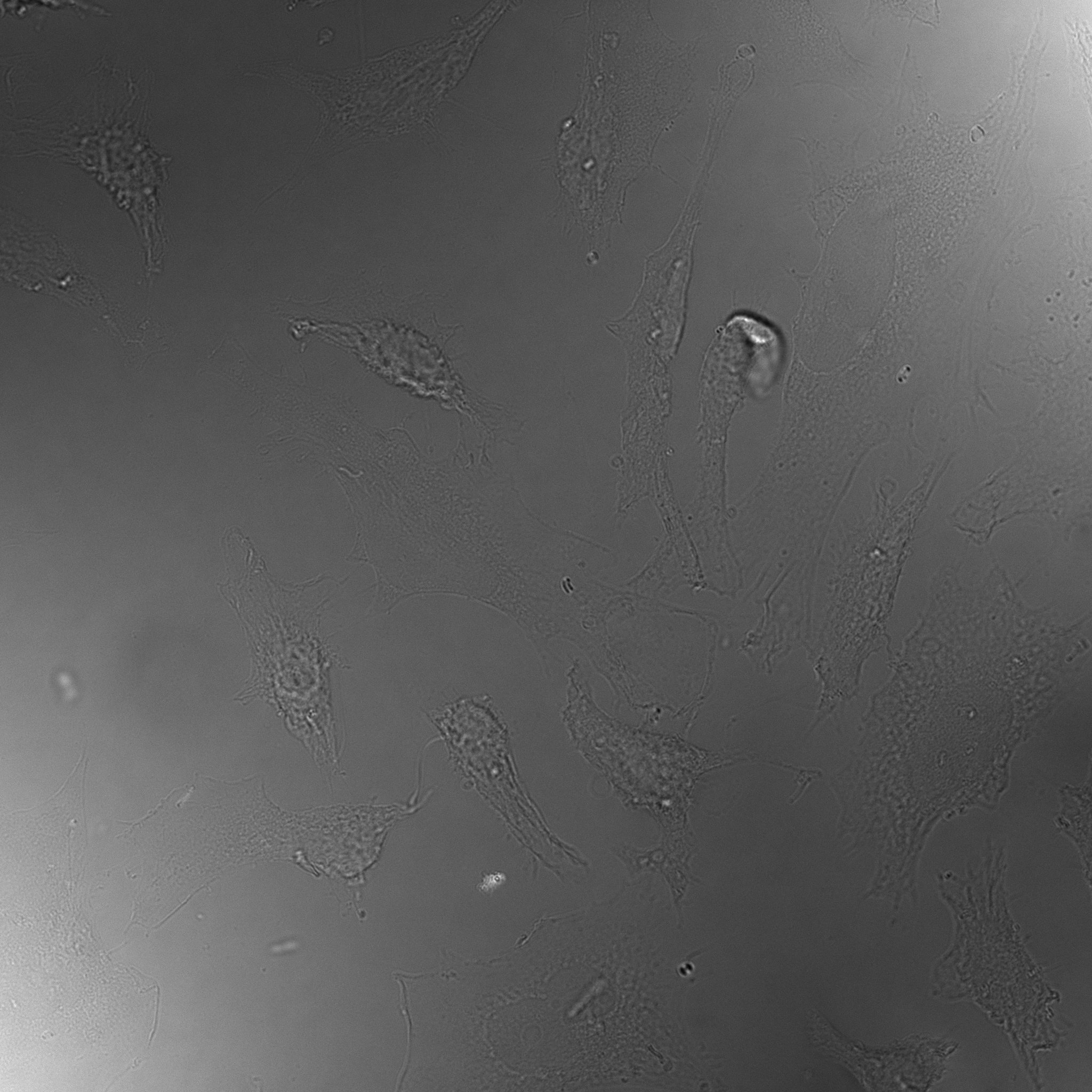

Supplement: Source Data Extended Data Fig. 3 — Microscopy images [file 41557_2022_972_MOESM9_ESM.zip › EDFig3_NPM_420_180s_DIC.jpg]

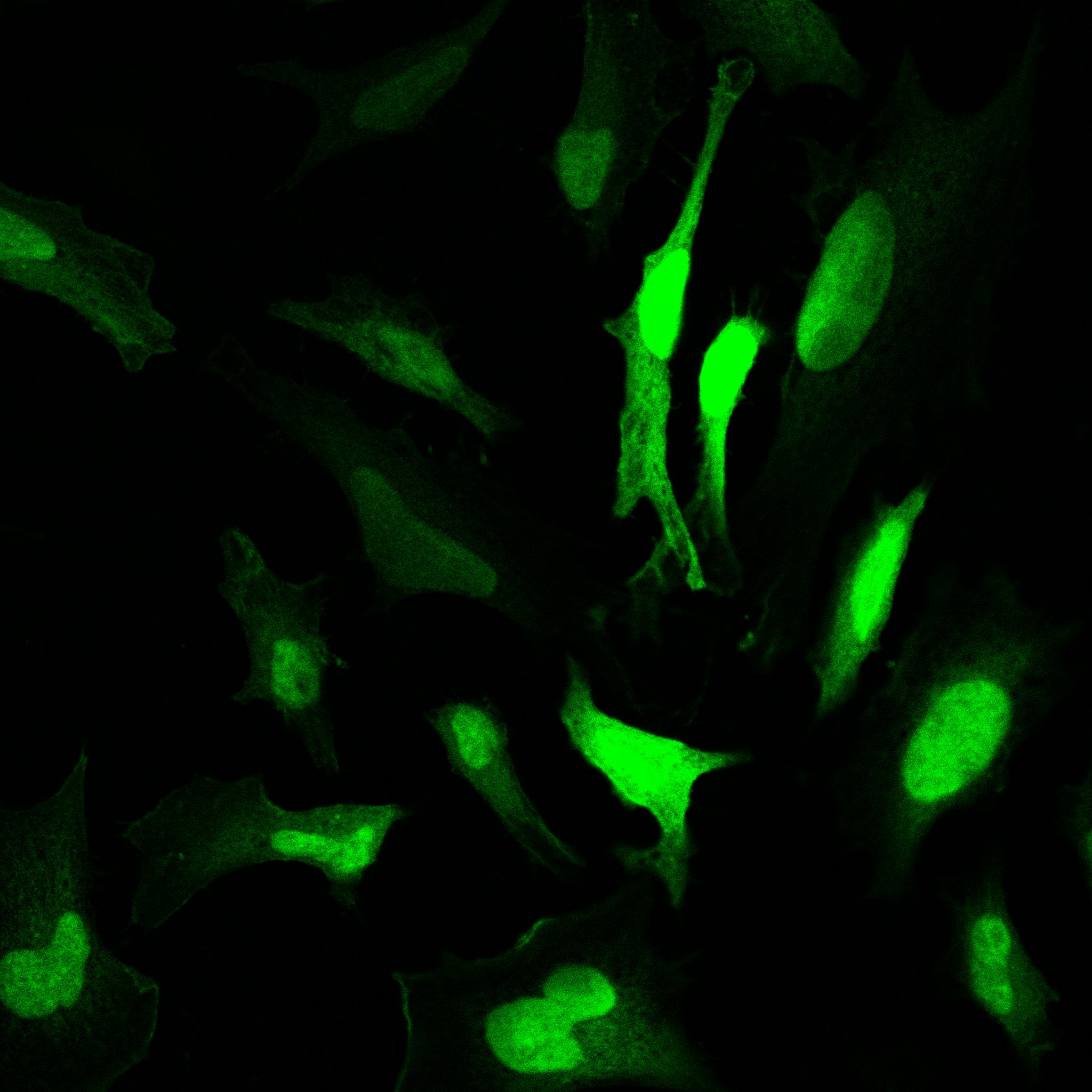

Supplement: Source Data Extended Data Fig. 3 — Microscopy images [file 41557_2022_972_MOESM9_ESM.zip › EDFig3_NPM_420_180s_eGFP.jpg]

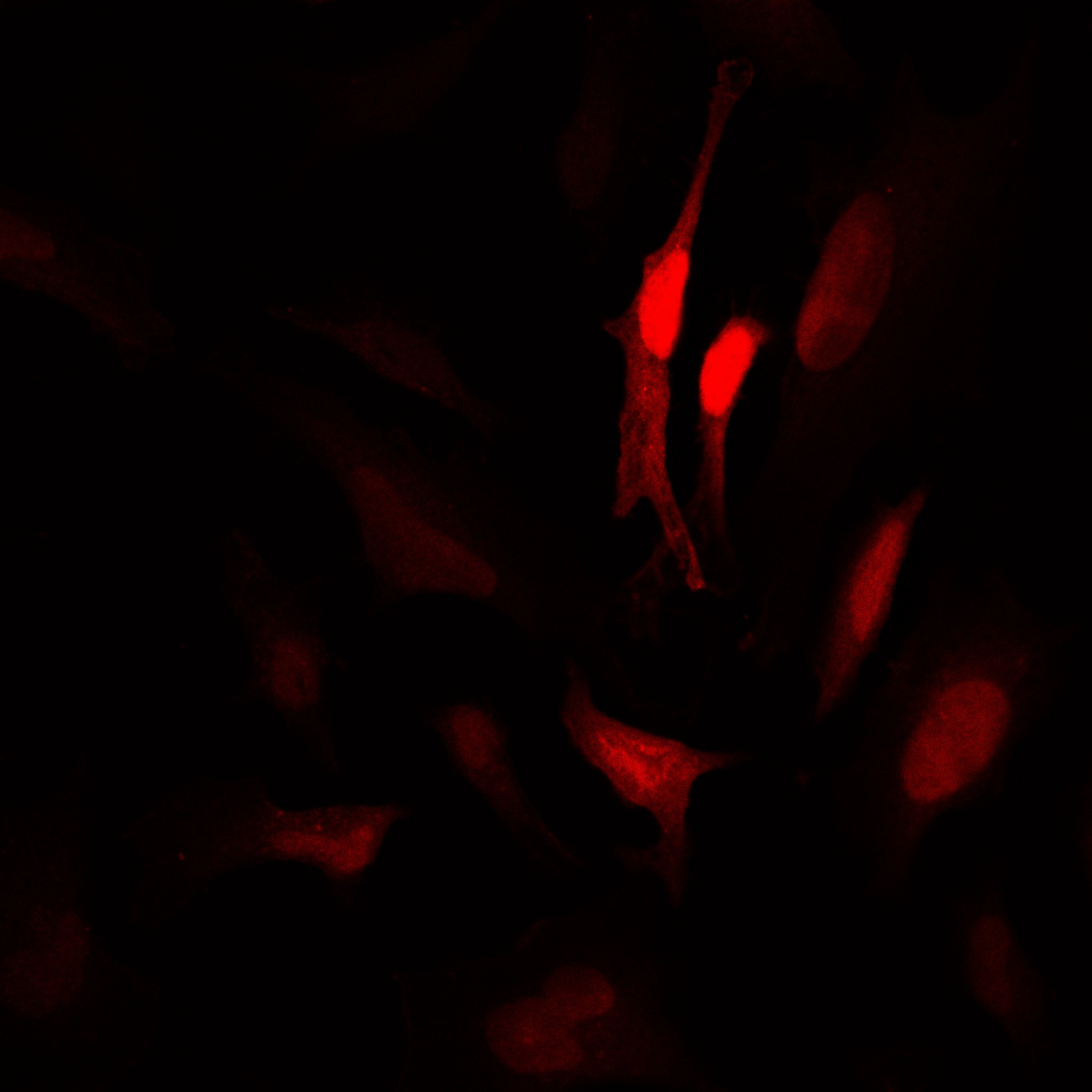

Supplement: Source Data Extended Data Fig. 3 — Microscopy images [file 41557_2022_972_MOESM9_ESM.zip › EDFig3_NPM_420_180s_scar.jpg]

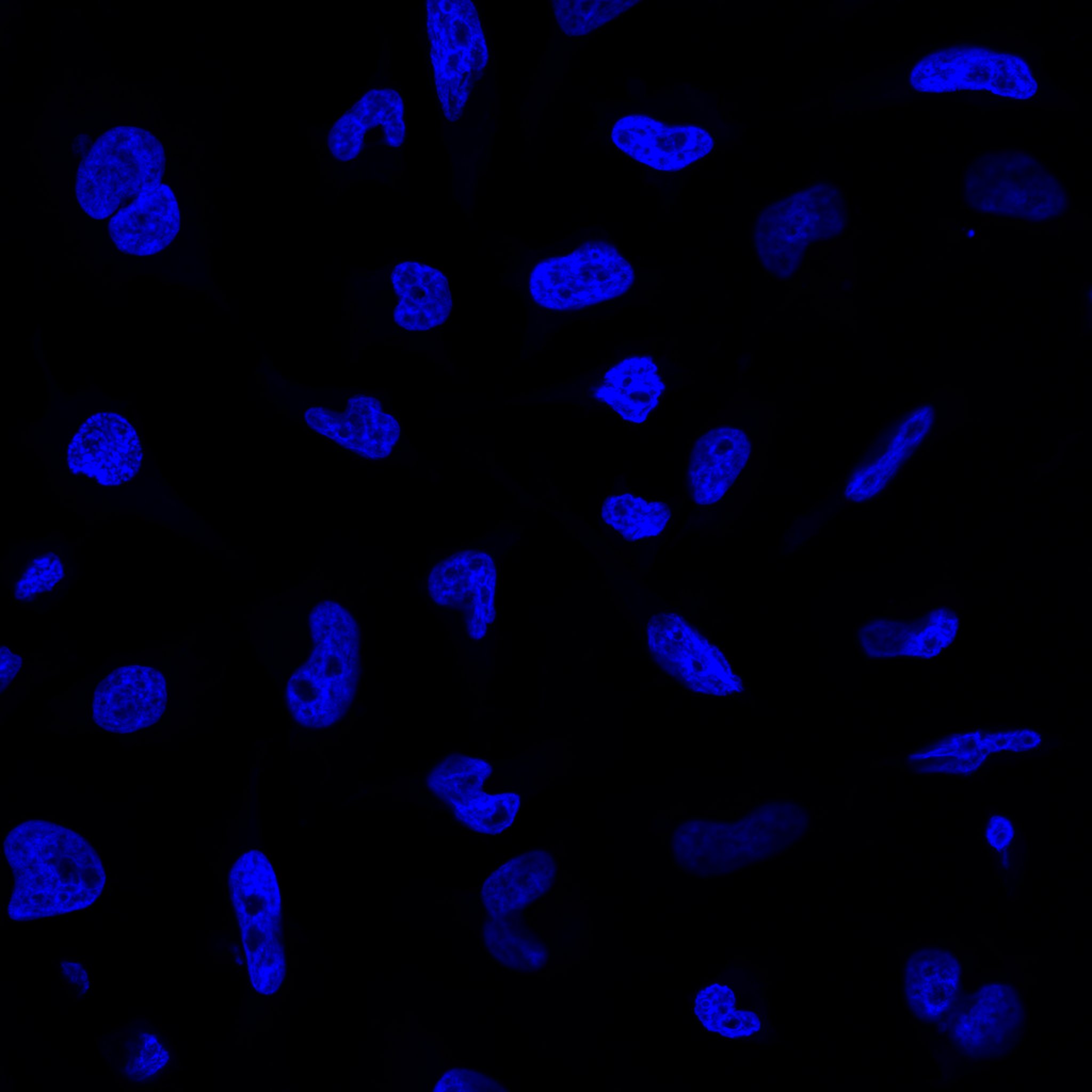

Supplement: Source Data Extended Data Fig. 3 — Microscopy images [file 41557_2022_972_MOESM9_ESM.zip › EDFig3_NPM_DAPI.jpg]

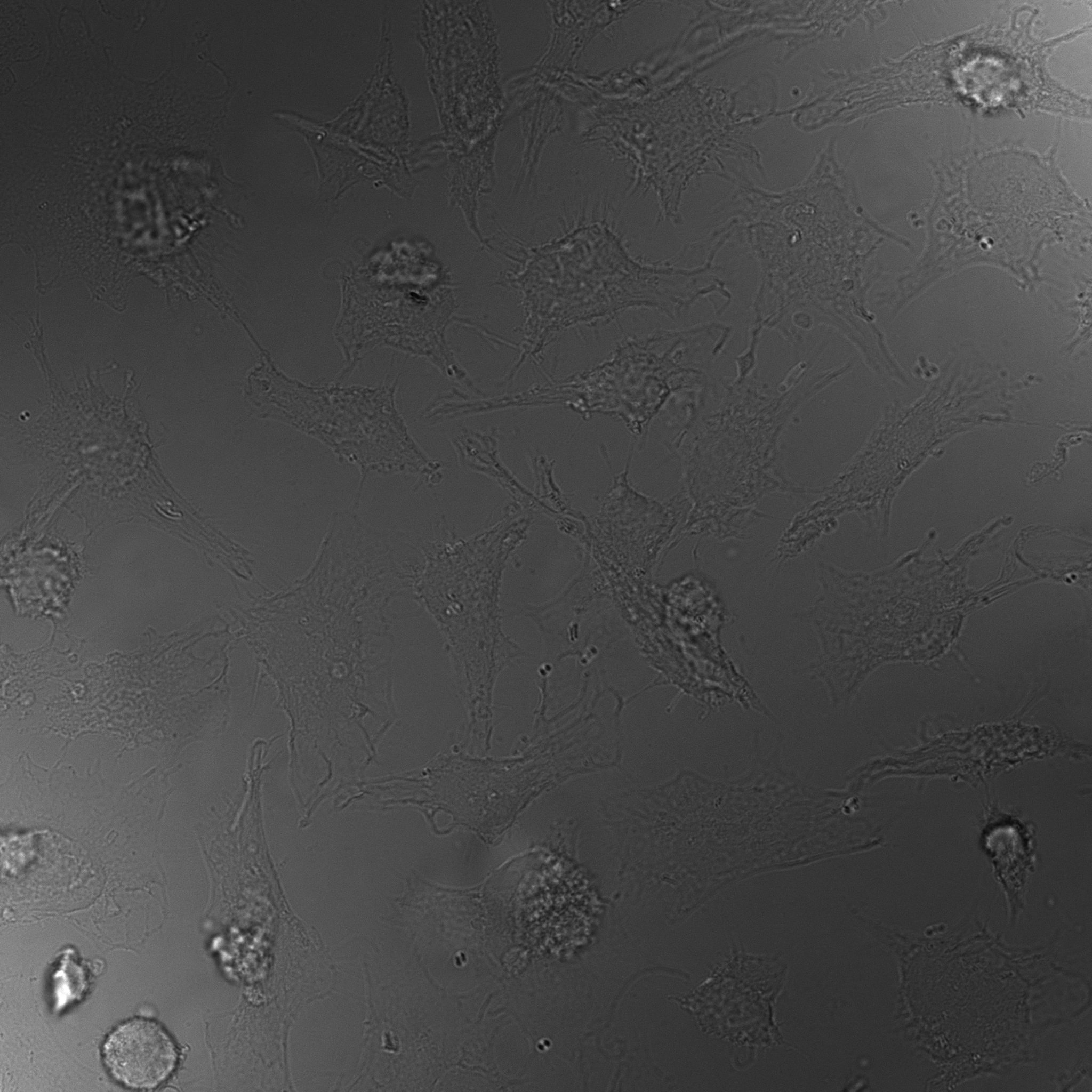

Supplement: Source Data Extended Data Fig. 3 — Microscopy images [file 41557_2022_972_MOESM9_ESM.zip › EDFig3_NPM_DIC.jpg]

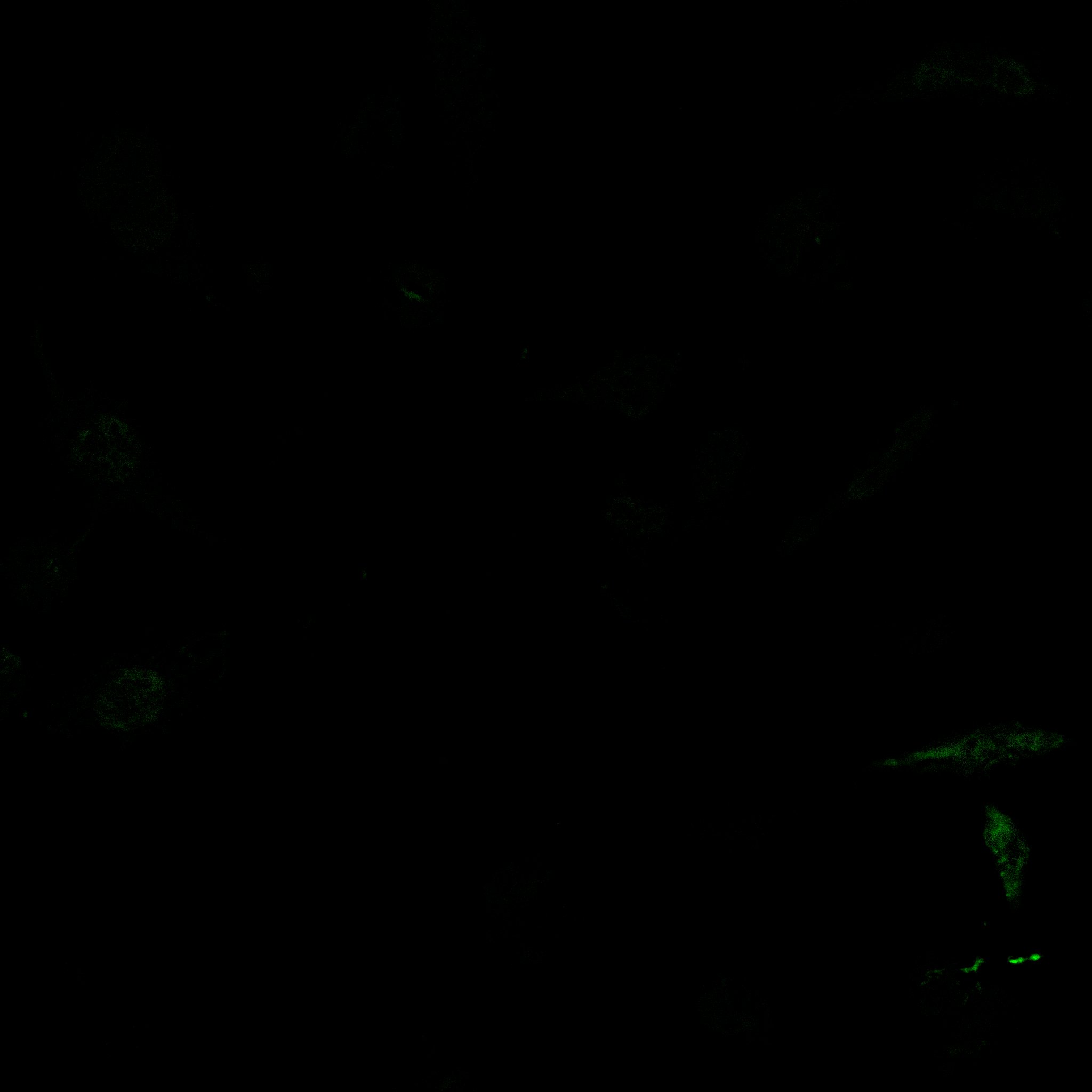

Supplement: Source Data Extended Data Fig. 3 — Microscopy images [file 41557_2022_972_MOESM9_ESM.zip › EDFig3_NPM_eGFP.jpg]

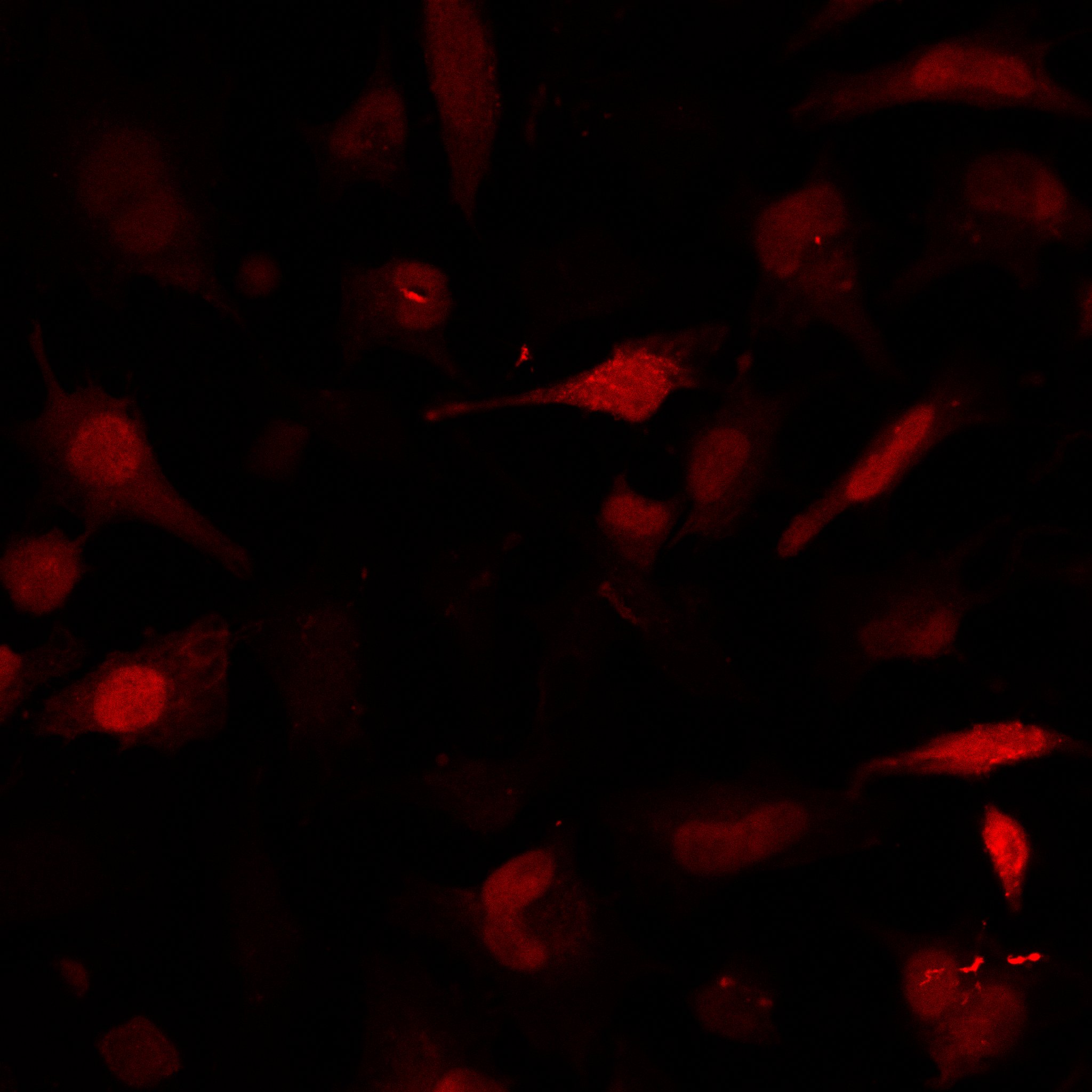

Supplement: Source Data Extended Data Fig. 3 — Microscopy images [file 41557_2022_972_MOESM9_ESM.zip › EDFig3_NPM_Scar.jpg]

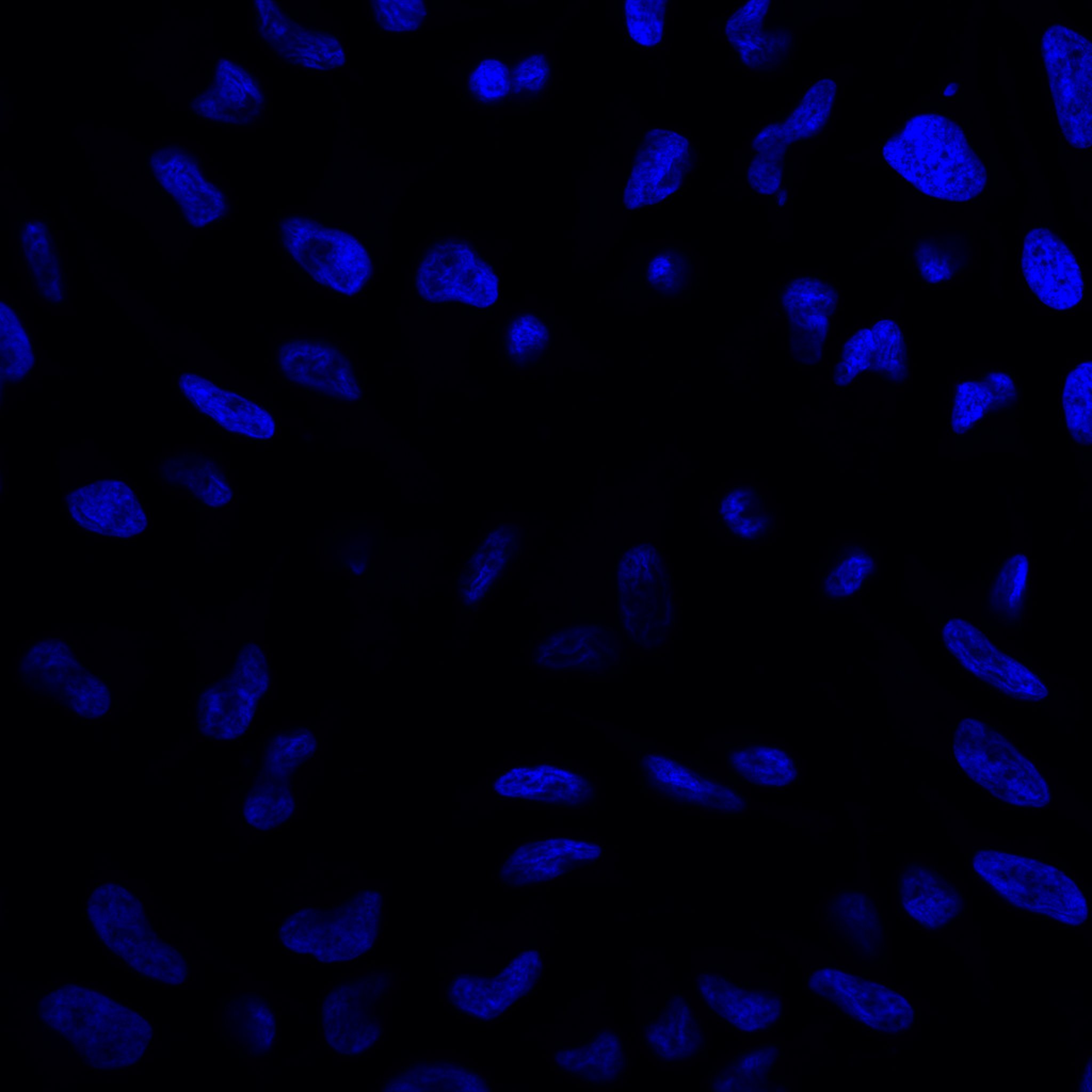

Supplement: Source Data Extended Data Fig. 3 — Microscopy images [file 41557_2022_972_MOESM9_ESM.zip › EDFig3_untransfected_DAPI.jpg]

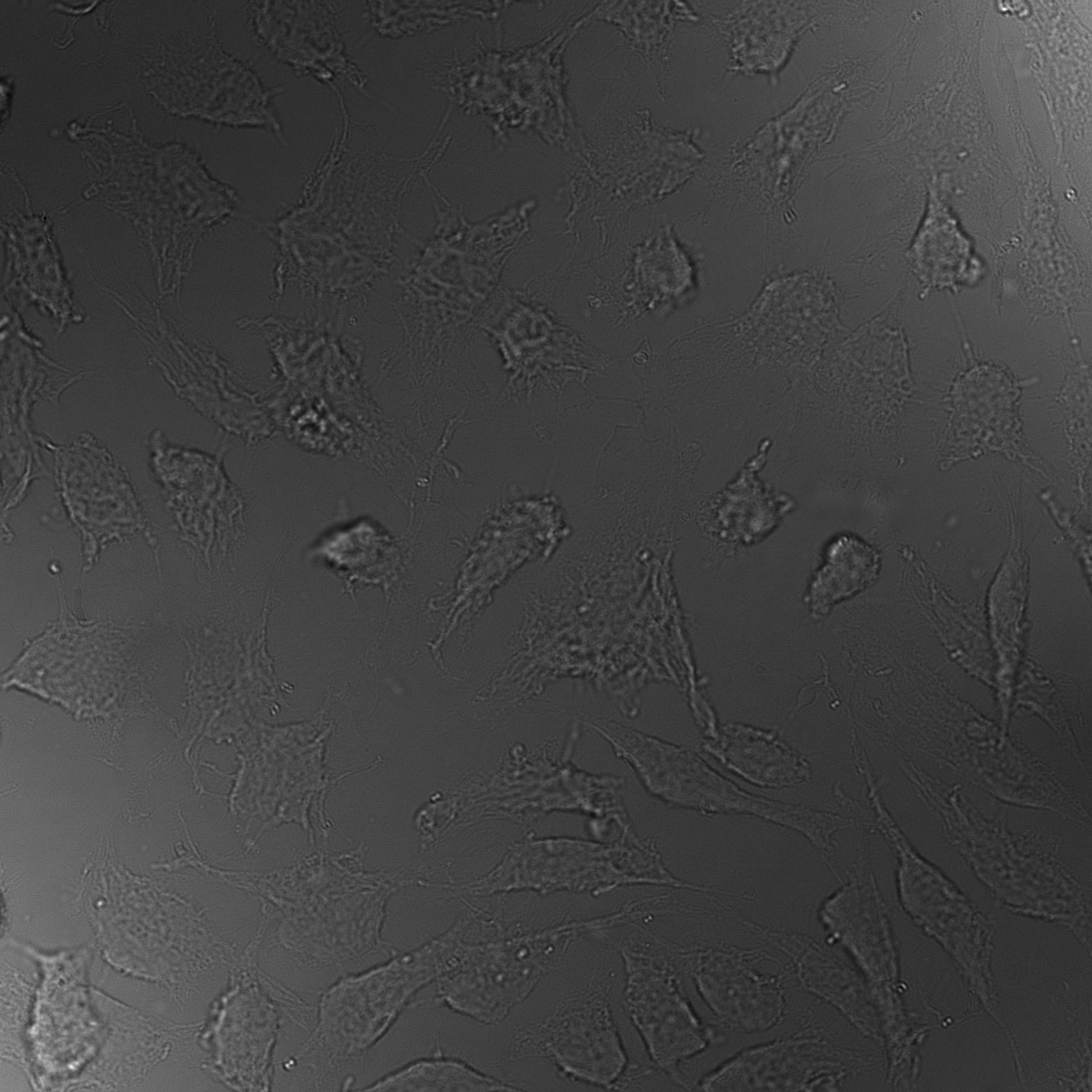

Supplement: Source Data Extended Data Fig. 3 — Microscopy images [file 41557_2022_972_MOESM9_ESM.zip › EDFig3_untransfected_DIC.jpg]

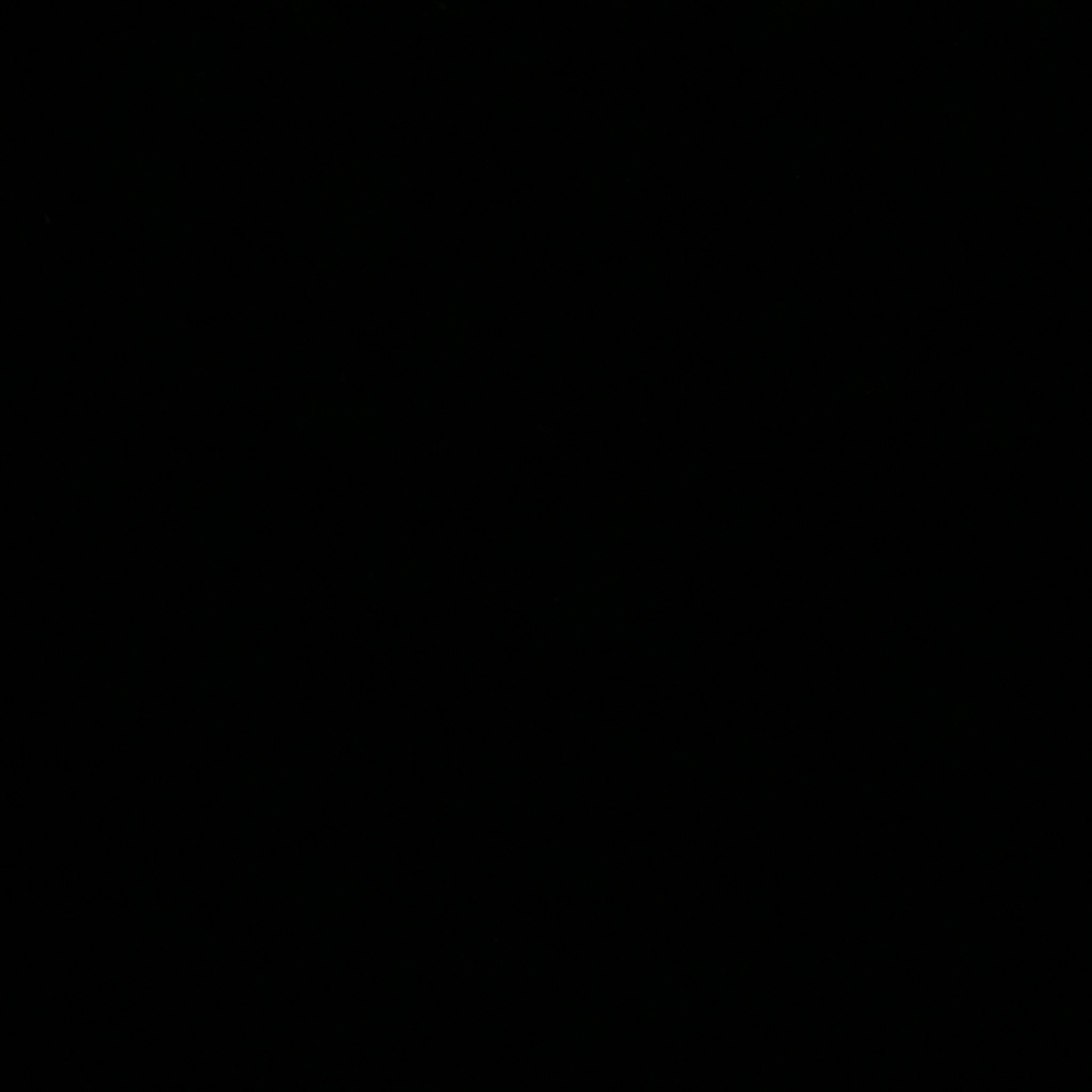

Supplement: Source Data Extended Data Fig. 3 — Microscopy images [file 41557_2022_972_MOESM9_ESM.zip › EDFig3_untransfected_eGFP.jpg]

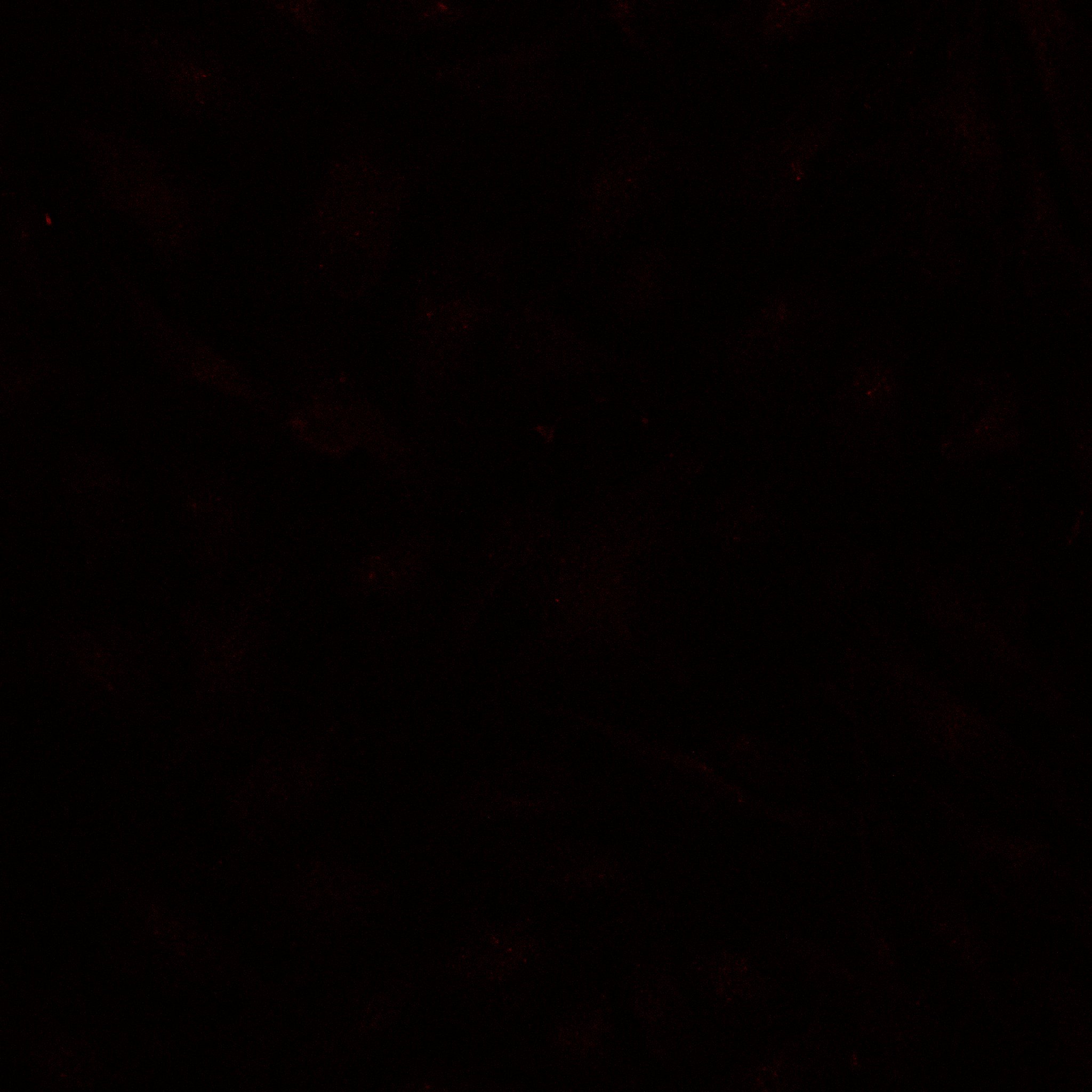

Supplement: Source Data Extended Data Fig. 3 — Microscopy images [file 41557_2022_972_MOESM9_ESM.zip › EDFig3_untransfected_scar.jpg]

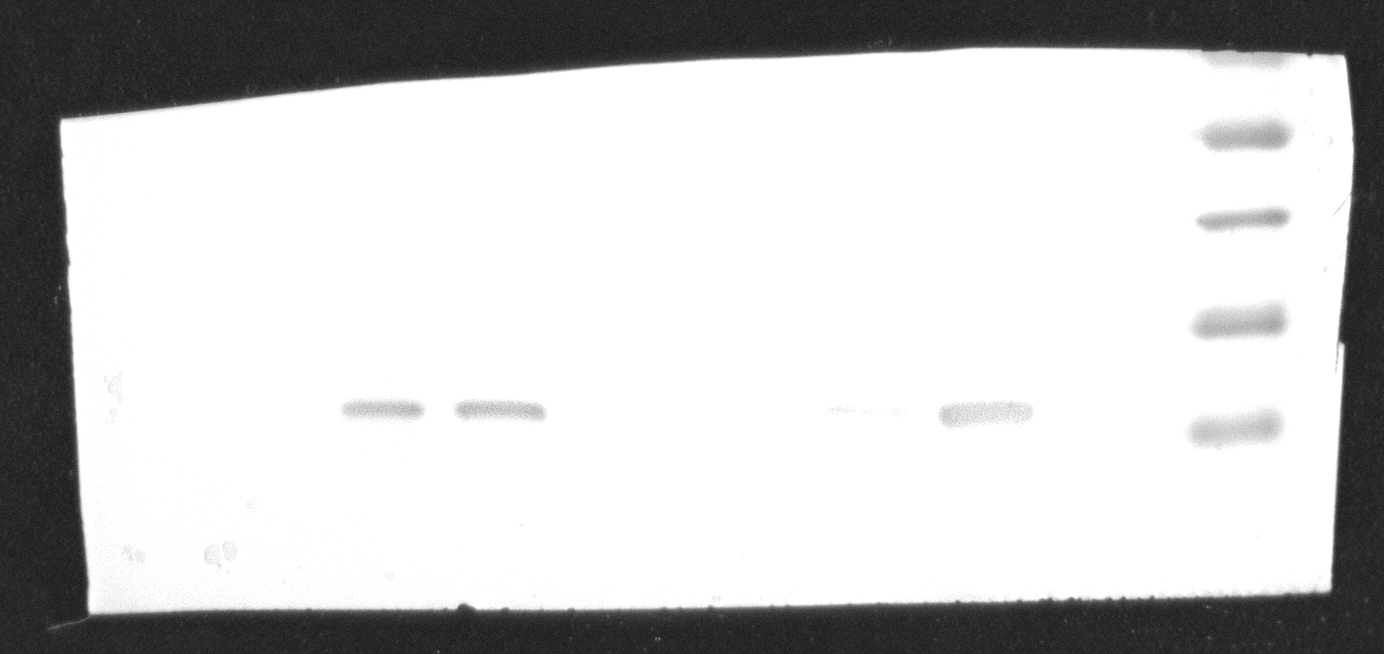

Supplement: Source Data Extended Data Fig. 4 — Unprocessed and uncropped western blots [file 41557_2022_972_MOESM10_ESM.zip › EDFigure4_HEK_eGFP.tif]

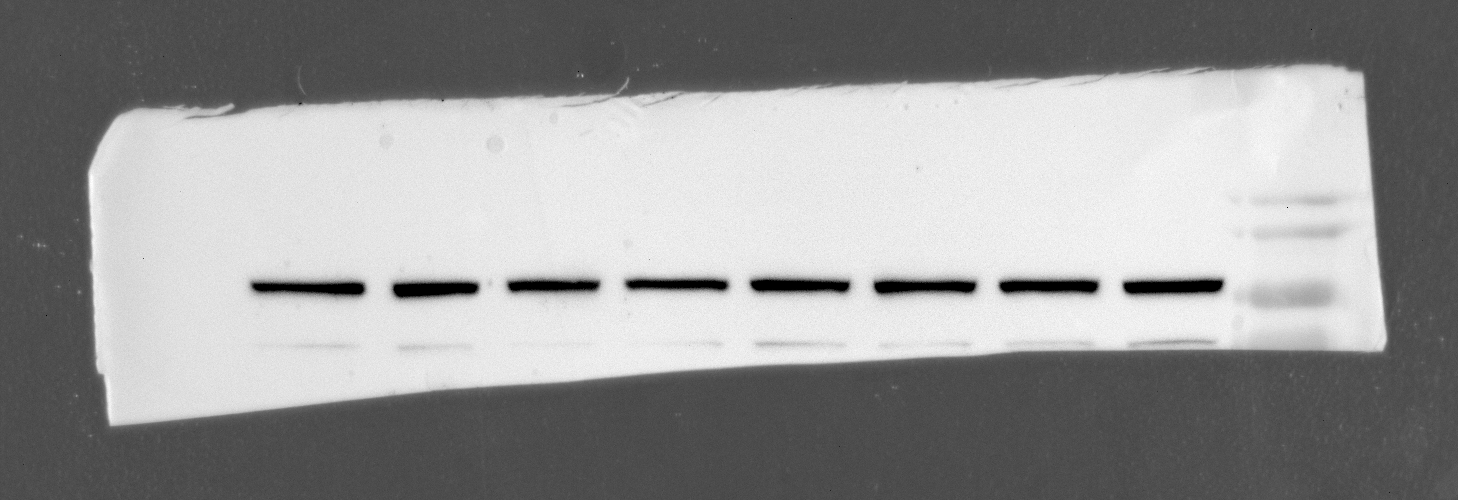

Supplement: Source Data Extended Data Fig. 4 — Unprocessed and uncropped western blots [file 41557_2022_972_MOESM10_ESM.zip › EDFigure4_HEK_eGFP_nucleoline.tif]

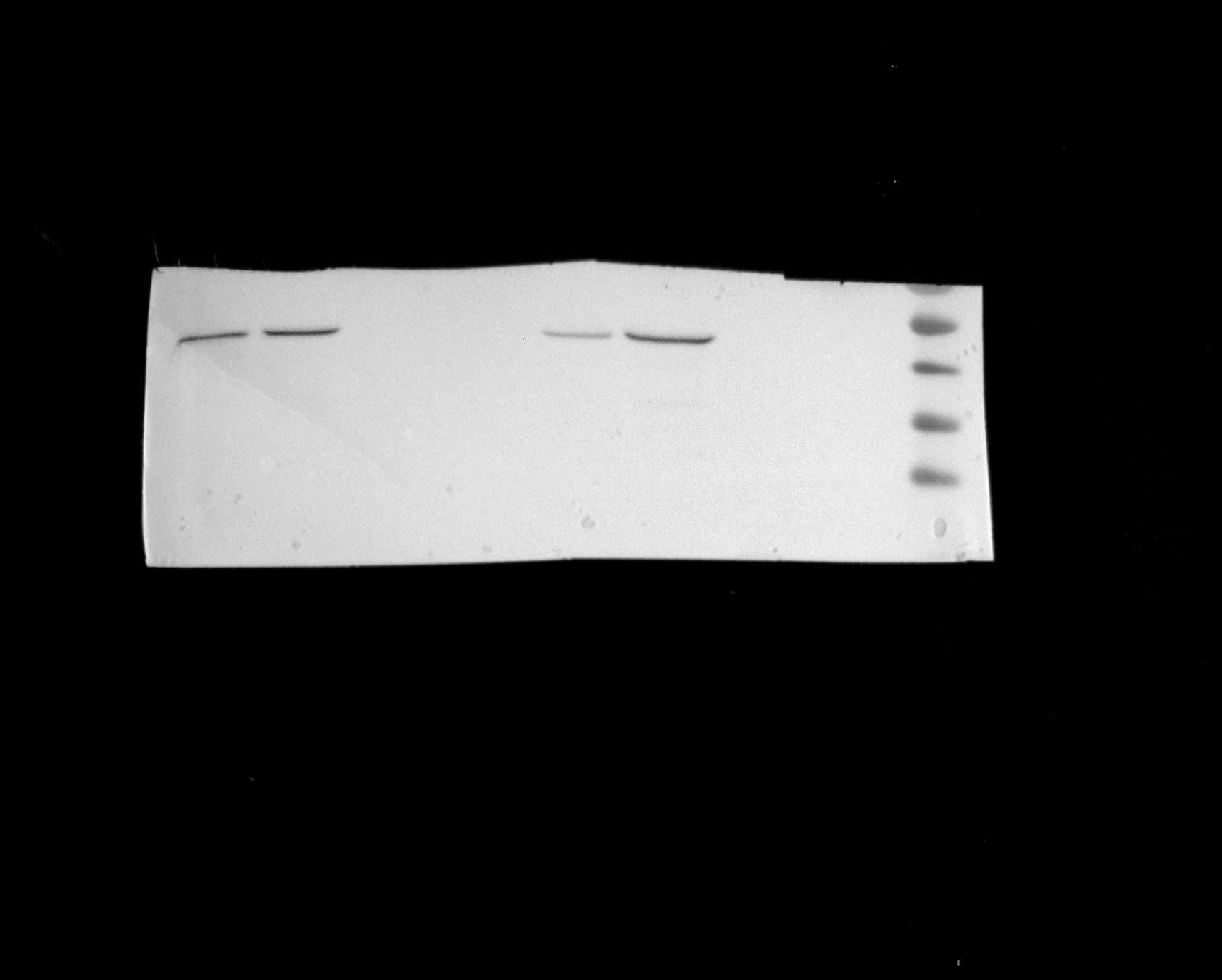

Supplement: Source Data Extended Data Fig. 4 — Unprocessed and uncropped western blots [file 41557_2022_972_MOESM10_ESM.zip › EDFigure4_HEK_Rheb_eGFP.tif]

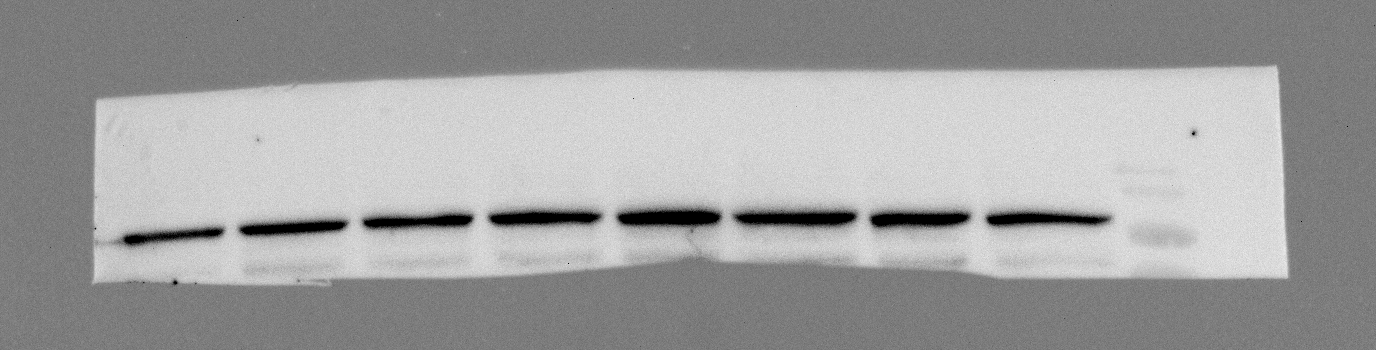

Supplement: Source Data Extended Data Fig. 4 — Unprocessed and uncropped western blots [file 41557_2022_972_MOESM10_ESM.zip › EDFigure4_HEK_Rheb_eGFP_nucleoline.tif]

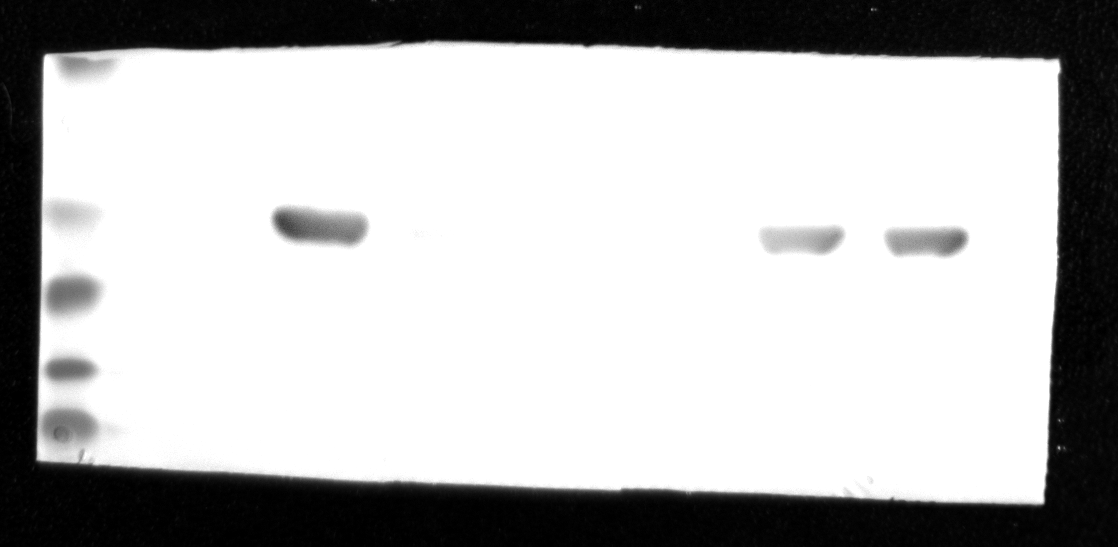

Supplement: Source Data Extended Data Fig. 4 — Unprocessed and uncropped western blots [file 41557_2022_972_MOESM10_ESM.zip › EDFigure4_HeLa_eGFP.tif]

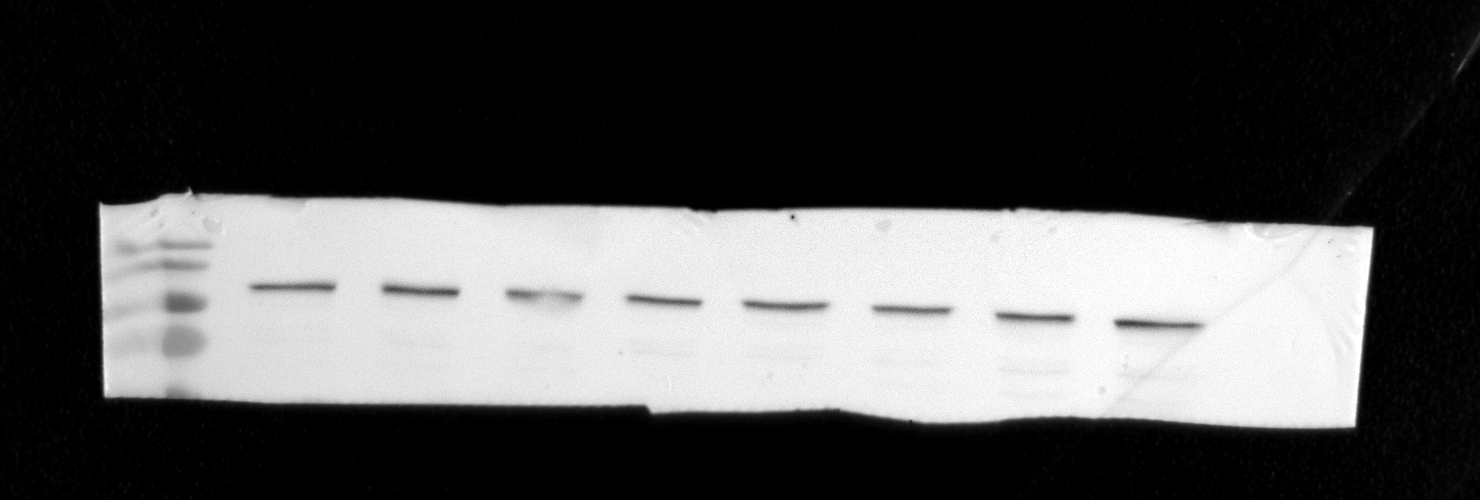

Supplement: Source Data Extended Data Fig. 4 — Unprocessed and uncropped western blots [file 41557_2022_972_MOESM10_ESM.zip › EDFigure4_HeLa_eGFP_nucleoline.tif]

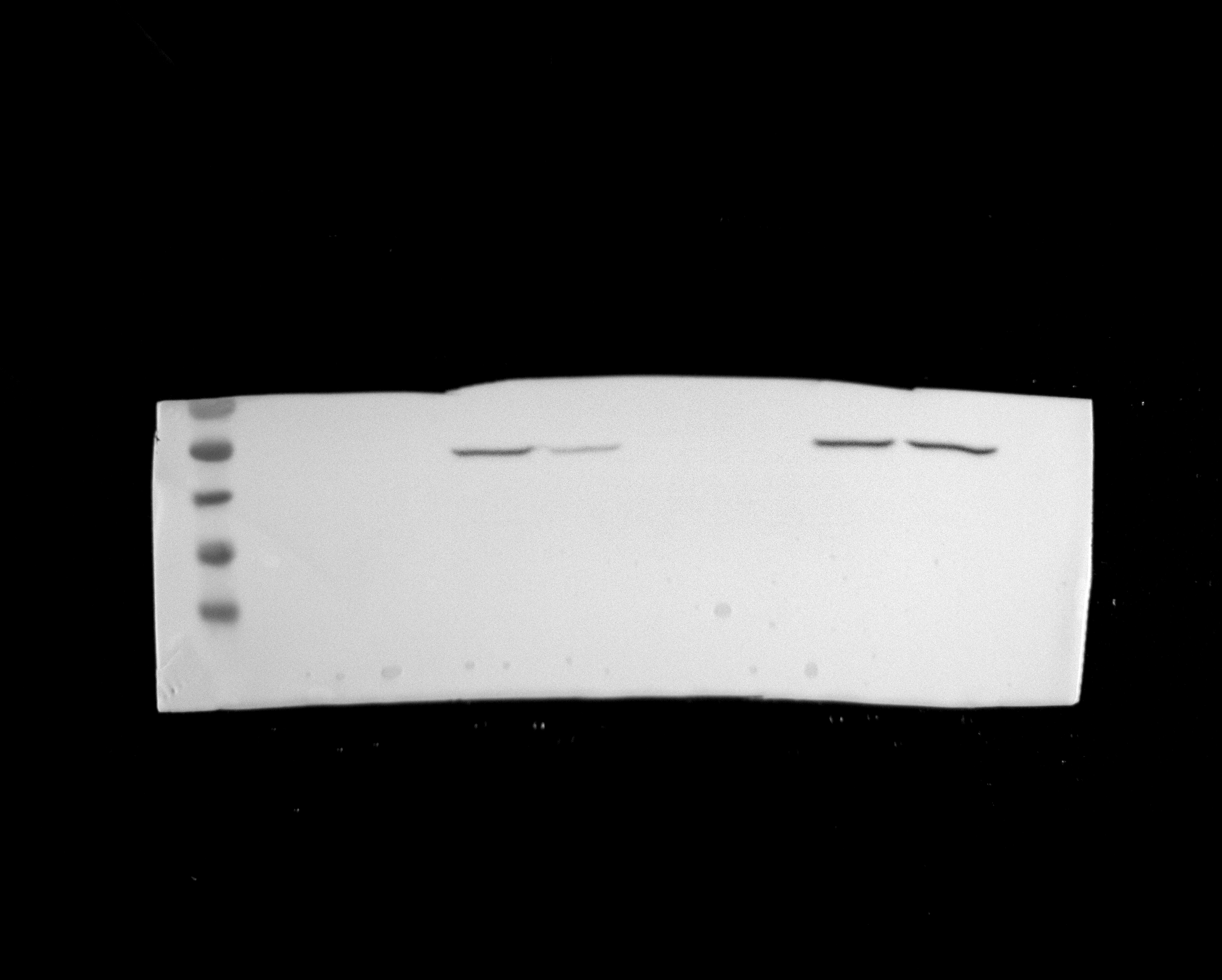

Supplement: Source Data Extended Data Fig. 4 — Unprocessed and uncropped western blots [file 41557_2022_972_MOESM10_ESM.zip › EDFigure4_HeLa_Rheb_eGFP.tif]

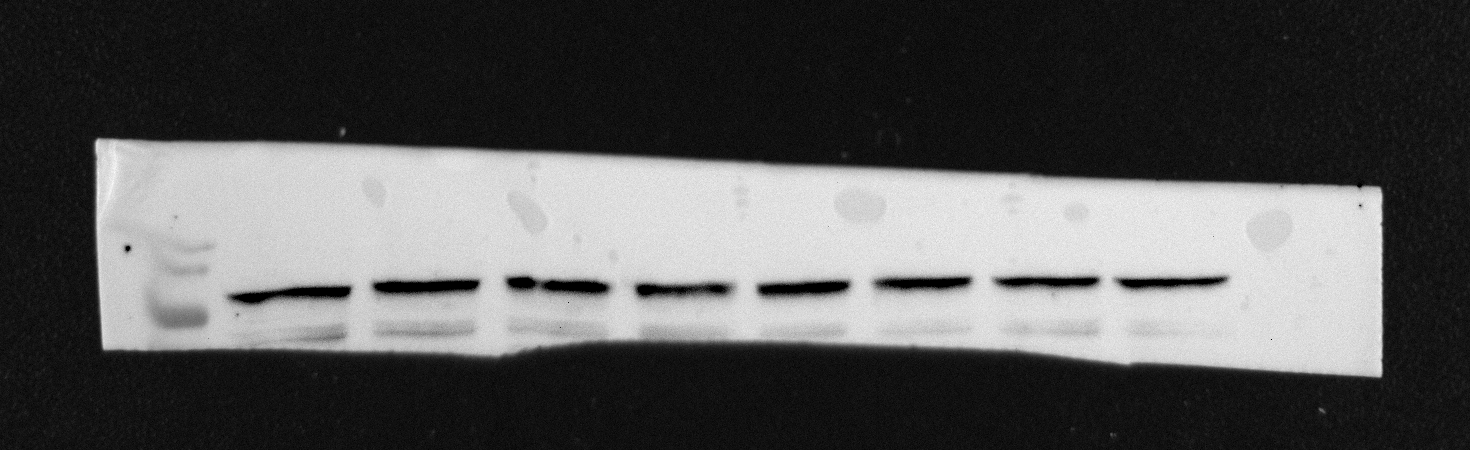

Supplement: Source Data Extended Data Fig. 4 — Unprocessed and uncropped western blots [file 41557_2022_972_MOESM10_ESM.zip › EDFigure4_HeLa_Rheb_eGFP_nucleoline.tif]
